# Flotillin scaffold activity contributes to type VII secretion system assembly in *Staphylococcus aureus*

**DOI:** 10.1371/journal.ppat.1006728

**Published:** 2017-11-22

**Authors:** Benjamin Mielich-Süss, Rabea M. Wagner, Nicole Mietrach, Tobias Hertlein, Gabriella Marincola, Knut Ohlsen, Sebastian Geibel, Daniel Lopez

**Affiliations:** 1 Research Center for Infectious Diseases ZINF, University of Würzburg, Würzburg, Germany; 2 Institute for Molecular Infection Biology IMIB, University of Würzburg, Würzburg, Germany; 3 National Center for Biotechnology, Consejo Superior de Investigaciones Científicas (CNB-CSIC), Madrid, Spain; 4 Rudolf Virchow Center - DFG Research Center for Experimental Biomedicine, University of Würzburg, Würzburg, Germany; University of Tubingen, GERMANY

## Abstract

Scaffold proteins are ubiquitous chaperones that promote efficient interactions between partners of multi-enzymatic protein complexes; although they are well studied in eukaryotes, their role in prokaryotic systems is poorly understood. Bacterial membranes have functional membrane microdomains (FMM), a structure homologous to eukaryotic lipid rafts. Similar to their eukaryotic counterparts, bacterial FMM harbor a scaffold protein termed flotillin that is thought to promote interactions between proteins spatially confined to the FMM. Here we used biochemical approaches to define the scaffold activity of the flotillin homolog FloA of the human pathogen *Staphylococcus aureus*, using assembly of interacting protein partners of the type VII secretion system (T7SS) as a case study. *Staphylococcus aureus* cells that lacked FloA showed reduced T7SS function, and thus reduced secretion of T7SS-related effectors, probably due to the supporting scaffold activity of flotillin. We found that the presence of flotillin mediates intermolecular interactions of T7SS proteins. We tested several small molecules that interfere with flotillin scaffold activity, which perturbed T7SS activity *in vitro* and *in vivo*. Our results suggest that flotillin assists in the assembly of *S*. *aureus* membrane components that participate in infection and influences the infective potential of this pathogen.

## Author summary

The recently discovered functional membrane microdomains (FMM) of prokaryotic cells contain a protein homologous to the scaffold protein flotillin found in eukaryotic lipid rafts. It remains to be elucidated whether, like their eukaryotic counterparts, flotillin homolog proteins have a scaffold function in bacteria. Here we show that the *Staphylococcus aureus* flotillin FloA acts as a scaffold protein, to promote more efficient assembly of membrane-associated protein interacting partners of multi-enzyme complexes. In a case study, we provide biochemical evidence that FloA participates in assembly of the Type VII secretion system and thus contributes to *S*. *aureus* infective potential. Targeted dispersion of FMM-related processes using anti-FMM molecules opens up new perspectives for microbial therapies to treat persistent *S*. *aureus* infections.

## Introduction

Scaffold proteins, which are responsible for tethering proteins and facilitating multi-enzymatic biological reactions [1–3], are found ubiquitously in eukaryotic and prokaryotic cells. These proteins were first identified in eukaryotic cells, where they have been traditionally studied. They play an important role in numerous signaling cascades, as they increase the interaction efficiency of signaling proteins by concentrating them locally and positioning kinases near their substrates [4,5]. Biological reactions are more efficient if scaffold proteins tether protein partners and facilitate interactions. This scaffold role might apply to both eukaryotic and prokaryotic cells, although the precise function in prokaryotes is not well understood. Several scaffold proteins have nonetheless been described in bacteria and their molecular mechanisms characterized. For instance, the scaffold UspC regulates the K^+^ uptake signaling cascade in *Escherichia coli* [6], and GraX is a scaffold that participates in a signaling transduction cascade in response to antibiotics in the Gram-positive bacterium *S*. *aureus* [7,8].

A new type of scaffold protein was recently found in association with bacterial cell membranes. These proteins are homologs of the so-called flotillin proteins that localize preferentially to membrane lipid rafts in eukaryotic cells [9–14]. Bacterial flotillins are found in discrete membrane regions termed functional membrane microdomains (FMM), which differ in lipid composition from the rest of the membrane and spatially confine several proteins involved in signal transduction (sensor kinases), protein trafficking (ABC transporters and protein secretion machineries) and other multi-protein enzymatic reactions [15,16]. FMM thus resemble the lipid rafts of eukaryotic cells in certain organizational and functional aspects [17]. Flotillin scaffold activity in eukaryotic lipid rafts centers on recruiting raft-associated proteins to the rafts and catalyzing more efficient interaction or oligomerization [18–22]. Bacterial flotillins probably have a similar role, and their scaffold activity might also facilitate more efficient interactions and oligomerization of protein partners within FMM [3,23]. The biological significance of bacterial flotillins is nonetheless incompletely understood [24,25].

The Gram-positive bacterium *Bacillus subtilis* is currently the best-established model for study of the physiological relevance of flotillins and FMM [9–14]. *B*. *subtilis* FMM contain two flotillin-like proteins, FloT and FloA, which interact physically and recruit various proteins to the FMM. Strains lacking both of these flotillins show altered function of FMM-associated protein complexes, such as sensor kinase dimerization [25], FtsH-mediated protease activity [13], and protein secretion via Sec machinery [9]. Current research links flotillin activity and correct function of virulence-related cell processes in Gram-positive and -negative bacteria. The Gram-positive pathogen *Bacillus anthracis* expresses a flotillin homolog, FlotP, that is structurally similar to *B*. *subtilis* FloT (~65% identity) [26]. Subcellular FlotP localization is associated with membrane integrity, and alteration of its distribution correlates with a decrease in toxin secretion. In Gram-negative pathogens, a flotillin-defective *Campylobacter jejuni* strain is unable to adhere to or be internalized by epithelial cells, resulting in impaired virulence [27]; experiments in mice showed that this mutant did not cause campylobacteriosis [28]. Despite the number of examples that correlate flotillin activity with correct function of cell processes in bacteria, a precise molecular description of how flotillin contributes to the activation of these processes has yet to be elucidated.

**Fig 1:**
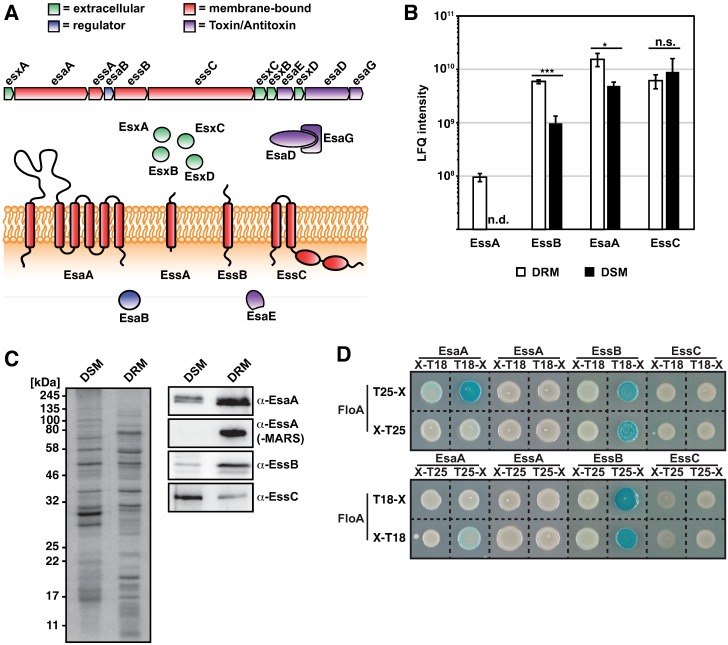
Type 7 secretion system proteins are associated with the DRM in *S*. *aureus*. (A) Organization of the T7SS operon (top) and of proteins in the cell (bottom). Small, secreted Esx-proteins are green; membrane proteins, red; genetic regulators, blue; toxin-antitoxin components, purple. (B) Label-free quantification (LFQ) of DRM and DSM proteins in stationary growth phase. Graph shows LFQ intensities of EsaA, EssA, EssB and EssC in DRM and DSM fractions. The experiment was performed using three biological replicates (*n* = 3). Statistical analysis was carried out using unpaired Student’s t-test, (*P<0.05; ***P<0.001). n.s. = not significant. n.d. = not detected. (C) Right, immunoblot analysis using anti-EsaA, -EssB, -EssC and -mCherry in DSM and DRM fractions. Left, Coomassie blue-stained gel of DSM and DRM fractions used as loading control. (D) Bacterial two-hybrid analysis to test interaction (blue) of T7SS membrane proteins with flotillin (FloA). FloA and T7SS membrane proteins EsaA, EssA, EssB and EssC were fused both C- and N-terminally with T18- and T25-fragments of an adenylate cyclase. *E*. *coli* bearing the two combinations were plated on X-Gal plates.

The flotillin of the human pathogen *S*. *aureus* is 84% identical to *B*. *subtilis* FloA [12]. Inhibition of flotillin activity interferes with oligomerization of FMM-associated proteins in other bacterial models, and flotillin-lacking pathogenic strains show attenuated virulence. Such inhibition could thus be a strategy by which to disable virulence-related protein complexes in *S*. *aureus*, which is currently a major problem in both clinical and community settings [29]. MRSA (methicillin-resistant *S*. *aureus*) invasive infections are difficult to treat and have a ~20% mortality rate in clinical contexts [30]. The ESAT-6 or type VII secretion system (T7SS) is a membrane-bound protein complex with a role in *S*. *aureus* virulence [31–33]. This system mediates formation of persistent abscesses, modulates immune responses and is involved in interspecies competition [31–35]. It consists of four membrane-bound proteins (EsaA, EssA, EssB, EssC), a cytosolic regulator (EsaB) and several secreted effectors [31–34,36] (Fig 1A): EsaE, D and G encode a toxin-antitoxin system [34,35], and EsxA-EsxD are small secreted proteins (up to 15 kDa) with roles in pneumonia and abscess development, probably through interference with the host apoptosis pathway [31–35,37,38].

Successful translocation of these T7SS substrates through the membrane requires the four membrane proteins EsaA, EssA-EssC [31,33]. While little is known of the molecular function of EsaA or EssA, available crystal structures of EssB and EssC suggest that EssB is a single-pass transmembrane protein that forms a conditional dimer with large extra- and intracellular domains [39–41]. EssC is a FtsK/SpoIIIE homolog with several ATPase domains, probably to provide energy for translocation [42–44]. The oligomerization state of the *S*. *aureus* T7SS is currently debated. The homologous secretion system from mycobacteria typically forms membrane-bound complexes >1 MDa [45–50] and similarly sized complexes have recently been purified from *S*. *aureus* [51]. This is in contrast to a recent report showing that T7SS membrane proteins form only homo-oligomers and might not interact with one another [52]; these proteins might not form large oligomers, at difference from other bacterial T7SS. Structural analyses of EssB and EssC in *S*. *aureus* nonetheless identified distinct domains that act as hubs for protein-protein interactions (the EssC FHA domain or the EssB pseudokinase fold), which suggests that multimerization is an important feature of this protein machinery [40,42,43,51].

Here we identified the T7SS as a complex associated with the FMM in *S*. *aureus*. We studied its functional dependence on FMM-harbored scaffold protein FloA and show that intact FMM are crucial for full T7SS activity *in vitro* and *in vivo*. We demonstrate that microdomain dissociation by genetic depletion of the FloA scaffold leads to decreased T7SS activity, probably due to less efficient T7SS membrane protein interaction. FMM dispersal by small anti-FMM molecule inhibitors and concomitant reduction of FloA scaffold activity similarly leads to reduced T7SS activity.

## Results

### T7SS proteins are part of the DRM

To identify new FMM-associated protein complex candidates, we quantified proteomes of detergent-sensitive (DSM) and -resistant (DRM) membrane fractions using a biochemical approach designed to purify eukaryotic raft-associated proteins [53,54]. This approach is based on the ability of rafts to resist disaggregation by treatment with non-ionic detergent (Triton X-100, Brij, CHAPS). Differences in lipid composition make rafts more compact than the remainder of the cell membrane and more resistant to detergent disaggregation [53,54]. After detergent treatment, large hydrophobic membrane fragments enriched in FMM can be concentrated in a DRM fraction using a phase separation approach. This allows comparison to a DSM fraction that is sensitive to detergent disaggregation, as it is comprised mainly of phospholipids and does not concentrate to the hydrophobic phase during phase separation [6]. As FloA expression is higher and its activity possibly more important, we used stationary cultures of *S*. *aureus* cells to isolate DRM and DSM fractions and quantify the proteins by label-free quantification (LFQ) mass spectrometry [55]. We plotted LFQ intensities of DRM fraction proteins against those of the DSM fraction, which led to separation into four protein populations (I-IV; S1A Fig). The first population (I) consisted of proteins detected exclusively in the DSM fraction; population II and III proteins were found in both DRM and DSM, with greater abundance in the DSM (II) or DRM fraction (III). Population IV was associated exclusively with the DRM fraction (S1A Fig). Most of the proteins we associated with DRM fractions were histidine kinases, lipoproteins, transporters, or components of protein complexes; we identified 12 of 13 known membrane-bound histidine kinases and more than 85% of all identified components of *S*. *aureus* membrane transporters (ABC transporters; ion, nutrient and metabolite transporters) in DRM-associated populations (III, IV) (S1B Fig). Proteins involved in division, like FtsA, FtsY or FtsI, were generally detected in DSM populations (S1B and S1D Fig). Nonetheless, sequence analysis of the DRM and DSM proteins categorized by this approach did not lead to the identification of any common features within each protein population (I-IV) (S1C Fig); all proteins identified for each population are listed in S1 Table. These findings are consistent with the hypothesis that FMM, which are very concentrated in the DRM fractions, mainly contain proteins that require protein-protein interaction or form part of protein complexes.

During our data analysis, we detected several proteins of the *S*. *aureus* T7SS, significantly enriched in the DRM fraction (Fig 1B); these included membrane proteins EsaA, EssA, EssB, which suggested that *S*. *aureus* T7SS is physically and functionally associated to FMM. We used purified DRM and DSM fractions to measure T7SS membrane proteins EsaA, EssA, EssB and EssC by immunodetection, using polyclonal antibodies to EsaA, EssB or EssC [33]. EssA was fused to the codon-optimized RFP variant MARS and detected with polyclonal anti-mCherry antibodies [56]. Western blot analyses confirmed EsaA, EssA and EssB enrichment in the DRM fraction (Fig 1C). Signals attributable to EssB and EsaA were enriched in the DRM fraction (~10- and 2-fold, respectively), and immunodetection of EssA-MARS showed most of the signal associated with this fraction. EssC was also detected in the DRM fraction, although it was not markedly enriched in this fraction compared to the DSM. These data are consistent with the LFQ mass spectrometry findings and correlate T7SS and DRM in *S*. *aureus*. Based on these results, we hypothesized that T7SS proteins are associated with the *S*. *aureus* FMM, probably in a transient manner, as we did not detect all T7SS membrane-associated proteins exclusively in the DRM fraction.

The FMM are specific membrane regions that probably act as oligomerization platforms. They spatially confine interacting protein partners and promote efficient interaction/oligomerization of multiprotein complexes [23,25,57]. The membrane-bound scaffold protein flotillin localizes preferentially to the FMM. Flotillin probably has an important role in tethering interacting proteins and facilitates oligomerization, similar to its function in eukaryotic lipid rafts [19,22,23]. To determine whether the T7SS-related membrane proteins in the DRM fractions are among the flotillin-tethered protein partners, we used a bacterial two-hybrid assay in a heterologous *E*. *coli* system, in which *S*. *aureus* flotillin (FloA) and T7SS membrane proteins were tagged with T25 or T18 fragments of an adenylate cyclase. After flotillin interaction with T7SS proteins, the enzyme is reconstituted, produces cAMP and triggers expression of a measurable cAMP-inducible *lacZ* reporter [58]. We detected strong interaction between FloA and EssB (>2000 Miller Units; a 700 Miller Unit threshold defines positive and negative interaction signals), with no consistent interactions between FloA and EsaA, EssA or EssC (Fig 1D and S2 Fig). Our protein-protein interaction analyses in a heterologous system thus suggest interaction between flotillin and the membrane-bound T7SS protein EssB from *S*. *aureus* T7SS.

### Flotillin interacts with EssB in *S*. *aureus* cells

**Fig 2:**
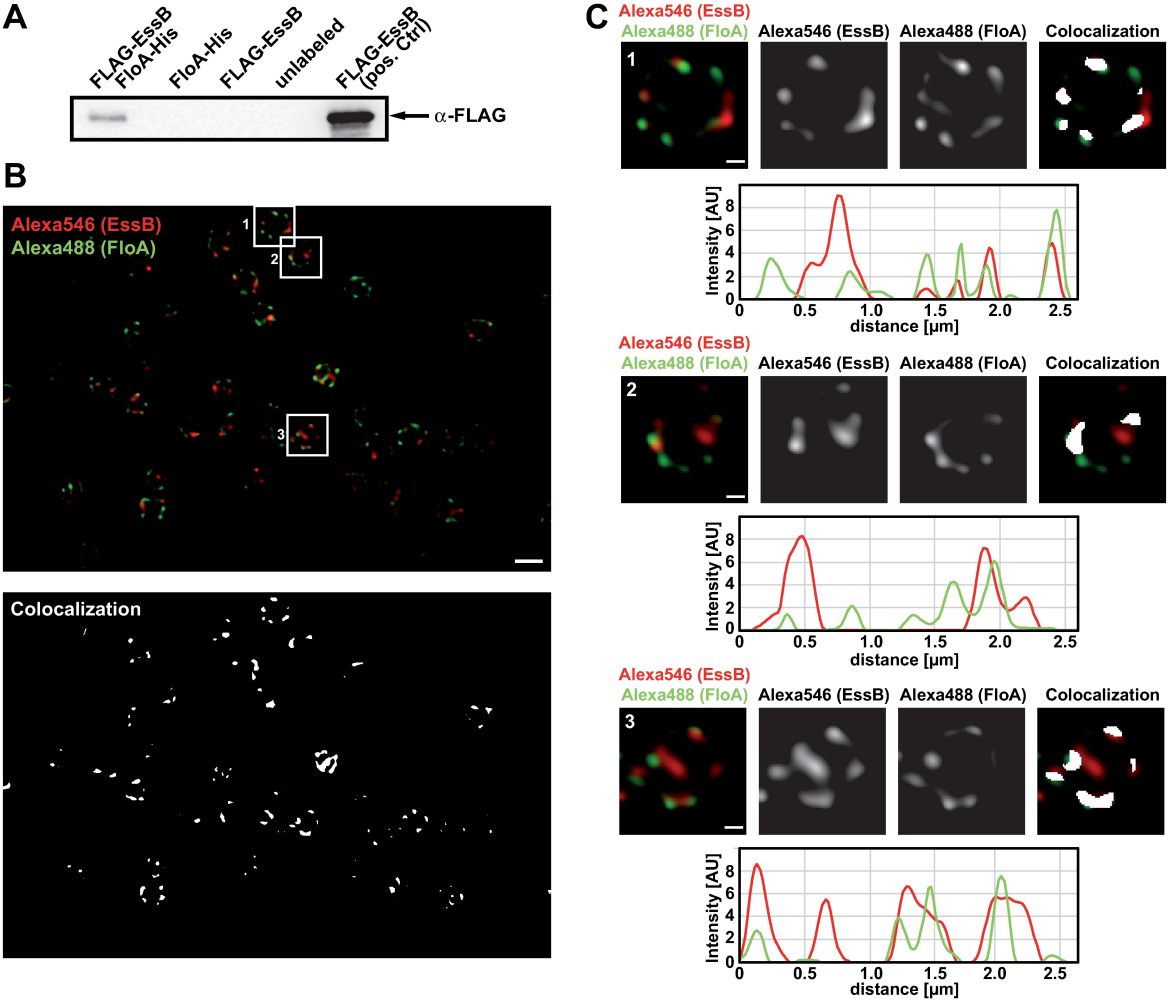
Flotillin interacts with the T7SS protein EssB. (A) Immunoblot analysis of pulldown assay to show the *in vivo* EssB and FloA interaction in *S*. *aureus*. Lane 1 shows FLAG-EssB FloA-His elution fraction from a Ni-NTA column. Negative controls (lanes 2, 3) are single-labeled strains and lane 4 is an unlabeled strain; lane 5 is the FLAG-EssB FloA-His double-labeled strain membrane fraction as positive control. (B) STED (stimulated emission depletion) microscopy images of a *ΔspA* strain double-immunolabeled with anti-FloA (Alexa488) and anti-EssB (Alexa546) antibodies. Top panel shows overlay of red and green fluorescent signals, false colored with red and green, respectively. Three individual cells are highlighted and further analyzed in panel C. Bottom panel shows colocalization analysis performed with the ImageJ JACoP plugin. Each pixel containing signal in both red and green fluorescent channel is now represented with a white pixel. Bar, 1 μm. (C) Three representative cells showing EssB and FloA colocalization. Top rows show overlay of false colored red and green fluorescent signals on the left and single red (EssB; Alexa546) and green (FloA, Alexa488) fluorescent channels in the center. Image on the right shows overlay of the white signal of colocalization analysis with the false-colored red and green-fluorescent channels. Bar, 0.2 μm. Each bottom panel shows pixel intensity analysis clockwise around the outline of the cell starting at the top.

We extended the FloA-EssB interaction analyses to *S*. *aureus* cells and performed pulldown experiments to identify interaction between FloA and EssB, using a FLAG-EssB, FloA-His double-labeled strain. FloA-His is a functional construct used in previous studies [59]. The FLAG-EssB construct was functional when expressed in a *ΔessB* genetic background, as immunodetection experiments of the EsxC substrate in culture supernatants showed that this complemented strain rescued the *ΔessB* mutant secretion defect (S3 Fig) [31,33,39]. A purified membrane fraction of this FLAG-EssB, FloA-His double-labeled strain was loaded on a column of Ni-NTA/His-tag resin selective for proteins that bind directly or indirectly to FloA-His. Eluted proteins were resolved by SDS-PAGE and FLAG-EssB was detected by immunoblotting using monoclonal anti-FLAG antibodies. A FLAG-EssB signal was detected in the eluted sample of the double-labeled strain, suggesting that EssB co-eluted with FloA (Fig 2A). In contrast, the elution fraction of FloA-His and FLAG-EssB single-labeled strains showed no signal, which implies that EssB retention on the column was FloA-dependent.

We used stimulated emission depletion (STED) microscopy to examine EssB and FloA signal colocalization in *S*. *aureus* cells. STED microscopy is a super-resolution technique previously used in *B*. *subtilis* to demonstrate partial colocalization of FMM-associated proteins with the scaffold protein FloA [24]. Pull-down experiments or BN-PAGE coupled to immunoblotting have shown that flotillin interacts directly or indirectly with these FMM-associated proteins, and that this interaction contributes to FMM-associated complex oligomerization [9,57]. Partial colocalization of FloA with FloA-interacting FMM protein cargo is likely because flotillin does not form part of these oligomeric complexes and is thus not strictly necessary for their activity. The role of flotillin could be transient, to facilitate oligomerization of multimeric complexes that then act in a flotillin-independent manner. This is consistent with the role of FMM as oligomerization platforms, to promote efficient interaction between protein partners. To study FloA and EssB colocalization, we immunodetected these proteins in *S*. *aureus* cells, using specific antibodies. Subcellular signal localization in double-labeled samples was examined using STED microscopy (Fig 2B, top panel). Traditional colocalization studies focus on 1:1 ratios of red- and green-labeled proteins, resulting in a merged yellow signal. Proteins abundant at different ratios still colocalize, but are falsely disregarded using this approach. We determined protein colocalization independent of the relative FloA and EssB abundance, and present the results in a white colocalization map (Fig 2B, bottom). The flotillin signal was distributed in 1–6 fluorescent foci per cell, whereas the EssB signal formed 1–4 membrane foci. Using this method, we determined that 27% of the total EssB signal showed an overlap with FloA fluorescent signal. Thus, 73% of the EssB did not overlap with FloA and 68% of FloA did not overlap with EssB (Fig 2C). This partial colocalization indicates biochemical interactions of EssB and FloA and suggests that this EssB and FloA interaction occurs in a transient manner, similar to previous flotillin-associated interactions.

### T7SS secretion is low in *S*. *aureus* cells lacking flotillin

**Fig 3:**
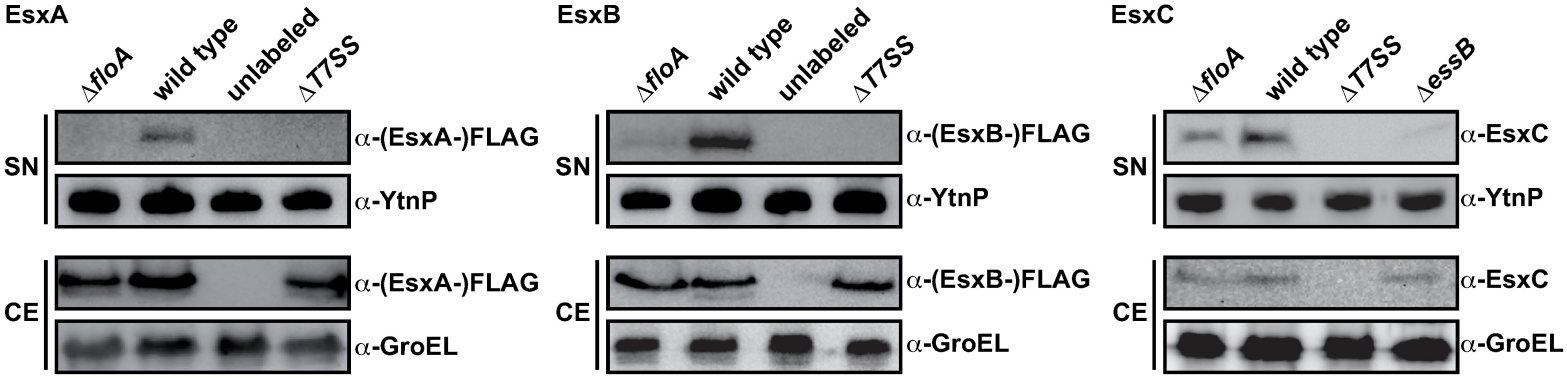
FloA mutant reduces secretion efficiency of T7SS substrates EsxA, EsxB and EsxC. Cells were grown to early stationary growth phase. Filtered supernatants (SN) were supplemented with recombinant YtnP as a control of equal loading, then precipitated and immunoblotted and proteins detected with anti-FLAG, -YtnP and -EsxC antibodies. Cell extracts (CE) were probed with anti-FLAG and -EsxC antibodies. Detection of GroEL served as loading control. For FLAG-labeled EsxA and EsxB, an unlabeled wild type strain served as negative control and ΔT7SS strain was used as a secretion-negative strain. For EsxC secretion, a ΔT7SS mutant was used as a negative control and a Δ*essB* mutant strain as a secretion negative strain.

Having determined that EssB is enriched in the DRM fraction and interacts with FloA, we analyzed FloA influence on T7SS activity; this is an important aspect of *S*. *aureus* virulence, since EssB is essential for secretion of T7SS effectors during infection [31,33,39]. We compared the levels of the T7SS substrates EsxA and EsxB and of EsxC in wild type or FloA-defective cells in culture supernatants. EsxA and EsxB were C-terminally labeled with a FLAG-tag and expressed under the control of a constitutive promoter. Culture supernatant proteins were concentrated by trichloroacetic acid (TCA) precipitation, and EsxA- and EsxB-FLAG were immunodetected with monoclonal anti-FLAG antibodies and EsxC with EsxC-specific polyclonal antibodies [33]. The *ΔfloA* mutation might alter other secretory protein complexes in *S*. *aureus*, like the Sec system defect in a *B*. *subtilis* flotillin-deficient strain [9]. To normalize total *ΔfloA* mutant-to-wild type secreted protein in immunoblot analyses, we normalized cell culture optical density and added a defined concentration of an unrelated protein to supernatants. We added purified denatured YtnP lactonase (25 μg/ml) from *B*. *subtilis* [60] to supernatants before TCA precipitation, and we used polyclonal anti-YtnP antibodies to trace YtnP concentration and thus ensure comparable concentration of the supernatant. In this system, EsxA-FLAG and EsxB-FLAG showed a marked decrease in supernatants from the Δ*floA* mutant compared to wild type (Fig 3); EsxC substrate was also decreased in Δ*floA* compared to wild type supernatants, which implied an important flotillin role in T7SS activity. Quantitative differences in T7SS substrates in the supernatant are probably due to reduced T7SS secretion efficiency rather than reduced abundance of its components, as immunodetection indicated similar EsaA, EssA, EssB and EssC protein levels in wild type and Δ*floA* mutant whole cell extracts (S4 Fig).

### Flotillin mediates T7SS intermolecular interactions

**Fig 4:**
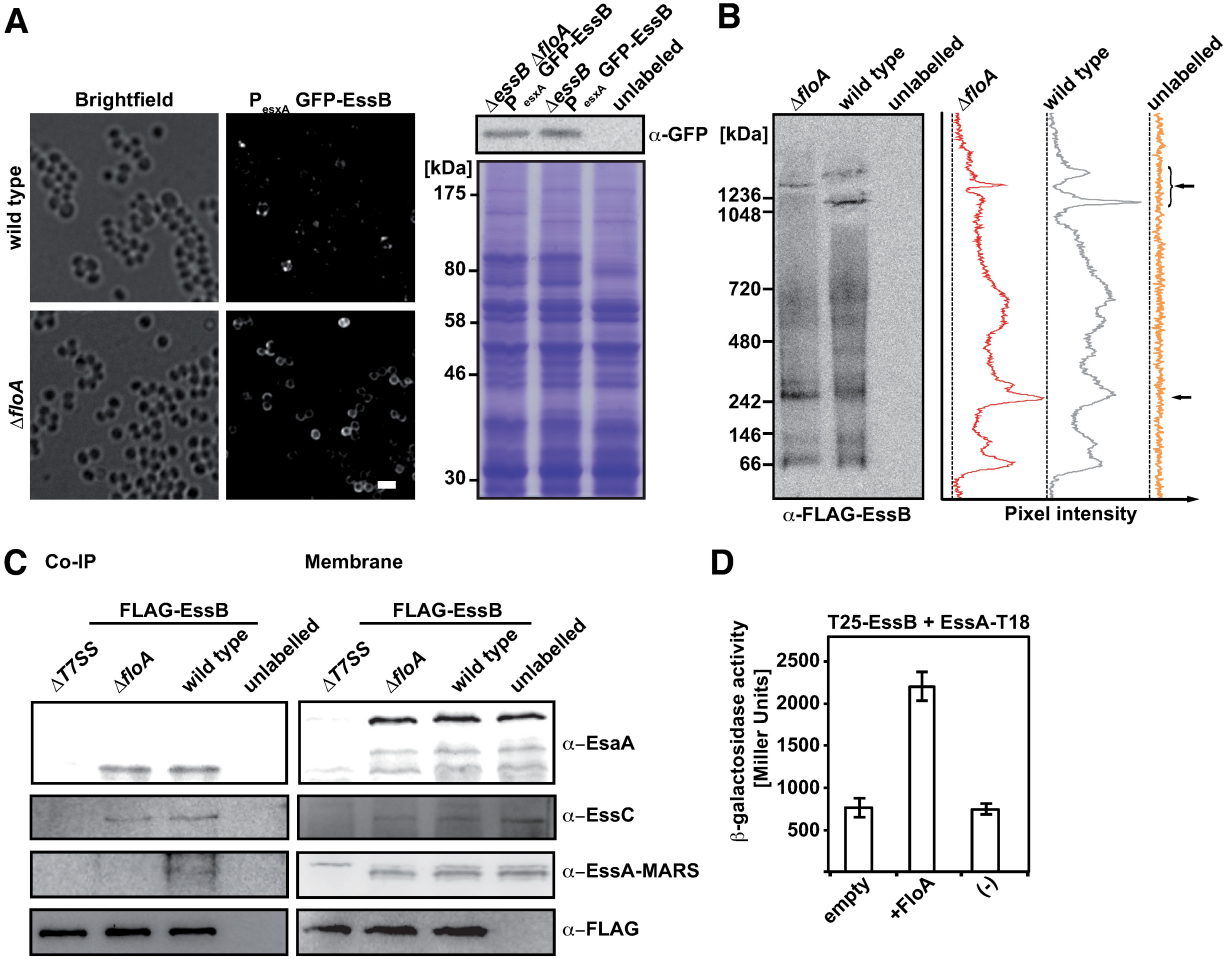
FloA is important for intermolecular interactions of T7SS membrane proteins. (A) Subcellular localization of GFP-EssB. Bright field and green fluorescence of a complemented GFP-EssB controlled by its own promoter (P_esxA_) in a wild type and a Δ*floA* background in stationary growth phase. Bar, 1.5 μm (right). Corresponding immunoblot analysis of P_esxA_ GFP-EssB strains using polyclonal anti-GFP antibodies. An unlabeled wild type strain served as a negative control (left). (B) BN-PAGE analysis of DSP-crosslinked membrane fractions of *S*. *aureus* expressing complemented FLAG-EssB on a wild type or a Δ*floA* background using a monoclonal anti-FLAG antibody (left). The right panel shows a pixel intensity analysis of this BN-PAGE. The top arrow indicates higher molecular weight oligomers of EssB and the bottom arrow, low molecular weight oligomers. (C) Pulldown analysis of FLAG-tagged EssB using FLAG-capture beads. The blots show the eluted fractions of wild type, Δ*floA* mutant and a ΔT7SS mutant expressing FLAG-EssB; an unlabeled wild type served as negative control. EsaA and EssC were detected using polyclonal antibodies, EssA-MARS was detected using a polyclonal antibody to the mCherry protein. Immunoblot of CoIP elution fractions (Co-immunoprecipitation) (left) and of the input membrane fractions (right). Besides full-length EsaA, several fragments were detected, but only one co-eluted with FLAG-tagged EssB in the pull-down experiment. (D) Bacterial three-hybrid assay to study EssB interaction with EssA, alone or with flotillin on a third plasmid (pSEVA). Quantification of T25-EssB and EssA-T18 interactions were assayed with β-galactosidase activity assay using empty plasmid (pSEVA641), plasmid bearing flotillin (pSEVA641*-floA*) or no pSEVA plasmid (-).

The most direct hypothesis as to how flotillin influences T7SS activity is that its scaffold activity promotes T7SS stability or assembly by tethering interacting proteins [3]. In the absence of FloA, EssB might oligomerize less efficiently and negatively affect correct T7SS organization. We thus used fluorescence microscopy to analyze EssB subcellular distribution in wild type and Δ*floA* mutant cells labeled with a GFP-EssB translational fusion. We constructed a GFP-EssB Δ*essB* complemented strain and confirmed its function as above (S3 Fig). Compared to the punctate GFP-EssB pattern on a wild type background, EssB foci were undetectable in Δ*floA* mutants, and the GFP-EssB signal was distributed uniformly over large portions of the membrane (Fig 4A, left) whereas protein abundance was unaffected (Fig 4A, right). This suggests that flotillin determines correct subcellular localization of EssB.

EssB oligomerizes *in vitro* and forms conditional dimers [39–41], although *in vivo* homo- or hetero-oligomerization has not been detected [52]. We thus tested whether FloA scaffold activity affects EssB oligomerization directly. FLAG-EssB-labeled *S*. *aureus* was cultured to stationary growth phase and membrane protein extracts (0.25% DDM, 4°C) (S5A Fig) were resolved by 3-12% gradient Blue-Native (BN)-PAGE, which allows separation of membrane protein complexes into their natural oligomeric states (30–10,000 kDa) [61–63]. FLAG-EssB was immunodetected using specific anti-FLAG antibodies. We observed a signal attributable to EssB at >250 kDa, which indicates that EssB forms stable oligomers *in vivo* (S5B Fig). Protein samples crosslinked using DSP (dithiobis(succinimidyl propionate)) prior to cell lysis showed EssB-containing oligomers >1 MDa (S5B Fig), which indicated the existence of large EssB-containing complexes in *S*. *aureus* cells. We applied this experimental approach using anti-FLAG epitope antibodies to compare FLAG-EssB-labeled wild type and Δ*floA* mutant membrane fractions (Fig 4B). Wild type cells usually showed two signals that corresponded to distinct EssB oligomeric states (>1 MDa), whereas Δ*floA* mutant showed one >1 MDa signal that did not coincide with either wild type band (Fig 4B). In addition to these differences, we detected an intensity increase in a low molecular weight signal (~250 kDa) in the Δ*floA* mutant compared to the wild type strain (Fig 4B, right, bottom arrow). These results suggest an important role for FloA in formation of EssB-containing high molecular weight complexes in *S*. *aureus*.

To ascertain whether these very large protein complexes are EssB homo-oligomers or are constituted by other T7SS proteins, we analyzed FLAG-EssB in a pulldown analysis using FLAG-capture beads. Beads captured FLAG-EssB as well as EssB-associated proteins, which were eluted, resolved in SDS-PAGE, and immunoblotted, which indicated EssA, EssC and an EsaA fragment (Fig 4C). The polyclonal EsaA antibody did not detect full-length EsaA in the pull-down assay, but showed fragmented EsaA, which is also detected in membrane fractions. Similar fragmentation is reported for the *B*. *subtilis* EsaA homolog [64]. The wild type and Δ*floA* eluted fractions showed EssC and the EsaA fragment in comparable amounts, but no EssA was detected in the Δ*floA* fraction (Fig 4C). These data suggest that flotillin mediates the EssB-EssA interaction.

To better understand the flotillin effect on the EssB-EssA interaction, we used a bacterial three-hybrid assay [25,57] to measure EssB-EssA oligomerization efficiency, alone or with FloA (Fig 4D). A bacterial two-hybrid assay in which EssB and EssA were tagged respectively with the T25 and T18 catalytic domains, was complemented with a modular vector [65] that expressed *floA*. Whereas there was no EssB-EssA interaction in the absence of FloA, the EssB-EssA interaction signal was increased when FloA was present (Fig 4D). FloA did not affect the EssB interaction with other T7SS membrane proteins (EsaA or EssC) (S6 Fig), which implied that FloA scaffold activity is specific to the EssB-EssA interaction. This finding indicates the importance of FloA expression in T7SS protein-protein interactions and supports the hypothesis that flotillin acts as a scaffold to promote EssB-EssA interaction.

**Fig 5:**
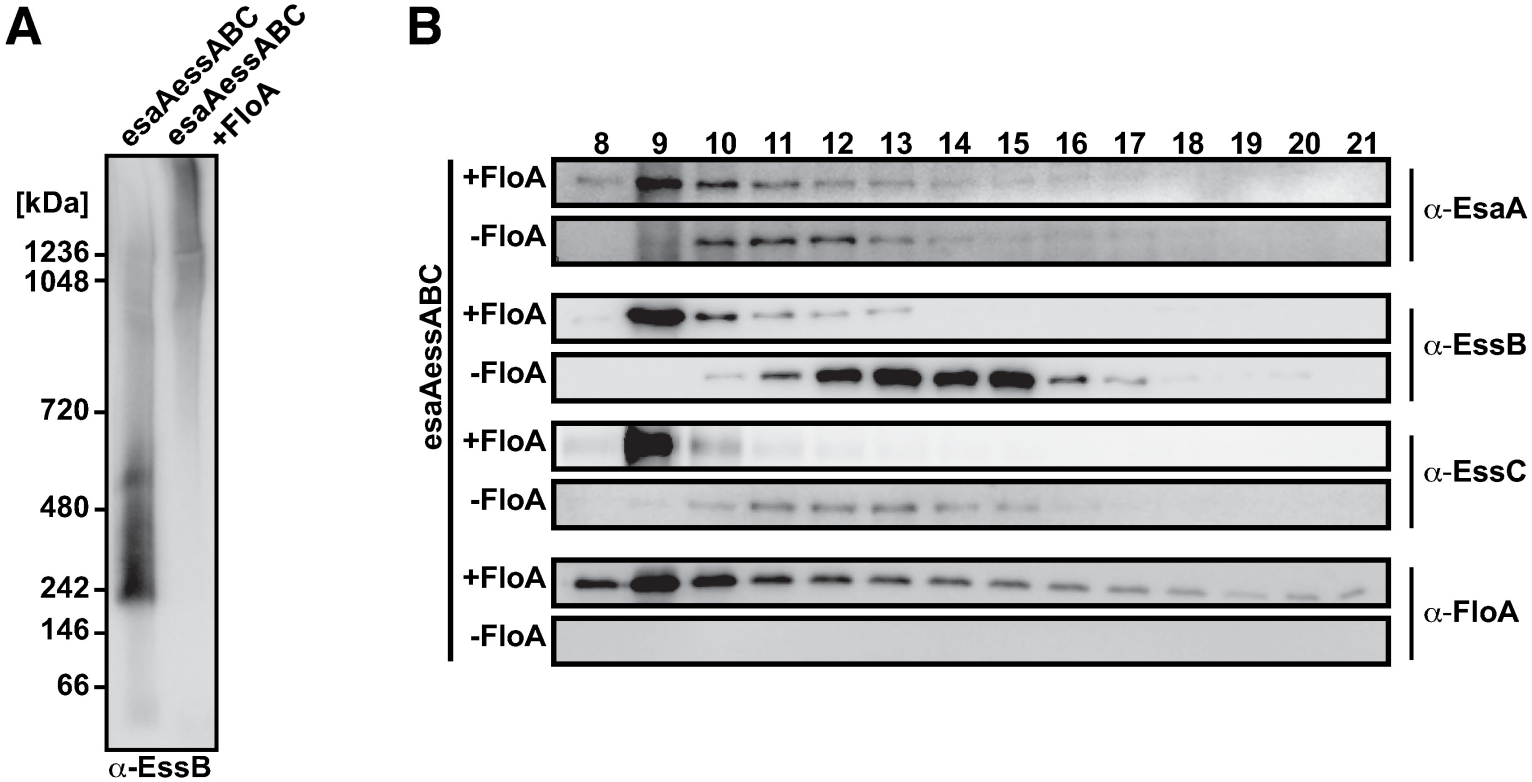
Effect of FloA on reconstituted T7SS in *E*. *coli*. (A) BN-PAGE and immunoblot analysis of solubilized *E*. *coli* membranes expressing structural T7SS proteins EsaA, EssA, EssB and EssC alone or with FloA. EssB was detected using polyclonal antibodies. (B) Size exclusion chromatography on a Superose 6 column with solubilized membrane fractions expressing structural T7SS proteins EsaA, EssA, EssB and EssC alone (- FloA) or with FloA (+FloA). The fractions corresponding to the elution volumes at 8–21 ml were separated in SDS-PAGE and detected by immunoblotting with polyclonal anti-FloA, -EsaA, -EssB or -EssC antibodies.

To determine whether this FloA activity is sufficient to promote T7SS protein oligomerization, we genetically engineered an orthogonal T7SS system in *E*. *coli*, in which the T7SS membrane proteins EsaA, EssA, EssB and EssC were isolated from their native complex oligomerization network, and thus free from interference by potential staphylococcal oligomerization inputs. EsaA, EssA, EssB and EssC proteins were expressed alone (-FloA) or in the presence of FloA (+FloA). Solubilized membrane fractions were purified, proteins extracted and their oligomerization states identified by BN-PAGE and immunoblotting using anti-EssB antibody. In the absence of FloA (-FloA), EssB signals were observed in the 200-400 kDa range (Fig 5A), similar to EssB oligomers detected in uncrosslinked staphylococcal membranes (see Fig 4B and S5B Fig). In the presence of FloA (+FloA), EssB oligomeric species shifted towards higher molecular weight complexes (Fig 5A), indicating that FloA affects EssB oligomerization and potentially other T7SS complex components in the orthogonal biosystem.

To ascertain whether FloA exclusively affects EssB oligomerization or influences oligomerization of additional T7SS proteins in this system, we purified *E*. *coli* membrane fractions, extracted proteins and identified their oligomerization states by size-exclusion chromatography. We used immunoblot to analyze 1 ml fractions of the column volumes in an 8–21 ml range to detect FloA, EsaA, EssB and EssC (Fig 5B). In -FloA fractions, EsaA, EssB and EssC signals were detected in later-eluting fractions of 10–16 ml, corresponding to a 1-0.4 MDa range. In +FloA samples, the signal was detected in early-eluting fractions and concentrated in the 9 ml fraction, which corresponds to high molecular weight complexes >1 MDa (Fig 5B). These results suggest that flotillin affects T7SS membrane protein oligomerization in this orthogonal system, and further highlights the importance of this scaffold protein for *S*. *aureus* T7SS.

### The T7SS secretion defect of Δ*floA* is relevant during an infection

**Fig 6:**
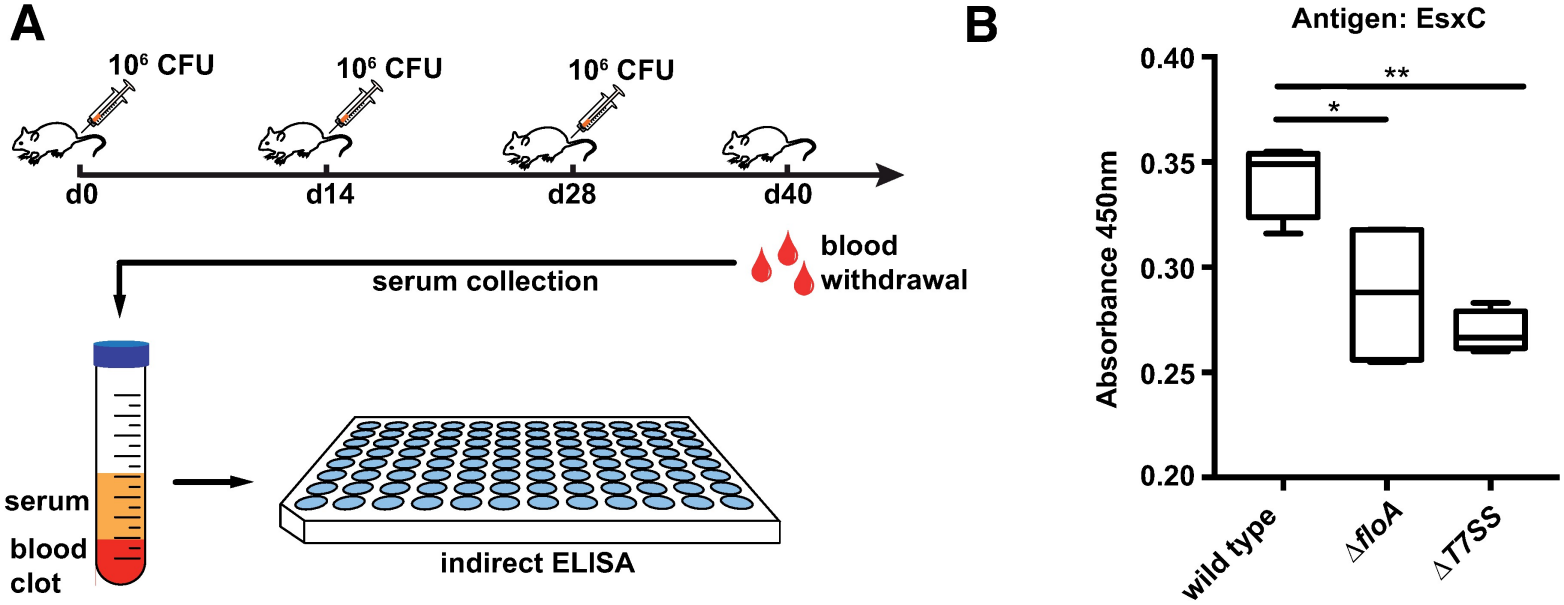
Lack of flotillin lowers EsxC-antibody titers in infected mice. (A) Scheme of workflow. Mice were challenged three times with sublethal doses of *S*. *aureus* (10^6^ CFU) on day 0, 14 and 28. After 40 days, blood samples were collected, serum isolated and used for indirect ELISA. (B) BALB/c mice were challenged as in (A) and IgM titers against EsxC were determined by indirect ELISA. Absorbance corresponds to 1:50 diluted sera. Statistical analysis was carried out using one-way ANOVA (*P<0.05; **P<0.01).

Targeting of flotillin scaffold activity could be an appropriate strategy for fighting bacterial infection by perturbing oligomerization of multiple virulence-related protein complexes such as T7SS. In mice, the T7SS Esx substrates participate in formation of persistent abscesses, probably due to the virulence of EsxA, EsxB, EsxC and EsxD secreted proteins [31,32,37,38,66]. In addition, EsxC is an immunogenic substrate, and kidney abscesses are associated with the generation of anti-EsxC antibodies [32]. In a murine infection model similar to that of Burts et al. [32], we evaluated the link between FloA inactivation and reduced *S*. *aureus* virulence mediated by low T7SS activity by measuring EsxC immunoreactivity. Cohorts of 3-week-old BALB/c mice received intravenous injections of sublethal doses of staphylococcal wild type, Δ*floA* mutant and Δ*T7SS* mutant strains. The humoral immune response was boosted by *Staphylococcus* challenge on days 14 and 28 [67]. After 40 days, mice were sacrificed and serum collected to determine anti-EsxC immunoglobulin titers by indirect ELISA (Fig 6A). We observed significantly lower IgM antibody titers against EsxC in a Δ*floA* mutant compared to wild type, consistent with *in vitro* experiments lowering EsxC secretion in the Δ*floA* mutant (Fig 6B). As control, we detected antibodies to the unrelated staphylococcal cell wall protein IsaA [68], which showed that all mice established an *S*. *aureus* infection (S7 Fig). These data indicate the importance of FMM integrity in T7SS-mediated virulence phenotypes in an *in vivo* infection.

### Small anti-FMM molecules reduce T7SS activity

To target flotillin activity exogenously and develop alternative strategies against staphylococcal infections, we tested several small molecules known to interfere with FMM organization in *S*. *aureus* [59]. The small molecule zaragozic acid (ZA) is a potential inhibitor of flotillin activity [12], as it blocks *S*. *aureus* squalene synthase [69], which is needed to produce the polyisoprenoid lipids that stabilize flotillin in the FMM. When bacteria are exposed to micromolar ZA concentrations, flotillin organizes in a smaller number of membrane foci, concomitant with a reduction in its chaperone activity [12]. Similar to ZA, the cholesterol-lowering drug simvastatin used to treat patients with hypercholesterolemia, inhibits the same polyisoprenoid lipid production pathways in *S*. *aureus*. Simvastatin is a competitive inhibitor of HMG-CoA reductase, an enzyme upstream of squalene synthase in the constituent lipid biosynthesis pathway [70]. We included the small molecule 5-DSA (5-doxyl-stearic acid), a lipid probe that accumulates in biological membranes and is used to monitor membrane fluidity in lipid raft organization studies in eukaryotic cells [71,72]. In addition, 5-DSA is reported to displace certain membrane lipids and alter the function of various membrane-associated proteins [73,74].

**Fig 7:**
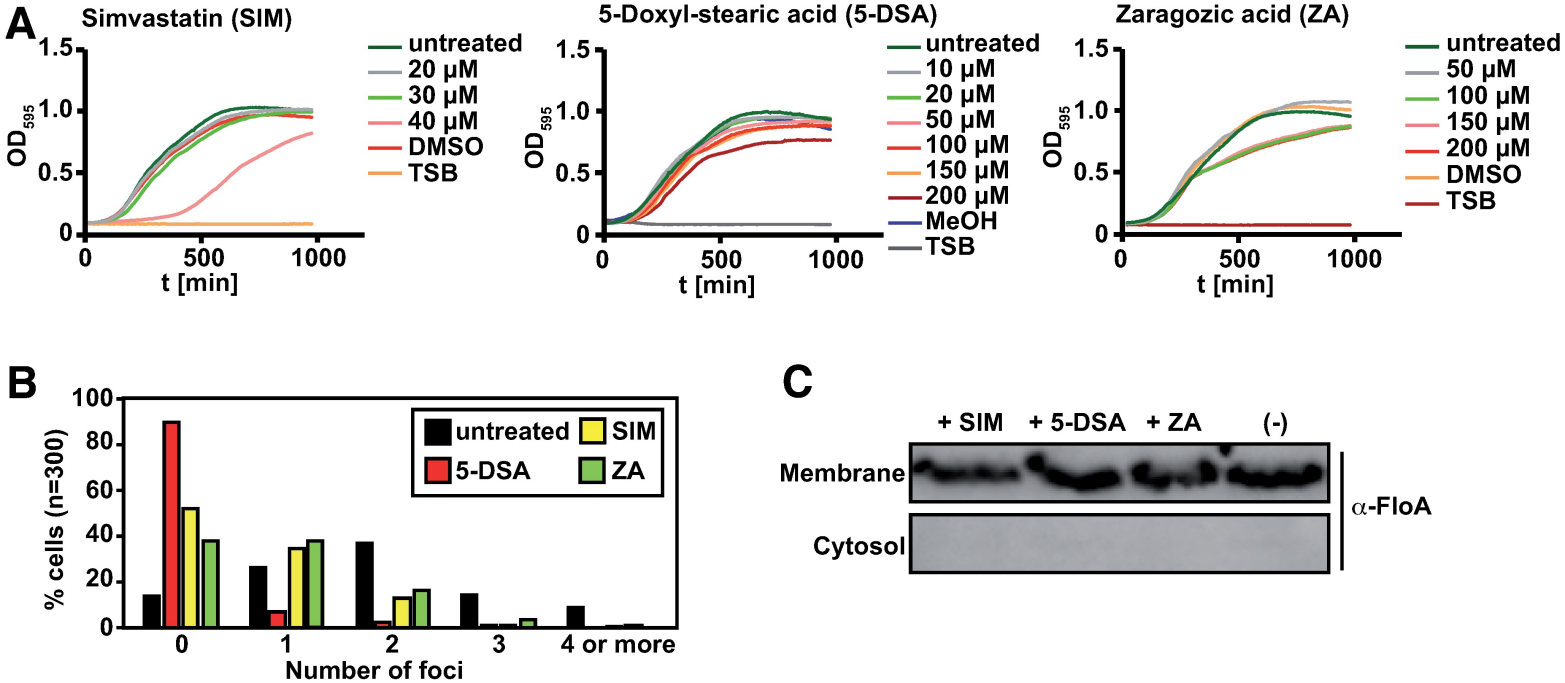
Anti-FMM molecules affects membrane organization of FloA foci. (A) Effect on growth of different concentrations of anti-FMM molecules simvastatin (SIM), zaragozic acid (ZA) or 5-doxyl-stearic acid (5-DSA) in TSB medium. (B) Fluorescent foci in FloA-MARS-labeled cells after treatment with 20 μM SIM, 150 μM 5-DSA or 50 μM ZA compared to untreated cells. (C) Immunoblot analysis of membrane and cytosol fractions of wild type cells treated with 20 μM SIM, 150 μM 5-DSA or 50 μM ZA. Untreated cells served as control. Flotillin was detected using polyclonal anti-FloA antibody.

Wild type *S*. *aureus* strains were grown in liquid TSB medium with different concentrations of simvastatin, 5-DSA and ZA to define the highest concentrations that did not affect *S*. *aureus* growth (20 μM simvastatin, 150 μM 5-DSA, 50 μM ZA; Fig 7A). We used fluorescence microscopy to monitor anti-FMM activity by evaluating FloA subcellular distribution. We quantified the number of fluorescent foci of a FloA-MARS strain grown with simvastatin, 5-DSA or ZA. Most untreated cells showed one, two or three fluorescent foci; occasional cells had no fluorescent foci or had four or more foci. Treatment with 20 μM simvastatin or 50 μM ZA reduced the number of fluorescent FloA foci per cell, and 150 μM 5-DSA treatment showed a drastic reduction in the number of foci per cell (Fig 7B, S8 Fig). The altered subcellular localization of FloA-MARS in the presence of anti-FMM molecules was not a result of decreased flotillin in the cell membrane or its displacement to the cytosol, as shown by immunoblot analysis of fractionated cells after anti-FMM treatment (Fig 7C). We thus suggest that these molecules severely alter FMM organization and probably affect associated processes.

**Fig 8:**
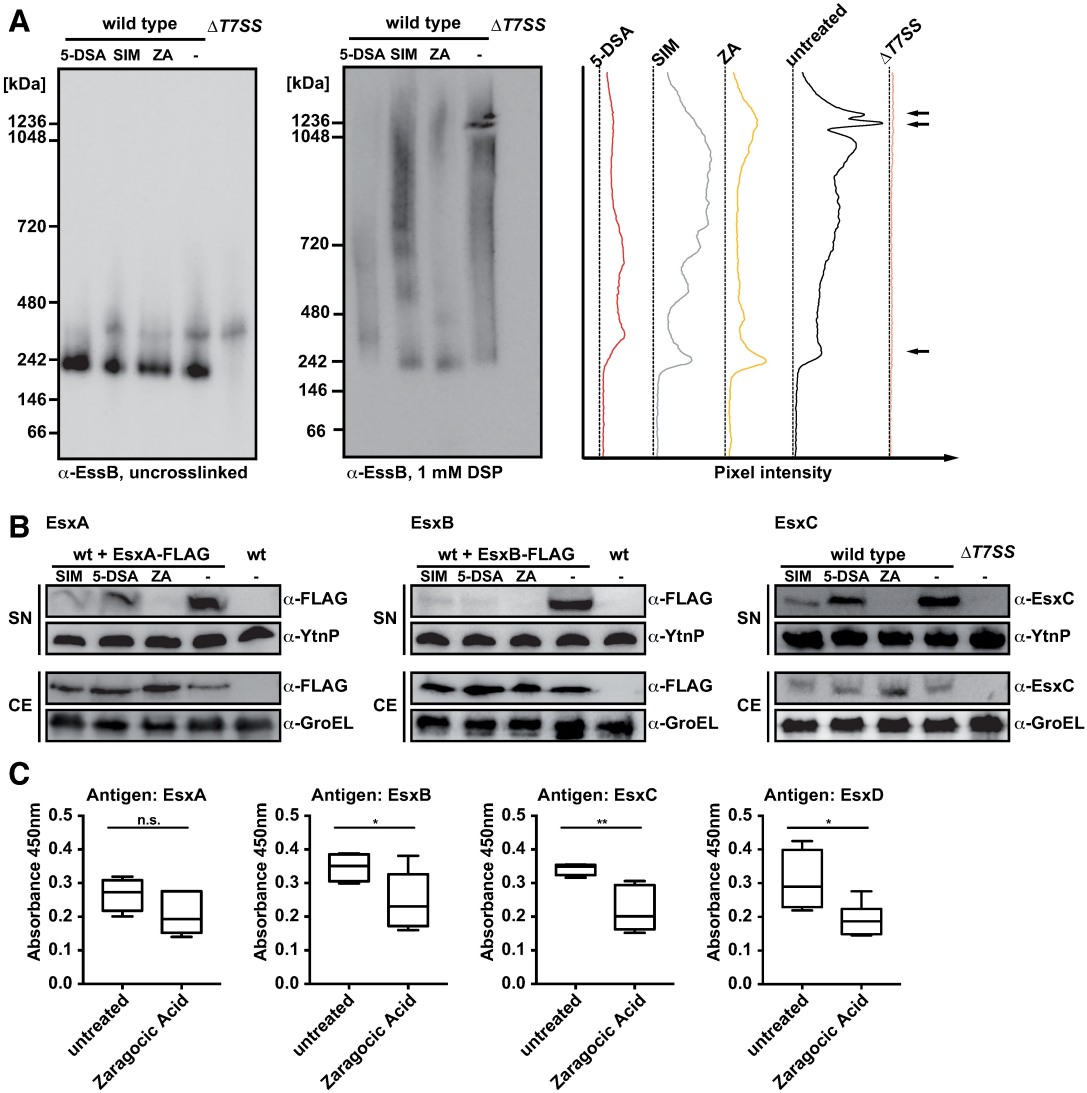
Anti-FMM molecules interfere with T7SS complex formation and can inhibit secretion of T7SS substrates *in vitro* and *in vivo*. (A) Effect of anti-FMM molecules on T7SS complex formation. BN-PAGE analysis of untreated vs. DSP-crosslinked membrane fractions of *S*. *aureus* cells grown to stationary phase in the presence of 20 μM SIM, 150 μM 5-DSA or 50 μM ZA. Pixel intensity analysis of DSP-crosslinked samples are shown (right). (B) Effect of anti-FMM molecules on secretion of T7SS substrates. Cells were grown to the end of exponential growth phase in the presence of 20 μM SIM, 150 μM 5-DSA or 50 μM ZA. Precipitated supernatant (SN) and corresponding cell extracts (CE) were separated in SDS-PAGE and tested in immunoblot with anti-FLAG or -EsxC antibodies. Recombinant YtnP was added to supernatants as loading control and probed with anti-YtnP antibodies; for cell extracts anti-GroEL was used as a loading control. (C) Indirect ELISA to study the ZA effect on antibody response to EsxA-D. Zaragozic acid was administered to BALB/c mice by intraperitoneal injection, followed by challenge with a sublethal dose of staphylococci. The procedure was repeated on days 14 and 28 to boost the antibody response. Graphs show IgM antibody titers to EsxA-D in untreated *vs*. treated mice. Statistical analysis was performed using an unpaired t-test (*P<0.05; **P<0.01).

To determine whether 5-DSA, simvastatin or ZA also affected T7SS assembly and organization, we repeated BN-PAGE analysis as above. Without crosslinking, the FloA-interacting protein EssB only formed oligomers at ~250 kDa; high molecular weight protein complexes were not detected (Fig 8A, left). DSP treatment before cell lysis allowed stabilization of high molecular weight complexes, and 5-DSA, simvastatin, and ZA treatment led to a decrease in the high molecular weight signal in wild type cells (Fig 8A, center). This was consistent with the increase in low molecular weight species detected in 5-DSA-, simvastatin-, or ZA-treated samples compared to wild type (Fig 8A, right, bottom arrow). BN-PAGE analysis thus suggested that 5-DSA, simvastatin, or ZA treatments interfere with correct T7SS assembly or organization, probably by inhibiting FloA scaffolding activity.

We tested whether 5-DSA, simvastatin or ZA treatments also inhibit T7SS secretion *in vitro*. To define this, we monitored secretion of the T7SS substrates EsxA, EsxB and EsxC in stationary phase cultures. Concentrated proteins from supernatants of untreated and simvastatin-, 5-DSA- and ZA-treated cultures were resolved by SDS-PAGE and analyzed in immunoblot. Simvastatin-, 5-DSA- and ZA-treated cultures showed a notable reduction in secreted EsxA, EsxB and EsxC, particularly marked in ZA-treated cells, for which no signals were detected (Fig 8B). To determine the localization of the T7SS substrates, we analyzed cell extracts by immunoblot, which showed EsxA, EsxB and EsxC signals in the cytoplasmic fraction of treated cultures. Simvastatin, 5-DSA and ZA in *S*. *aureus* cultures thus compromised T7SS activity and reduced T7SS substrate secretion (Fig 8B). While treatment of *S*. *aureus* cultures probably affects T7SS organization and assembly, simvastatin, 5-DSA or ZA addition did not alter EsaA, EssB or EssC concentrations in cell extracts (S9 Fig).

The ZA inhibitory effect on T7SS activity via FMM perturbation led us to test ZA-mediated T7SS inhibition *in vivo* in the murine infection model. We administered 20 mg/kg ZA to a cohort of BALB/c mice via intraperitoneal injection, followed by challenge with sublethal doses of *S*. *aureus*. This procedure was repeated twice (days 14 and 28); after 40 days mice were sacrificed and serum was collected for ELISA determination of immunoglobulin titers to EsxA, EsxB, EsxC and EsxD substrates. IgM antibody titers against all T7SS Esx substrates decreased for all T7SS Esx substrates, which was statistically significant for EsxB, EsxC and EsxD compared to levels in infected untreated mice (Fig 8C). These results indicate that ZA inhibits secretion of T7SS-related substrates *in vivo*, probably by reducing secretion efficiency. This observation makes ZA an attractive molecule for development as an alternative antimicrobial therapy in *S*. *aureus* infections.

## Discussion

The role of scaffold proteins in prokaryotic cells is being studied for a number of bacterial species. These proteins are thought to have a central role in regulating assembly of protein-protein interactions; their activity is thus important for correct function of many biological reactions [2,3,75]. We show that the scaffold protein flotillin (FloA) promotes more efficient interaction in a multimeric complex involved in staphylococcal virulence. Here we used FloA-mediated T7SS secretion as a case study to evaluate FloA scaffold activity, although FloA might also contribute to oligomerization of other DRM fraction multimeric complexes with a role in *S*. *aureus* virulence.

Lack of FloA causes a reduction in T7SS substrate secretion *in vitro* and *in vivo*, which implies an important FloA function in T7SS oligomerization. Additional possibilities for the role of flotillin in T7SS oligomerization should also be considered, for instance, that lack of flotillin causes changes in membrane stiffness [9], which might affect T7SS oligomerization indirectly. We nonetheless found that FloA interacts with the T7SS membrane component EssB, thus facilitating its interaction with EssA and probably with other T7SS protein components; this highlights the importance of FloA for T7SS oligomerization, although T7SS organization remains unclear. Whereas FloA scaffold activity assists oligomerization of complexes such as T7SS, FloA is not a T7SS structural protein; it probably contributes to more efficient complex assembly, although FloA activity is not absolutely essential for the activity of these complexes [76]. In fact, some T7SS oligomerization and secretion of T7SS effectors are detectable in the absence of FloA. Bacterial flotillin might thus have a transient role, acting as a scaffold for oligomerization of membrane-associated protein complexes, whose subsequent activity is flotillin-independent. Flotillin might contribute to organizing FMM membrane microdomains, which are specialized in confining specific protein complexes, and to promoting their efficient oligomerization; these complexes will be excluded from the FMM once oligomerized.

Bacterial FMM appear to facilitate efficient oligomerization of FMM-associated protein complexes (FMM act as “oligomerization factories”) [12], and flotillin scaffold activity has a key role in facilitating interaction of FMM-associated membrane protein partners. Biochemical approaches such as pull-down experiments or BN-PAGE coupled with immunodetection demonstrated flotillin interaction with FMM-associated proteins, as well as its importance for correct oligomerization of protein partners in various bacterial species [9,14,25,55,57,77].

FMM are dynamic structures and move across the bacterial membrane in milliseconds. STED fluorescence microscopy was used to show partial flotillin colocalization (milliseconds) with various FMM protein partners such as FtsH and SecA [24], previously identified in pull-down studies as flotillin interactors [9,13]. In the STED study, partial FloA colocalization with FtsH and SecA contrasted with full flotillin colocalization with NfeD, which implies that partial colocalization was not due to experimental factors [24]. The structural FMM proteins FloA and NfeD form part of the same operon, are expressed at similar levels, and are thus present in an equal ratio. When one is green- and one is red-labeled, colocalization is detected as a merged yellow signal. Most FMM cargo proteins are not expressed at the same ratio as FloA, however, and this approach can lead to false negative results due to differences in protein levels/signal intensities. To overcome these drawbacks, we studied FloA colocalization with FMM cargo proteins using a method that focuses on the signal, independent of its intensity [78]. Colocalization of proteins in unequal ratios can be detected using high-resolution microscopy images. Full colocalization of cargo proteins with flotilin would not be anticipated, as flotillin is not a structural component of these complexes.

Protein interaction with FloA in pull-down experiments, and partial colocalization using STED microscopy support the hypothesis that FMM act as oligomerization platforms, in which flotillin and probably other structural proteins like NfeD assist FMM organization. The NfeD/FloA interaction is stable and would help maintain correct FMM architecture. In contrast, flotillin interaction with the FMM protein cargo is probably transient, as this interaction facilitates efficient oligomerization of protein partners. Protein complexes will thus dissociate from flotillin once oligomerized and will abandon FMM.

**Fig 9:**
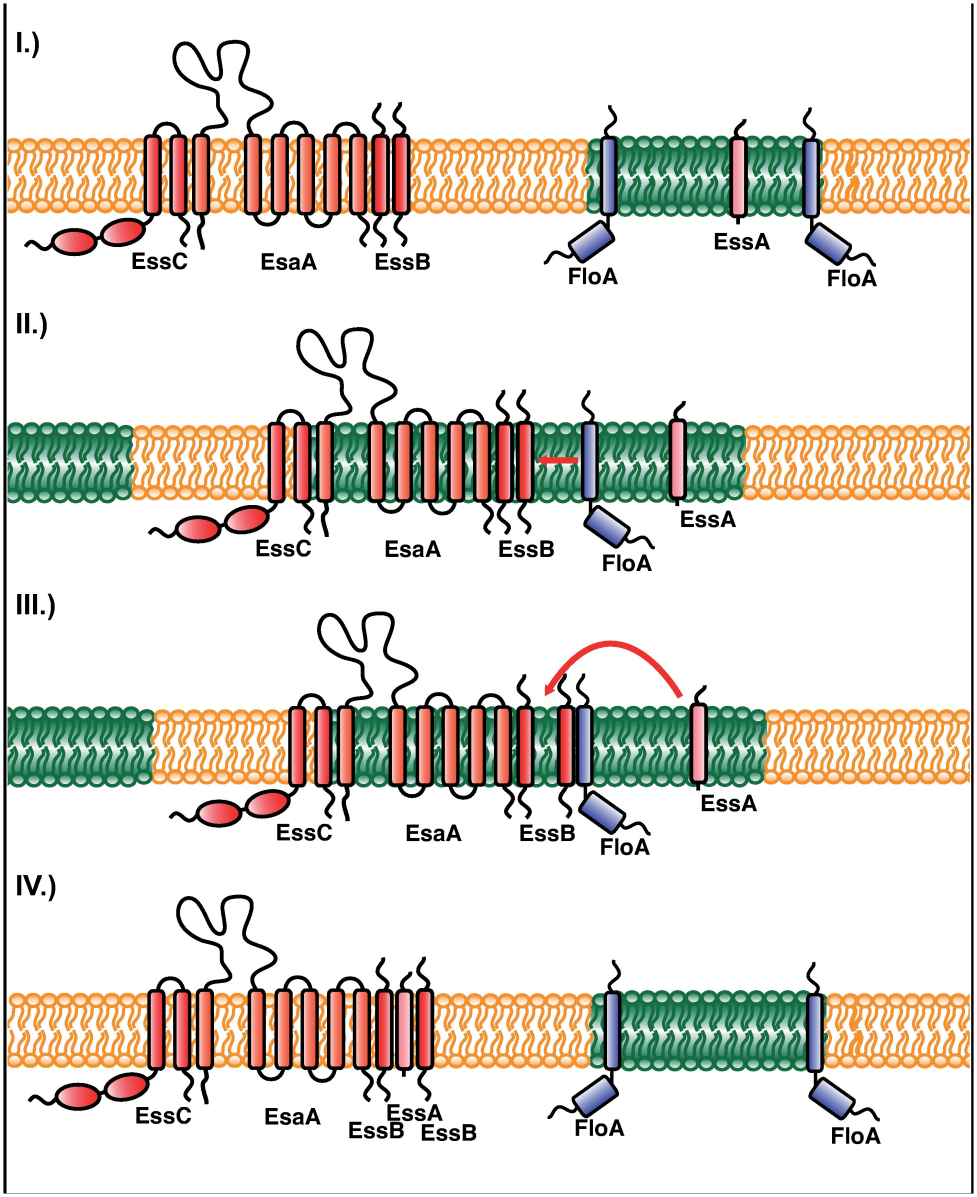
Model of FloA/FMM-mediated T7SS assembly. I.) A pre-existing, probably non-functional T7SS complex consisting of EssC, EsaA and an EssB oligomer (here represented by two EssB molecules) resides in the non-FMM membrane (yellow). FMM (green) contain EssA and FloA. II.) The EssC-EsaA-EssB pre-complex is transiently recruited to the FMM. This allows FloA to interact with EssB. III.) FloA scaffold activity promotes EssB-EssA interaction. This could help to incorporate EssA into the EssC-EsaA-EssB pre-complex. IV.) The fully assembled T7SS complex is released from FMM and is now functional.

Using a number of biochemical approaches, we detected FloA interaction with EssB, and showed partial FloA/EssB colocalization using STED microscopy. We thus propose a model to explore the role of FMM and FloA in T7SS organization, as illustrated in Fig 9. The FloA interaction with EssB facilitates EssB-EssA binding; as EssB and EssA are significantly enriched in the DRM, this binding probably takes place in FMM. In contrast, FloA does not influence EssB interaction with other T7SS proteins (EsaA, EssC), since EsaA and EssC are detected in pull-down experiments in a Δ*floA* mutant background. We hypothesize the formation of an EssC-EsaA-EssB pre-complex, and EssA incorporation via FloA scaffold activity to generate T7SS in a flotillin-dependent manner.

Although our data indicate a large T7SS hetero-oligomer, the nature of T7SS assembly is currently debated. Some reports argue that T7SS assembly in *S*. *aureus* resembles that in mycobacteria [46,48,50,51], but there are also experimental evidences that interactions between T7SS protein components in *S*. *aureus* cells are exclusively homomeric [52]. Based on our results, we consider that both hypotheses are probably correct and are not necessarily mutually exclusive. T7SS assembly is influenced by the activity of proteins that catalyze T7SS oligomerization, such as flotillin. It is possible that T7SS assembly is transient during the bacterium lifespan, and is regulated by the activity of scaffold proteins expressed at a specific time during growth or in response to a specific signal. These signals are likely to be produced during an infection in which T7SS activity is necessary, which impedes reproduction of these conditions in the laboratory. In addition, laboratory conditions might affect results for T7SS assembly; the choice of detergent and its concentration for membrane proteins solubilization is critical for an appropriate balance between membrane disaggregation and extraction of membrane proteins in their natural oligomeric states, and subtle variations in detergent use can affect results notably [79].

During an infection in a murine model, the *S*. *aureus* T7SS is critical for abscess development [31,32]. Using a variety of virulence factors, *S*. *aureus* is able to evade immune mechanisms and eventually disseminate into peripheral organs to establish the formation of a purulent abscess [80]. Secretion of the T7SS substrates EsxA and EsxB is important in kidney and liver abscess formation [31]. EsxC is crucial for maturation of these abscesses over a prolonged period, leading to establishment of persistent infection during which EsxC continues to be produced, since infected mice generate antibody responses against EsxC [32]. Although the actual host cell targets are unknown, one can speculate that Esx substrates interfere with several immune mechanisms (apoptosis, cytokine responses), similar to T7SS substrates of *Mycobacterium tuberculosis*, to generate a severe persistent infection [38,81,82].

Targeting the activity of bacterial scaffold proteins could thus be an innovative antimicrobial strategy that could reduce the virulence potential of *S*. *aureus* during a persistent infection. Here we show the potential antimicrobial effect of simvastatin, 5-DSA and ZA by their ability to perturb FMM organization, reduce T7SS function, and thus inhibit T7SS-mediated secretion of the EsxA, EsxB and EsxC substrates. Treated cells showed reduced extracellular EsxA, EsxB and EsxC levels, both *in vitro* and in an *in vivo* mouse infection model. Anti-FMM compounds could be a promising anti-microbial strategy to interfere simultaneously with molecular pathways that contribute to the virulence potential of *S*. *aureus*, toward eliminating hard-to-treat *S*. *aureus* infections. Such infections are considered endemic in hospitals, and their ~20% mortality rate in invasive MRSA infections makes them a leading cause of death by a single infectious agent [30].

## Materials and methods

### Strains, media and culture conditions

Strains used in this study were *Staphylococcus aureus* RN4220 [29] and *S*. *aureus* USA300_TCH1516 [83]. *Escherichia coli* DH5α and XL-1 Blue were used for cloning, and BL-21 Gold for recombinant protein expression. *E*. *coli* strains and *S*. *aureus* were propagated on LB and TSB media, respectively. Selective LB plates for *E*. *coli* were prepared using ampicillin (100 μg/ml), chloramphenicol (25 μg/ml), kanamycin (50 μg/ml) and gentamycin (2 μg/ml). *S*. *aureus* was selected on TSB plates containing spectinomycin (600 μg/ml) or erythromycin (2 μg/ml for RN4220 isolates and 100 μg/ml for the USA300_TCH1516 isolate, which bears an erythromycin resistance gene). For blue/white screenings, X-Gal (5-bromo-4-chloro-3-indolyl-β-D-galactopyranoside) was added to the plates (final concentration 50 μg/ml). If proteins were controlled by inducible promoters and induction was required, IPTG (isopropyl-β-thiogalactopyranoside) or xylose was added at 1 mM or 1% (v/v), respectively. The anti-FMM molecules 5-doxyl-stearic acid and simvastatin were purchased from Sigma-Aldrich and zaragozic acid from Santa Cruz. Simvastatin and zaragozic acid were dissolved in DMSO to a 10 mg/ml stock solution and 5-doxyl-stearic acid was dissolved in methanol to 75 mM stock solution. Compounds were added to cultures at specified concentrations.

### Strain construction

All strains and plasmids were generated according to standard molecular biology techniques [84]. A complete list of strains, plasmids and primers used can be found in S2 and S3 Tables. For protein tagging (with GFPmut2, RFPMars or FLAG-Tag), the CDS of the protein of interest was fused to the respective tag via LFH (long flanking homology)-PCR [85]; the PCR fragment was subsequently cloned into target plasmids using suitable restriction enzymes. If the native promoter was required, a 500 bp fragment upstream of the corresponding operon was fused upstream to the CDS by LFH-PCR. For gene deletion and ectopic gene expression in *S*. *aureus*, we used the pMAD plasmid and its derivatives pAmy and pLac [86,87]. pMAD plasmids were constructed and maintained in *E*. *coli* and then transformed in *S*. *aureus* RN4220 via electroporation. After successful integration of the entire plasmid into the genome (first recombination), constructs were shuttled to USA300 via ϕ11-phage transduction and selected for blue color and erythromycin resistance. To eliminate the pMAD plasmid backbone (containing the erythromycin resistance gene and β-galactosidase), strains were grown at 42°C, plated on TSB X-Gal and white colonies were screened for loss of plasmid and presence of target construct by colony PCR (second recombination).

The flotillin deletion mutant (Δ*floA*::*spc*) was constructed previously [59] and transferred into the USA300_TCH1516 isolate by ϕ11-phage transduction. The MARS-fluorescent protein fusion to FloA was generated by replacing the CDS of *floA-yfp* with *floA-mars* in a pCel plasmid created previously [59].

For analysis of protein-protein interaction via bacterial two- and three-hybrid systems [25,57,58], the coding sequences of the corresponding gene without the stop codon were cloned in-frame into each of the bacterial two-hybrid vectors (pKT25, pKNT25, pUT18, pUT18C). For bacterial three-hybrid analysis, the CDS of flotillin controlled by an IPTG-inducible P_lac_ promoter was introduced into plasmid pSEVA631 of the SEVA system [59,65].

For recombinant expression of EsxA, EsxB, EsxC and EsxD, we used the plasmid pET20b(+), which allows tagging with a hexahistidine tag for purification. The plasmid also contains a periplasm-targeting sequence that was removed by cloning the inserts in-frame with NdeI and XhoI. For recombinant expression of FloA and EssB, the CDS were cloned into pASK-IBA3 using reverse PCR to linearize the vector bearing a C-terminal StrepII-tag. The construct to express recombinant T7SS structural genes EsaA, EssA, EssB and EssC was from GenScript. The genes are codon-optimized for *E*. *coli* and contain a ribosome-binding site between each gene to ensure translation of all components. The *esaAessABC*-CDS was cloned into the pBAD-HisB vector by in-fusion PCR, excluding the hexahistidine-tag encoded in the vector.

### EssB antibody production

For antibody production, pASK-IBA3C*-essB* was transformed in *E*. *coli* TOP10 (Life Technologies) and a single colony used to inoculate an overnight pre-culture. The pre-culture was diluted 1:100 and grown at 37°C to an optical density of OD_600_ = 0.4–0.6. EssB was expressed by adding 2.5 mM anhydrotetracycline (24 h, 18°C). Cells were harvested by centrifugation (6000 xg, 20 min, 4°C) and resuspended in 300 mM NaCl, 50 mM Tris pH 8.0. Cells were lysed by two rounds of French press-mediated lysis (10,000 psi) and the membrane fraction of cleared lysate was collected by ultracentrifugation (185,000 xg, 1 h, 4°C). Membrane proteins were then extracted in 300 mM NaCl, 50 mM Tris pH 8.0, 1% DDM (1 h, 4°C). Insoluble proteins were removed by ultracentrifugation (185,000 xg, 1 h, 4°C). Membrane proteins were loaded on a pre-equilibrated 2x 1 ml StrepTrap HP column (GE Healthcare) and eluted with 2.5 mM desthiobiotin. Peak fractions were concentrated with a 10 kDa concentrator and further purified on an S200 size exclusion chromatography column. For antibody generation, protein was diluted in 1x PBS + 0.05% DDM (final concentration 0.3 mg/ml). Rabbit immunization and antibody production were performed by ImmunoGlobe GmbH (Himmelstadt, Germany).

### LFQ protein quantification by mass spectrometry

For label-free quantification, protein samples were reduced in 1x Laemmli buffer (BioRad) containing 50 mM dithiothreitol (DTT, Thermo Scientific; 5 min, 95°C). Proteins were alkylated with 120 mM iodoacetamide (20 min, room temperature, light-protected) then precipitated and washed three times in ice-cold acetone. Protein pellets were dissolved in 100 mM ammonium bicarbonate containing 0.5% (w/v) sodium deoxycholate (Sigma-Aldrich). Digestions were performed with the lysyl endopeptidase LysC (Wako; 1 h, 30°C), followed by treatment with trypsin (Promega; overnight, 37°C). Sodium deoxycholate was removed by ethylacetate extraction [88] and samples were dried using a vacuum concentrator. Peptides were desalted using C18 stage tips with five C18 Empore SPE disks (3M) and eluted with 60% (v/v) acetonitrile/0.1% (v/v) formic acid. NanoLC-MS/MS analyses were performed on an Orbitrap Fusion instrument equipped with an EASY-Spray Ion Source coupled to an EASY-nLC 1000 (Thermo Scientific). Peptides were loaded on a trapping column (2 cm x 75 μm ID, PepMap C18, 3 μm particles, 100 Å pore size) and separated on an EASY-Spray column (25 cm x 75 μm ID, PepMap C18, 2 μm particles, 100 Å pore size) with a 120 min linear gradient from 3 to 32% acetonitrile and 0.1% formic acid. MS scans were acquired in the Orbitrap analyzer at 120,000 resolution at m/z 200. Data-dependent MS/MS scans were measured by a Top Speed method (cycle time 3 s in the ion trap analyzer, with rapid scan rate and HCD fragmentation with 35% normalized collision). Dynamic exclusion was applied for 60 s; isotopes, singly charged precursors and charge states >7 were excluded from selection. Minimum signal threshold for precursor selection was 1 x 10^4^. Predictive AGC was used with a target value of 2 x 10^5^ for MS scans. For MS/MS scans, recommended universal method settings were applied (AGC target 3 x 10^3^, max. injection time 0.25 s, injection of ions for all available parallelizable time). EASY-IC was used for internal calibration. Data analysis was performed using MaxQuant v1.5.3.30.

### Bacterial two/three-hybrid assays

Bacterial two- and three-hybrid assays were performed using a kit (Euromedex) and screened as reported [25,57]. Briefly, plasmids were transformed in the BTH101 strain and selected on LB plates containing ampicillin and kanamycin. Single colonies were picked and grown at 30°C in liquid LB medium with antibiotics. After overnight growth, 2 μl were spotted on LB plates with ampicillin, kanamycin, 0.5 mM IPTG and 40 μg/ml X-Gal and incubated (48 h, 30°C). For quantification of interactions, single transformants were picked and grown in 1 ml LB medium (48 h, 30°C) with antibiotics and inducer (0.5 mM IPTG), and β-galactosidase activity was determined in Miller Units, as described [89].

### Recombinant protein expression and size exclusion chromatography

For recombinant expression of Esx-proteins (EsxA-EsxD) and YtnP, the pET20b(+) vector containing the respective His-tagged protein was transformed in *E*. *coli* BL-21 DE3 Gold (Stratagene). Cells were grown to an optical density of OD_600_ = ~0.6 and expression of recombinant proteins was induced with 1 mM IPTG (4–5 h, 37°C). Cell pellets were resuspended in 50 mM Tris-HCl pH 7.5, 500 mM NaCl, 20 mM imidazole, 10% (v/v) glycerol, 1% (v/v) Tween-20 and 0.2 μg/ml lysozyme (Sigma-Aldrich; 10 min, 37°C), then lysed mechanically in a fast-prep shaker (two times, 45 s each, 6.5 m/s) and lysate was cleared by centrifugation (10,000 xg, 10 min, 4°C). Lysates were mixed with pre-equilibrated Ni-NTA resin (Qiagen) and incubated (30 min, 4°C with mild agitation). The resin was washed twice in 50 mM Tris-HCl pH 7.5, 500 mM NaCl, 10% (v/v) glycerol and 1 mM PMSF (phenylmethylsulfonylfluoride; Sigma-Aldrich) with increasing imidazole concentrations from 20 to 50 mM. His-tagged proteins were eluted from the resin with 50 mM Tris-HCl pH 7.5, 500 mM NaCl, 1 M imidazole, 10% (v/v) glycerol and 1 mM PMSF. Imidazole was removed using PD-10 desalting columns (GE Healthcare). Proteins were supplemented with 20% (v/v) glycerol and stored at -80°C.

pBAD-*esaAessABC* was transformed in *E*. *coli* One Shot TOP10 expression strain (Life-Technologies) and if necessary, pASK-IBA3C-*floA* was co-transformed. Cells were grown at 37°C in liquid LB medium to an optical density of OD_600_ = ~0.8 and expression induced with 0.2% (w/v) L-arabinose (for pBAD plasmid) and 0.2 μg/ml anhydrotetracycline (for pASK-IBA3C) (24 h, 18°C). Cell pellets were resuspended in 50 mM Tris-HCl pH 8.0, 300 mM NaCl, 20 mM MgCl_2_ and 1 mM DTT and lysed in a French-press (3 cycles at 10,000 psi). The lysate was clarified by centrifugation (10,000 xg, 20 min, 4°C) and membrane fractions collected by ultracentrifugation (185,000 xg, 1 h, 4°C). Membranes were homogenized and solubilized in 50 mM Tris-HCl pH 8.0, 50 mM NaCl, 10 mM MgCl_2_, 1 mM EDTA, 1 mM DTT, 0.25% (w/v) n-tetradecyl β-D-maltoside (TeDM) (Anatrace). Membrane proteins were clarified by ultracentrifugation (100,000 xg 1 h, 4°C). The supernatant was loaded onto a Superose 6 10/300 column (GE Healthcare) equilibrated with 50 mM Tris-HCl pH 8.0, 50 mM NaCl, 10 mM MgCl_2_ and 0.00002% (w/v) TeDM. Eluted fractions were collected and analyzed by western blot.

### Sample preparation for SDS-PAGE and immunoblot analysis

For analysis of whole cell extracts by immunoblot, cells were grown to the stated growth phase. Cells equivalent to 1 ml at optical density OD_600_ = 1.5 were collected by centrifugation (9000 xg, 3 min) and suspended in 50 μl lysis buffer (10 mM EDTA, 50 mM Tris-HCl pH 7.5) supplemented with 50 μg/ml lysostaphin (Ambi Products) and 1 mM PMSF. After incubation (37°C, 30 min), 50 μl 2x Laemmli buffer were added and samples boiled (10 min, 95°C). Depending on protein, 5–20 μl were loaded on an SDS-PAGE gel for subsequent immunoblot analysis.

Culture supernatants were filter-sterilized with 0.25 μm syringe filters and precipitated with 5% trichloroacetic acid (overnight, 4°C), washed with ice-cold acetone, and resuspended in 1x Laemmli buffer. Samples were boiled (10 min, 95°C) and proteins equivalent to 0.4 ml (for YtnP detection), 0.6 ml (for EsxC) or 1.6 ml culture supernatant (for FLAG) were resolved on 18% SDS-PAGE gels with a Tris-Tricine buffer system.

Western blot was performed using standard protocols for semi-dry and wet-blot methods. Proteins were transferred to a PVDF membrane, blocked with 5% (w/v) non-fat dried milk powder in TBS-T, incubated with primary antibodies overnight, followed by 1 h incubation with secondary antibodies. Antibodies were used as follows: anti-mCherry (1:5000; BioVision), -GFP (1:5000; Takara), -FLAG (1:1000; Sigma-Aldrich), -EsxC (1:2000; [33]), -EssC (1:10,000; [33]), -EsaA (1:10,000; [33]), -EssB (1:4000), -YtnP (1:1000; [60]), -GroEL (1:5000, Sigma-Aldrich), -FloA (1:10000; [55]), chicken IgY-HRP (1:2,500; Life Technologies), -mouse IgG-HRP (1:10,000; Life Technologies) and -rabbit IgG-HRP (1:20,000; Bio-Rad). Polyclonal antibodies to EsxC, EsaA and EssC were a kind gift of Tracy Palmer (School of Life Science, University of Dundee, Scotland). Image processing was performed using ImageJ software [90]. Signals were quantified with the ImageJ Gel-Analyzing tool.

### Isolation of crude membrane fraction of *S*. *aureus*

For isolation of the crude membrane fraction, cells were grown in 50 ml liquid TSB medium overnight with vigorous agitation. Cells were collected by centrifugation (4000 xg, 15 min) and washed in PBS. If required, cells were chemically crosslinked using amine-reactive crosslinker DSP (dithiobis(succinimidyl propionate); Thermo Scientific) prior to lysis. For crosslinking, cells were resuspended in 10 ml PBS supplemented with 1 mM DSP and incubated on ice for 2 h. Crosslinking reaction was quenched by adding Tris-HCl pH 7.5 to a final concentration of 20 mM (on ice, 15 min). Cells were then collected and resuspended in 10 ml PBS lysis buffer (PBS, 250 mM sucrose, 1 mM EDTA) supplemented with 50 μg/ml lysostaphin, 1 mM PMSF followed by incubation (37°C, 10 min). Cells were lysed mechanically with glass beads in a fast-prep shaker (twice 45 s each with 6.5 m/s); debris and unbroken cells were removed by centrifugation (11,000 xg, 10 min, 4°C). The supernatant containing cytosolic and membrane proteins was ultracentrifuged (100,000 xg, 1 h, 4°C). Pelleted membrane proteins were resuspended in PBS + 250 mM sucrose and 10% (v/v) glycerol, flash-frozen in liquid nitrogen and stored at -80°C.

### DRM-DSM isolation

To isolate detergent-resistant and -sensitive membrane (DRM and DSM) fractions, we used the Cellytic MEM protein extraction kit (Sigma-Aldrich) according to manufacturer’s protocols with minor modifications. Briefly, the crude membrane fraction was isolated as above and 1 μg total protein was mixed with kit lysis and separation buffer. After separating DRM from DSM proteins, DRM was washed three times and fractions precipitated with acetone, air-dried (5 min), suspended in 200 μl Laemmli buffer, and 10-20 μl loaded on an SDS-PAGE gel.

### Co-immunoprecipitation (Ni-NTA, FLAG capture)

His- or FLAG-tagged proteins were co-immunoprecipitated with Ni-NTA resin (Qiagen) or M2 FLAG-capture beads (Sigma-Aldrich), respectively. For immunoprecipitation with His-tagged FloA, crude membrane fractions were isolated as above, solubilized in His-binding buffer (50 mM Tris-HCl pH 7.5, 500 mM NaCl, 20 mM imidazole, 10% (v/v) glycerol, 1% (v/v) Tween-20) containing 0.25% DDM. Insoluble material was removed by centrifugation (100,000 xg, 1 h, 4°C). Solubilized protein (1 mg) was incubated with 150 μl Ni-NTA resin and rotated (2 h, 4°C), washed twice in buffer W1 (50 mM Tris-HCl pH 7.5, 500 mM NaCl, 20 mM imidazole, 10% (v/v) glycerol), twice in buffer W2 (50 mM Tris-HCl pH 7.5, 500 mM NaCl, 50 mM imidazole, 10% (v/v) glycerol), and eluted with 200 μl elution buffer (50 mM Tris-HCl pH 7.5, 500 mM NaCl, 1 M imidazole, 10% (v/v) glycerol). The eluted fraction was concentrated by TCA precipitation before SDS-PAGE and immunoblot.

To capture FLAG-tagged proteins, crude membrane fractions were solubilized in 50 mM Tris-HCl pH 7.5, 50 mM NaCl supplemented with 0.25% DDM. After removal of insoluble material (100,000 xg, 1 h, 4°C), a total of 4 mg protein was mixed with 20 μl pre-equilibrated beads. After rotation (2 h, 4°C), beads were washed four times in 50 mM Tris-HCl pH 7.5, 50 mM NaCl with decreasing amounts of detergent (two washes with 0.125% DDM, two washes with 0.02% DDM). Captured FLAG-tagged proteins were eluted by boiling beads in 50 μl Laemmli buffer, followed by SDS-PAGE and immunoblot.

### BN-PAGE

Blue Native PAGE (BN-PAGE) was performed with the Novex NativePAGE Bis-Tris system (Life Technologies), used according to manufacturer’s protocols with minor modification. Briefly, crude membrane fractions were isolated as described above and solubilized (overnight, 4°C) in 1x Native PAGE sample buffer with 0.25% DDM. Insoluble material was removed by centrifugation (20,000 xg, 30 min, 4°C) and 150 μg solubilized membranes were loaded on 3–12% gradient gels. After electrophoresis and blotting, PVDF membranes were fixed (15 min, 8% acetic acid), air-dried and rewetted in methanol before blocking and antibody incubation.

### Microscopy

For microscopy analysis, cells were harvested from liquid TSB culture, washed twice with PBS and fixed with 4% paraformaldehyde (5 min). After two additional washes in PBS, cells were spotted on an agarose pad (0.8% agarose in PBS) to immobilize them for image acquisition. Epifluorescence microscopy was performed using a Leica DMI6000B microscope, equipped with a Leica CRT 60000 illumination system and DFC630FX camera. Raw images were processed and deconvoluted using image processing software Leica LAS AF v3.7.

For super-resolution stimulated emission depletion (STED) microscopy, immunofluorescent staining of the samples was guided by [91]. Δ*spa* mutant cells were grown to mid-exponential phase and fixed with 4% paraformaldehyde for 30 min. After washing with PBS, cells were resuspended in GTE buffer (50 mM glucose, 20 mM Tris-HCl pH 7.5, 10 mM EDTA pH 8) and mounted on 0.1% poly-L-lysine-coated coverslips. Samples were treated with 10 μg/ml lysostaphin in GTE for 5 min, washed with PBS and incubated in 70% EtOH for 10 min. Coverslips were air dried and blocked with 5% BSA for 30 min. Blocking solution was removed and the primary antibody (anti-EssB 1:25 and anti-FloA 1:100) was incubated overnight in 0.5% BSA at 4°C. For each experiment a negative control was incubated without the primary antibody. Coverslips were washed with PBS and the Alexa conjugated secondary antibody was incubated for 2 h in the dark at room temperature (anti-rabbit-Alexa546 1:250, anti-chicken-Alexa488 1:500, ThermoFisher). After washing with PBS, coverslips were air dried and mounted on microscope slides with 3 μl of Prolong Gold Antifade Mountant (ThermoFisher). Slides were incubated in the dark overnight and images taken with a Leica SP8 TCS STED 3X system. Dual channel images were obtained exciting Alexa 546 and Alexa 488 at 561 nm and 488 nm respectively, with a pulsed white light laser depleting at 660 nm and 592 nm, respectively. Deconvolution of z-stacks was obtained with the software Huygens Professional and colocalization analysis was performed with the Costes’ threshold function of the JACoP Plugin for ImageJ [78,92].

### Animal experiments and ELISA

Cohorts of 3-week-old BALB/c mice (*n* = 6) were infected intravenously with 100 μl of suspension containing 10^6^ CFU staphylococci in PBS. For anti-FMM treatment, 20 mg/kg zaragozic acid (in PBS) was administered intraperitoneally 30 min prior to challenge with staphylococci. The procedure was repeated twice (days 14 and 28). Mice were sacrificed after 40 days and blood samples collected by cardiac puncture. Serum EsxA-D and IsaA antibody titers were examined by ELISA. Briefly, recombinant His-tagged Esx proteins and IsaA were purified as above, diluted in PBS to 10 μg/ml final concentration and coated on 96-well plates (Nunc, MaxiSorp). Plates were incubated (overnight, 4°C) and blocked with 5% BSA (bovine serum albumin) in PBS. Serum samples were diluted (1:50 in PBS), incubated on plates (1 h, 37°C); plates were washed with PBS-T (PBS + 0.05% Tween) and incubated with anti-mouse IgM antibodies (1:5000; Life Technologies; 1 h, 37°C). After a final wash with PBS-T, 100 μl TMB (3,3’,5,5’-tetramethylbenzidine; Life Technologies) was used to develop the reaction, which was terminated with 100 μl 1 N NaOH and absorbance measured at 450 nm.

### Ethics statement

All experimental animal studies were approved by the Committee on the Ethics of Animal Experiments of the government of Lower Franconia (55.2-2532-2-57) and were in strict accordance with the guidelines for animal care and experimentation of German Animal Protection Law and EU Directive 2010/63/EU. Mice were housed in cages in standardized lighting conditions and had ad libitum access to food and water. All efforts were made to minimize suffering and animals were sacrificed at the end of the experiment by CO_2_ inhalation.

### Statistical methods

Statistical analysis was performed with GraphPad Prism (GraphPad software, version 7) using appropriate statistical methods as indicated in the figure legends. P-values ≤0.05 were considered significant. Pairwise comparisons were assessed using unpaired Student’s t-test. Analysis of variance (ANOVA) was performed to determine whether a group of means was significantly different from each other.

## Supporting information

S1 Fig Label-free quantification (LFQ) analysis of the *S*. *aureus* membrane proteome in stationary growth phase. (A) Scatterplot shows LFQ intensities of DRM proteins plotted against DSM proteins. Pink represents DSM proteins, which are exclusively found in DSM (dark pink; population I) or DSM enriched (light pink; population II). Green represents DRM proteins, which are enriched (light green; population III) or found exclusively (dark green; population IV) in the DRM fractions. Arrows indicate T7SS membrane proteins EsaA, EssA, EssB and EssC and the FMM scaffold protein FloA. (B) Distribution of ABC transporter components, histidine kinases and divisome proteins within the four populations shown in (A). Underlined protein (FtsA) was further analyzed in panel (D). (C) Sequence analysis of selected proteins of each fraction. Bar graphs show mean size of proteins (left) and mean length of transmembrane domain (TMD; center). TMD length was determined with Phobius TMD prediction tool. Boxplot (right) shows ΔGapp of individual TMDs of all fractions, determined with the ΔG prediction server. (D) Example of a membrane-associated protein (FtsA) not affected by FMM/FloA. Left panel shows a growth curve of wild type and Δ*floA* strain in TSB medium. Center and right panels show bacterial two-hybrid analysis of FloA and FtsA. Center panel shows blue/white screening of a bacterial two-hybrid analysis of FloA and FtsA; right panel shows the corresponding quantification of β-galactosidase activity. Positive control plasmids were provided by manufacturer; negative controls are empty plasmids.
(TIFF)

S2 Fig Bacterial two-hybrid analysis to study FloA interaction with T7SS membrane proteins. Quantification of the interaction with β-galactosidase activity assay of flotillin with T7SS proteins EsaA, EssA, EssB and EssC. Positive controls are plasmids provided by manufacturer and negative controls are empty plasmids.
(TIFF)

S3 Fig Synthetic strains render functional T7SS. Immunoblot analysis of culture supernatants of Δ*essA* and Δ*essB* and complementation with EssA-MARS and FLAG-/GFP-EssB, respectively. Cells were grown to early stationary growth phase, sterile-filtered supernatants were precipitated and amount corresponding to 0.6 ml culture was used for immunoblot and detected with anti-EsxC antibodies.
(TIFF)

S4 Fig Stability of T7SS membrane proteins in flotillin-deficient background. (A) Wild type and mutants were grown overnight; 20 μl of cell extracts were used for immunoblot analysis and detected with antibodies against EsaA, EssB and EssC. Detection of GroEL served as loading control. (B) Wild type and Δ*floA* mutant expressing complemented EssA-MARS construct were grown over night and 20 μl of cell extracts were loaded on gel. Immunoblot analysis was performed using anti-mCherry antibody. An unlabeled wild type strain served as negative control and GroEL was detected as a loading control.
(TIFF)

S5 Fig Solubilization and crosslinking of T7SS proteins. (A) Western blot analysis to determine extraction of T7SS membrane proteins and flotillin from *S*. *aureus* crude membranes using 0.25% DDM. Equal amounts of crude membranes and soluble (sol.) and insoluble (insol.) material were loaded on SDS-PAGE gel and detected with polyclonal antibodies directed against EsaA, EssB, EssC or FloA. EssA-MARS was detected using polyclonal antibodies against mCherry. (B) *In vivo* crosslinking with 1 mM DSP reveals oligomeric pattern of EssB. Stationary cells were treated with 1 mM DSP, lysed and isolated crude membrane fraction was solubilized with 0.25% DDM. Subsequently, solubilized proteins were mounted on a BN-PAGE gel and detected using polyclonal antibodies against EssB.
(TIFF)

S6 Fig Bacterial three-hybrid assay to study EssB interaction with other membrane-bound T7SS components, alone or with flotillin on a third plasmid (pSEVA). T25-EssB fusion was tested for interaction against C- and N-terminal fusions of the T18 fragment. Interactions were assayed with empty plasmid (pSEVA641), plasmid bearing flotillin (pSEVA641*-floA*), or absence of the pSEVA plasmid. Interaction was quantified using β-galactosidase activity assay. The negative control carries empty bacterial two-hybrid plasmids.
(TIFF)

S7 Fig Indirect ELISA to show serum titers of the cell-wall associated protein IsaA. (A) BALB/c mice were challenged with wild type, Δ*floA*, ΔT7SS and wild type cells treated with 50 μM ZA at day 0, 14 and 28 and serum was collected after 40 days by cardiac puncture. IgM titers to IsaA were determined by indirect ELISA. Absorbance corresponds to 1:50 diluted sera.
(TIFF)

S8 Fig Distribution of FloA-MARS foci after treatment with anti-FMM molecules. Fluorescence microscopy images of *S*. *aureus* cells expressing FloA-MARS were grown until late-exponential growth phase in the absence of anti-FMM molecules (top row) and with 150 μM 5-doxyl-stearic acid (5-DSA), 20 μM simvastatin (SIM) or 50 μM zaragozic acid (ZA). Left panel shows bright field images; center panels, epifluorescence and corresponding deconvoluted fluorescent signals of FloA-MARS. The right panel shows a merge of brightfield channel and deconvoluted fluorescent signal, with the fluorescence signal false-colored in red. Bar, 2 μm.
(TIFF)

S9 Fig Treatment with anti-FMM molecules does not affect T7SS membrane protein abundance. Immunoblot analysis to determine protein levels of EsaA, EssB and EssC in the presence of 20 μM SIM, 150 μM 5-DSA or 50 μM ZA. Cells were grown overnight and whole cell extracts were loaded on a SDS-gel for immunoblot analysis using polyclonal antibodies directed against EsaA, EssB or EssC. A strain lacking the entire T7SS (Δ*T7SS*) operon served as a negative control strain. Immunoblot against GroEL was used as a loading control.
(TIFF)

S1 Table Proteins identified in label-free quantification (LFQ) analysis. (PDF)

S2 Table Strains and plasmids used in this study. (PDF)

S3 Table Primers used in this study. (PDF)
