# Supplementary figures and images for: Flotillin scaffold activity contributes to type VII secretion system assembly in Staphylococcus aureus
Source: PLoS Pathog. 2017 Nov 22;13(11):e1006728. doi: 10.1371/journal.ppat.1006728 (PMC5718613; doi:10.1371/journal.ppat.1006728)

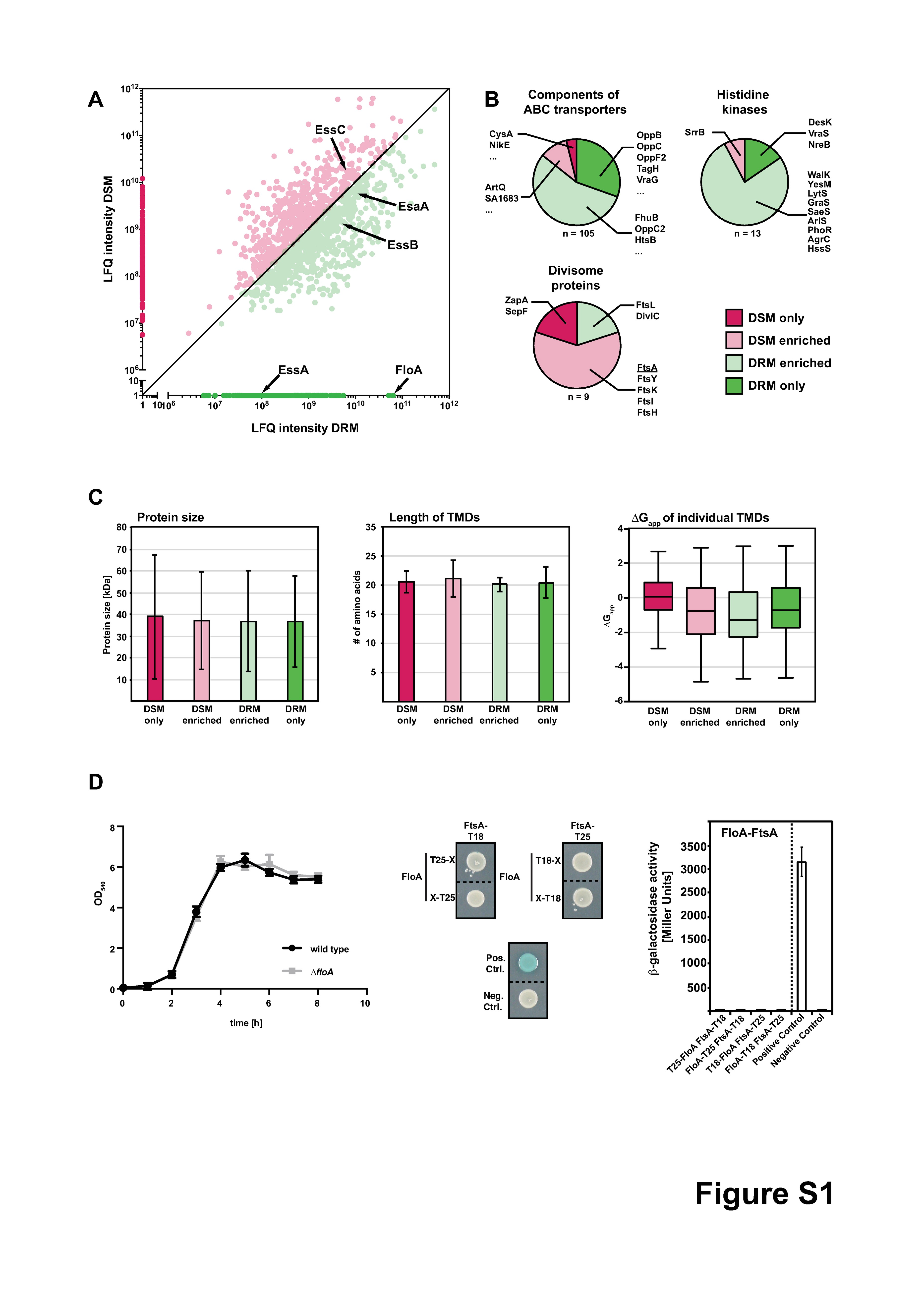

Supplement: S1 Fig — (A) Scatterplot shows LFQ intensities of DRM proteins plotted against DSM proteins. Pink represents DSM proteins, which are exclusively found in DSM (dark pink; population I) or DSM enriched (light pink; population II). Green represents DRM proteins, which are enriched (light green; population III) or found exclusively (dark green; population IV) in the DRM fractions. Arrows indicate T7SS membrane proteins EsaA, EssA, EssB and EssC and the FMM scaffold protein FloA. (B) Distribution of ABC transporter components, histidine kinases and divisome proteins within the four populations shown in (A). Underlined protein (FtsA) was further analyzed in panel (D). (C) Sequence analysis of selected proteins of each fraction. Bar graphs show mean size of proteins (left) and mean length of transmembrane domain (TMD; center). TMD length was determined with Phobius TMD prediction tool. Boxplot (right) shows ΔGapp of individual TMDs of all fractions, determined with the ΔG prediction server. (D) Example of a membrane-associated protein (FtsA) not affected by FMM/FloA. Left panel shows a growth curve of wild type and ΔfloA strain in TSB medium. Center and right panels show bacterial two-hybrid analysis of FloA and FtsA. Center panel shows blue/white screening of a bacterial two-hybrid analysis of FloA and FtsA; right panel shows the corresponding quantification of β-galactosidase activity. Positive control plasmids were provided by manufacturer; negative controls are empty plasmids. (TIFF) [file ppat.1006728.s001.tiff]

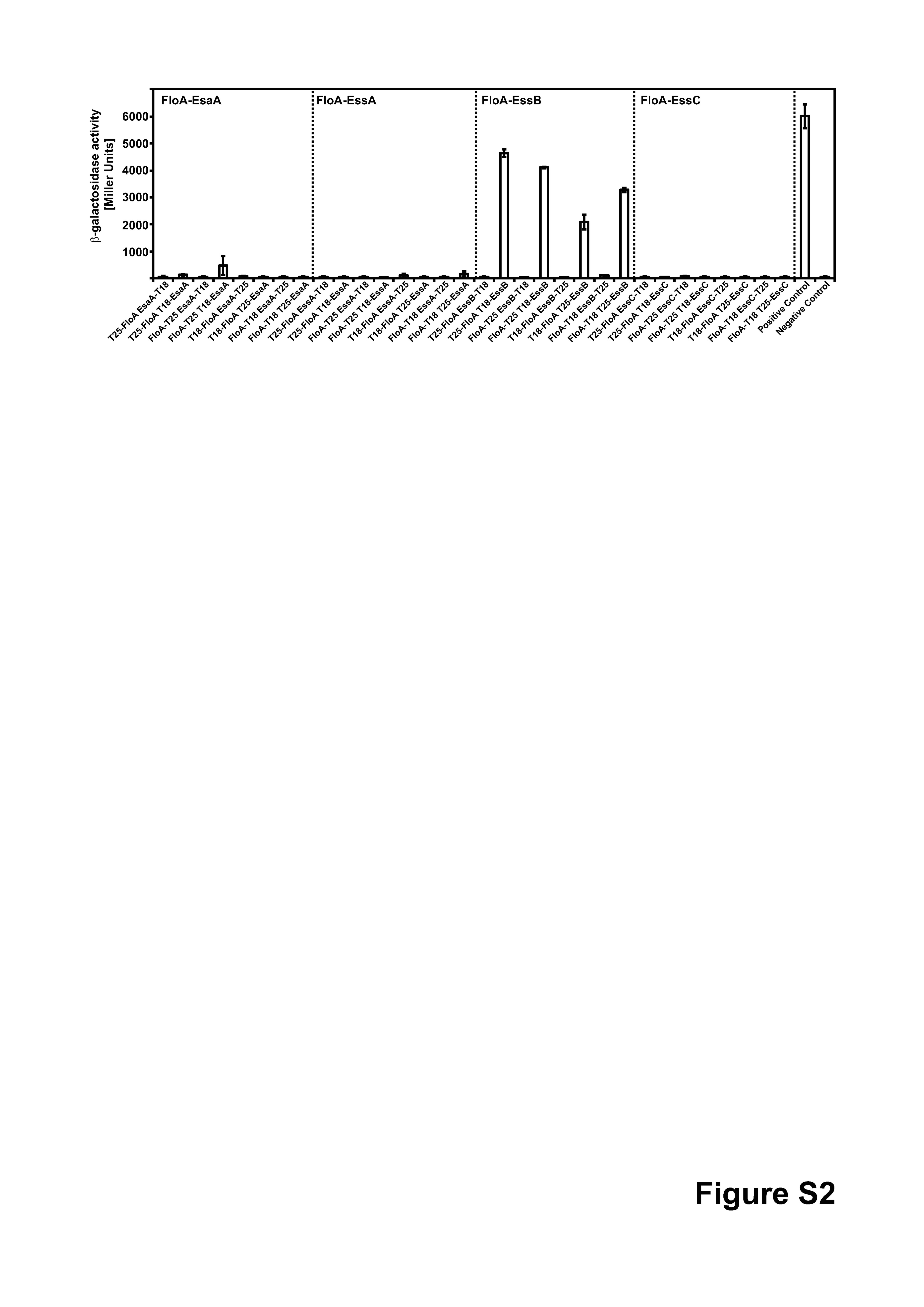

Supplement: S2 Fig — Quantification of the interaction with β-galactosidase activity assay of flotillin with T7SS proteins EsaA, EssA, EssB and EssC. Positive controls are plasmids provided by manufacturer and negative controls are empty plasmids. (TIFF) [file ppat.1006728.s002.tiff]

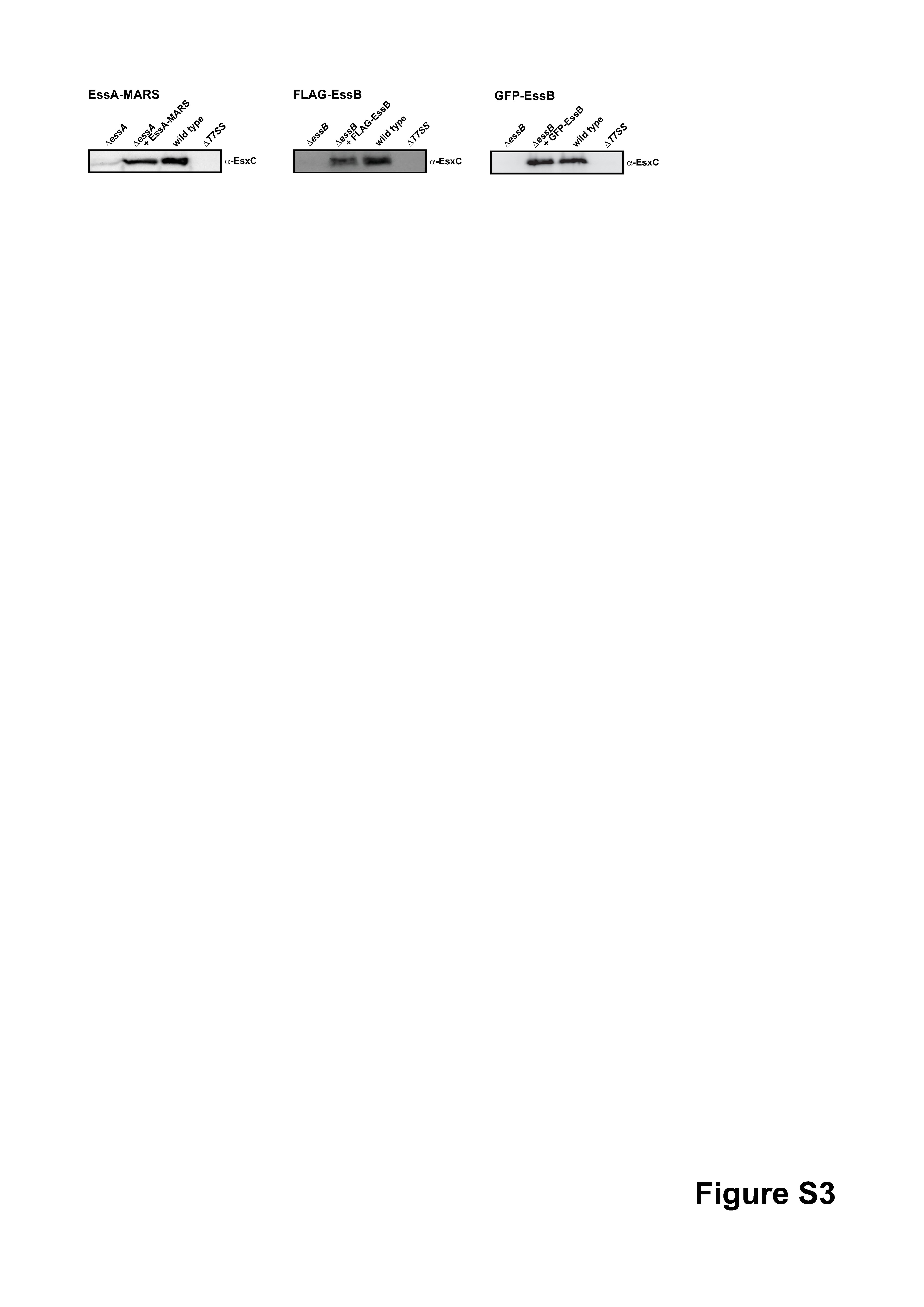

Supplement: S3 Fig — Immunoblot analysis of culture supernatants of ΔessA and ΔessB and complementation with EssA-MARS and FLAG-/GFP-EssB, respectively. Cells were grown to early stationary growth phase, sterile-filtered supernatants were precipitated and amount corresponding to 0.6 ml culture was used for immunoblot and detected with anti-EsxC antibodies. (TIFF) [file ppat.1006728.s003.tiff]

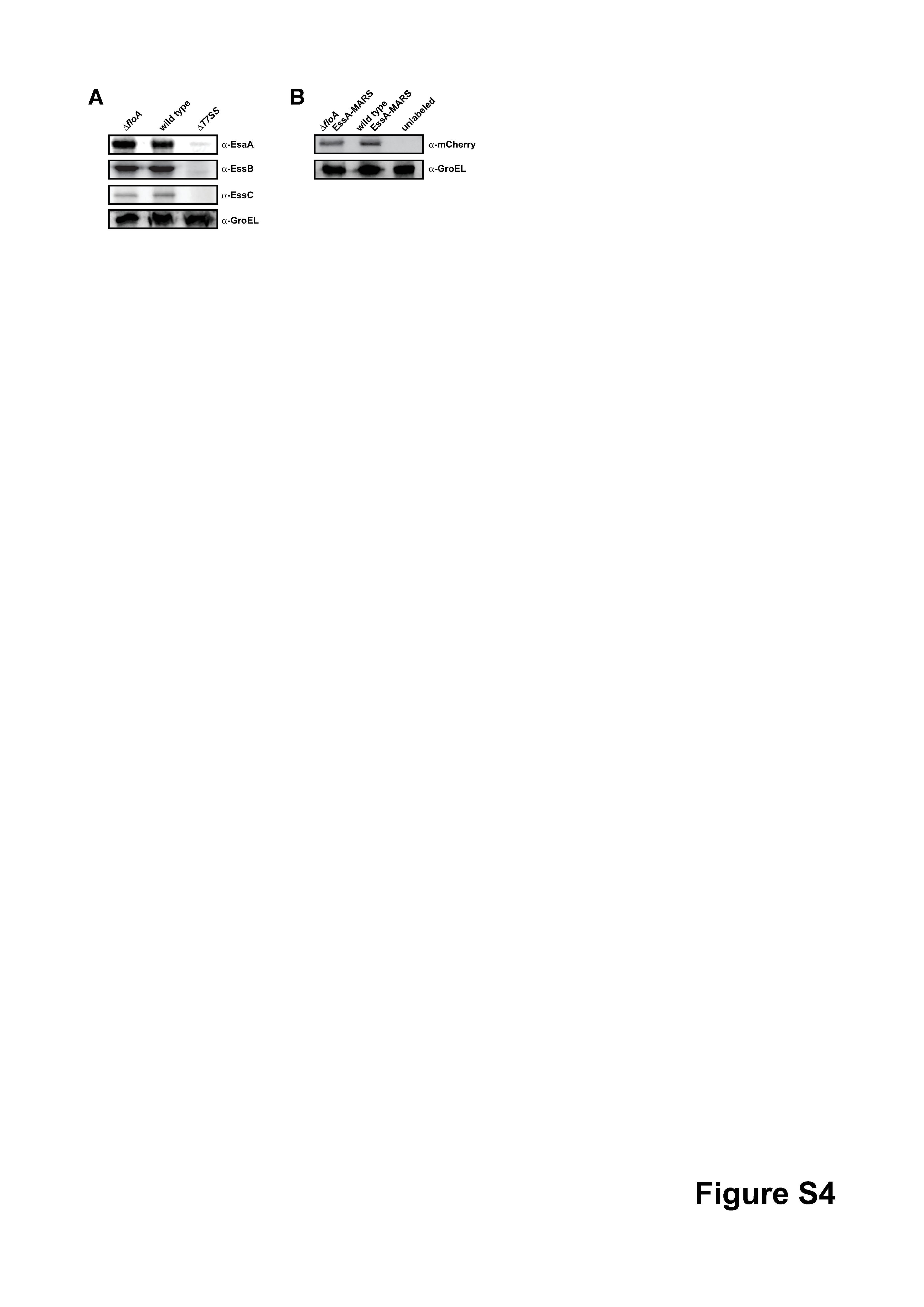

Supplement: S4 Fig — (A) Wild type and mutants were grown overnight; 20 μl of cell extracts were used for immunoblot analysis and detected with antibodies against EsaA, EssB and EssC. Detection of GroEL served as loading control. (B) Wild type and ΔfloA mutant expressing complemented EssA-MARS construct were grown over night and 20 μl of cell extracts were loaded on gel. Immunoblot analysis was performed using anti-mCherry antibody. An unlabeled wild type strain served as negative control and GroEL was detected as a loading control. (TIFF) [file ppat.1006728.s004.tiff]

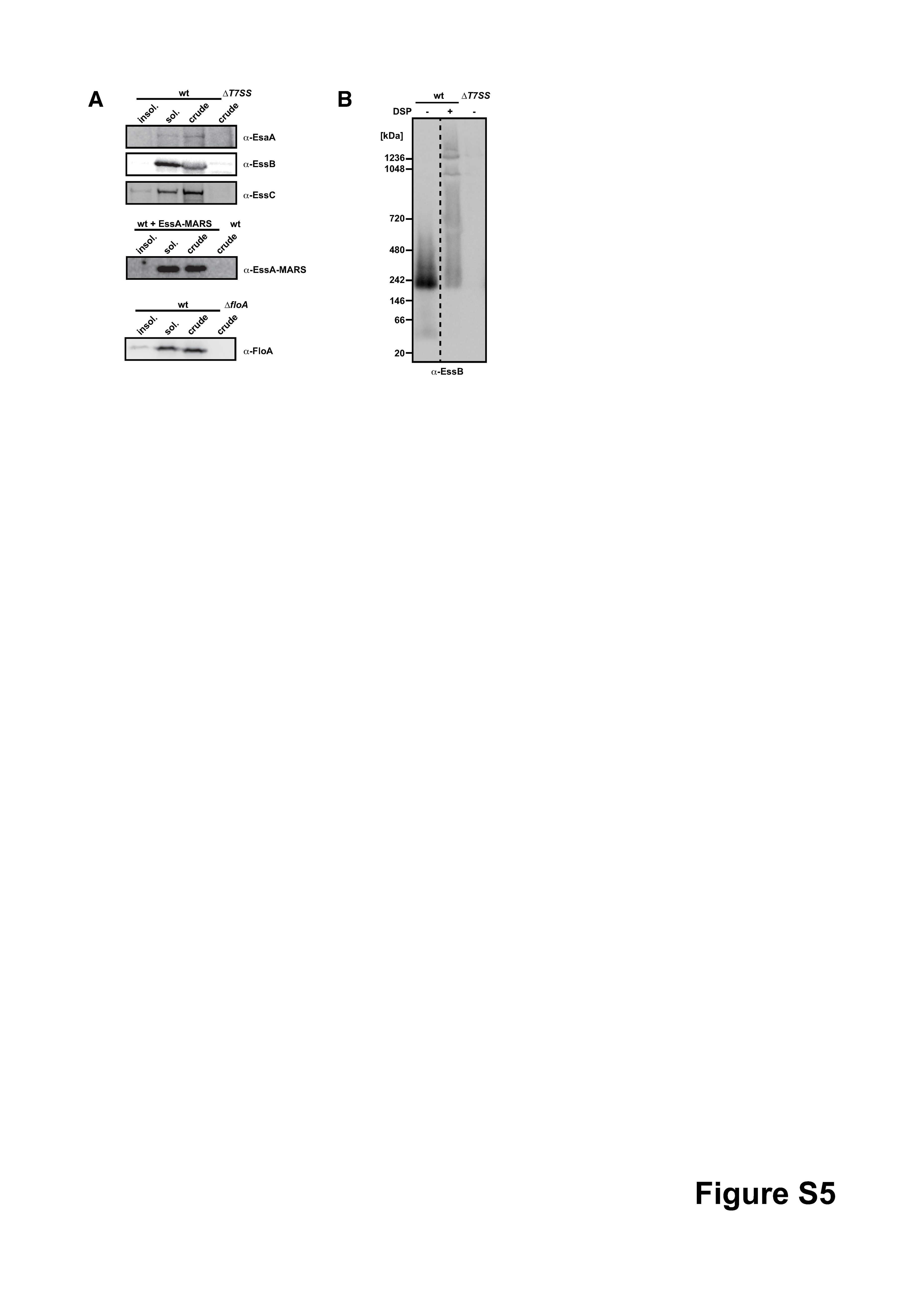

Supplement: S5 Fig — (A) Western blot analysis to determine extraction of T7SS membrane proteins and flotillin from S. aureus crude membranes using 0.25% DDM. Equal amounts of crude membranes and soluble (sol.) and insoluble (insol.) material were loaded on SDS-PAGE gel and detected with polyclonal antibodies directed against EsaA, EssB, EssC or FloA. EssA-MARS was detected using polyclonal antibodies against mCherry. (B) In vivo crosslinking with 1 mM DSP reveals oligomeric pattern of EssB. Stationary cells were treated with 1 mM DSP, lysed and isolated crude membrane fraction was solubilized with 0.25% DDM. Subsequently, solubilized proteins were mounted on a BN-PAGE gel and detected using polyclonal antibodies against EssB. (TIFF) [file ppat.1006728.s005.tiff]

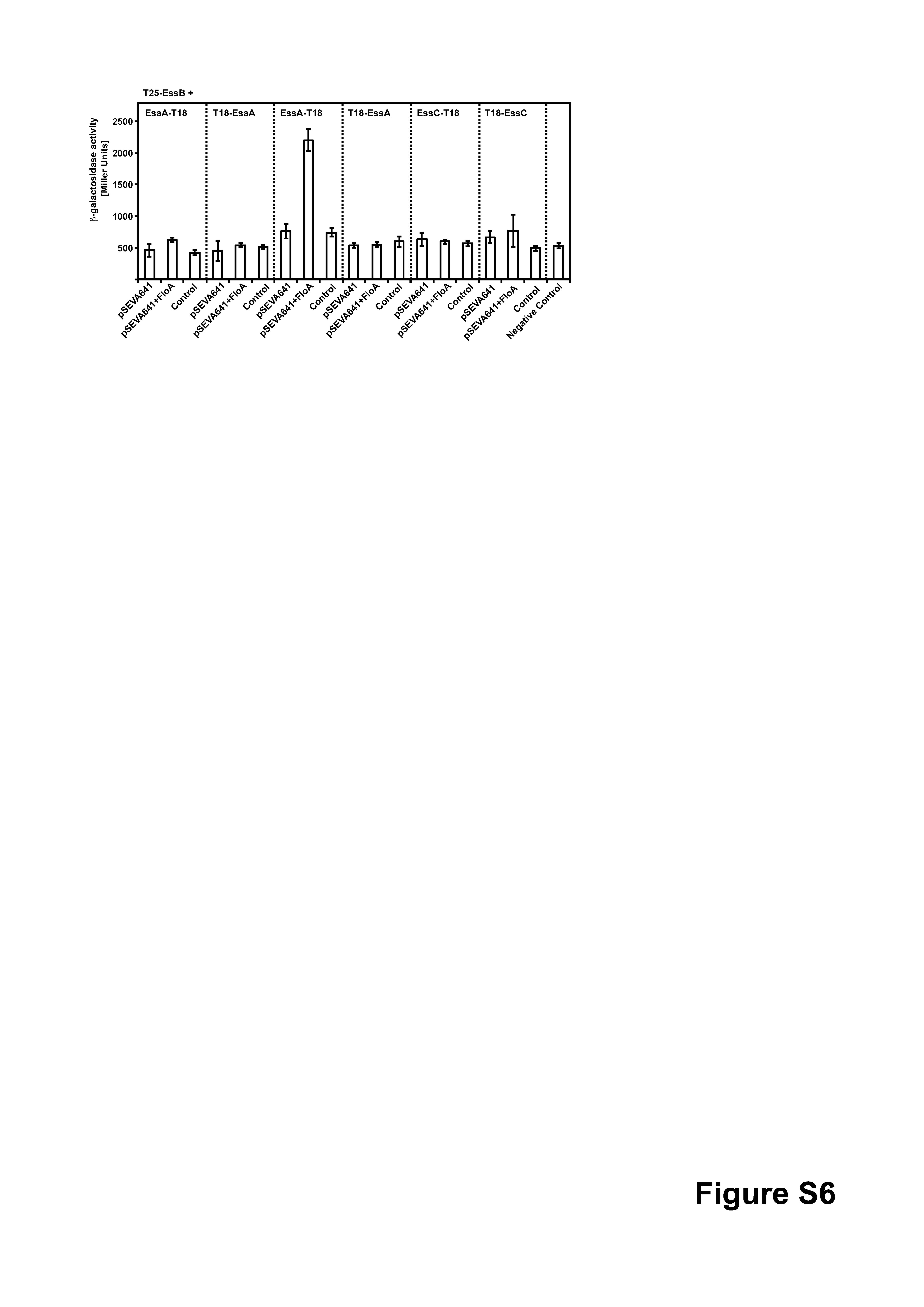

Supplement: S6 Fig — T25-EssB fusion was tested for interaction against C- and N-terminal fusions of the T18 fragment. Interactions were assayed with empty plasmid (pSEVA641), plasmid bearing flotillin (pSEVA641-floA), or absence of the pSEVA plasmid. Interaction was quantified using β-galactosidase activity assay. The negative control carries empty bacterial two-hybrid plasmids. (TIFF) [file ppat.1006728.s006.tiff]

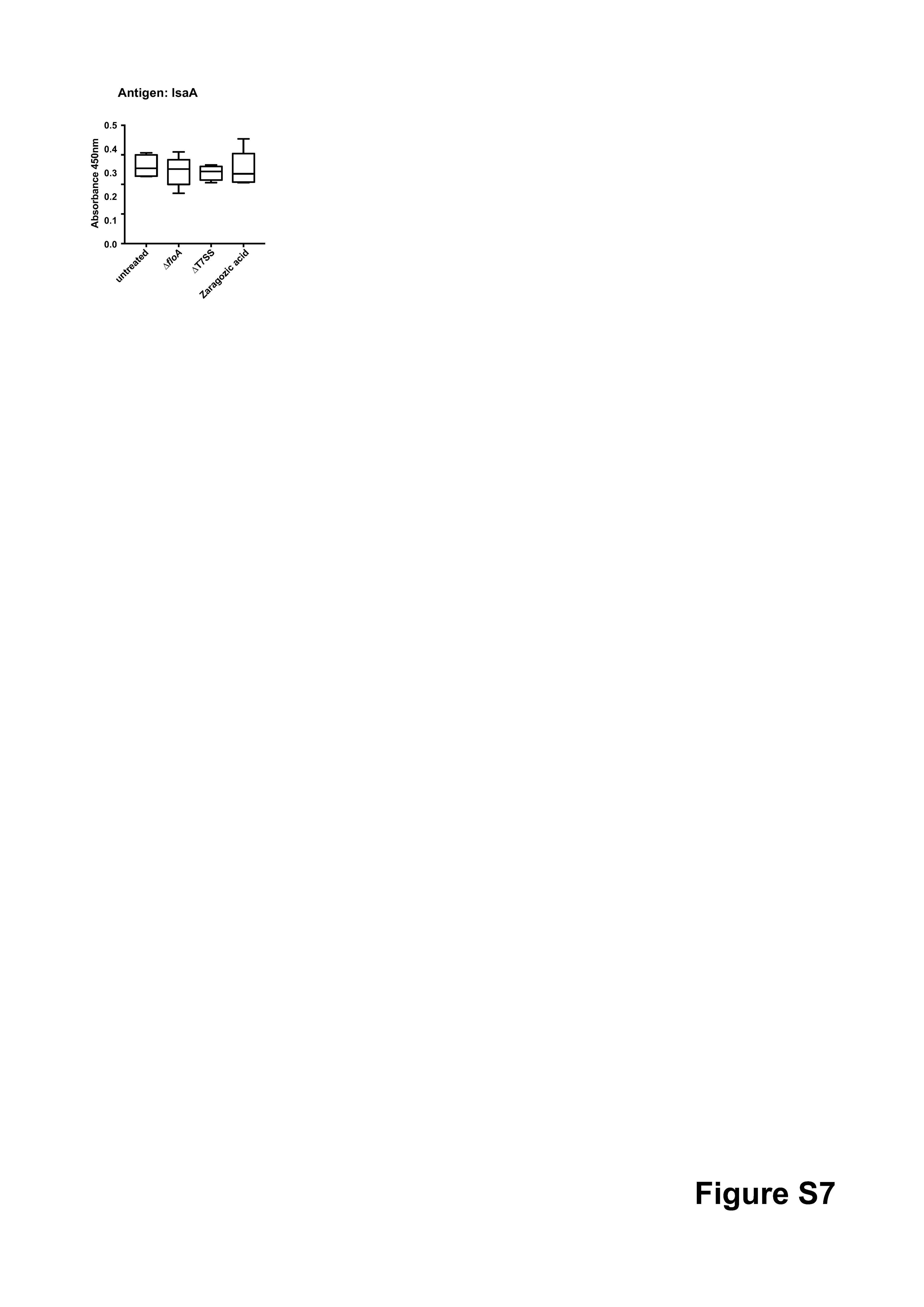

Supplement: S7 Fig — (A) BALB/c mice were challenged with wild type, ΔfloA, ΔT7SS and wild type cells treated with 50 μM ZA at day 0, 14 and 28 and serum was collected after 40 days by cardiac puncture. IgM titers to IsaA were determined by indirect ELISA. Absorbance corresponds to 1:50 diluted sera. (TIFF) [file ppat.1006728.s007.tiff]

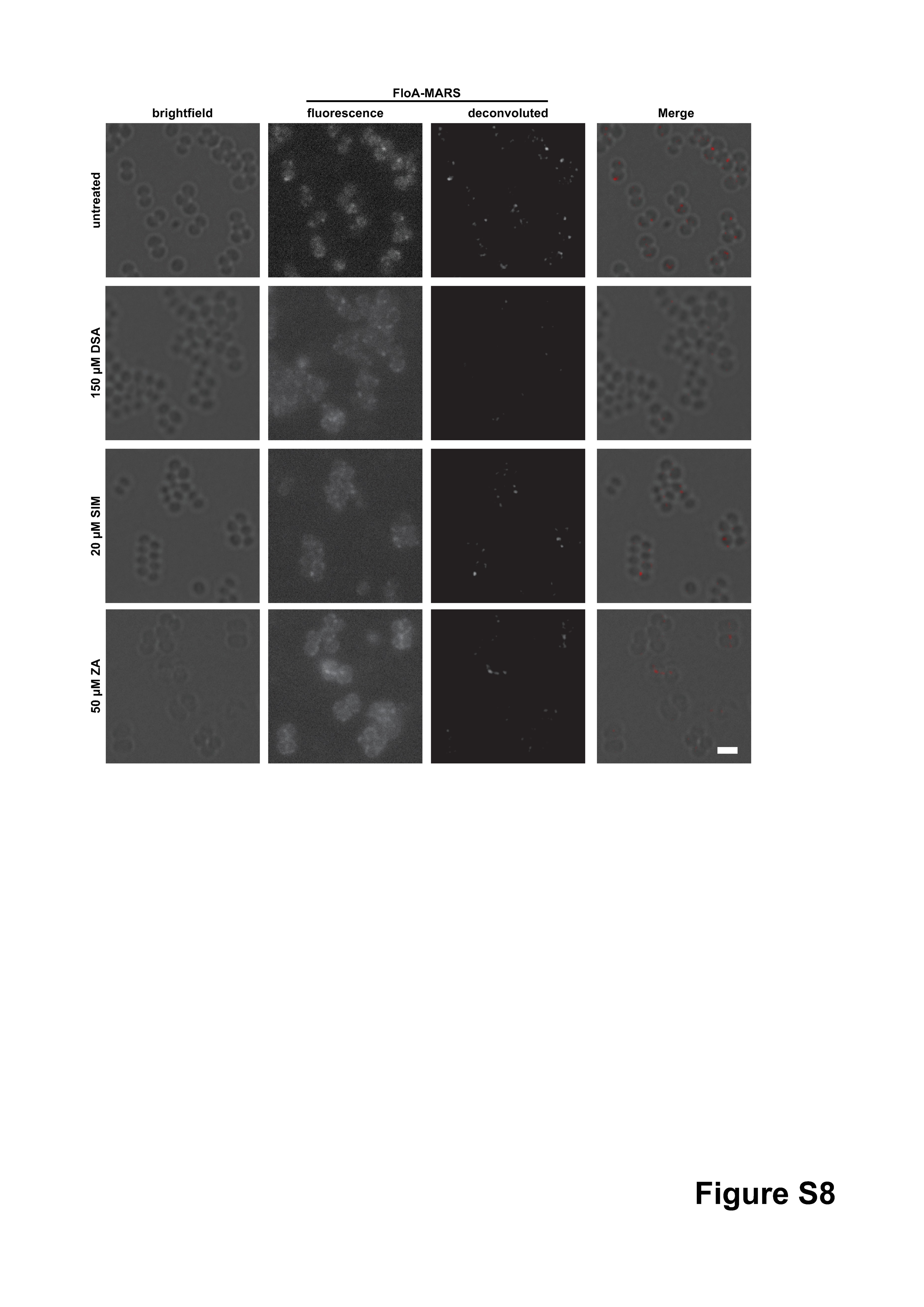

Supplement: S8 Fig — Fluorescence microscopy images of S. aureus cells expressing FloA-MARS were grown until late-exponential growth phase in the absence of anti-FMM molecules (top row) and with 150 μM 5-doxyl-stearic acid (5-DSA), 20 μM simvastatin (SIM) or 50 μM zaragozic acid (ZA). Left panel shows bright field images; center panels, epifluorescence and corresponding deconvoluted fluorescent signals of FloA-MARS. The right panel shows a merge of brightfield channel and deconvoluted fluorescent signal, with the fluorescence signal false-colored in red. Bar, 2 μm. (TIFF) [file ppat.1006728.s008.tiff]

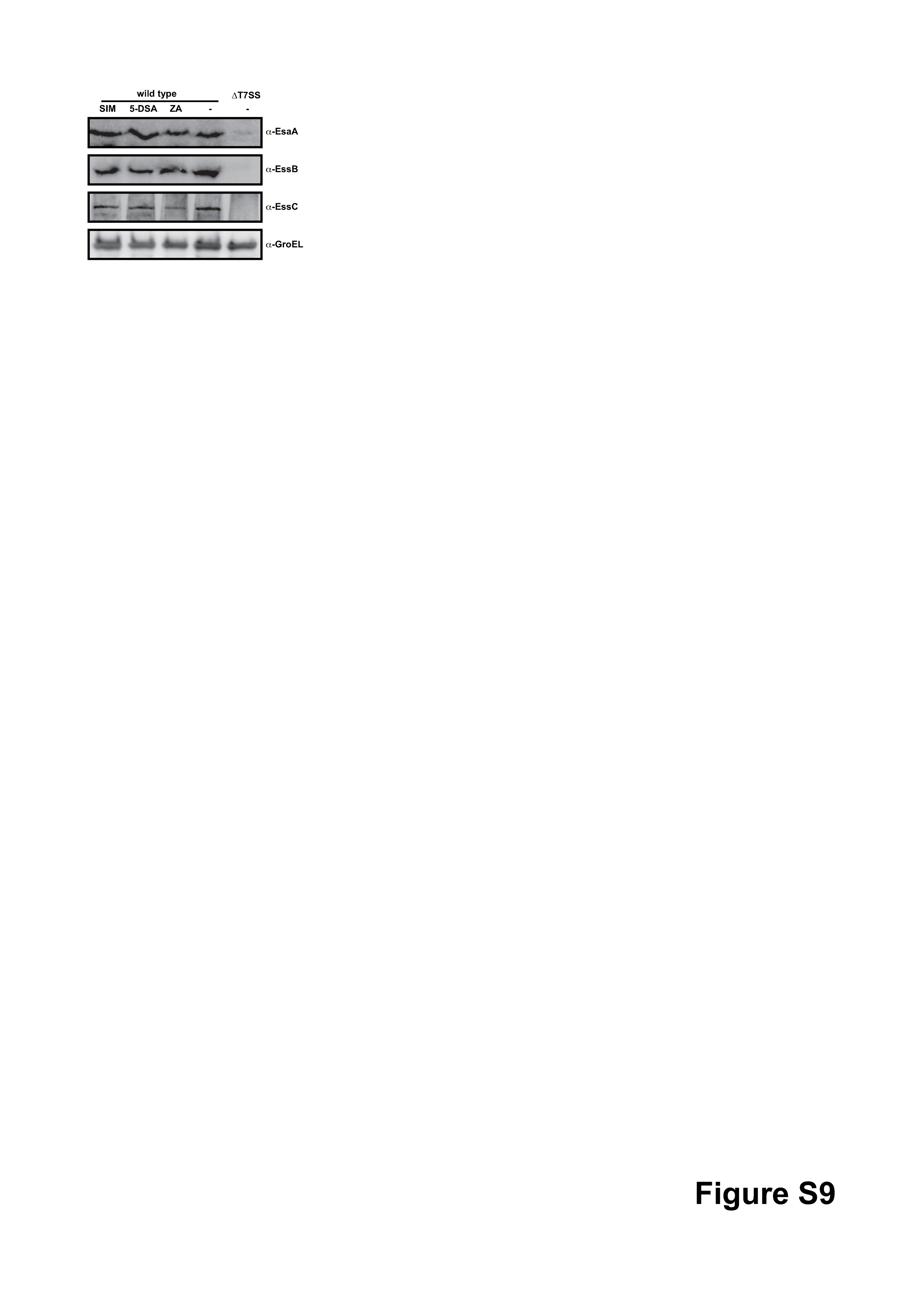

Supplement: S9 Fig — Immunoblot analysis to determine protein levels of EsaA, EssB and EssC in the presence of 20 μM SIM, 150 μM 5-DSA or 50 μM ZA. Cells were grown overnight and whole cell extracts were loaded on a SDS-gel for immunoblot analysis using polyclonal antibodies directed against EsaA, EssB or EssC. A strain lacking the entire T7SS (ΔT7SS) operon served as a negative control strain. Immunoblot against GroEL was used as a loading control. (TIFF) [file ppat.1006728.s009.tiff]
