# Supplementary material for: Flotillin scaffold activity contributes to type VII secretion system assembly in Staphylococcus aureus
Source: PLoS Pathog. 2017 Nov 22;13(11):e1006728. doi: 10.1371/journal.ppat.1006728 (PMC5718613; doi:10.1371/journal.ppat.1006728)
Supplement: S1 Table — (PDF) [file ppat.1006728.s010.pdf]

# S1_Table

## Population I. DSM only

| Uniprot-ID | Protein Name | Gene Name | LFQ Intensity DRM | LFQ Intensity |
|---|---|---|---|---|
| **A0A033UA13** | Dihydrolipoyllysine-residue succinyltransferase component of 2-oxoglutarate dehydrogenase complex | V070_02751 | 1,000E+00 | 6,892E+08 |
| **A0A033ULA4** | Heme ABC transporter, heme-binding protein isdE | V070_02180 | 1,000E+00 | 3,365E+08 |
| **A0A033UTI3** | Uncharacterized protein | V070_01525 | 1,000E+00 | 4,922E+07 |
| **A0A033UVP1** | Uncharacterized protein | V070_01199 | 1,000E+00 | 1,639E+09 |
| **A0A033V047** | Ribonuclease R | rnr | 1,000E+00 | 3,923E+08 |
| **A0A033V433** | DNA topoisomerase 4 subunit A | parC | 1,000E+00 | 4,115E+08 |
| **A0A069G604** | Probable GTP-binding protein EngB | engB | 1,000E+00 | 3,293E+08 |
| **A0A069G848** | Serine--tRNA ligase | serS | 1,000E+00 | 9,651E+07 |
| **A0A069G9N6** | Exonuclease DnaQ family/helicase DinG family | CO98_1927 | 1,000E+00 | 2,001E+08 |
| **A0A069GCH7** | ABC transporter, ATP-binding protein | CO98_2450 | 1,000E+00 | 3,091E+08 |
| **A0A069GEH3** | Catabolite control protein A | ccpA | 1,000E+00 | 1,138E+07 |
| **A0A077U6B2** | PaaD-like protein involved in Fe-S cluster assembly;DNA methyltransferase;Uncharacterized protein | paaD;ACH32_11700;NWMN_08 | 1,000E+00 | 8,277E+07 |
| **A0A0D1FUI0** | Uncharacterized protein;Putative uncharcterised protein;Putative membrane protein | ACR58_14070;ERS179246_01967;BN1326_90089 | 1,000E+00 | 4,583E+07 |
| **A0A0D1GJW4** | Arsenate reductase;Putative oxidoreductase with thioredoxin domain | ACH32_03055;yusI | 1,000E+00 | 1,207E+10 |
| **A0A0D1GV70** | 6-carboxy-5,6,7,8-tetrahydropterin synthase;6-pyruvoyl tetrahydropterin synthase;6-carboxy-5,6,7,8-tetrahydropterin synthase O | queD;ST398NM01_0788 | 1,000E+00 | 5,694E+07 |
| **A0A0D1GVC8** | GntR family transcriptional regulator;GntR family regulatory protein;Transcriptional regulator, GntR family protein | ACR58_12515;NWMN_1870 | 1,000E+00 | 2,977E+08 |
| **A0A0D1H5K7** | Putative DUF2187-containing protein | ACH32_11995 | 1,000E+00 | 3,390E+07 |
| **A0A0D1I1R5** | HTH-type transcriptional regulator rot | ACH32_01675;HMPREF0776_2797 | 1,000E+00 | 5,619E+08 |
| **A0A0D1I4Y4** | RNA-binding protein;S4 domain protein YaaA | ACH32_07050;yaaA | 1,000E+00 | 2,391E+08 |
| **A0A0D1I792** | ArsR family transcriptional regulator | czrA | 1,000E+00 | 5,241E+08 |
| **A0A0D1ID37** | Heat shock protein 15;Ribosome-associated heat shock protein implicated in the recycling of the 50S subunit | ACH32_09740;SAZ172_0508 | 1,000E+00 | 1,565E+09 |
| **A0A0D1IFV2** | Acetyltransferase;O-acetyl transferase | ACR58_06610;SAKOR_00136;ST398NM01_0170 | 1,000E+00 | 4,848E+07 |
| **A0A0D1IGG4** | DNA-binding protein;Uncharacterized protein | ACR58_02295;ST398NM01_1314;SAKOR | 1,000E+00 | 2,972E+08 |
| **A0A0D1IJ80** | Purine nucleoside phosphorylase DeoD-type | deoD;de | 1,000E+00 | 1,120E+09 |
| **A0A0D1IWZ6** | Uncharacterized protein | ACH32_12430 | 1,000E+00 | 1,008E+08 |
| **A0A0D1JL45** | XRE family transcriptional regulator | ACR58_00595 | 1,000E+00 | 3,021E+09 |
| **A0A0D1JPP9** | Peptidoglycan-binding protein LysM | QU38_13840 | 1,000E+00 | 1,476E+07 |
| **A0A0D1JT97** | Cro/Cl family transcriptional regulator;HTH domain protein%2C binds to mecA promoter region | ACH32_02150;BN1326_90082 | 1,000E+00 | 1,058E+09 |
| **A0A0D1JZR1** | HAD family hydrolase;Hydrolase (HAD superfamily) | ACR58_01155;SAKOR_01540 | 1,000E+00 | 7,562E+07 |
| **A0A0D1K2T0** | Putative cytosolic protein | ACH32_02455 | 1,000E+00 | 7,212E+07 |
| **A0A0D1K3I9** | Cold shock protein | cspC | 1,000E+00 | 4,787E+08 |
| **A0A0D3Q6G5** | Cell division protein ZapA | zapA;QU38_12010 | 1,000E+00 | 1,433E+08 |
| **A0A0D3QB11** | Uncharacterized protein | AFO87_06250;ERS179246_01401;HMPREF0776_0566 | 1,000E+00 | 4,166E+08 |
| **A0A0D6G8V3** | N-acetylglucosamine-6-phosphate deacetylase | nagA;SAOUHSC_00710 | 1,000E+00 | 1,406E+08 |
| **A0A0D6GHT2** | IolE protein-like protein;Sugar phosphate isomerase/epimerase;Uncharacterized protein | AFO87_08680;iolE;NWMN_0156 | 1,000E+00 | 1,269E+08 |
| **A0A0D6GSH2** | Precorrin-2 dehydrogenase;SA2412 protein | sirC_2;SA2412;SAKOR_02615 | 1,000E+00 | 7,870E+07 |
| **A0A0D6GY03** | Nickel transport ATP-binding protein;Peptide ABC transporter ATP-binding protein;NikE | cysA;QU38_08110;ST398NM01_2513 | 1,000E+00 | 5,250E+07 |
| **A0A0D6GYC2** | Endoglucanase M;M42 glutamyl aminopeptidase;Peptidase M28 | ysdC_1;HMPREF0776_0515;ysdC_2 | 1,000E+00 | 1,309E+09 |
| **A0A0D6GZA8** | Epimerase;MW2366 protein;SA2231 protein;NAD dependent epimerase/dehydratase family protein | AFO87_00160;MW2366;SA2231 | 1,000E+00 | 6,853E+07 |
| **A0A0D6WDP8** | Acetyl-CoA carboxylase;Acetyl-CoA carboxylase subunit (Biotin carboxyl carrier subunit); | accB_1;accB | 1,000E+00 | 1,134E+09 |
| **A0A0E0VN88** | Glyoxalase family protein;3-demethylubiquinone-9 3-methyltransferase | ST398NM01_1204;ACH32_12975 | 1,000E+00 | 2,939E+08 |
| **A0A0E0VQC1** | Uncharacterized protein | ST398NM01_1688;ACH32_00965;S | 1,000E+00 | 2,288E+07 |
| **A0A0E1AFQ2** | Uncharacterized protein;Protein of uncharacterised function (DUF1806) | SAZ172_0570;yojF;NWMN_0 | 1,000E+00 | 8,616E+07 |
| **A0A0E1AHI2** | Cell division protein SepF | sepF | 1,000E+00 | 1,112E+08 |
| **A0A0E1AIJ6** | Putative Dihydrolipoamide dehydrogenase;Uncharacterized protein;Dihydrolipoamide dehydrogenase | SAZ172_0597;SAOUHSC_00581 | 1,000E+00 | 6,977E+07 |
| **A0A0E1AJH9** | Uncharacterized protein;UPF0435 protein SAOUHSC_02093;UPF0435 protein SA1696 | SAZ172_1891;SAOUHSC_02093 | 1,000E+00 | 1,209E+08 |
| **A0A0E1VJ74** | Uncharacterized protein;ComA operon protein 2 | HMPREF0776_1933;SAZ172_0885;ydiI | 1,000E+00 | 4,498E+07 |
| **A0A0E1VLN5** | Uncharacterized protein;Uncharacterised protein | HMPREF0776_1574;AFO87_11205;BN1321_150022 | 1,000E+00 | 3,185E+08 |
| **A0A0E1VPK7** | Uncharacterized protein;Putative EsaC protein analog (Listeria type 3) | HMPREF0776_0647;esaC_2;BN1321 | 1,000E+00 | 5,670E+07 |
| **A0A0E1VQD7** | LytTr DNA-binding domain protein;Uncharacterized HTH-type transcriptional regulator SAOUHSC_02645;LytTR | HMPREF0776_0417;SAOUHSC_02645 | 1,000E+00 | 5,069E+08 |
| **A0A0E1XD35** | Serine-rich adhesin for platelets;Adhesin;Serine-threonine rich protein | sraP;CH51_14150;RU53_2717 | 1,000E+00 | 1,293E+08 |
| **A0A0E7DX57** | Peptidase%2C M16 family;Zinc protease;SA1122 protein | AFO87_05515;SAKOR_01207;SA1122 | 1,000E+00 | 3,266E+07 |
| **A0A0E7NTZ3** | Aminopeptidase;M42 glutamyl aminopeptidase | ysdC_1;ysdC_2;HMPREF0776_2384 | 1,000E+00 | 3,764E+09 |
| **A0A0E7R738** | 3-ketoacyl-CoA thiolase;Putative acyl-CoA acetyltransferase FadA;Acetyl-CoA acetyltransferase, putative | thlA;fadA | 1,000E+00 | 3,342E+08 |
| **A0A0E8HDY3** | Phage capsid protein;Major capsid protein;Phage major capsid protein | AFO87_05745;ST398NM01_2929;SAZ172_1975 | 1,000E+00 | 1,510E+08 |
| **A0A0G2LR37** | Uncharacterized protein;UPF0298 protein MW1004;UPF0298 protein SAZ172_1060 | CH51_05440;MW1004;SAZ172_1060 | 1,000E+00 | 4,208E+07 |
| **A0A0G2LV93** | Acetyltransferase;Acetyltransferase, GNAT family | CH51_13490;HMPREF0776_0579;AFO87_06175 | 1,000E+00 | 6,500E+07 |
| **A0A0G2LWI9** | Acetyltransferase;SA2490 protein;Uncharacterized protein | CH51_14365;nhoA;SA2490 | 1,000E+00 | 1,256E+08 |
| **A0A0H2WYG3** | Uncharacterized protein;Alanine racemase domain-containing protein | SACOL0929;AFO87_02380;BN1321_190049 | 1,000E+00 | 3,456E+07 |
| **A0A0H2WZ03** | Acetyl-CoA carboxylase, biotin carboxyl carrier protein, putative;Acetyl-CoA carboxylase;MW1557 protein | SACOL1662;accB_1;MW155 | 1,000E+00 | 1,958E+08 |
| **A0A0H3JMN2** | Uncharacterized protein;Addiction module toxin, Txe/YoeB family;Addiction module antitoxin RelB | SA2195;HMPREF0769_10737 | 1,000E+00 | 3,475E+08 |
| **A0A0H3JQ54** | Uncharacterized protein | SA1340 | 1,000E+00 | 1,505E+08 |
| **A0A0H3JWG6** | Uncharacterized protein;PadR family transcriptional regulator;Transcriptional regulator, PadR family | SA2484;AFO87_13960 | 1,000E+00 | 6,480E+07 |
| **A0A0H3JWX4** | Uncharacterized protein;Osmotically inducible protein C | MW1664;AFO87_07105;SAKOR_01663 | 1,000E+00 | 1,815E+08 |
| **A0A0H3K279** | MW0612 protein;Dihydroxyacetone kinase;PTS-dependent dihydroxyacetone kinase subunit I O | MW0612;dhaK | 1,000E+00 | 5,945E+07 |
| **A0A0H3K8X5** | Uncharacterized protein;Beta-lactamase | NWMN_1708;AFO87_00050 | 1,000E+00 | 2,575E+08 |
| **A0A0H3KIV1** | Transcriptional regulator MarR family protein;MarR family transcriptional regulator | NWMN_2411;yvnA | 1,000E+00 | 4,274E+07 |
| **A0A0M1TXD0** | Ribosomal-protein-serine N-acetyltransferase;Acetyltransferase, GNAT family;50S ribosomal protein L7 | RU53_317;HMPREF0776_1087 | 1,000E+00 | 1,964E+08 |
| **A0A0M1U3G4** | Phospholipase/carboxylesterase;Carboxylesterase | RU53_2581;SAZ172_2616;AFO87_06190 | 1,000E+00 | 2,495E+07 |
| **A0A0M1U3N2** | LPXTG cell wall surface anchor family protein;Uncharacterized protein;Adhesin | sasF;NWMN_2545;CH51_14110 | 1,000E+00 | 2,135E+07 |
| **A0A0S2J9G7** | Phnb;3-demethylubiquinone-9 3-methyltransferase;PhnB protein;Uncharacterized protein OS | ASU36_11230;AFO87_06745;SAZ172_2712 | 1,000E+00 | 2,173E+08 |
| **A0A0T7M9R1** | Uncharacterised protein;Uncharacterized protein;Unchara | ERS093009_02685;AFO87_09045;HMPREF0769_11886 | 1,000E+00 | 1,121E+08 |
| **A0A0U0TVG1** | NADH-dependent dehydrogenase;Uncharacterized protein;Oxidoreductase, NAD- | ycjS_2;NWMN_0155 | 1,000E+00 | 1,178E+08 |
| **A0A0U1MDM9** | Putative L-serine dehydratase, beta chain;L-serine dehydratase subunit beta;Serine dehydratase | BN1326_150037;sdhB_2;QU38_16485 | 1,000E+00 | 8,890E+07 |
| **A0A0U1MEZ0** | Polysaccharide biosynthesis family protein;Polysaccharide biosynthesis protein | BN1321_120025;HMPREF0769_12807 | 1,000E+00 | 4,694E+07 |
| **A0A0U1MGU0** | Acetyltransferase family protein;Uncharacterized protein;Acetyltransferase, GNAT family | BN1321_180034;MW0763;HMPREF0769_1 | 1,000E+00 | 1,361E+09 |
| **A0A0U1MIA1** | Putative acetyltransferase;Acetyltransferase, GNAT family;Acetyltransferase GNAT family | BN1326_50159;BN1321_150097;ERS093009_00152 | 1,000E+00 | 5,348E+08 |
| **A0A0U1MIZ1** | Ribulose-phosphate 3-epimerase | rpe | 1,000E+00 | 6,906E+07 |
| **A0A0U1MJG3** | Uncharacterized protein;Uncharacterized protein O | BN1321_240140;MW1174;CH51_06640 | 1,000E+00 | 1,375E+08 |
| **A0A0U1MKV3** | 50S ribosomal protein L7;Ribosomal protein L7Ae | rplGA;ACR58_02520 | 1,000E+00 | 4,040E+08 |
| **A0A0U1ML44** | Endonuclease III | nth | 1,000E+00 | 2,633E+08 |
| **A0A0U1MME7** | Sucrose operon repressor | scrR | 1,000E+00 | 8,436E+07 |
| **A0A0U1MN33** | Integrase;Phage integrase;Bact | int;Int-Tn;ACR61_03850 | 1,000E+00 | 6,138E+07 |
| **A0A0U1MNE3** | Aminotransferase class V;Soluble hydrogenase subunit;MW1665 protein OS | AUC48_08825;ERS092844_02144;ERS179246_02457 | 1,000E+00 | 1,160E+08 |
| **A0A0U1MTU7** | DNA gyrase subunit B | gyrB | 1,000E+00 | 1,684E+08 |
| **A0A0U1MVG7** | CHAP domain protein;CHAP domain-containing protein | AUC48_12985;HMPREF0769_10606;ACR58_13710 | 1,000E+00 | 5,339E+08 |
| **A0A0U1MZS8** | Glycerophosphoryl diester phosphodiesterase | glpQA | 1,000E+00 | 4,157E+07 |
| **A0A0V1TIE0** | MarR family transcriptional regulator;Uncharacterized protein | ACR61_14550;BN1321_410017 | 1,000E+00 | 9,296E+08 |
| **A6QGF9** | Ribosome-recycling factor | frr | 1,000E+00 | 8,170E+08 |
| **A6QHC8** | 30S ribosomal protein S20 | rpsT | 1,000E+00 | 6,763E+09 |
| **A6QJD1** | Urease accessory protein UreE | ureE | 1,000E+00 | 2,254E+08 |
| **D2J692** | Uncharacterised protein | ERS092844_02683 | 1,000E+00 | 1,266E+07 |
| **D2J695** | Hypothetical cytosolic protein;SAP031 protein;Uncharacterized protein | ERS092844_02686;SAP031;HMPREF0769_11640 | 1,000E+00 | 3,417E+09 |
| **D2J696** | Putative transcriptional regulator;DNA-binding helix-turn-helix protein | ERS092844_02687;CO98_2119 | 1,000E+00 | 3,250E+08 |
| **G5ELA4** | CsoR-like sulfur transferase-regulated gene B;Uncharacterized protein;Zn-dependent hydrolase | pksB_2;SAOUHSC_00036;QU38_ | 1,000E+00 | 5,695E+07 |
| **H6UH62** | Sugar isomerase;Uncharacterized protein;Transcriptional regulator, RpiR family | BN1321_80036;NWMN_0259;ST398NM01_0330 | 1,000E+00 | 3,550E+08 |
| **P0A0J3** | Superoxide dismutase [Mn] 1 | sodA | 1,000E+00 | 4,849E+09 |
| **P64147** | Dihydroneopterin aldolase;7,8-dihydroneopterin aldolase;7,8-dihydr | folB | 1,000E+00 | 7,942E+07 |
| **P65201** | Adenylate kinase | adk;ST398NM01_2282 | 1,000E+00 | 6,847E+08 |
| **P66689** | Ribonuclease P protein component | rnpA; | 1,000E+00 | 6,170E+07 |
| **P66715** | Probable DNA-directed RNA polymerase subunit delta;DNA-directed RNA polymerase subunit delta | rpoE | 1,000E+00 | 3,358E+09 |
| **P66830** | Superoxide dismutase [Mn/Fe] 2;Superoxide dismutase | sodM;sodA | 1,000E+00 | 2,611E+08 |
| **P66863** | SsrA-binding protein | smpB | 1,000E+00 | 4,891E+08 |
| **P67249** | UPF0122 protein SA1079;UPF0122 protein HMPREF0769_12542;UPF0122 protein ylxM | SA1079;HMPREF0769_12542;ylxM | 1,000E+00 | 9,041E+07 |
| **P67403** | Urease subunit alpha | ureC | 1,000E+00 | 3,370E+08 |
| **P67620** | Transcription factor FapR | fapR | 1,000E+00 | 2,117E+08 |

| | | | | |
|---|---|---|---|---|
| **P99068** | Nucleoside diphosphate kinase | ndk | 1,000E+00 | 6,077E+08 |
| **Q0WXP5** | Domain of uncharacterised function (DUF1413) | AL493_01525 | 1,000E+00 | 3,784E+07 |
| **Q2FF55** | Alanine racemase;Alanine racemase 1;Alanine racemase OS | alr;alr1 | 1,000E+00 | 1,195E+08 |
| **Q2FHG8** | Ribosome-binding factor A | rbfA | 1,000E+00 | 2,761E+08 |
| **Q2FHM8** | DNA-directed RNA polymerase subunit omega | rpoZ | 1,000E+00 | 8,024E+09 |
| **Q2FIL9** | Triosephosphate isomerase | tpiA | 1,000E+00 | 2,364E+09 |
| **Q2FJ70** | 3-hexulose-6-phosphate synthase;D-arabino-3-hexulose 6-phosphate formaldehyde lyase | SAUSA300_0555;SACOL0617 | 1,000E+00 | 1,152E+09 |
| **Q2FV40** | Uncharacterized protein;5-carboxyvanillic acid decarboxylase | SAOUHSC_02899;ERS179246_01666;MW250 | 1,000E+00 | 3,169E+08 |
| **Q2FV63** | Copper chaperone CopZ;Copper chaperone copZ | copZ;QU38_16345 | 1,000E+00 | 3,074E+08 |
| **Q2FVJ6** | Adenosylmethionine-8-amino-7-oxononanoate aminotransferase;Adenosylmethionine-8 | bioA | 1,000E+00 | 5,653E+06 |
| **Q2FWN3** | 10 kDa chaperonin | groS;groES | 1,000E+00 | 2,531E+08 |
| **Q2FXW2** | UPF0473 protein SAOUHSC_01719;UPF0473 protein V070_00674 | SAOUHSC_01719;V070_00674 | 1,000E+00 | 4,116E+08 |
| **Q2FXW7** | Transcription elongation factor GreA | greA | 1,000E+00 | 9,739E+08 |
| **Q2FY47** | Exodeoxyribonuclease 7 small subunit | xseB | 1,000E+00 | 1,569E+08 |
| **Q2FYN6** | Uncharacterized hydrolase SAOUHSC_01399;Putative zinc peptidase;Amidohydrolase | SAOUHSC_01399;RU53_1426 | 1,000E+00 | 5,145E+07 |
| **Q2FZD9** | Phenylalanine--tRNA ligase alpha subunit | pheS | 1,000E+00 | 1,239E+09 |
| **Q2G0I3** | Uncharacterized protein;DNA-binding protein;Transcriptional regulator, BadM/Rrf2 family;DN | SAOUHSC_00582;ywnA;BN1321_150036 | 1,000E+00 | 2,031E+08 |
| **Q2G1R3** | tRNA-dihydrouridine synthase;Probable tRNA-dihydrouridine synthase | SAOUHSC_00039;dus | 1,000E+00 | 1,795E+08 |
| **Q2G2J7** | Uncharacterized protein | SAOUHSC_01414 | 1,000E+00 | 3,320E+07 |
| **Q2G2M4** | Mini-ribonuclease 3 | mrnC | 1,000E+00 | 7,981E+07 |
| **Q2TF02** | CadX;Cadmium efflux system accessory protein;ArsR family transcriptional regulator;ArsR family transcriptional regulator | cadX;cadC;CH51_14500 | 1,000E+00 | 5,565E+08 |
| **Q5HD29** | D-lactate dehydrogenase | ldhD | 1,000E+00 | 1,215E+08 |
| **Q5HDM3** | Formimidoylglutamase | hutG | 1,000E+00 | 1,726E+08 |
| **Q5HDV8** | 50S ribosomal protein L3 | rplC | 1,000E+00 | 1,887E+08 |
| **Q5HE19** | Zinc-type alcohol dehydrogenase-like protein SACOL2177;Uncharacterized protein | SACOL2177;SAKOR_02154 | 1,000E+00 | 5,320E+07 |
| **Q5HEM1** | Aspartyl/glutamyl-tRNA(Asn/Gln) amidotransferase subunit C | gatC | 1,000E+00 | 9,924E+08 |
| **Q5HF65** | Alanine dehydrogenase 2;Alanine dehydrogenase | ald2;ald;SAKOR_01650 | 1,000E+00 | 3,841E+08 |
| **Q5HFH9** | Protein GrpE | grpE | 1,000E+00 | 5,202E+08 |
| **Q5HG56** | Probable tautomerase SACOL1399;Tautomerase | SACOL1399;graC | 1,000E+00 | 1,406E+09 |
| **Q5HGQ3** | Transcriptional regulator MraZ | mraZ | 1,000E+00 | 4,542E+09 |
| **Q5HGZ3** | Peptide deformylase | def | 1,000E+00 | 1,158E+09 |
| **Q5HH02** | Phosphocarrier protein HPr | ptsH;ST398NM01_1080 | 1,000E+00 | 2,936E+08 |
| **Q5HH40** | 2-succinyl-5-enolpyruvyl-6-hydroxy-3-cyclohexene-1-carboxylate synthase | menD | 1,000E+00 | 1,585E+08 |
| **Q5HH86** | Adapter protein MecA;Adapter protein MecA | mecA | 1,000E+00 | 1,128E+08 |
| **Q5HJ88** | Protein EsaB | esaB;SAOUHSC_00260 | 1,000E+00 | 5,012E+08 |
| **Q7A0X3** | Cold shock protein CspA | cspA;V070_00472 | 1,000E+00 | 5,754E+09 |
| **Q7A1Y8** | Heme oxygenase (staphylobilin-producing) 2;Heme oxygenase (staphylobilin-producing) | isdI;ACH32_08100 | 1,000E+00 | 2,581E+08 |
| **Q7A2N3** | Endoribonuclease MazF;mRNA interferase;mRNA interfer | mazF;HMPREF0769_11093;ndoA | 1,000E+00 | 1,985E+09 |
| **Q7DI47** | IraB;Uncharacterized protein | iraB;BN1326_140282;QU38_16720;BN1321_41005 | 1,000E+00 | 4,173E+08 |
| **Q7DJC4** | Glycerophophory diester phosphodiesterase homolog | ugpQ | 1,000E+00 | 5,747E+07 |
| **Q8NUR1** | Putative acetyltransferase MW2476;Maltose O-acetyltransferase;Bacterial transferase hexapeptide repeat protein | MW2476;RU53_2619 | 1,000E+00 | 8,106E+07 |
| **Q8NV90** | Urease subunit beta | ureB | 1,000E+00 | 8,297E+07 |
| **Q8NVT4** | Uncharacterized RNA methyltransferase MW1838;Uncharacterized RNA methyltransferase SA1713 | MW1838;SA1713 | 1,000E+00 | 6,182E+08 |
| **Q8NW56** | UPF0173 metal-dependent hydrolase MW1650;UPF0173 metal-dependent hydrolase BN1321_260339 | MW1650;BN1321_260339 | 1,000E+00 | 8,475E+08 |
| **Q8NX50** | Fibrinogen-binding protein | fib | 1,000E+00 | 3,371E+08 |
| **Q93CC9** | Cro/CI family transcriptional regulator | stl | 1,000E+00 | 6,055E+08 |
| **Q93SM4** | Long-chain fatty acid--CoA ligase;Long-chain-fatty-acid--CoA ligase;SA0226 protein | fadE;fadD;SA0226 | 1,000E+00 | 2,594E+07 |
| **Q99S93** | UPF0457 protein SA1975.1;Uncharacterized protein | SA1975.1;RU53_2241 | 1,000E+00 | 4,199E+09 |
| **Q99T00** | Low molecular weight protein-tyrosine-phosphatase PtpA | ptpA | 1,000E+00 | 5,091E+07 |
| **Q99T18** | Peroxide-responsive repressor PerR;Transcriptional regulator (Fur family) | perR | 1,000E+00 | 1,118E+08 |
| **Q99T33** | UPF0342 protein SAV1845;UPF0342 protein HMPREF0769_11833;UPF0342 protein yheA | SAV1845;HMPREF0769_11833;yhe | 1,000E+00 | 3,767E+09 |
| **Q99VH8** | Organic hydroperoxide resistance protein-like;Peroxiredoxin, Ohr subfamily;Peroxiredoxin | SAV0828;HMPREF0776_1818 | 1,000E+00 | 6,588E+07 |
| **Q99WJ6** | NADPH-dependent oxidoreductase | nfrA | 1,000E+00 | 4,713E+07 |
| **Q9EUT0** | Chromosome replication initiation protein dnaD;DnaD domain protein | dnaD;HMPREF0776_2477 | 1,000E+00 | 1,276E+08 |
| **Q9EZ11** | 4-hydroxy-tetrahydrodipicolinate reductase | dapB | 1,000E+00 | 4,575E+07 |
| **Q9F4G3** | HTH-type transcriptional regulator TcaR;Uncharacterized protein | tcaR;tcaR_1;SAOU | 1,000E+00 | 3,158E+08 |
| **T1Y6J8** | Histidine ammonia-lyase | hutH | 1,000E+00 | 1,762E+08 |
| **T1Y8T9** | PBS lyase HEAT-like repeat protein;Scaffolding protein | SAKOR_01367;cvfC_1;ACH32_14140 | 1,000E+00 | 3,696E+08 |
| **T1YCD3** | Transcriptional regulator, TetR family protein;HTH-type transcriptional regulator MW2498 | SAKOR_02572;MW2498 | 1,000E+00 | 5,402E+07 |
| **U5NYY0** | Lysophospholipase | ytpA_1 | 1,000E+00 | 6,446E+08 |
| **W8TPW5** | Uncharacterised protein;Uncharacterized protein | AFO87_11930;QU38_14935;BN1326_30033 | 1,000E+00 | 9,778E+07 |
| **W8TRW2** | GntR family transcriptional regulator;Transcriptional regulator of pyridoxine metabolism | gabR;gabR_1;BN1321_130001 | 1,000E+00 | 6,660E+08 |
| **W8TV80** | Cystine ABC transporter (ATP-binding protein);ArtP;GlnQ protein | glnQ;ST398NM01_1923 | 1,000E+00 | 9,670E+08 |
| **W8TVN3** | DNA mismatch repair protein MutT;Uncharacterized protein | AFO87_12065;QU38_08565;BN1321_260308 | 1,000E+00 | 1,876E+08 |
| **W8U0B2** | Glycerophosphodiester phosphodiesterase;Glycerophosphoryl diester phosphodiesterase | glpQ | 1,000E+00 | 7,238E+07 |
| **W8U6A7** | Uncharacterised protein | AFO87_10460 | 1,000E+00 | 9,774E+08 |
| **W8U862** | Molecular chaperone GroEL;Uncharacterized protein | AFO87_12710;SA2238;ERS179246_00936 | 1,000E+00 | 3,036E+09 |
| **W8URR1** | N5-carboxyaminoimidazole ribonucleotide mutase | purE | 1,000E+00 | 9,677E+07 |
| **X5DWV7** | Glycerophosphodiester phosphodiesterase;Glycerophosphodiester phosphodiesterase family protein;MW1663 protein | ugpQ_2;HMPREF0776_2752 | 1,000E+00 | 8,465E+08 |
| **X5E039** | Riboflavin synthase alpha chain;Riboflavin synthase, alpha subunit | ribB;r | 1,000E+00 | 2,126E+08 |
| **X5E1D9** | Formate dehydrogenase;4-phosphoerythronate dehydrogenase | fdh;RU53_160;pdxB | 1,000E+00 | 2,460E+08 |
| **X5E3N2** | DNA-binding protein;Putative dNA-binding protein;Uncharacterized protein | AFO87_08250;BN1321_240015;AL078_05710 | 1,000E+00 | 5,534E+08 |
| **X5EKM1** | Cro/CI family transcriptional regulator;Uncharacterized HTH-type transcriptional regulator YgzD;Putative transcriptional regulator | AFO87_05025;ygzD | 1,000E+00 | 5,270E+08 |

## Population II. DSM enriched

| **Uniprot-ID** | **Protein Name** | **Gene Name** | **LFQ Intensity DRM** | **LFQ Intensity** |
|---|---|---|---|---|
| **A0A033UTU4** | Beta-lactam-inducible penicillin-binding protein | V070_01423 | 9,268E+08 | 1,817E+09 |
| **A0A033UZD3** | Chaperone protein DnaK | dnaK | 7,229E+07 | 1,909E+08 |
| **A0A033V2K7** | Threonine--tRNA ligase | thrS | 3,043E+08 | 4,514E+08 |
| **A0A062W679** | CI-like repressor%2C phage associated | lexA_2 | 9,073E+08 | 8,369E+09 |
| **A0A069FM85** | Export membrane protein | CO98_1599 | 5,442E+08 | 1,239E+09 |
| **A0A069FR69** | Phosphoglycerate kinase | pgk | 1,653E+08 | 2,359E+08 |
| **A0A069FSS7** | DNA-directed RNA polymerase subunit beta | rpoB | 2,594E+08 | 1,315E+09 |
| **A0A069FXY2** | Ferritin | CO98_2184;V070_02700 | 2,733E+08 | 1,154E+09 |
| **A0A069G712** | Pyruvate dehydrogenase E1 component subunit beta | pdhB | 2,807E+09 | 1,851E+10 |
| **A0A069G7V5** | Pyruvate kinase | pyk | 2,381E+09 | 3,008E+09 |
| **A0A0B6XLW5** | 3-dehydroquinate dehydratase | aroC_1;aroD | 4,240E+07 | 1,039E+08 |
| **A0A0B6XMY5** | Alanine racemase;Pyridoxal-5-phosphate family protein | yggS;ACR58_02940;SAKOR_01114 | 5,415E+08 | 1,439E+09 |
| **A0A0B6XQ62** | Aminopeptidase;Thermophilic metalloprotease (M29);Aminopeptidase T;Aminopeptidase | pepS;HMPREF0776_0019;CH51_10245 | 4,693E+08 | 8,646E+08 |
| **A0A0D1EZR2** | Thioredoxin | ACH32_01585 | 4,293E+07 | 3,628E+08 |
| **A0A0D1F4Q8** | Cell shape-determining protein MreC | ACH32_01090;SAKOR_01597 | 2,182E+09 | 5,106E+09 |
| **A0A0D1FER1** | Uncharacterized protein;Putative cytosolic protein | ACR58_11475;NWMN_0576;ST398 | 7,092E+08 | 1,998E+09 |
| **A0A0D1FFQ5** | Competence protein ComK;ComK family protein;Competence transcription factor | ACH32_11980;BN1326_60138;ST398NM01_1023 | 3,357E+09 | 1,080E+10 |
| **A0A0D1FGD6** | Uncharacterized protein | ACH32_13395;BN1326_60402 | 5,868E+08 | 3,019E+09 |
| **A0A0D1FJV3** | Glucose-6-phosphate 1-dehydrogenase | zwf | 5,720E+08 | 6,702E+08 |
| **A0A0D1FLC7** | Amino acid ABC transporter ATP-binding protein;MW2334 protein;Amino acid ABC transporter, ATP-binding protein | tcyC;MW2334 | 5,201E+09 | 1,947E+10 |
| **A0A0D1FY59** | MarR family transcriptional regulator;MW2549 protein;Transcriptional regulator, MarR family | ACR58_09835;MW2549 | 4,569E+07 | 2,293E+09 |
| **A0A0D1G023** | Uncharacterized protein | ACH32_05000;HMPREF0776_0373 | 1,615E+08 | 2,488E+08 |
| **A0A0D1GE69** | Thioredoxin;Putative thiol-disulfide oxidoreductase with thioredoxin domain;Uncharacterized protein | ACR58_04520;yusE;HMP | 3,383E+07 | 6,618E+07 |
| **A0A0D1GNT7** | Bacillithiol system protein YtxJ;Putative cytosolic protein;YtxJ like protein | ACH32_11050;ST398NM01_0817;ERS093009_002 | 6,759E+07 | 1,581E+08 |
| **A0A0D1GX94** | 4-hydroxybenzoyl-CoA thioesterase;Thioesterase;4-hydroxybenzoyl-CoA thioesterase family active site protein | ACH32_13730;ST398NM01_1353 | 1,188E+08 | 4,092E+08 |
| **A0A0D1GYV5** | ADP-dependent (S)-NAD(P)H-hydrate dehydratase | nnrD | 1,367E+08 | 6,058E+08 |
| **A0A0D1H1G7** | Cell division protein FtsA | ftsA | 8,290E+09 | 1,240E+10 |
| **A0A0D1H3M2** | Acetyltransferase;Acetyltransferase, GNAT family | ACR58_14475;SAKOR_01356;HMPREF0769_ | 1,248E+07 | 3,332E+08 |
| **A0A0D1H4D9** | Putative universal stress protein;Universal stress family protein | ACH32_01360;HMPREF0776_2738 | 5,016E+09 | 6,537E+10 |
| **A0A0D1H5H3** | Antibiotic ABC transporter ATP-binding protein;ABC transporter, ATP-binding protein;SA1745 protein | ACH32_02755;HMPREF0776_0093 | 1,487E+10 | 6,371E+10 |
| **A0A0D1H739** | Peroxiredoxin Q/BCP;Bacterioferritin comigratory protein;Antioxidant, AhpC/TSA family | bcp;NWMN_1755;HMPR | 1,934E+08 | 3,147E+09 |
| **A0A0D1HCR3** | RNA-binding protein S1;Putative RNA degradation protein polyribonucleotide nucleotidyltransferase or phosphorylase | ACH32_09750;yugI_2 | 4,896E+07 | 4,907E+09 |
| **A0A0D1HV16** | DNA-binding protein;Putative RNA binding protein putative new fold | ACH32_13295;ylxR | 1,949E+08 | 1,584E+09 |
| **A0A0D1HYM2** | DNA repair exonuclease;Calcineurin-like phosphoesterase family protein;Phosphoesterase | ACH32_02130;BN1321_290014;ST398 | 5,892E+09 | 1,316E+10 |
| **A0A0D1I3Y7** | Uncharacterized protein;Uncharacterised protein | ACH32_09130;ERS179246_01599 | 2,224E+08 | 5,869E+08 |
| **A0A0D1I8F1** | Thioredoxin;Uncharacterized protein;Uncharac | ACH32_02745;ERS179246_01904;HMPREF0776_0091 | 1,217E+10 | 2,975E+10 |
| **A0A0D1IKI9** | Cell-cycle regulation protein HIT;Adenosine 5-monophosphoramidase | ACH32_02100;SAKOR_01791 | 3,652E+08 | 1,380E+09 |

| | | | | |
|---|---|---|---|---|
| **A0A0D1IKS3** | Ktr system potassium uptake protein C;Trk system potassium uptake protein trkA;Potassium uptake protein | ktrC;trkA | 3,231E+09 | 5,700E+09 |
| **A0A0D1IP51** | Aldo/keto reductase family oxidoreductase;Oxidoreductase of aldo/keto reductase family%2C subgroup 1;SA0658 protein | ACR58_05120;ERS179246_00200 | 3,112E+08 | 3,984E+08 |
| **A0A0D1IR83** | RpiR family HTH-type transcriptional regulator;RpiR family regulatory protein | ACR58_07940;BN1326_140111 | 8,561E+08 | 1,985E+09 |
| **A0A0D1J251** | HTH-type transcriptional regulator;Uncharacterized protein | ACH32_00975;SAOUHSC_01732 | 6,838E+08 | 3,665E+09 |
| **A0A0D1J5Y0** | NifU domain-containing protein;NifU domain protein;NifU-like protein | ACH32_11510;SACOL0939;ST398NM01_0919 | 1,862E+08 | 2,336E+08 |
| **A0A0D1JF17** | Nitrogen regulatory-like protein;Protein from nitrogen regulatory protein P-II (GLNB) family | ACH32_09610;yaaQ | 8,069E+07 | 1,814E+09 |
| **A0A0D1JL07** | 2-oxoglutarate dehydrogenase | QU38_09355 | 1,190E+09 | 1,322E+09 |
| **A0A0D1JSZ8** | DNA mismatch repair protein MutT;Mutator mutT protein (7,8-dihydro-8-oxoguanine-triphosphatase);Mutator mutT protein | ACR58_08900;SAZ172_2586 | 4,262E+07 | 1,147E+08 |
| **A0A0D1JTT5** | 2,5-diketo-D-gluconic acid reductase;Morphine 6-dehydrogenase;Oxidoreductase, aldo/keto reductase family protein | ACR58_07330;ST398NM01_2260 | 1,346E+09 | 2,500E+09 |
| **A0A0D1JY50** | HAD family hydrolase;Hydrolase (HAD superfamily);HAD hydrolase, IA, variant 1 family protein | ACR58_11515;ST398NM01_0692 | 6,669E+08 | 9,086E+08 |
| **A0A0D3Q434** | Putative cytosolic protein;Uncharacterized protein | AFO87_05325;ERS195423_01542;ST398NM01_0310 | 5,820E+08 | 6,749E+08 |
| **A0A0D3Q6N2** | Signal recognition particle receptor FtsY | ftsY | 3,375E+09 | 4,113E+09 |
| **A0A0D3QA93** | Zinc ABC transporter substrate-binding protein;Uncharacterized protein;MW2328 protein | zinT;BN1321_400054;MW2328 | 1,232E+09 | 2,216E+09 |
| **A0A0D3QAJ7** | Uncharacterised protein;Uncharacterized protein;Uncharacterized protein OS | AFO87_00970;AUC48_11675;MW2214 | 5,613E+08 | 8,070E+08 |
| **A0A0D6FS79** | Flavohemoprotein (Hemoglobin-like protein) (Flavohemoglobin) (Nitric oxide dioxygenase);Oxidoreductase, FAD-binding protein | hmp;HMPREF0776_0968 | 9,589E+08 | 1,400E+09 |
| **A0A0D6G869** | Glycosyl transferase family 1;Poly (Glycerol-phosphate) alpha-glucosyltransferase;SA0522 protein | tagE_2;RU53_552 | 2,247E+08 | 2,426E+09 |
| **A0A0D6G8D7** | HAD family hydrolase;Cof-like hydrolase;Uncharacterized protein | ywpJ_1;BN1321_130047;MW0514 | 2,302E+09 | 2,390E+09 |
| **A0A0D6GAF3** | Restriction endonuclease subunit M;Type I restriction-modification system, M subunit | hsdM;SAOUHSC_00397 | 7,807E+08 | 5,832E+09 |
| **A0A0D6GB57** | Restriction endonuclease subunit S;Sau1hsdS1;Type I restriction modification DNA specificity domain protein | hsdS_1;sau1hsdS1;HMPREF076 | 1,399E+09 | 9,651E+08 |
| **A0A0D6GDE2** | ABC transporter ATP-binding protein;ABC transporter family protein | AFO87_05015;BN1321_80070;SAKOR_ | 9,480E+08 | 4,607E+09 |
| **A0A0D6GH02** | Oligoendopeptidase F | pepF1_1;ACH32_11840;ST398NM01_0996 | 5,894E+08 | 5,183E+09 |
| **A0A0D6GHM0** | SepS16B protein;Uncharacterized protein | sepS16B;QU38_14805;s | 3,191E+08 | 1,493E+09 |
| **A0A0D6GJ33** | Pyruvate decarboxylase;MW0162 protein;Thiamine pyrophosphate enzyme, N-terminal TPP binding domain protein | ipdC;MW0162 | 5,662E+07 | 3,584E+08 |
| **A0A0D6H7B9** | Alpha-acetolactate decarboxylase | aldC_2;aldB;budA1 | 2,187E+08 | 3,001E+08 |
| **A0A0D6HBX1** | Radical SAM/CxCxxxxC motif protein YfkAB;Thioredoxin-like oxidoreductase;Uncharacterized protein | AFO87_08785;SAKOR_01833 | 3,121E+08 | 2,092E+09 |
| **A0A0D6HDZ6** | Aldo/keto reductase family protein;Oxidoreductase of aldo/keto reductase family, subgroup 1;2,5-diketo-D-gluconic acid reductase | yvgN;SAZ172_1799 | 9,027E+07 | 2,364E+08 |
| **A0A0D6HHA6** | Malate dehydrogenase;NADP-dependent malic enzyme | maeB;SAZ172_1715;ACH32_01340 | 5,685E+08 | 1,081E+09 |
| **A0A0D6HHM0** | Formamidopyrimidine-DNA glycosylase | mutM;fpg | 1,050E+08 | 4,809E+08 |
| **A0A0D6HHM2** | Free methionine-(R)-sulfoxide reductase;GAF domain protein;Uncharacterized protein | msrC;MW1661 | 1,162E+08 | 2,679E+08 |
| **A0A0D6HHY4** | Oxidoreductase;NAD dependent epimerase/dehydratase family protein;Oxidoreductase ylbE | ylbE;HMPREF0776_1400 | 3,194E+08 | 1,761E+09 |
| **A0A0D6HJ55** | Fic family protein;Adenosine monophosphate-protein transferase SoFic | AFO87_10960;RU53_1700 | 3,174E+08 | 4,931E+08 |
| **A0A0D6HL17** | Tripeptidase T;Peptidase T, putative;MW1465 protein;Peptidase T-like protein | pepT_1;SAOUHSC_01606;MW1465 | 4,052E+08 | 4,161E+08 |
| **A0A0D6HLL6** | ATP-dependent DNA helicase;DEAD/DEAH box helicase | recQ_2;BN1321_260120;recQ | 3,822E+07 | 4,183E+07 |
| **A0A0D6HQE4** | 2-oxoacid ferredoxin oxidoreductase subunit alpha;2-oxoacid:acceptor oxidoreductase, alpha subunit | porA;HMPREF0769_12489 | 8,800E+07 | 4,048E+08 |
| **A0A0D6HTX7** | LSU m5C1962 methyltransferase RlmI;Putative SAM-dependent methyltransferases;Uncharacterized protein | rlmI;RU53_1078 | 2,138E+09 | 1,053E+10 |
| **A0A0D6HWT3** | NADH-dependent flavin oxidoreductase | AFO87_02530;CH51_04615;QU38 | 5,955E+08 | 1,597E+09 |
| **A0A0D6HWV3** | Kinase;Kinase-associated protein B | kapB;ST398NM01_0936;ACH32_11595 | 1,238E+08 | 5,235E+08 |
| **A0A0D6HYA6** | Ribonuclease R | rnr;CH51_04080 | 5,064E+08 | 9,280E+09 |
| **A0A0E0VLS3** | Branched-chain-amino-acid aminotransferase | ST398NM01_0629;ilvE | 2,498E+09 | 4,510E+09 |
| **A0A0E0VN64** | DNA Polymerase X family;DNA polymerase;PHP domain protein | ST398NM01_1139;polX;HMPREF0769_12639 | 7,422E+07 | 3,488E+08 |
| **A0A0E0VP52** | Phosphoribosylformylglycinamidine synthase subunit PurS | purS | 3,481E+08 | 9,742E+08 |
| **A0A0E0VSL8** | ParB;Chromosome partitioning protein ParB;DNA-binding protein Spo0J-like | ST398NM01_2754;ACH32_07010;noc | 4,694E+08 | 4,515E+09 |
| **A0A0E0VSM0** | ATP synthase subunit beta | atpD | 7,269E+10 | 1,045E+11 |
| **A0A0E1AE12** | Putative hydrolase;3-oxoadipate enol-lactonase | SAZ172_0626;dehH1 | 8,998E+08 | 6,612E+09 |
| **A0A0E1AFA2** | 2,3-cyclic-nucleotide 2-phosphodiesterase;2%2C3-cyclic-nucleotide 2-phosphodiesterase;2, 3-cyclic nucleotide 2-phosphodiesterase | SAZ172_0965;yfkN_3 | 1,042E+08 | 1,273E+08 |
| **A0A0E1AH29** | Ribosomal RNA small subunit methyltransferase E;Ribosomal RNA small subunit methyltransferase E | SAZ172_1590;rsmE | 1,465E+08 | 3,893E+08 |
| **A0A0E1AJ76** | Putative esterase;Uncharacterized protein;MW2271 protein | SAZ172_2453;SAOUHSC_02627;MW2271 | 1,052E+09 | 3,362E+09 |
| **A0A0E1AKN8** | Putative oxidoreductase YncB;Alcohol dehydrogenase;Oxidoreductase, zinc-binding dehydrogenase family protein | SAZ172_2291;curA | 1,305E+09 | 1,694E+09 |
| **A0A0E1VI94** | Ribosomal RNA small subunit methyltransferase H | mraW;rsmH | 2,154E+09 | 2,999E+09 |
| **A0A0E1VIX6** | Arginine repressor (Fragment);Arginine repressor | argR;ahrC | 1,068E+09 | 2,188E+09 |
| **A0A0E1VJA5** | Ornithine aminotransferase;Ornithine aminotransferase 2 | rocD;rocD2;roc | 4,479E+08 | 4,491E+09 |
| **A0A0E1VLD0** | Thermonuclease | nuc | 4,119E+07 | 3,985E+08 |
| **A0A0E1VLD1** | S1 RNA binding domain protein;General stress protein | HMPREF0776_1944;yugI_1;QU38_11390 | 1,538E+08 | 3,114E+08 |
| **A0A0E1VMI0** | Fructose-1,6-bisphosphate aldolase, class II;Fructose-bisphosphate aldolase | fba | 1,258E+09 | 6,252E+09 |
| **A0A0E1VMK6** | Cof-like hydrolase;HAD family hydrolase;Putative cytosolic protein | HMPREF0776_1393;ywpJ_2;SAKOR_02119 | 9,512E+07 | 1,851E+08 |
| **A0A0E1VMN2** | Sensor protein SrrB;Osmosensitive K+ channel histidine kinase SrrB | srrB | 1,477E+09 | 7,115E+09 |
| **A0A0E1VR62** | Pseudouridine synthase | HMPREF0776_2518;rluB | 9,786E+08 | 7,520E+09 |
| **A0A0E1VS63** | 50S ribosomal protein L19 (Fragment);50S ribosomal protein L19 | rplS | 7,019E+09 | 1,287E+10 |
| **A0A0E1VSM9** | Coenzyme A disulfide reductase | cdr | 6,207E+08 | 2,330E+09 |
| **A0A0E1VYN8** | Uncharacterized protein;Leucine carboxyl methyltransferase | HMPREF0776_0175;AL493_12285;AF | 5,871E+07 | 8,729E+07 |
| **A0A0E1VZ65** | Uncharacterized protein;Putative membrane associated protein;Membrane associated protein | HMPREF0776_0022;ACH32_02485 | 4,072E+08 | 3,057E+09 |
| **A0A0E1X703** | Uncharacterized protein;Uncharacterised protein | HMPREF0769_12194;AFO87_01630;MW1439 | 3,558E+08 | 2,065E+09 |
| **A0A0E1X7D7** | Diaminopimelate decarboxylase | lysA | 2,321E+08 | 2,919E+08 |
| **A0A0E1X7E5** | Dehydrogenase E1 component;Branched-chain alpha-keto acid dehydrogenase E1 | HMPREF0769_12161;bfmBAA | 1,164E+09 | 1,407E+09 |
| **A0A0E1X7T8** | Uncharacterized protein | HMPREF0769_11617;HMPREF0776_1686 | 9,216E+07 | 5,167E+08 |
| **A0A0E1XGT1** | DivIVA domain protein;Cell division initiation protein;Cell division initiation protein DivIVA | HMPREF0769_12587;divIVA;Q | 5,146E+08 | 1,163E+09 |
| **A0A0E7MG06** | Pyridine nucleotide-disulfide oxidoreductase;Uncharacterized protein | AFO87_10185;SAZ172_1785 | 2,988E+07 | 5,742E+07 |
| **A0A0E7NNB9** | Nitroreductase;Nitroreductase family protein;Uncharacterized protein | AFO87_10670;SAKOR_00809;MW0783 | 2,479E+08 | 2,106E+09 |
| **A0A0E7R271** | Bacillithiol biosynthesis deacetylase BshB2 | mshB;bshB2 | 1,794E+08 | 3,273E+08 |
| **A0A0E7RYR7** | DNA-binding protein;Uncharacterized protein | AFO87_07720;SAOUHSC_02393 | 1,738E+09 | 2,600E+09 |
| **A0A0E8G694** | Pseudouridine synthase | rluD | 2,525E+09 | 4,322E+09 |
| **A0A0E8G771** | Radical activating enzyme protein;Uncharacterized protein | AFO87_04435;BN1321_230004;HMPREF0776_2074 | 2,213E+09 | 2,425E+09 |
| **A0A0E8G8A7** | Pyruvate carboxylase | cfiB_1;SAZ172_1053 | 1,313E+09 | 1,189E+10 |
| **A0A0E8GAU0** | UvrABC system protein A | uvrA | 2,665E+09 | 2,825E+09 |
| **A0A0E8GBF4** | Aldo/keto reductase family protein;Uncharacterized protein;Aldo/keto reductase;Oxidoreductase | ydhF;ST398NM01_0766 | 1,473E+08 | 8,617E+08 |
| **A0A0E8GCM1** | Fibronectin-binding protein;Uncharacterized protein;SA1051 protein | AFO87_09195;NWMN_1119;SA1051 | 6,262E+08 | 1,343E+09 |
| **A0A0E8GDK3** | Dihydrolipoyl dehydrogenase;Dihydrolipoyl dehydrogen | pdhD;CH51_05315 | 2,207E+10 | 4,831E+11 |
| **A0A0E8GFB0** | Putative cytosolic protein;Uncharacterized protein | ywqG | 3,191E+08 | 3,207E+08 |
| **A0A0E8GNP9** | Transaldolase | tal;ACH32_01770;BN1326_90019;S | 4,008E+08 | 1,871E+09 |
| **A0A0E8GPH7** | Smooth muscle caldesmon;Uncharacterized protein;Sm | AFO87_07005;NWMN_1631;SAUSA300_1684 | 2,412E+10 | 2,612E+10 |
| **A0A0E8GPJ7** | NAD(FAD)-utilizing dehydrogenase;Uncharacterized protein;NAD | AL493_10735;ERS179246_01525;SA1576 | 2,994E+08 | 8,344E+08 |
| **A0A0E8HBP5** | ABC transporter ATP-binding protein;ABC-type nitrate/sulfonate/bicarbonate transporter, TauB | ssuB_1;tauB | 1,909E+09 | 5,287E+09 |
| **A0A0E8HLR7** | Integrase;Uncharacterized protein | AL493_04605;SAZ172_2691;ER12_002930;CH51_ | 2,731E+08 | 4,084E+09 |
| **A0A0E8IKQ5** | PhiSLT ORF78-like protein;Conserved hypothetical phage protein;Uncharacterized protein | AFO87_05895;SAUSA300_1965;CH51_08075 | 2,390E+08 | 3,265E+09 |
| **A0A0E8J2Q8** | Single-stranded DNA-binding protein | ssb_2;MW2021; | 3,895E+08 | 2,369E+09 |
| **A0A0E8JDK7** | Methionine--tRNA ligase;Methionine--tR | metS;metG | 9,735E+08 | 3,150E+09 |
| **A0A0G2LQP6** | Nitrogen fixation protein NifU;Iscu;Iron-sulfur cluster assembly scaffold protein NifU | CH51_04420;ST398NM01_0900;nifU | 4,483E+07 | 1,014E+08 |
| **A0A0G2LVP9** | RpiR family transcriptional regulator | CH51_12515;ybbH_2;QU38_0 | 6,269E+08 | 1,051E+09 |
| **A0A0H2AL62** | DNA topoisomerase 4 subunit A | parC | 6,516E+08 | 9,686E+09 |
| **A0A0H2B964** | Uncharacterized conserved protein;Uncharacterized protein | AFO87_00900;SAZ172_2408;HMPREF076 | 8,861E+08 | 2,767E+09 |
| **A0A0H2WYK8** | Uncharacterized protein;Phi77 ORF017-like protein;Toxin-antitoxin system, toxin component, MazF family | SACOL0319;AFO87_03400;BN1326_100140 | 4,354E+08 | 7,180E+08 |
| **A0A0H2XHQ4** | Alkyl hydroperoxide reductase, subunit F;Alkyl hydroperoxide reductase subunit F;Alkyl hydroperoxide reductase, F subunit | ahpF | 5,447E+09 | 8,497E+09 |
| **A0A0H2XI89** | Ribosomal RNA small subunit methyltransferase B | sun;rsmB | 1,250E+09 | 2,005E+09 |
| **A0A0H3JK71** | SA0653 protein;DeoR C terminal sensor domain protein;Transcriptional regulator, DeoR family | SA0653;ACH32_10815 | 4,555E+07 | 2,595E+08 |
| **A0A0H3JN52** | SA1675 protein;Amino acid ABC transporter periplasmic protein;Extracellular glutamine-binding protein | SA1675;artQ | 6,472E+08 | 1,641E+09 |
| **A0A0H3JNF9** | 2-dehydropantoate 2-reductase | SA2232;panE_1;QU38_08000 | 5,054E+08 | 6,689E+08 |
| **A0A0H3JP54** | Tagatose-6-phosphate kinase;Tagatose-6-phosphate kinase O | fruB | 9,921E+07 | 1,106E+08 |
| **A0A0H3JPQ1** | SA1000 protein;Extracellular fibrinogen binding protein | SA1000;ecb | 1,215E+09 | 2,463E+09 |
| **A0A0H3JSM8** | SA0224 protein;3-hydroxyacyl-CoA dehydrogenase, NAD binding domain protein;3-hydroxyacyl-CoA dehydrogenase | SA0224;HMPREF0776_0960 | 4,486E+08 | 2,815E+09 |
| **A0A0H3JTK4** | Uncharacterized protein;Disulfide oxidoreductase | SA0798;ACR58_04270;NWMN_0808 | 1,007E+08 | 1,793E+08 |
| **A0A0H3JTX5** | Aldehyde-alcohol dehydrogenase | adhE;ACR58_06650 | 2,220E+07 | 3,526E+07 |
| **A0A0H3JUR8** | SA1666 protein;Uncharacterized protein;LuxR family transcriptional regulator | SA1666;ST398NM01_1914;ACR58 | 8,381E+07 | 6,923E+08 |
| **A0A0H3JUX8** | Aldehyde dehydrogenase | aldH;ST398NM01_2015 | 3,872E+09 | 3,880E+10 |
| **A0A0H3JWJ1** | Uncharacterized protein;Putative tRNA-m1A22 methylase | MW1512;SACOL1617;trmK | 4,766E+07 | 7,174E+07 |
| **A0A0H3JXF0** | Uncharacterized protein;Choloylglycine hydrolase | MW1866;SAZ172_1938;QU38_06900 | 2,494E+08 | 9,986E+08 |
| **A0A0H3JY52** | Cmp-binding-factor 1;3-5 exonuclease | cbf1;ACH32_02120;yhaM | 8,049E+08 | 4,375E+09 |
| **A0A0H3JZ81** | Probable methylated DNA-protein cysteine methyltransferase | adaB | 4,694E+08 | 1,725E+09 |
| **A0A0H3JZU4** | Primosomal protein N | priA | 1,768E+08 | 6,784E+08 |
| **A0A0H3K1I2** | IMMUNOGLOBULIN G BINDING PROTEIN A;Peptigoglycan-binding protein LysM;Immunoglobulin G binding protein | spa;CH51_00450 | 2,613E+08 | 1,950E+09 |
| **A0A0H3K1V2** | MW0336 protein;SA0348 protein;Site-specific DNA-binding protein;ParB-like protein | MW0336;SA0348;parB | 3,578E+08 | 6,049E+08 |
| **A0A0H3K5L3** | Uncharacterized protein;Transcriptional regulator, GntR family;Cell wall surface anchor family protein | NWMN_0079;ST398NM01_0149 | 7,638E+07 | 9,987E+08 |
| **A0A0H3K763** | UvrB/UvrC motif domain protein;DNA binding protein, UvrB/UvrC family protein;Protein-arginine kinase activator protein | NWMN_0485;SAKOR_00508 | 5,302E+07 | 7,054E+07 |
| **A0A0H3K908** | FtsK/SpoIIIE (DNA translocase stage III) family protein;Cell division protein FtsK | NWMN_1634;sftA | 2,108E+09 | 4,212E+09 |
| **A0A0H3KCP9** | Uncharacterized protein;TelA-like protein SAOUHSC_01408;Tellurite resistance protein | NWMN_1317;SAOUHSC_01408;ERS17924 | 1,940E+09 | 2,187E+09 |
| **A0A0H3KED7** | 50S ribosomal protein L29 | rpmC | 9,949E+08 | 1,964E+10 |
| **A0A0K7CIC9** | Invertase;Invertase O | scrB;QU38_06710 | 4,535E+08 | 7,142E+08 |
| **A0A0K7UDI1** | Putative glutathione transporter%2C ATP-binding component;ABC transporter ATP-binding protein | oppF_3;oppF_1 | 1,012E+09 | 1,077E+09 |
| **A0A0M1TX48** | Putative bifunctional signaling protein/50S ribosomal protein L9;Phosphoesterase%2C DHH family protein | RU53_16;nrnA_2 | 5,441E+09 | 1,954E+10 |

| | | | | |
|---|---|---|---|---|
| **A0A0M1U096** | Cysteine-rich secretory protein family protein;Uncharacterized protein;SCP family extracellular protein | RU53_1343;MW1225 | 1,940E+08 | 2,047E+08 |
| **A0A0M1U0I5** | Ferric uptake regulation protein Fur;Ferric uptake regulation protein;Ferric uptake regulation protein FUR | fur | 3,549E+08 | 6,723E+08 |
| **A0A0M1U0W4** | Dihydrolipoyl dehydrogenase | lpdA;AFO | 6,819E+07 | 9,024E+08 |
| **A0A0M1U105** | Lipoamide acyltransferase component of branched-chain alpha-keto acid dehydrogenase complex | bfmB | 1,577E+08 | 2,168E+08 |
| **A0A0M1U3S0** | Phage infection protein;Putative membrane spanning protein;SA2436 protein | RU53_2706;SAKOR_02644 | 4,647E+09 | 5,602E+09 |
| **A0A0T7M2Z6** | Fibrinogen-binding protein;Clumping factor A | clfA | 1,502E+09 | 6,247E+09 |
| **A0A0T7M3E5** | Putative glycosyl transferases;Glycosyl transferase family 1;Glycosyl transferase, group 1 family protein | tagE_3;NWMN_0906 | 6,578E+08 | 9,746E+09 |
| **A0A0T7M481** | 2-oxoglutarate oxidoreductase%2C beta subunit;2-oxoglutarate ferredoxin oxidoreductase subunit beta | porB;HMPREF0776_2291 | 1,713E+08 | 2,617E+08 |
| **A0A0T7M4M7** | 5-nucleotidase;LPXTG-motif cell wall anchor domain protein;MW0023 protein | yfkN_2;BN1321_430113 | 3,686E+07 | 2,866E+08 |
| **A0A0T7M6B9** | DNA/RNA helicase of DEAD/DEAH box family | srmB; | 5,020E+08 | 5,485E+09 |
| **A0A0T7M7H9** | Glycosyl transferase family protein;Glycosyl transferase;Glycosyl transferase group 2 family protein | ERS093009_01950;AFO87_07610;NWMN_01 | 3,915E+09 | 1,755E+10 |
| **A0A0T7M883** | SdrH protein;Serine-aspartate repeat family protein;SA1839 protein | ERS093009_02099;ST398NM01_2069;SA1839 | 4,598E+07 | 4,726E+07 |
| **A0A0T7MUQ9** | Two component system histidine kinase ArlS | arlS | 2,244E+09 | 4,090E+09 |
| **A0A0T7MUR4** | Acyl-CoA dehydrogenase;MW2203 protein;Putative acyl-CoA dehydrogenase YdbM | hsaA;MW2203 | 2,374E+08 | 3,329E+08 |
| **A0A0U0TR45** | LysR family regulatory protein;MW2253 protein | cynR;NWMN_2233;MW2253 | 2,875E+08 | 6,509E+08 |
| **A0A0U1MI56** | Uncharacterized protein;Membrane protein | BN1321_230026;ERS093009_00534;RU53_1101 | 2,862E+08 | 4,440E+08 |
| **A0A0U1MJX5** | Catalase | katA;ST398NM01_1 | 3,234E+09 | 1,888E+10 |
| **A0A0U1MK69** | Pyruvate dehydrogenase (E1 beta subunit) | pdhB | 1,733E+10 | 8,803E+10 |
| **A0A0U1MLN5** | ADP-ribose pyrophosphatase;ADP-ribose pyrophosphatase, NudF | nudF | 3,121E+08 | 6,418E+08 |
| **A0A0U1MLZ6** | HTH-type transcriptional regulator MalR;Maltose operon transcriptional repressor MalR%2C LacI family | malR;mal | 1,081E+08 | 5,881E+08 |
| **A0A0U1MMC9** | tRNA threonylcarbamoyladenosine dehydratase (T(6)A37 dehydratase);ThiF/MoeB family protein | tcdA;ST398NM01_1692 | 8,439E+08 | 1,174E+09 |
| **A0A0U1MMN4** | Beta-ketoacyl-acyl carrier protein reductase;3-ketoacyl-ACP reductase;3-oxoacyl-[acyl-carrier-protein] reductase FabG | fabG;ACH32_13095 | 3,448E+09 | 5,368E+09 |
| **A0A0U1MNK9** | Transcriptional regulator (Fur family);Ferric uptake regulation protein | zur;fur | 1,069E+08 | 1,504E+08 |
| **A0A0U1MP91** | Cell shape-determining protein MreC | BN1326_80190 | 4,374E+08 | 6,767E+08 |
| **A0A0U1MQQ6** | Acetolactate synthase | ilvB | 7,660E+06 | 2,211E+07 |
| **A0A0U1MQU9** | YwpF protein;Uncharacterized protein | ywpF;ACR58_10765;NWMN_2002 | 4,999E+08 | 5,943E+08 |
| **A0A0U1MRD1** | Regulatory protein RecX;Regulatory protein Rec | recX | 5,595E+07 | 1,089E+09 |
| **A0A0U1MSV9** | Nitrate reductase (Beta subunit);Nitrate reductase beta chain narH;NarH protein;Nitrate reductase O | narH | 3,321E+07 | 1,157E+08 |
| **A0A0U1MTM3** | Transcriptional regulator, TetR family;TetR family transcriptional regulator;Uncharacterized protein | BN1321_420075;ACH32_06275 | 8,180E+07 | 2,610E+08 |
| **A0A0U1MTZ5** | Mannose-6-phosphate isomerase | pmi | 3,442E+07 | 8,975E+07 |
| **A0A0U1MTZ8** | Isochorismatase family protein;Isochorismatase | BN1321_430024;ACR58_09925;HMPREF | 6,935E+07 | 2,073E+08 |
| **A0A0U1MWF6** | Multiple sugar-binding transporter ATP-binding protein;Multiple sugar-binding transport ATP-binding protein | msmX | 2,798E+08 | 5,916E+08 |
| **A0A0U1MWH3** | Glycosyl transferase 2 family protein | BN1321_60071 | 1,093E+08 | 2,631E+08 |
| **A0A0U1MX95** | Putative sporulation transcription regulator WhiA | whiA | 3,381E+08 | 3,445E+08 |
| **A0A0U1MXR4** | Histidine transporter;Na+/H+ antiporter family protein;Na+/H+ antiporter NhaC-like protein | hisP;SACOL0946;ACH32_11545 | 2,755E+08 | 3,585E+08 |
| **A6QD96** | HTH-type transcriptional regulator SarS | sarS | 2,350E+08 | 7,731E+09 |
| **A6QED3** | Nucleoid-associated protein NWMN_0443 | NWMN_0443 | 9,228E+07 | 5,375E+08 |
| **A6QEF5** | Peptidyl-tRNA hydrolase | pth | 4,585E+08 | 7,881E+08 |
| **A6QEH1** | Pyridoxal 5-phosphate synthase subunit PdxS | pdxS | 2,489E+09 | 4,446E+09 |
| **A6QEJ7** | 30S ribosomal protein S12 | rpsL | 1,821E+09 | 1,095E+10 |
| **A6QEN4** | Uracil-DNA glycosylase | ung | 7,933E+07 | 4,525E+08 |
| **A6QF37** | NADPH-dependent 7-cyano-7-deazaguanine reductase | queF | 1,114E+08 | 2,438E+08 |
| **A6QFE5** | D-alanine--poly(phosphoribitol) ligase subunit 2 | dltC | 5,487E+08 | 8,636E+08 |
| **A6QFH3** | Glucose-6-phosphate isomerase | pgi | 1,098E+08 | 9,934E+09 |
| **A6QFL7** | NAD kinase | nadK;ppnKA | 3,905E+08 | 3,464E+09 |
| **A6QG41** | Ribonuclease HIII | rnhC | 8,731E+07 | 1,106E+08 |
| **A6QGD8** | 30S ribosomal protein S16 | rpsP | 7,932E+08 | 2,491E+10 |
| **A6QGE5** | Succinyl-CoA ligase [ADP-forming] subunit beta | sucC | 1,756E+09 | 4,755E+09 |
| **A6QGG8** | Translation initiation factor IF-2 | infB | 3,348E+10 | 4,333E+10 |
| **A6QGH0** | tRNA pseudouridine synthase B | truB | 3,756E+08 | 8,292E+08 |
| **A6QH73** | Elongation factor P;Translation elongation factor P%3B Translation initiation factor 5A | efp | 3,912E+08 | 2,484E+09 |
| **A6QHF7** | Putative pre-16S rRNA nuclease | NWMN_1517;yrrK | 3,322E+08 | 8,317E+08 |
| **A6QHN4** | Acetyl-coenzyme A carboxylase carboxyl transferase subunit alpha | accA | 2,021E+09 | 2,238E+09 |
| **A6QHU1** | Leucine--tRNA ligase | leuS | 7,482E+08 | 1,563E+09 |
| **A6QIG2** | 65 kDa membrane protein;77 kDa membrane protein;Protein map | NWMN_1872;SACOL2002;CH51_10545 | 9,219E+10 | 2,018E+11 |
| **A6QJ81** | 50S ribosomal protein L24 | rplX | 4,669E+07 | 5,333E+09 |
| **A6QJ90** | 50S ribosomal protein L23 | rplW | 2,726E+09 | 1,988E+10 |
| **A6QJ91** | 50S ribosomal protein L4 | rplD | 2,441E+09 | 1,569E+10 |
| **A6QK89** | L-lactate dehydrogenase 2;L-lactate dehydrogenase | ldh2;ldhB;ldh | 1,357E+09 | 3,681E+09 |
| **A8IK61** | Uncharacterised protein | AL493_00390 | 2,558E+09 | 1,290E+10 |
| **A8IK64** | Rep protein;Replication protein Rep;Replication protein | ERS092844_02738;ERS365775_02652;SAKOR_02719 | 2,435E+08 | 2,868E+09 |
| **D2J6B7** | DNA integration/recombination/inversion protein;BinL protein;Resolvase, N-terminal domain protein | bin;binL;HMPREF0769_11633 | 1,419E+08 | 2,181E+09 |
| **E9KNS1** | Ornithine carbamoyltransferase | arcB;arcB_2;argF | 7,308E+07 | 1,576E+08 |
| **I7H3R7** | Abortive infection bacteriophage resistance protein;Abi-like protein | AFO87_10805;HMPREF0769_10093 | 4,817E+08 | 1,450E+09 |
| **P0A037** | Glyceraldehyde-3-phosphate dehydrogenase 1;Glyceraldehyde-3-phosphate dehydrogenase | gapA1;gapA | 3,820E+09 | 7,225E+09 |
| **P0A081** | Peptide methionine sulfoxide reductase MsrA 1;Peptide methionine sulfoxide reductase MsrA | msrA1;msrA | 6,998E+08 | 1,071E+09 |
| **P0A0A1** | Pyruvate dehydrogenase E1 component subunit beta | pdhB | 2,365E+10 | 6,193E+11 |
| **P0A0K0** | Uncharacterized protein SAV1875;General stress protein;Intracellular protease, PfpI family | SAV1875;QU38_07190 | 1,108E+08 | 1,274E+09 |
| **P0A0M6** | Putative acetyltransferase SAV1054;Uncharacterized protein;Acetyltransferase, GNAT family | SAV1054;BN1321_220017 | 2,025E+08 | 2,955E+08 |
| **P0A0N9** | Tyrosine recombinase XerD | xerD;xerD_2 | 1,488E+08 | 5,194E+08 |
| **P0C048** | Protein EssC;Type VII secretion protein EssC;Protein essC | essC | 6,302E+09 | 9,332E+09 |
| **P0C1S3** | Transcription termination/antitermination protein NusG | nusG | 5,059E+09 | 6,561E+09 |
| **P60070** | Anti-sigma-B factor antagonist | rsbV | 1,726E+08 | 1,516E+09 |
| **P60090** | Pyruvate dehydrogenase E1 component subunit alpha;Pyruvate dehydrogenase E1 component alpha subunit | pdhA;ST398NM01_1090 | 1,210E+10 | 3,995E+11 |
| **P60380** | Regulatory protein Spx;Regulatory protein Spx OS | spxA | 7,193E+07 | 1,504E+09 |
| **P60430** | 50S ribosomal protein L2 | rplB | 8,257E+09 | 6,052E+10 |
| **P60448** | 50S ribosomal protein L3 | rplC | 1,841E+09 | 2,020E+10 |
| **P63844** | GTP-sensing transcriptional pleiotropic repressor CodY | codY | 2,708E+10 | 3,440E+10 |
| **P63871** | Cysteine synthase | cysK;SAKOR_00498 | 2,360E+10 | 6,655E+10 |
| **P64108** | 3-hydroxyacyl-[acyl-carrier-protein] dehydratase FabZ | fabZ | 7,905E+07 | 5,497E+09 |
| **P64213** | Glycine cleavage system H protein | gcvH | 2,545E+08 | 1,231E+09 |
| **P64290** | Glutathione peroxidase homolog BsaA;Glutathione peroxidase | bsaA;HMPREF0776_2306 | 6,380E+08 | 1,857E+09 |
| **P64333** | Delta-aminolevulinic acid dehydratase | hemB;QU38_08615 | 1,438E+08 | 9,458E+08 |
| **P65139** | Translation initiation factor IF-3 | infC | 8,907E+08 | 1,532E+10 |
| **P65330** | S-ribosylhomocysteine lyase | luxS | 2,267E+08 | 3,876E+08 |
| **P65645** | Probable transglycosylase IsaA;Putative transglycosylase IsaA | isaA | 1,298E+08 | 3,022E+08 |
| **P65806** | Peptidase T | pepT;pepT_1 | 6,448E+07 | 2,276E+08 |
| **P65862** | Phosphate acetyltransferase | pta;p | 4,732E+08 | 2,096E+09 |
| **P65907** | Dihydroorotase | pyrC | 7,337E+08 | 1,007E+09 |
| **P65924** | CTP synthase | pyrG | 7,901E+08 | 1,497E+09 |
| **P66061** | 50S ribosomal protein L7/L12 | rplL | 4,211E+08 | 8,954E+09 |
| **P66133** | 50S ribosomal protein L27 | rpmA | 3,692E+08 | 5,771E+09 |
| **P66196** | 50S ribosomal protein L31 type B | rpmE2 | 3,145E+08 | 4,353E+09 |
| **P66232** | 50S ribosomal protein L33 2;50S ribosomal protein L33 3;50S ribosomal protein L33 | rpmG2;rpmG3;rpmG_2 | 2,257E+08 | 2,586E+08 |
| **P66317** | 50S ribosomal protein L9 | rplI | 4,427E+08 | 5,798E+09 |
| **P66388** | 30S ribosomal protein S13 | rpsM | 2,252E+09 | 2,941E+10 |
| **P66545** | 30S ribosomal protein S2 | rpsB | 2,072E+10 | 2,541E+10 |
| **P66562** | 30S ribosomal protein S4 | rpsD | 9,214E+09 | 5,369E+10 |
| **P66646** | 30S ribosomal protein S9 | rpsI | 2,875E+09 | 3,409E+10 |
| **P66775** | Diacetyl reductase [(S)-acetoin forming];Diacetyl reductase ((S)-acetoin forming);(R,R)-butanediol dehydrogenase | butA | 6,984E+09 | 9,285E+09 |
| **P66866** | Succinyl-CoA ligase [ADP-forming] subunit alpha;Succinyl-CoA ligase | sucD;SAZ172_1250 | 7,040E+08 | 3,408E+09 |
| **P66962** | Transketolase | tkt;tk | 2,326E+09 | 7,851E+09 |
| **P67021** | Glutamate--tRNA ligase | gltX | 5,189E+07 | 3,040E+09 |
| **P67765** | Serine acetyltransferase# | cysE | 1,449E+09 | 2,844E+09 |
| **P68819** | Phosphoglycerate kinase | pgk | 4,004E+09 | 4,889E+09 |
| **P95844** | RNA polymerase sigma factor;RNA polymerase | sigB | 6,717E+07 | 6,891E+08 |
| **P99065** | Peptide methionine sulfoxide reductase MsrB | msrB | 1,926E+08 | 3,774E+08 |
| **P99110** | Chaperone protein DnaK;Ch | dnaK | 8,429E+09 | 2,578E+10 |
| **P99148** | Aconitate hydratase A;Aconitate hydratase;Aconitate hy | acnA;citB;QU38_13120 | 4,922E+09 | 1,016E+10 |
| **P99157** | Alkaline shock protein 23 | asp23 | 8,241E+09 | 4,725E+10 |
| **P99175** | Catabolite control protein A;Transcriptional regulator (LacI family) | ccpA;QU38_08240 | 7,523E+09 | 2,445E+10 |
| **Q1XG25** | Uncharacterised protein | SAP003 | 2,000E+09 | 2,463E+09 |
| **Q2FDH4** | UPF0312 protein SAUSA300_2620;Uncharacterized protein;YceI-like domain protein | SAUSA300_2620;QU38_15655;ERS179246_00851 | 3,042E+08 | 5,259E+08 |
| **Q2FDQ4** | Fructose-bisphosphate aldolase class 1 | fda | 6,548E+09 | 1,371E+10 |
| **Q2FDS6** | Uncharacterized hydrolase SAUSA300_2518;Uncharacterized hydrolase MW2501;Uncharacterized hydrolase SAOUHSC_02900 | SAUSA300_2518;MW2501 | 4,245E+09 | 5,365E+09 |
| **Q2FDY9** | Fructose-1,6-bisphosphatase class 3 | fbp | 4,791E+07 | 7,084E+08 |
| **Q2FE21** | Uncharacterized oxidoreductase SAUSA300_2422;Oxidoreductase, short-chain dehydrogenase/reductase family;Short-chain alc | SAUSA300_2422;SAZ172_2580 | 1,647E+09 | 2,343E+09 |
| **Q2FEQ3** | 30S ribosomal protein S8 | rpsH | 2,077E+09 | 1,325E+10 |

| | | | | |
|---|---|---|---|---|
| **Q2FER5** | DNA-directed RNA polymerase subunit alpha | rpoA | 3,034E+09 | 3,140E+10 |
| **Q2FES1** | 50S ribosomal protein L13 | rplM | 4,258E+09 | 3,777E+10 |
| **Q2FEX1** | Phosphoglucosamine mutase;Phosphoglucosamine | glmM | 1,613E+09 | 1,988E+09 |
| **Q2FF10** | Peptide chain release factor 1 | prfA | 7,177E+08 | 1,592E+09 |
| **Q2FF15** | Serine hydroxymethyltransferase | glyA;CH | 2,430E+09 | 1,419E+10 |
| **Q2FF21** | ATP synthase subunit delta | atpH | 1,972E+09 | 1,935E+10 |
| **Q2FF25** | ATP synthase epsilon chain | atpC | 1,392E+09 | 6,780E+09 |
| **Q2FFK2** | Bacterial non-heme ferritin# | ftnA;HMPREF0769_11279;ST398NM01_1986 | 4,222E+10 | 5,718E+10 |
| **Q2FG06** | Formate--tetrahydrofolate ligase | fhs | 2,727E+09 | 1,424E+10 |
| **Q2FG30** | Uncharacterized peptidase SAUSA300_1654;Uncharacterized peptidase SAV1708;Uncharacterized peptidase MW1651 | SAUSA300_1654;SAV1708 | 1,021E+08 | 3,460E+08 |
| **Q2FG58** | 50S ribosomal protein L20 | rplT | 2,414E+09 | 2,185E+10 |
| **Q2FG80** | 50S ribosomal protein L21 | rplU | 8,674E+09 | 5,457E+10 |
| **Q2FG84** | UPF0735 ACT domain-containing protein SAUSA300_1599;UPF0735 ACT domain-containing protein MW1593 | SAUSA300_1599;MW1593 | 7,223E+07 | 7,224E+07 |
| **Q2FGE8** | 30S ribosomal protein S21 | rpsU | 3,302E+08 | 4,530E+09 |
| **Q2FGG6** | Probable endonuclease 4 | nfo | 6,321E+07 | 2,207E+09 |
| **Q2FGK9** | N utilization substance protein B homolog | nusB | 4,487E+08 | 4,832E+08 |
| **Q2FH94** | LexA repressor | lexA | 4,815E+07 | 2,238E+09 |
| **Q2FH96** | GMP reductase | guaC | 1,066E+08 | 2,787E+09 |
| **Q2FHG5** | 30S ribosomal protein S15;30S ribosomal | rpsO | 1,490E+09 | 1,519E+10 |
| **Q2FHH3** | Ribosome maturation factor RimP | rimP | 1,567E+08 | 2,057E+09 |
| **Q2FHI5** | ATP-dependent protease subunit HslV | hslV;clpQ | 2,379E+08 | 2,874E+08 |
| **Q2FHI8** | DNA topoisomerase 1 | topA | 5,463E+07 | 6,119E+09 |
| **Q2FHT5** | UvrABC system protein C | uvrC | 3,203E+08 | 7,063E+08 |
| **Q2FHT6** | Thioredoxin | trxA;ST398NM01_1141 | 1,538E+09 | 1,073E+10 |
| **Q2FHV3** | 50S ribosomal protein L32 | rpmF | 2,738E+08 | 6,996E+09 |
| **Q2FHX4** | UPF0637 protein SAUSA300_1006;UPF0637 protein MW0989;UPF0637 protein QU38_05785 | SAUSA300_1006;MW0989;QU38_057 | 2,566E+09 | 2,352E+10 |
| **Q2FI12** | Phosphoribosylaminoimidazole-succinocarboxamide synthase | purC | 1,624E+09 | 1,903E+09 |
| **Q2FI15** | Bifunctional protein FolD | folD | 5,934E+09 | 1,048E+10 |
| **Q2FIA8** | ATP-dependent helicase/nuclease subunit A | addA | 4,397E+08 | 2,087E+09 |
| **Q2FIL7** | Enolase | eno | 7,703E+09 | 1,472E+11 |
| **Q2FIM5** | ATP-dependent Clp protease proteolytic subunit | clpP | 8,349E+08 | 6,003E+09 |
| **Q2FIX0** | Probable transcriptional regulatory protein SAUSA300_0655;Probable transcriptional regulatory protein yeeI | SAUSA300_0655;yeeI | 7,040E+08 | 1,718E+09 |
| **Q2FJ94** | 30S ribosomal protein S7 | rpsG | 8,964E+09 | 4,886E+10 |
| **Q2FJ97** | DNA-directed RNA polymerase subunit beta | rpoC | 2,371E+10 | 7,843E+10 |
| **Q2FJ98** | DNA-directed RNA polymerase subunit beta | rpoB | 1,418E+10 | 5,992E+10 |
| **Q2FJE2** | Bifunctional protein GlmU | glmU | 4,254E+08 | 8,918E+08 |
| **Q2FJE4** | Putative septation protein SpoVG | spoVG | 1,842E+08 | 3,630E+10 |
| **Q2FKQ0** | DNA gyrase subunit A;DNA gyrase subunit A | gyrA | 4,696E+09 | 2,055E+10 |
| **Q2FUQ3** | tRNA uridine 5-carboxymethylaminomethyl modification enzyme MnmG | mnmG | 3,013E+08 | 1,263E+09 |
| **Q2FUU5** | Lipase 1;Lipase;Triacylglycerol lipase | lipA;lipA_2;SAKOR_02673 | 3,079E+09 | 1,018E+10 |
| **Q2FVK8** | 2,3-bisphosphoglycerate-dependent phosphoglycerate mutase | gpmA | 4,338E+08 | 4,186E+09 |
| **Q2FVV9** | Putative formate dehydrogenase SAOUHSC_02582;Formate dehydrogenase%2C alpha subunit;Putative formate dehydrogenase SA2 | SAOUHSC_02582;CH51_12440 | 3,691E+09 | 1,152E+10 |
| **Q2FVW4** | Putative 2-hydroxyacid dehydrogenase SAOUHSC_02577;Putative 2-hydroxyacid dehydrogenase SAV2305 | SAOUHSC_02577;SAV2305 | 9,242E+08 | 4,234E+09 |
| **Q2FW28** | Translation initiation factor IF-1 | infA | 2,011E+08 | 2,677E+09 |
| **Q2FW31** | 30S ribosomal protein S11 | rpsK | 3,290E+09 | 1,147E+10 |
| **Q2FW96** | Mannitol-1-phosphate 5-dehydrogenase | mtlD | 1,569E+08 | 3,577E+08 |
| **Q2FWE6** | Uracil phosphoribosyltransferase | upp | 1,141E+10 | 1,442E+10 |
| **Q2FWE9** | ATP synthase gamma chain | atpG | 9,367E+09 | 1,734E+10 |
| **Q2FXD1** | Type I restriction-modification enzyme, S subunit, EcoA family, putative;Probable specificity determinant HsdS | SAOUHSC_01932;hsdS | 2,472E+08 | 1,898E+09 |
| **Q2FXG2** | 6,7-dimethyl-8-ribityllumazine synthase | ribH | 1,471E+08 | 7,363E+09 |
| **Q2FXI2** | tRNA (guanine-N(7)-)-methyltransferase | trmB | 1,307E+08 | 2,047E+08 |
| **Q2FXP1** | Dephospho-CoA kinase | coaE | 1,202E+08 | 7,456E+08 |
| **Q2FXQ0** | 50S ribosomal protein L35 | rpmI | 6,515E+07 | 5,885E+08 |
| **Q2FXQ6** | Trigger factor | tig | 5,886E+08 | 1,731E+10 |
| **Q2FXQ8** | Probable GTP-binding protein EngB | engB | 5,069E+08 | 2,468E+09 |
| **Q2FY15** | DEAD-box ATP-dependent RNA helicase CshB | cshB | 3,557E+09 | 6,936E+09 |
| **Q2FY68** | Pyrroline-5-carboxylate reductase | proC | 6,688E+08 | 9,570E+08 |
| **Q2FY77** | Segregation and condensation protein B | scpB | 1,750E+08 | 1,855E+08 |
| **Q2FYH2** | Uncharacterized protein;Putative TPR-repeat-containing protein, component of Menaquinone-cytochrome C reductase | SAOUHSC_01480;AFO87_08460 | 2,392E+08 | 6,222E+08 |
| **Q2FYN7** | 2,3,4,5-tetrahydropyridine-2,6-dicarboxylate N-acetyltransferase | dapH | 3,432E+08 | 4,936E+08 |
| **Q2FYP3** | Conserved virulence factor B | cvfB | 8,372E+08 | 1,044E+10 |
| **Q2FZ23** | Elongation factor Ts;Elongation factor | tsf | 1,215E+10 | 4,948E+10 |
| **Q2FZ44** | Ribosome maturation factor RimM | rimM | 1,091E+08 | 1,377E+09 |
| **Q2FZ71** | Orotidine 5-phosphate decarboxylase | pyrF | 7,792E+07 | 1,198E+08 |
| **Q2FZ82** | Isoleucine--tRNA ligase | ileS | 1,606E+09 | 5,420E+09 |
| **Q2FZ91** | Cell division protein DivIB | divIB;ftsQ | 1,884E+09 | 2,920E+09 |
| **Q2FZF6** | Uncharacterized protein;Site-specific DNA-methyltransferase (Adenine-specific) | SAOUHSC_01074;RU53_1119 | 2,051E+08 | 2,911E+08 |
| **Q2FZG8** | UPF0356 protein SAOUHSC_01036;UPF0356 protein QU38_05700 | SAOUHSC_01036;QU38_05700 | 1,959E+08 | 7,567E+09 |
| **Q2FZX0** | Acid sugar phosphatase;HAD family hydrolase | nagD;ACH32_11470 | 6,113E+08 | 1,245E+09 |
| **Q2G045** | HPr kinase/phosphorylase | hprK | 6,768E+08 | 1,446E+09 |
| **Q2G0F8** | Arginine--tRNA ligase | argS | 1,634E+09 | 2,789E+09 |
| **Q2G0F9** | Uncharacterized protein | SAOUHSC_00610;MW0570;ACH32_10350 | 4,284E+07 | 6,295E+07 |
| **Q2G0P0** | 50S ribosomal protein L1 | rplA | 1,903E+10 | 6,504E+10 |
| **Q2G0P5** | ATP-dependent Clp protease ATP-binding subunit ClpC;Clp protease ATP binding subunit;Clp protease ClpX | clpC;RU53_511 | 3,001E+10 | 5,155E+10 |
| **Q2G1C0** | 2-C-methyl-D-erythritol 4-phosphate cytidylyltransferase | ispD | 8,937E+08 | 1,844E+09 |
| **Q2G1D8** | Formate acetyltransferase;Formate C-acetyltransferase;Pyruvate formate lyase I | pflB | 1,185E+09 | 2,128E+09 |
| **Q2G1J0** | Putative aldehyde dehydrogenase AldA;Aldehyde dehydrogenase B | aldA | 1,837E+08 | 5,205E+09 |
| **Q2G1W2** | Phosphoenolpyruvate carboxykinase [ATP] | pckA | 3,074E+08 | 3,574E+09 |
| **Q2G1Y0** | DNA ligase | ligA;lig | 4,999E+08 | 4,446E+09 |
| **Q2G227** | Phosphopentomutase | deoB;drm | 7,442E+08 | 1,925E+09 |
| **Q2G235** | Nicotinate phosphoribosyltransferase | SAOUHSC_02133;ERS092844_01372 | 1,957E+08 | 2,233E+09 |
| **Q2G236** | NH(3)-dependent NAD(+) synthetase | nadE | 6,752E+08 | 8,709E+08 |
| **Q2G260** | Uncharacterized protein SAOUHSC_00094;Putative cell-wall-anchored protein SasD (LPXAG motif) | SAOUHSC_00094;sasD | 2,696E+07 | 4,999E+08 |
| **Q2G2F3** | Signal transduction protein TRAP | traP | 7,479E+08 | 7,992E+09 |
| **Q53725** | Cell division protein FtsI (Peptidoglycan synthetase);Penicillin-binding protein 1 | pbpA | 1,088E+10 | 1,388E+10 |
| **Q53753** | Methyltransferase;Ribosomal RNA methyltransferase | ORF202;ybxB;QU38_09720 | 1,582E+08 | 1,328E+09 |
| **Q53757** | DNA integration/recombination/inversion protein;Transposon DNA-invertase | sin;ACR62_14520;B | 2,164E+08 | 2,485E+09 |
| **Q5HCQ9** | Immunodominant staphylococcal antigen B | isaB | 2,679E+09 | 1,493E+10 |
| **Q5HCV3** | 3-methyl-2-oxobutanoate hydroxymethyltransferase | panB | 3,945E+07 | 3,238E+09 |
| **Q5HDL2** | Putative 3-methyladenine DNA glycosylase | SACOL2339;ERS179246_01328 | 6,196E+07 | 7,677E+07 |
| **Q5HDT4** | Molybdopterin molybdenumtransferase | moeA | 3,358E+08 | 7,010E+08 |
| **Q5HDV7** | 30S ribosomal protein S10 | rpsJ | 3,634E+09 | 5,644E+09 |
| **Q5HDW2** | 30S ribosomal protein S19 | rpsS;rps | 2,595E+09 | 4,312E+10 |
| **Q5HDW8** | 50S ribosomal protein L14 | rplN | 7,838E+09 | 1,787E+10 |
| **Q5HDX4** | 50S ribosomal protein L18 | rplR | 5,851E+08 | 8,997E+09 |
| **Q5HDX6** | 50S ribosomal protein L30 | rpmD | 2,806E+08 | 1,870E+09 |
| **Q5HDX7** | 50S ribosomal protein L15 | rplO | 6,544E+09 | 7,739E+10 |
| **Q5HDY1** | 50S ribosomal protein L36 | rpmJ | 7,128E+08 | 4,587E+09 |
| **Q5HDY5** | 50S ribosomal protein L17 | rplQ | 5,467E+08 | 1,975E+10 |
| **Q5HDY9** | tRNA pseudouridine synthase A | truA | 5,374E+07 | 6,656E+07 |
| **Q5HE08** | Lactose phosphotransferase system repressor | lacR | 9,693E+07 | 1,755E+08 |
| **Q5HE95** | ATP synthase subunit alpha | atpA | 2,360E+10 | 1,626E+11 |
| **Q5HEF5** | Redox-sensing transcriptional repressor Rex | rex | 1,222E+09 | 2,141E+09 |
| **Q5HEH2** | 60 kDa chaperonin | groL;groEL | 5,263E+09 | 1,041E+10 |
| **Q5HEN6** | Methionine aminopeptidase | map;mapB | 5,723E+08 | 2,115E+09 |
| **Q5HF61** | Probable thiol peroxidase | tpx | 5,926E+08 | 2,585E+09 |
| **Q5HFA8** | Valine--tRNA ligase | valS | 1,048E+09 | 1,646E+09 |
| **Q5HFC1** | Holliday junction ATP-dependent DNA helicase RuvA | ruvA | 2,426E+08 | 9,445E+08 |
| **Q5HFE4** | Alanine--tRNA ligase;Alanine-- | alaS | 2,906E+09 | 3,165E+09 |
| **Q5HFE5** | UPF0297 protein SACOL1672;UPF0297 protein ST398NM01_1681 | SACOL1672;ST398NM01_1681 | 3,021E+08 | 3,286E+08 |
| **Q5HFI2** | Ribosomal protein L11 methyltransferase | prmA | 4,968E+08 | 5,847E+08 |
| **Q5HFK1** | GTP cyclohydrolase 1 type 2 homolog | SACOL1616;MW1511 | 7,556E+07 | 1,874E+08 |
| **Q5HFM2** | Aminomethyltransferase | gcvT | 6,910E+08 | 1,402E+09 |
| **Q5HFM3** | Probable glycine dehydrogenase (decarboxylating) subunit 1 | gcvPA | 7,276E+08 | 2,052E+09 |
| **Q5HFR2** | 6-phosphogluconate dehydrogenase, decarboxylating | gnd;gndA | 2,597E+08 | 3,222E+09 |
| **Q5HFV7** | Chorismate synthase | aroC;aroC_1;aroC_2 | 6,826E+08 | 6,913E+08 |
| **Q5HFZ9** | Glucose-specific phosphotransferase enzyme IIA component;PTS system, glucose subfamily, IIA component | crr;ptbA | 3,052E+08 | 2,268E+09 |
| **Q5HG04** | Response regulator ArlR;Two-component response regulator (YkoH) | arlR;ykoG | 4,578E+08 | 5,884E+08 |

| | | | | |
|---|---|---|---|---|
| **Q5HGC3** | Glutamine synthetase;Glutamine synthetase OS | glnA | 2,155E+10 | 4,175E+10 |
| **Q5HGC9** | tRNA dimethylallyltransferase | miaA | 1,540E+08 | 3,047E+08 |
| **Q5HGF6** | Ribonuclease J 2;Ribonuclease J | rnj2;ERS179246_02419;HMPREF0769_12503 | 5,443E+09 | 3,318E+10 |
| **Q5HGP5** | Cell division protein FtsZ | ftsZ | 3,974E+10 | 7,374E+10 |
| **Q5HGT2** | Non-canonical purine NTP pyrophosphatase | SACOL1162;QU38_12065 | 1,238E+08 | 2,122E+08 |
| **Q5HGU5** | Phenylalanine--tRNA ligase beta subunit | pheT | 4,951E+08 | 6,069E+09 |
| **Q5HGV4** | Iron-regulated surface determinant protein A;Iron (Fe2+)-regulated surface determinant protein IsdA | isdA | 2,001E+08 | 6,214E+08 |
| **Q5HH01** | Phosphoenolpyruvate-protein phosphotransferase;Phosphoenolpyruvate-protein phosphotransferase OS | ptsI | 3,641E+09 | 5,490E+09 |
| **Q5HH16** | Phosphoribosylformylglycinamidine synthase subunit PurQ | purQ | 9,267E+07 | 1,397E+08 |
| **Q5HH71** | Putative phosphoesterase SACOL1020;Putative phosphoesterase SAV1015 | SACOL1020;SAV1015 | 1,250E+10 | 5,042E+10 |
| **Q5HH88** | Tryptophan--tRNA ligase;Tryptophan--tRNA ligase | trpS | 4,767E+08 | 7,717E+08 |
| **Q5HHA1** | 3-oxoacyl-[acyl-carrier-protein] synthase 2 | fabF | 7,181E+08 | 1,038E+09 |
| **Q5HHA2** | 3-oxoacyl-[acyl-carrier-protein] synthase 3 | fabH | 1,362E+08 | 2,429E+08 |
| **Q5HHB8** | ATP-dependent helicase/deoxyribonuclease subunit B | addB | 1,103E+08 | 1,239E+09 |
| **Q5HHK4** | Methionine import ATP-binding protein MetN 2;Methionine import ATP-binding protein MetN | metN2;metN | 9,211E+09 | 1,192E+10 |
| **Q5HHQ4** | Thioredoxin reductase | trxB;ST398NM01_0840 | 7,353E+08 | 2,652E+09 |
| **Q5HI51** | Transcriptional regulator SarA;Staphylococcal accessory regulator A (SarA) | sarA | 1,371E+09 | 7,727E+10 |
| **Q5HIC5** | Putative pyridoxal phosphate-dependent acyltransferase | SACOL0596 | 5,880E+08 | 1,091E+09 |
| **Q5HID8** | 50S ribosomal protein L11;50S ribosomal protein | rplK | 1,326E+09 | 6,536E+09 |
| **Q5HIR0** | UPF0355 protein SACOL0457;Transcriptional regulator;UPF0355 protein SA0372 | SACOL0457;ERS179246_01611;SA0372 | 5,361E+08 | 4,262E+09 |
| **Q5HIR5** | Alkyl hydroperoxide reductase subunit C;Alkyl hydroperoxide reductase (Small subunit) | ahpC | 2,010E+10 | 6,100E+10 |
| **Q5HIS7** | 30S ribosomal protein S18 | rpsR | 4,615E+08 | 3,837E+09 |
| **Q5HIS8** | Single-stranded DNA-binding protein 1;Single-stranded DNA-binding protein | ssb | 1,335E+08 | 2,745E+09 |
| **Q5HIS9** | 30S ribosomal protein S6 | rpsF | 4,179E+08 | 4,636E+10 |
| **Q5HJH8** | Type-1 restriction enzyme R protein;Type I restriction-modification system restriction subunit | hsdR;SAKOR_00175 | 1,689E+09 | 3,271E+09 |
| **Q5HJZ1** | DNA gyrase subunit B | gyrB | 5,137E+09 | 7,580E+09 |
| **Q6PST7** | CidC;Putative thiamine pyrophosphate-containing protein YdaP;Putative pyruvate oxidase | cidC;ydaP;HMPREF0769_10612 | 1,128E+10 | 1,279E+10 |
| **Q7A0V6** | Cell cycle protein GpsB | gpsB | 2,107E+08 | 1,047E+10 |
| **Q7A0W1** | Conserved virulence factor C | cvfC | 1,472E+08 | 1,798E+08 |
| **Q7A0Y9** | Uncharacterized protein MW1239;Uncharacterized protein;HesB-like protein | MW1239;BN1321_250043;HMPREF0769_12396 | 4,014E+08 | 5,827E+09 |
| **Q7A154** | UPF0348 protein MW1008;UPF0348 protein ERS093009_00554;UPF0348 protein SAZ172_1064 | MW1008;ERS093009_00554;SAZ172_1064 | 1,056E+08 | 1,802E+08 |
| **Q7A1C0** | Putative peptidyl-prolyl cis-trans isomerase;Peptidyl-prolyl cis-trans isomerase | MW0836;BN1321_190076 | 6,206E+08 | 5,308E+09 |
| **Q7A1G5** | Uncharacterized protein MW0714;Uncharacterized protein SACOL0815;Ribosomal subunit interface protein | MW0714;SACOL0815;hpf | 1,563E+10 | 5,530E+10 |
| **Q7A2R1** | Putative dipeptidase SAV1751;Dipeptidase PepV;Aminoacyl-histidine dipeptidase | SAV1751;QU38_04625 | 6,097E+09 | 7,635E+09 |
| **Q7A2R6** | Transcriptional regulatory protein SrrA;Two-component response regulator | srrA;resD | 1,334E+09 | 2,156E+09 |
| **Q7A455** | DNA topoisomerase 3;Putative DNA topoisomerase;DNA topoisomerase | topB;HMPREF07 | 1,539E+08 | 1,297E+09 |
| **Q7A460** | 50S ribosomal protein L22 | rplV | 3,734E+09 | 5,273E+10 |
| **Q7A466** | 50S ribosomal protein L6 | rplF | 5,495E+09 | 5,364E+10 |
| **Q7A4P4** | Uncharacterized protein SA1737;Uncharacterized protein SAUSA300_1902;Uncharacterized protein | SA1737;SAUSA300_1902;BN1321_3 | 2,247E+08 | 7,728E+08 |
| **Q7A4R9** | Response regulator protein VraR;LuxR family two component transcriptional regulator | vraR | 1,787E+09 | 3,565E+09 |
| **Q7A4T3** | Putative multidrug export ATP-binding/permease protein SA1683 | SA1683;ST398N | 4,148E+08 | 2,103E+09 |
| **Q7A5M6** | UPF0403 protein SA1261;Uncharacterized protein | SA1261;CH51_07495;QU38_13560 | 1,191E+08 | 2,360E+08 |
| **Q7A5V7** | Aerobic glycerol-3-phosphate dehydrogenase;Glycerol-3-phosphate dehydrogenase | glpD;HMPREF0776_2302 | 8,336E+09 | 2,378E+10 |
| **Q7A5Z3** | Malonyl CoA-acyl carrier protein transacylase | fabD | 2,219E+08 | 6,461E+08 |
| **Q7A6R4** | Peptide chain release factor 2 | prfB | 1,951E+08 | 6,405E+08 |
| **Q7A6X2** | HTH-type transcriptional regulator MgrA | mgrA | 4,596E+09 | 3,226E+10 |
| **Q7A7B3** | 50S ribosomal protein L25 | rplY | 5,159E+09 | 2,410E+10 |
| **Q7A7S4** | Virulence factor EsxA;WXG100 family type VII secretion target;ESAT-6-like protein | esxA;HMPREF0776_1016 | 5,275E+08 | 2,863E+09 |
| **Q7BWD1** | Beta-lactamase regulator BlaI;CopY family transcriptional regulator | blaI;ACR61_14565;A | 1,895E+08 | 2,099E+08 |
| **Q8NUR2** | 1-pyrroline-5-carboxylate dehydrogenase | rocA | 1,495E+09 | 1,783E+10 |
| **Q8NUR7** | ATP-dependent Clp protease ATP-binding subunit ClpL | clpL | 8,712E+09 | 1,839E+10 |
| **Q8NVA9** | Lipid II:glycine glycyltransferase | femX | 2,561E+09 | 1,366E+10 |
| **Q8NVB4** | 30S ribosomal protein S17 | rpsQ;rps | 5,253E+08 | 3,006E+09 |
| **Q8NVF5** | Deoxyribose-phosphate aldolase 2;Deoxyribose-phosphate aldolase | deoC2;dra_2 | 2,616E+09 | 9,170E+09 |
| **Q8NW84** | tRNA-specific 2-thiouridylase MnmA | mnmA | 1,528E+09 | 1,701E+09 |
| **Q8NWD0** | Probable glycine dehydrogenase (decarboxylating) subunit 2;Glycine dehydrogenase | gcvPB;CH51_08350 | 1,058E+09 | 2,516E+09 |
| **Q8NWM8** | 30S ribosomal protein S1;SSU ribosomal protein S1P | rpsA;QU38_13800;SAKOR_01419 | 1,782E+10 | 7,704E+10 |
| **Q8NWQ9** | Dihydrofolate reductase | folA;dfrA | 1,734E+08 | 4,300E+08 |
| **Q8NWR6** | 2-oxoglutarate dehydrogenase E1 component | odhA | 1,002E+09 | 1,739E+11 |
| **Q8NWR7** | Dihydrolipoyllysine-residue succinyltransferase component of 2-oxoglutarate dehydrogenase complex | odhB | 1,753E+09 | 8,090E+10 |
| **Q8NX76** | Dihydrolipoyllysine-residue acetyltransferase component of pyruvate dehydrogenase complex | pdhC | 1,247E+10 | 6,021E+11 |
| **Q8NXL5** | 2,3-bisphosphoglycerate-independent phosphoglycerate mutase | gpmI | 3,909E+08 | 1,258E+09 |
| **Q8NXY2** | Molecular chaperone Hsp31 and glyoxalase 3 | hchA | 1,031E+09 | 5,426E+09 |
| **Q8NY70** | Inosine-5-monophosphate dehydrogenase | guaB | 5,765E+10 | 6,591E+10 |
| **Q8NY94** | 5-methyltetrahydropteroyltriglutamate--homocysteine methyltransferase | metE | 4,569E+08 | 1,404E+09 |
| **Q8NY95** | Probable acetyl-CoA acyltransferase | MW0330;SACOL0426 | 2,103E+08 | 2,967E+08 |
| **Q8NYY2** | Serine--tRNA ligase | serS | 3,280E+08 | 1,405E+09 |
| **Q931G1** | Urocanate hydratase;Urocanate hydratase | hutU;CH51_12550 | 3,478E+08 | 9,935E+08 |
| **Q931T1** | Ribonuclease 3 | rnc; | 1,720E+09 | 3,867E+09 |
| **Q99RP3** | HTH-type transcriptional regulator SarZ | sarZ | 1,226E+09 | 1,625E+09 |
| **Q99S28** | 50S ribosomal protein L16 | rplP | 1,601E+09 | 3,158E+09 |
| **Q99S39** | Protein translocase subunit SecY | secY | 4,962E+09 | 5,241E+09 |
| **Q99TF3** | Putative universal stress protein SAV1710 | SAV1710 | 3,249E+10 | 3,406E+10 |
| **Q99TQ0** | 5-methylthioadenosine/S-adenosylhomocysteine nucleosidase | mtnN | 6,853E+07 | 1,563E+08 |
| **Q99TT5** | RNA polymerase sigma factor SigA | sigA | 4,046E+08 | 7,533E+09 |
| **Q99U17** | DNA-binding protein HU;Non-specific DNA-binding protein HBsu signal recognition particle-like (SRP) component | hup;hbs | 4,203E+09 | 5,930E+11 |
| **Q99U34** | Probable ATP-dependent helicase DinG homolog;ATP-dependent helicase | dinG | 7,378E+08 | 2,951E+09 |
| **Q99U83** | Acylphosphatase | acyP;SAKOR_01341;ST398NM01_1406 | 1,952E+09 | 3,638E+09 |
| **Q99UJ8** | Polyribonucleotide nucleotidyltransferase;Polyribonucleotide nucleotidyltransferase | pnp;pnpA | 2,215E+09 | 1,670E+10 |
| **Q99UT4** | Uncharacterized N-acetyltransferase SA1019;Uncharacterized N-acetyltransferase SAV1176;Uncharacterized N-acetyltransferase | SA1019;SAV1176 | 1,749E+09 | 7,040E+09 |
| **Q99UZ0** | UPF0358 protein SAV1112;Uncharacterized protein;UPF0358 protein ST398NM01_1109 | SAV1112;ACH32_12440 | 8,594E+07 | 9,903E+07 |
| **Q99V03** | Spermidine/putrescine import ATP-binding protein PotA | potA | 6,055E+08 | 7,813E+08 |
| **Q99V09** | Ribonuclease J 1 | rnj1;ST398NM01_1086;SAKOR_01011 | 1,417E+10 | 4,872E+10 |
| **Q99X11** | FMN-dependent NADH-azoreductase | azoR;azo | 1,849E+09 | 2,497E+09 |
| **Q9AGT0** | Putative TrmH family tRNA/rRNA methyltransferase;23S rRNA Gm2251 methyltransferase | NWMN_0494;SAKOR_00517 | 7,706E+08 | 5,459E+09 |
| **Q9EZ12** | 4-hydroxy-tetrahydrodipicolinate synthase | dapA | 6,854E+07 | 1,781E+08 |
| **Q9EZ13** | Aspartate-semialdehyde dehydrogenase | asd | 2,797E+09 | 4,013E+09 |
| **Q9F0R1** | HTH-type transcriptional regulator SarR | sarR | 1,561E+09 | 3,348E+10 |
| **Q9FDP3** | Replication initiator A domain-containing protein;Rep protein | rep;ERS093009_02656 | 7,705E+08 | 5,544E+09 |
| **Q9KWZ8** | Putative staphylococcal protein;Uncharacterized protein;Uncharacterised protein | ORF141;HMPREF0776_2786;ERS179246_01528 | 9,501E+08 | 9,267E+09 |
| **Q9L4P8** | Betaine aldehyde dehydrogenase;Betaine-aldehyde dehydrogenase | gbsA;QU38_16060 | 2,779E+06 | 6,077E+06 |
| **Q9R744** | PBP2;Penicillin binding transpeptidase domain protein;Uncharacterized protein | pbp2;BN1321_260090 | 1,158E+10 | 1,969E+10 |
| **Q9RL93** | DNA-3-methyladenine glycosidase;DNA-3-methyladenine glycosylase;DNA-3-methyladenine glycosylase I | tag;SACOL1711; | 2,223E+08 | 4,127E+09 |
| **T1Y6B2** | Acid phosphatase;5-nucleotidase | SAKOR_00299;hel;SAZ172_0307 | 2,870E+09 | 9,761E+09 |
| **T1Y6D6** | 33 kDa chaperonin | hslO | 5,408E+08 | 1,011E+09 |
| **T1Y6P6** | Putative heme-dependent peroxidase SAKOR_00577;Putative heme-dependent peroxidase SAV0587 | SAKOR_00577;SAV0587 | 3,218E+08 | 1,875E+09 |
| **T1Y6T0** | Glyoxylate reductase (NADP+);2-ketogluconate reductase;Glyoxylate reductase / Glyoxylate reductase / Hydroxypyruvate reductase | SAKOR_00841;ghrB_1 | 2,851E+08 | 3,865E+08 |
| **T1Y735** | GTP cyclohydrolase FolE2 | folE2 | 3,338E+08 | 7,352E+08 |
| **T1Y7K1** | GTP pyrophosphokinase | SAKOR_00922;ACH32_11865;SA | 9,920E+07 | 2,656E+08 |
| **T1Y8S5** | Staphopain;Staphopain B | SAKOR_00968;sspB | 6,143E+08 | 1,592E+09 |
| **T1Y8Z3** | Cytidylate kinase | cmk;CH51_07730 | 1,028E+09 | 1,130E+09 |
| **T1Y913** | D-alanine--poly(phosphoribitol) ligase subunit 1 | dltA | 3,329E+08 | 6,726E+08 |
| **T1Y918** | 23S rRNA methyltransferase;SpoU rRNA methylase family protein;RNA methyltransferase, TrmH family | SAKOR_01058;spoU | 3,880E+08 | 2,196E+09 |
| **T1Y925** | Queuine tRNA-ribosyltransferase | tgt | 4,298E+08 | 2,237E+09 |
| **T1Y9B4** | Methionyl-tRNA formyltransferase | fmt | 4,130E+08 | 1,484E+09 |
| **T1YA47** | Fumarate hydratase class II | fumC | 8,961E+08 | 2,217E+09 |
| **T1YA57** | DNA polymerase | SAKOR_01630;polA | 2,409E+09 | 1,323E+10 |
| **T1YAB2** | Pseudouridine synthase | SAKOR_01695;ORF231;HMPREF0776_2787 | 7,082E+07 | 3,966E+08 |
| **T1YAM2** | Single-stranded-DNA-specific exonuclease recJ;Single-stranded-DNA exonuclease;MW1586 protein | SAKOR_01583;recJ;MW1586 | 2,669E+08 | 4,025E+08 |
| **T1YBG9** | Salicylate hydroxylase;FAD binding domain protein | SAKOR_02272;HMPREF0776_0355;nagX | 2,268E+09 | 2,489E+09 |
| **T1YBI2** | Glyoxalase family protein;Glyoxalase;Glyoxalase/bleomycin resistance protein/dioxygenase superfamily protein | SAKOR_02511;catE_1 | 8,705E+07 | 1,680E+08 |
| **T1YBW1** | Sulfite reductase [NADPH] flavoprotein alpha-component | SAKOR_02616;SAOUHSC_02947 | 3,204E+08 | 3,406E+08 |
| **T1YBW9** | Transcription accessory protein (S1 RNA binding domain);RNA-binding transcriptional accessory protein | SAKOR_02028;AFO87_07805 | 1,451E+09 | 6,772E+09 |
| **T1YCC7** | Carboxylic ester hydrolase | SAKOR_02435;pnbA;NWMN_2350 | 5,732E+08 | 6,091E+08 |
| **W8TPA6** | Uncharacterised protein;Uncharacterized protein | AFO87_12365;MW0362;ST398NM01_0463 | 6,287E+08 | 8,901E+08 |
| **W8TRI2** | Protein of uncharacterised function (DUF402);Uncharacterized protein | AFO87_03035;BN1321_170001;NWMN_0640 | 2,834E+08 | 1,461E+09 |
| **W8TRN2** | Exported protein;Uncharacterized protein | AFO87_03325;RU53_676;HMPREF0776_ | 1,227E+09 | 2,790E+09 |
| **W8TU69** | DNA-binding transcriptional regulator;DeoR-like helix-turn-helix domain protein;DeoR faimly transcriptional regulator | AFO87_00880;BN1321_380150 | 3,333E+08 | 1,466E+09 |

| | | | | |
|---|---|---|---|---|
| **W8TVI2** | Glutamyl aminopeptidase;MW1688 protein;Peptidase M28;M42 family glutamyl aminopeptidase OS | pepA_2;MW1688;QU38_08185 | 1,092E+09 | 4,591E+09 |
| **W8TZV9** | DNA-binding protein;Putative cytosolic protein;Uncharacterized protein | ylbN;SAKOR_01048;BN1326_60243 | 6,900E+07 | 1,398E+08 |
| **W8U115** | Uncharacterised protein;Uncharacterized protein | AFO87_02995;ST398NM01_0756 | 8,094E+09 | 1,498E+10 |
| **W8U1I5** | Signal peptidase;PSP1 C-terminal domain protein;Signal peptidase II | yaaT;HMPREF0769_12828;QU38_03060 | 1,550E+09 | 3,610E+09 |
| **W8U2C9** | Alpha-helical coiled-coil protein;Uncharacterized protein | srpF;AL077_00800;BN1326_30034 | 2,953E+08 | 1,223E+09 |
| **W8U3A1** | Alkyl hydroperoxide reductase AhpD | AFO87_06430;ST398NM01_2524; | 8,849E+08 | 3,946E+09 |
| **W8U4T0** | Uncharacterised protein;Uncharacterized protein | AFO87_13550;ERS093009_00540;HMPREF0776_2104 | 1,384E+09 | 2,527E+09 |
| **W8U4V4** | Glucosamine-6-phosphate deaminase;Glucosamine-6-phosphate isomerase;Glucosamine-6-phosphate isomerase family protein | AFO87_07265;QU38_03975 | 1,665E+10 | 5,758E+10 |
| **W8U565** | tRNA-binding domain-containing protein;Putative tRNA binding protein;tRNA-binding protein | pheT_2;ytpR;QU38_08200 | 5,179E+08 | 1,448E+09 |
| **W8U635** | Oligoendopeptidase F;MW1272 protein | pepF1_1;MW1272;QU38_13330 | 3,511E+07 | 1,677E+09 |
| **W8U665** | 6-phospho 3-hexuloisomerase;6-phospho-3-hexuloisomerase (PHI);SIS domain protein | hxlB | 6,970E+07 | 1,948E+08 |
| **W8U6C2** | ATP-dependent zinc metalloprotease FtsH | ftsH | 4,239E+10 | 4,643E+10 |
| **W8U8R7** | Glyoxalase-like domain protein;Putative cytosolic protein;Uncharacterized protein | AFO87_01145;SAKOR_02227;NWMN_2164 | 5,187E+08 | 1,025E+09 |
| **W8URL3** | Uncharacterised protein;Uncharacterized protein | AFO87_13610;HMPREF0776_2115;QU38_05870 | 9,295E+07 | 1,069E+08 |
| **W8UVT0** | Uncharacterised protein;Uncharacterized protein;Uncharacterized protein OS | AFO87_08135;NWMN_2088;QU38_03705 | 7,032E+10 | 9,201E+10 |
| **W8UWM8** | Pseudouridine synthase;Ribosomal large subunit pseudouridine synthase D-like protein;Pseudouridine synthase, RluA family | yhcT;SAZ172_1861 | 1,578E+09 | 1,173E+10 |
| **W8UXT9** | Glyoxalase;Glyoxalase family protein | AFO87_05460;HMPREF0776_2430;SAKOR_01348 | 9,304E+09 | 1,570E+10 |
| **X5DRN2** | Ribosome biogenesis GTPase A | rbgA;MW1126;SA1086 | 1,127E+09 | 1,325E+09 |
| **X5DRS2** | Lipoprotein;Uncharacterized protein | AFO87_12765;SA0943;SAZ172_1031 | 5,732E+09 | 6,544E+09 |
| **X5DRY1** | ABC transporter;Uncharacterized protein;Protein co-occurring with transport systems | yigZ;CH51_03905;SAZ172_0758 | 1,302E+08 | 1,406E+08 |
| **X5DSI3** | Metal dependent hydrolase;Uncharacterized protein;Phosphohydrolase | yedJ;MW2012;SA1892 | 1,224E+08 | 2,186E+08 |
| **X5DXG8** | Mechanosensitive ion channel protein MscS | kefA | 2,920E+09 | 4,161E+09 |
| **X5DYJ1** | Transcription termination/antitermination protein NusA | nusA | 6,758E+09 | 1,538E+10 |
| **X5DZ51** | Ribosomal silencing factor RsfS | ybeB;rsfS | 1,025E+08 | 1,298E+09 |
| **X5DZG3** | Glutathione S-transferase;Uncharacterized protein;Glutathione S-transferase-related transmembrane protein | AFO87_00395;SA2193;ERS179246_017 | 1,667E+08 | 3,132E+08 |
| **X5E0J0** | Ribonuclease H;SA1266 protein;MW1323 protein | rnhA;SA1266;MW1323 | 1,339E+08 | 1,721E+08 |
| **X5E0N6** | Uncharacterised protein;Uncharacterized protein | AFO87_10535;AL077_10410;BN1326_60450 | 6,117E+08 | 2,022E+09 |
| **X5E151** | Toxin | map-w | 4,428E+07 | 1,065E+08 |
| **X5E164** | Thioredoxin;Putative thioredoxin or thiol-disulfide isomerase;MW0784 protein | trxA_3;ydbP;MW0784 | 3,763E+08 | 3,929E+09 |
| **X5E1N1** | DNA-binding protein;Uncharacterized protein | AFO87_12370;HMPREF0769_10071;ERS179246_01609 | 1,948E+07 | 6,274E+09 |
| **X5E2F5** | N6-adenine-specific DNA methylase;Methyltransferase;Site-specific DNA-methyltransferase (Adenine-specific) | rlmL;SAKOR_01386 | 1,953E+08 | 1,673E+09 |
| **X5EG20** | Glycine betaine ABC transport system%2C ATP-binding protein OpuAA;Glycine betaine transport ATP-binding protein | proV_1;SAKOR_00720 | 5,665E+09 | 8,456E+09 |
| **X5EGR8** | General stress protein;Pyridoxamine 5-phosphate oxidase family protein | ydaG;HMPREF0769_10765;QU3 | 5,476E+09 | 1,022E+10 |
| **X5EH11** | Leucine aminopeptidase 3, chloroplastic | pepA_2;pepA_1 | 1,366E+09 | 1,459E+09 |
| **X5EJM2** | Aldo/keto reductase family protein;Oxidoreductase ion channel;Oxidoreductase, aldo/keto reductase family protein | iolS;HMPREF0769_ | 5,072E+08 | 1,003E+09 |
| **X5EJX1** | Heptaprenyl diphosphate synthase (HEPPP synthase) subunit 1 family protein;Heptaprenyl diphosphate synthase component I | AFO87_08500;SAKOR_01414 | 3,353E+08 | 3,792E+08 |
| **X5EKN6** | Threonine synthase | thrC;ST398NM01_1331 | 2,947E+08 | 3,763E+08 |
| **X5EKP1** | RNA binding protein;RNA-binding protein, YhbY family;RNA-binding protein | yhbY;HMPREF0769_12085;ACH32_00820 | 1,460E+09 | 8,516E+09 |
| **X5EL59** | Cys-tRNA(Pro)/Cys-tRNA(Cys) deacylase | ybaK;HMPREF0769_11602 | 1,352E+08 | 3,771E+08 |
| **X5ELM4** | Glycosyl transferase family 1;Poly(Glycerol-phosphate) alpha-glucosyltransferase;MW0520 protein | tagE_1;SAKOR_00553;MW0520 | 7,288E+08 | 6,469E+09 |
| **X5EMW4** | HAD family hydrolase;Haloacid dehalogenase-like hydrolase | AFO87_10530;RU53_1336 | 2,893E+09 | 3,700E+09 |
| **X5ENI9** | Acyl-CoA thioester hydrolase;Uncharacterized protein | AFO87_05945;RU53_2601;QU38_16 | 1,189E+08 | 2,098E+08 |

## Population III. DRM enriched

| Uniprot-ID | Protein Name | Gene Name | LFQ Intensity DRM | LFQ Intensity |
|---|---|---|---|---|
| **A0A033V1Y1** | Asparagine--tRNA ligase | asnS | 2,053E+09 | 9,933E+08 |
| **A0A068DYF6** | ATP-dependent helicase;DNA polymerase III alpha subunit | dnaQ;ST398NM01_2958 | 1,910E+09 | 1,031E+09 |
| **A0A069G068** | ATP synthase subunit b | atpF | 1,172E+10 | 1,683E+09 |
| **A0A069G2W6** | DEAD-box ATP-dependent RNA helicase CshA | cshA | 7,171E+08 | 6,466E+08 |
| **A0A069G852** | DNA gyrase subunit B | gyrB | 6,130E+08 | 5,085E+08 |
| **A0A069G9I2** | Citrate synthase | citZ | 4,711E+08 | 4,423E+08 |
| **A0A069GED4** | Tyrosine--tRNA ligase | tyrS | 1,472E+08 | 1,099E+08 |
| **A0A0B6XMR5** | Signal recognition particle protein;Signal recognition particle | ffh;CH51_06360 | 3,350E+09 | 2,450E+09 |
| **A0A0D1FGD2** | Cro/Cl family transcriptional regulator;Helix-turn-helix family protein | ACR58_02450;BN1321_240130 | 5,031E+09 | 1,581E+08 |
| **A0A0D1G477** | Alkaline stress response protein;Uncharacterized protein | ACH32_00470;yqhY | 4,651E+09 | 1,620E+09 |
| **A0A0D1GMN8** | Hydrolase;Cof-like hydrolase;Hydrolase (HAD superfamily) | ACH32_11695;HMPREF0769_11409;yidA | 1,089E+09 | 3,504E+08 |
| **A0A0D1GNJ9** | Putative pit accessory protein;TIGR00153 family protein | ACH32_10630;CO98_0830 | 1,858E+09 | 1,553E+09 |
| **A0A0D1GPL9** | RipR family HTH-type transcriptional regulator;RpiR family transcriptional regulator | ACH32_08240;ybbH_3 | 1,485E+09 | 9,300E+08 |
| **A0A0D1GQ49** | Antibiotic ABC transporter ATP-binding protein;ATP-binding cassette transporter A | bmrA;MW0605 | 6,767E+10 | 1,463E+10 |
| **A0A0D1GTH3** | Uncharacterized protein | ACR58_08110;HMPREF0776_0401 | 6,686E+08 | 5,532E+08 |
| **A0A0D1GXI2** | Uncharacterized protein | ACH32_03885;BN1326_110071 | 3,746E+09 | 3,756E+08 |
| **A0A0D1H0H5** | Membrane protein;Uncharacterized protein;Uncharacterized protein (Fragment) | ACH32_13775;yhhT_2;BN1326_60478 | 1,307E+10 | 5,717E+08 |
| **A0A0D1H1I6** | Calcineurin-like phosphoesterase family protein;Putative metallophosphoesterase | ACH32_13400;HMPREF0769_12490 | 1,548E+09 | 6,014E+08 |
| **A0A0D1H5S7** | Preprotein translocase subunit YajC | yajC | 2,270E+10 | 1,686E+09 |
| **A0A0D1H7B3** | Octopine dehydrogenase;Opine dehydrogenase;NAD/NADP octopine/nopaline dehydrogenase, alpha-helical domain protein | ACR58_07860;SAKOR_02267 | 4,734E+09 | 1,677E+09 |
| **A0A0D1H7N5** | Uncharacterized protein | ACH32_02105 | 2,566E+10 | 2,794E+09 |
| **A0A0D1H8Q7** | Allophanate hydrolase;Allophanate hydrolase subunit 2 family protein;Regulator of kinase autophosphorylation inhibitor | ACH32_10920;ERS179246_00185 | 9,699E+08 | 2,257E+08 |
| **A0A0D1HAI9** | Manganese ABC transporter substrate-binding protein;ABC transporter, substrate-binding protein, putative | mntA;SAOUHSC_00634 | 4,886E+11 | 2,390E+10 |
| **A0A0D1HE14** | Exported protein;Putative N-acetyltransferase YedL | ACH32_02720;CO98_1537 | 3,531E+10 | 2,273E+09 |
| **A0A0D1HEG6** | Ribonucleoside-diphosphate reductase beta chain | nrdF;ST398NM01_0809 | 1,591E+10 | 6,774E+09 |
| **A0A0D1HFY3** | Bacitracin ABC transporter ATP-binding protein;ABC transporter, ATP-binding protein | ACR58_11760;HMPREF0776_1664 | 1,013E+09 | 4,416E+08 |
| **A0A0D1HHD7** | Alpha-hemolysin;Alpha-Hemolysin | QU38_12130;hly | 3,904E+09 | 2,235E+09 |
| **A0A0D1HHR3** | Aspartokinase | ACH32_13600;lysC2;thrA | 6,653E+08 | 1,857E+08 |
| **A0A0D1HLR6** | Iron transporter;Uncharacterized protein | ACR58_01050;BN1321_260210;AL078_01305 | 1,198E+10 | 9,481E+08 |
| **A0A0D1HPR5** | Uncharacterized protein;CBS domain protein | ACH32_11455;yutD;NWMN_0797 | 1,083E+09 | 2,864E+08 |
| **A0A0D1HRD3** | GTP pyrophosphokinase;GTP pyrophosphokinase (RelA/SpoT) | ACR58_01360;relA;rsh | 8,185E+09 | 5,970E+09 |
| **A0A0D1HT39** | NADH dehydrogenase;MW0820 protein;Pyridine nucleotide-disulfide oxidoreductase | ACH32_11520;MW0820 | 7,792E+09 | 4,372E+09 |
| **A0A0D1HVY3** | Nitroreductase;Nitroreductase family protein | ACR58_11995;HMPREF0769_11129 | 3,294E+08 | 7,232E+07 |
| **A0A0D1HW65** | GTP-binding protein YqeH;GTP-binding protein YqeH, required for biogenesis of 30S ribosome subunit;GTP-binding protein | ACR58_01175;SAZ172_1609 | 8,785E+08 | 4,535E+08 |
| **A0A0D1HWL9** | Acetyltransferase;Acetyltransferase family protein | ACR61_11680;BN1321_340069;RU53_2191 | 6,362E+08 | 5,191E+08 |
| **A0A0D1HYP5** | HTH-type transcriptional regulator;Uncharacterized protein | ACH32_03840;NWMN_2027;BN1326_110062 | 1,743E+08 | 7,126E+07 |
| **A0A0D1HZ15** | Cell division protein DivIC;Putative cell division protein | divIC;BN1326_40028 | 1,549E+09 | 1,439E+09 |
| **A0A0D1I198** | Ferrichrome transport ATP-binding protein fhuC;Ferrichrome transport ATP-binding protein fhuA | fhuC;fhuA | 6,409E+08 | 5,042E+08 |
| **A0A0D1I4G6** | SAM-dependent methyltransferase | QU38_14455;ubiE_1 | 6,072E+07 | 1,922E+07 |
| **A0A0D1IB15** | Phosphoserine phosphatase rsbU;RsbU;Putative sigma factor sigB regulation protein | rsbU;ST398NM01_2108 | 4,179E+09 | 1,432E+09 |
| **A0A0D1ICI0** | Pur operon repressor;PurR: transcription regulator associated with purine metabolism | ACH32_09685;purR | 8,779E+09 | 4,096E+09 |
| **A0A0D1IGF8** | Replicative DNA helicase | ACH32_07115;dnaC;SAKOR_000 | 8,769E+08 | 6,117E+08 |
| **A0A0D1IIB4** | Riboflavin biosynthesis protein | ribC;ribF | 1,250E+09 | 4,214E+08 |
| **A0A0D1IMW5** | Cytoplasmic protein;Uncharacterized protein | ACR58_07015;HMPREF0776_0816; | 6,041E+07 | 4,544E+07 |
| **A0A0D1IQC2** | DoxX family protein;Uncharacterized protein | ACH32_10865;AUC48_03600;BN1326_50209 | 3,048E+10 | 5,420E+08 |
| **A0A0D1IUZ1** | Citrate synthase;Uncharacterized protein | citZ;HMPREF0769_11994 | 3,762E+09 | 2,676E+09 |
| **A0A0D1JJX2** | Mevalonate kinase | mvk;mvaK1;ST398NM01_0664 | 2,294E+08 | 1,381E+08 |
| **A0A0D1JP24** | Alkaline shock protein;General stress protein%2C Gls24 family;Uncharacterized protein | ACH32_13065;ERS179246_01154;yloU | 8,853E+09 | 2,620E+09 |
| **A0A0D1JPY7** | Zinc metallopeptidase;Neutral zinc metallopeptidase family;Putative neutral zinc metallopeptidase | ACH32_00135;yugP | 3,036E+10 | 1,092E+09 |
| **A0A0D1JZA3** | 1-acyl-sn-glycerol-3-phosphate acyltransferase | ACH32_01480;plsC | 3,076E+09 | 1,039E+09 |
| **A0A0D1K1L5** | ABC transporter permease;ABC transporter%2C permease protein YbbP clustered with maltose/maltodextrin transporter / Tlr1762 p | ACH32_04135;cdaA | 4,177E+09 | 1,890E+08 |
| **A0A0D1K376** | Ribosomal RNA small subunit methyltransferase A | rsmA | 1,442E+09 | 6,097E+08 |
| **A0A0D1K843** | ComE operon protein 2 | comEB;HMPREF0769_12091;HMP | 4,911E+08 | 2,329E+08 |
| **A0A0D3Q3Y1** | Outer surface protein;Uncharacterized protein | AFO87_12035;SAOUHSC_00156;MW0164 | 1,213E+09 | 4,345E+08 |
| **A0A0D3Q467** | Putative secretion accessory protein EsaA/YueB | esaA;e | 1,619E+10 | 5,095E+09 |
| **A0A0D3Q4J1** | Putative cytosolic protein | AL493_02900 | 2,407E+08 | 8,838E+07 |
| **A0A0D3Q4K0** | ABC transporter permease protein;ABC transporter permease;ABC superfamily ATP binding cassette transporter, membrane protein | AFO87_05235;AL077_01500 | 5,539E+09 | 2,882E+08 |
| **A0A0D3Q778** | ABC transporter ATP-binding protein;ABC efflux transporter (ATP-binding protein) | yheS_1;ykpA;CH51_07295 | 6,513E+09 | 3,986E+09 |
| **A0A0D3Q7N8** | DNA repair protein RecN | recN;SAOUHSC_01615 | 1,708E+09 | 6,613E+08 |
| **A0A0D3Q886** | Acetoin utilization protein;Protein acetyltransferase | acuA;QU38_08250 | 1,093E+09 | 4,769E+08 |
| **A0A0D3Q8P4** | Lantibiotic ABC transporter ATP-binding protein;Lantibiotic immunity protein F;Lantibiotic transport ATP-binding protein SrtF | bsaF; | 4,364E+09 | 2,367E+09 |
| **A0A0D3Q904** | ABC transporter ATP-binding protein;Multidrug ABC transporter ATP-binding protein | ecsA_3;ACH32_02095;ST398NM01_19 | 8,139E+08 | 4,856E+08 |
| **A0A0D3Q935** | Exported protein;Uncharacterized protein | AFO87_15140;HMPREF0776_2820;QU38_04795 | 9,830E+10 | 1,301E+10 |
| **A0A0D3Q9P6** | ATPase;ATPase AAA | clpB_1;CH51_11960 | 9,721E+08 | 1,983E+08 |
| **A0A0D3QA12** | Multidrug effluc protein;Multidrug resistance protein (Function not yet clear) | AFO87_00670;yhbJ;ERS092844_01653 | 3,305E+09 | 4,477E+08 |
| **A0A0D3QA76** | ABC-type transport system%2C ATPase component;ABC transporter, ATP-binding protein | ecsA_2 | 7,293E+09 | 7,136E+09 |
| **A0A0D3QAA6** | Amino acid permease;Uncharacterized protein | AFO87_00175;AL078_02870;HMPREF0769_10705 | 7,640E+09 | 1,590E+09 |
| **A0A0D3QAD8** | Membrane protein;Putative membrane protein;Uncharacterized protein | AFO87_00935;BN1321_380138;ERS179246_01286 | 2,819E+09 | 8,167E+08 |

| | | | | |
|---|---|---|---|---|
| **A0A0D3QBD9** | Activator of the mannose operon (Transcriptional antiterminator)%2C BglG family;Transcription antiterminator, BglG family;SA2433 | licR_1;RU53_2703 | 3,304E+08 | 1,101E+08 |
| **A0A0D3QBK7** | Threonylcarbamoyl-AMP synthase | rimN_2;SACOL2108;SAOUHSC_02 | 8,025E+08 | 6,878E+08 |
| **A0A0D6G5Y4** | Alpha/beta fold family hydrolase;Hydrolase, alpha/beta domain protein;Lysophospholipase | pip;HMPREF0776_1620;QU | 3,513E+08 | 1,573E+08 |
| **A0A0D6G8E8** | PTS system trehalose-specific transporter subunit IIBC;Trehalose permease IIC protein | treB;QU38_03485 | 8,574E+09 | 2,470E+08 |
| **A0A0D6GCD0** | Lipoprotein;Peptidase;Putative secreted protease inhibitor | AFO87_12435;CH51_01905;ACH32_09125 | 4,202E+10 | 8,575E+09 |
| **A0A0D6GEC2** | Acetyl esterase;Uncharacterized protein;Uncharacterized protein | AFO87_05170;MW0298;SAKOR_00315 | 2,996E+08 | 1,621E+08 |
| **A0A0D6GFF2** | Uncharacterized conserved protein | AFO87_05290 | 8,808E+08 | 5,855E+08 |
| **A0A0D6GFY0** | CDP-glycerol glycerophosphotransferase;Uncharacterized protein;MW0230 protein | tagF_2;NWMN_0188;MW0230 | 8,747E+08 | 3,712E+08 |
| **A0A0D6GG32** | Ribokinase;Probable ribokinase | rbsK | 3,185E+08 | 2,960E+08 |
| **A0A0D6GG59** | Glycerophosphodiester phosphodiesterase;Glycerophosphoryl diester phosphodiesterase | ugpQ_1;NWMN_0164 | 1,708E+09 | 4,591E+08 |
| **A0A0D6GHM9** | ABC-type nitrate/sulfonate/bicarbonate transporter%2C TauA;SA0167 protein | tauA | 9,986E+09 | 4,055E+08 |
| **A0A0D6GM89** | LysR family transcriptional regulator;Transcriptional regulator, LysR family | gltR;QU38_15300 | 4,476E+08 | 3,721E+08 |
| **A0A0D6GS23** | Tributyrin esterase;MW2550 protein;Putative esterase | AFO87_06510;MW2550;SAZ172_2748 | 3,195E+08 | 1,232E+08 |
| **A0A0D6GTK5** | 3-hydroxy-3-methylglutaryl coenzyme A reductase | mvaA;CH51_13615 | 1,756E+09 | 9,572E+08 |
| **A0A0D6GUP8** | Esterase;Alpha/beta hydrolase fold-3 domain-containing protein | mlhB_1;ST398NM01_2585;aes | 1,310E+10 | 6,052E+09 |
| **A0A0D6GV05** | 3-hydroxy-3-methylglutaryl CoA synthase;Hydroxymethylglutaryl-CoA synthase | mvaS;SAKOR_02536;BN1321_ | 9,762E+08 | 5,698E+08 |
| **A0A0D6GXG5** | Methionine ABC transporter ATP-binding protein;ABC transporter, ATP-binding protein | ybbL_2;HMPREF0776_0514 | 6,472E+08 | 6,284E+08 |
| **A0A0D6GZM2** | Nitrate reductase;Nitrate reductase (Alpha subunit);Respiratory nitrate reductase subunit alpha | narG | 9,286E+07 | 8,696E+07 |
| **A0A0D6H1J5** | PTS maltose transporter subunit IIBC;Phosphotransferase system (PTS) trehalose-specific enzyme IIBC component | scrA;treP | 4,860E+09 | 9,974E+07 |
| **A0A0D6H9G0** | Lytic regulatory protein;Putative membrane protein;Uncharacterized protein | AFO87_07705;BN1321_350011;HMPREF0769_11010 | 2,957E+09 | 8,989E+08 |
| **A0A0D6HAQ4** | Histidine kinase;Sensor protein kdpD;KdpD | kdpD;QU38_06480;SAKOR_02044 | 1,241E+09 | 8,386E+08 |
| **A0A0D6HB15** | Cell wall surface anchor family protein;Uncharacterized protein;Plasmin and fibronectin-binding protein A | pfbA;SAKOR_01880 | 9,302E+08 | 5,871E+08 |
| **A0A0D6HBZ8** | Lipoprotein;Lipoprotein (Pheromone) | AFO87_10325;QU38_07020;camS | 1,395E+10 | 1,378E+09 |
| **A0A0D6HC09** | Inactive protein of metal-dependent protease family%2C putative molecular chaperone | ydiC;NWMN_1958 | 7,692E+08 | 2,722E+08 |
| **A0A0D6HD63** | DNA repair protein Rad50;Uncharacterized protein | AFO87_07330;NWMN_1735;MW1784 | 6,179E+10 | 1,801E+10 |
| **A0A0D6HDA2** | UDP-N-acetylmuramate--alanine ligase;UDP-N-acetylmuramyl tripeptide synthetase, putative | AFO87_14830;ERS179246_02392 | 1,611E+09 | 6,722E+08 |
| **A0A0D6HDD9** | Protoporphyrinogen IX oxidase%2C aerobic;Protoporphyrinogen oxidase | hemY | 1,886E+09 | 5,771E+08 |
| **A0A0D6HDQ3** | Sugar ABC transporter ATPase;Teichoic acid ABC transporter ATP-binding protein;ABC transporter, ATP-binding protein | tagH_2;QU38_07210 | 8,909E+09 | 1,299E+09 |
| **A0A0D6HFK0** | Adenine methyltransferase;Adenine-specific DNA methylase-like protein | AFO87_07160;QU38_08375 | 1,057E+09 | 6,411E+08 |
| **A0A0D6HHK7** | Luciferase family monooxygenase;Uncharacterized protein | limB_2;CH51_08785 | 2,169E+09 | 9,172E+08 |
| **A0A0D6HHV2** | Uncharacterised protein;Uncharacterized protein | AFO87_12060;QU38_08570;HMPREF0776_2710 | 6,298E+08 | 2,913E+08 |
| **A0A0D6HHY3** | Bifunctional oligoribonuclease and PAP phosphatase nrnA;DHHA1 domain protein;Exopolyphosphatase-like protein | nrnA;HMPREF0776_2736 | 6,134E+08 | 4,040E+08 |
| **A0A0D6HIY4** | DNA repair protein RecO | recO | 9,895E+07 | 3,083E+07 |
| **A0A0D6HM65** | Aldo/keto reductase;Uncharacterized protein;Similar to oxidoreductase;Aldo/keto reductase family protein | yhdN;SA1331 | 3,213E+09 | 1,042E+09 |
| **A0A0D6HT57** | Glycerophosphodiester phosphodiesterase;Glycerophosphoryl diester phosphodiesterase | ugpQ_3;ST398NM01_1117 | 4,036E+09 | 1,518E+09 |
| **A0A0D6W9J7** | Oligopeptide ABC transporter permease OppC;ABC transporter, permease protein;OppC | oppC_1;HMPREF0776_1976 | 6,706E+09 | 6,660E+07 |
| **A0A0D6WD10** | Acetyltransferase | paiA | 5,317E+07 | 4,598E+07 |
| **A0A0D6WGH1** | Lipoprotein;Uncharacterized protein;Putative lipoprotein | AM595_02130;ACR58_05445;MW0354 | 8,008E+09 | 3,532E+09 |
| **A0A0E0VMZ2** | L-threonine 3-dehydrogenase;Uncharacterized epimerase/dehydratase SAOUHSC_00535 | ST398NM01_0628;SAOUHSC_00535 | 1,135E+09 | 3,233E+08 |
| **A0A0E0VN55** | Uncharacterized protein;Nucleoside permease | ST398NM01_0596;ACH32_09900 | 2,328E+10 | 5,199E+08 |
| **A0A0E0VNH8** | Threonine/Serine Exporter;Integral membrane protein;Putative membrane protein | ST398NM01_0821;ACH32_11070;yjjP | 5,423E+08 | 1,322E+08 |
| **A0A0E0VPM3** | Uncharacterized protein;Chitinase | ST398NM01_1100;ACR58_03410;HMPRE | 7,145E+09 | 5,275E+09 |
| **A0A0E0VPZ2** | Large-conductance mechanosensitive channel | mscL | 7,609E+10 | 1,106E+10 |
| **A0A0E0VQW3** | KdpE;DNA-binding response regulator KdpE;PhoB family transcriptional regulator;Response regulator | ST398NM01_2121;kdpE | 1,955E+09 | 1,329E+09 |
| **A0A0E0VR77** | Uncharacterized protein;Membrane protein | ST398NM01_2049;ACR58_12545;BN1321_320021 | 9,492E+09 | 3,081E+09 |
| **A0A0E0VRI2** | Sodium/proline symporter | ST398NM01_1996;putP | 4,133E+09 | 4,828E+07 |
| **A0A0E0VRI6** | Molybdopterin-guanine dinucleotide biosynthesis protein B;Putative molybdopterin-guanine dinucleotide biosynthesis protein B | ST398NM01_2323;mob | 7,978E+07 | 4,834E+07 |
| **A0A0E0VRM0** | Uncharacterized protein;Exported protein | ST398NM01_2438;ACH32_05335;ERS179246_01371 | 3,832E+09 | 1,323E+09 |
| **A0A0E0VRN4** | UPF0354 protein ST398NM01_1796 | ST398NM01_1796 | 1,232E+09 | 1,062E+09 |
| **A0A0E0VTG0** | Uncharacterized protein;ATPase 1-like protein | ST398NM01_2376;ACH32_05015;MW2246 | 7,684E+08 | 6,636E+08 |
| **A0A0E1ADE1** | Tandem lipoprotein within Pathogenicity island;Uncharacterized lipoprotein SACOL0486 | SAZ172_0446;SACOL0486 | 1,579E+10 | 1,045E+09 |
| **A0A0E1ADP9** | N-acetyl-L,L-diaminopimelate deacetylase;Putative N-acyl-L-amino acid amidohydrolase | SAZ172_0551;yxeP_1 | 1,023E+09 | 3,602E+08 |
| **A0A0E1ADX5** | Pili retraction protein pilT;Uncharacterized protein;Membrane protein | SAZ172_0529;SAOUHSC_00508;pilT | 1,013E+10 | 9,920E+08 |
| **A0A0E1AFC3** | ;Putative 2-succinyl-6-hydroxy-2,4-cyclohexadiene-1-carboxylate synthase | SAZ172_0985;menH | 1,238E+08 | 1,118E+08 |
| **A0A0E1AGA5** | Lysophospholipase;Hydrolase;Hydrolase, alpha/beta domain protein | SAZ172_1305;CH51_06690;HMPREF0776_2303 | 2,610E+09 | 1,199E+09 |
| **A0A0E1AGR4** | Uncharacterized protein;CBS domain protein | SAZ172_1577;ccpN | 6,303E+08 | 2,591E+08 |
| **A0A0E1AGR7** | Oligopeptide transport system permease protein OppB;Oligopeptide transport system permease protein oppB | oppB;SAKOR_00903 | 6,889E+09 | 9,760E+07 |
| **A0A0E1AIA2** | Magnesium and cobalt efflux protein CorC;Hemolysin-like protein containing CBS domains;Putative transporter or sensor | SAZ172_0713;ytfL | 4,277E+09 | 6,684E+08 |
| **A0A0E1AIR4** | Cell surface hydrolase (Putative);Alpha/beta hydrolase | SAZ172_2292;AFO87_08115;QU38_03685 | 3,854E+09 | 1,573E+08 |
| **A0A0E1AJB4** | Uncharacterized protein;NTPase | SAZ172_1821;AL493_11020;NWMN_1698 | 4,226E+08 | 7,172E+07 |
| **A0A0E1AJK7** | Phosphoglucosamine mutase / Phosphomannomutase;Phosphoglucomutase | SAZ172_2588;pgcA | 9,486E+08 | 4,955E+08 |
| **A0A0E1AK20** | Two-component system histidine kinase;Histidine kinase | SAZ172_2743;graS;nsaS | 1,303E+09 | 1,241E+08 |
| **A0A0E1AKG4** | Chromosome partition protein Smc | smc | 3,063E+09 | 1,325E+09 |
| **A0A0E1AL79** | Uncharacterized protein;Putative cytosolic protein;Lipoprotein | SAZ172_1503;ST398NM01_1556;AFO87_01655 | 9,234E+09 | 9,836E+08 |
| **A0A0E1ALA4** | L-Cystine ABC transporter, permease protein TcyB;Amino acid ABC transporter permease | SAZ172_2517;QU38_07805 | 1,004E+10 | 5,928E+09 |
| **A0A0E1ALV6** | Phosphate regulon sensor protein PhoR (SphS);Histidine kinase-, DNA gyrase B-, and HSP90-like ATPase domain protein | phoR;SAOUHSC_01799 | 9,516E+08 | 6,246E+08 |
| **A0A0E1ANC6** | Uncharacterized protein;Integral membrane protein | SAZ172_2445;yhaI;MW2263 | 2,000E+09 | 2,242E+08 |
| **A0A0E1AND1** | Magnesium transport protein CorA | corA;corA_2 | 1,793E+09 | 1,111E+08 |
| **A0A0E1APE5** | Transport ATP-binding protein CydD;ABC transporter ATPase/permease;MW2352 protein | SAZ172_2534;irtA;MW2352 | 3,181E+08 | 5,390E+07 |
| **A0A0E1APK1** | Uncharacterized protein;Uncharacterized lipoprotein SAUSA300_2430;Tandem lipoprotein | SAZ172_2584;SAUSA300_2430 | 1,239E+09 | 5,474E+07 |
| **A0A0E1VHL5** | SWIM zinc finger domain protein;SWIM zinc finger domain-containing protein | HMPREF0776_2395;AFO87_04285 | 3,307E+08 | 1,897E+08 |
| **A0A0E1VHW9** | Peptidase M16 inactive domain protein;Non-proteolytic protein, peptidase family M16 | HMPREF0776_2279;SAKOR_01206 | 5,041E+08 | 3,138E+08 |
| **A0A0E1VIN0** | Magnesium transporter MgtE | mgtE;MW0890; | 4,642E+09 | 4,485E+08 |
| **A0A0E1VJG8** | Ribonucleoside-diphosphate reductase | HMPREF0776_1739;nrdE | 3,897E+10 | 2,669E+10 |
| **A0A0E1VK03** | ACT domain protein;ACT domain-containing protein;Protein with ACT domain | HMPREF0776_2282;ACH32_13365;BN1326_60 | 9,191E+08 | 5,329E+08 |
| **A0A0E1VK25** | GTPase HflX | hflX | 4,069E+08 | 7,673E+07 |
| **A0A0E1VK72** | Glyoxalase family protein (Fragment);Uncharacterized protein | HMPREF0776_1493;MW1077 | 1,364E+08 | 6,966E+07 |
| **A0A0E1VKC4** | Cardiolipin synthase | HMPREF0776_1457;cls;QU38_06435 | 8,928E+09 | 2,253E+09 |
| **A0A0E1VKT7** | Periplasmic binding protein | HMPREF0776_2026 | 2,269E+09 | 1,721E+08 |
| **A0A0E1VKX0** | Amidophosphoribosyltransferase | purF | 4,728E+08 | 2,857E+08 |
| **A0A0E1VLN4** | HD domain protein;Uncharacterized protein;HD domain-containing protein | HMPREF0776_1767;BN1321_170090;ACR58_048 | 2,818E+09 | 1,528E+09 |
| **A0A0E1VM10** | CobW/P47K family protein;Cobalamin biosynthesis protein CobW;MW0649 protein | HMPREF0776_1691;yjiA_1;MW0649 | 3,565E+08 | 1,289E+08 |
| **A0A0E1VM29** | Response regulator SaeR | saeR | 3,163E+09 | 3,012E+09 |
| **A0A0E1VMU0** | Ribosomal RNA small subunit methyltransferase I | rsmI | 2,094E+08 | 6,422E+07 |
| **A0A0E1VN29** | ABC transporter, substrate-binding protein, QAT family;Glycine/betaine ABC transporter substrate-binding protein | HMPREF0776_0506;opuCC_2 | 1,208E+10 | 1,240E+09 |
| **A0A0E1VNG3** | Luciferase-like monooxygenase;Luciferase | HMPREF0776_1083;luxA;RU53_ | 6,171E+08 | 3,382E+08 |
| **A0A0E1VP82** | Uncharacterized protein;Exported protein | HMPREF0776_0088;SAZ172_1939;AFO87_13215 | 2,660E+09 | 1,015E+08 |
| **A0A0E1VQD5** | Hydrolase, alpha/beta domain protein;Alpha/beta hydrolase domain-containing protein;Uncharacterized protein OS | HMPREF0776_2798;ACH32_01680 | 3,056E+09 | 2,991E+09 |
| **A0A0E1VQF1** | Uncharacterized protein;Membrane protein | HMPREF0776_2813;AFO87_10145;QU38_04760 | 4,077E+09 | 9,059E+08 |
| **A0A0E1VSQ2** | Putative phage head-tail adaptor;Oligopeptide ABC transporter%2C periplasmic oligopeptide-binding protein oppA | HMPREF0776_1979;oppA_1 | 9,855E+09 | 7,592E+08 |
| **A0A0E1VW39** | Sensor protein LytS;Autolysis histidine kinase LytS | lytS | 2,132E+09 | 4,933E+08 |
| **A0A0E1VWL4** | Transporter, lactate permease (LctP) family;L-lactate permease | HMPREF0776_0843;lctP_1;lctP | 2,911E+10 | 9,731E+08 |
| **A0A0E1VXG7** | Uncharacterized protein;Membrane protein;ABC transporter ATP-binding protein | HMPREF0776_0572;AFO87_06210;QU38_ | 2,166E+08 | 1,872E+07 |
| **A0A0E1X4S8** | TIGR00370 family protein;Uncharacterized protein;Allophanate hydrolase | HMPREF0769_12070;SAOUHSC_01712;kipI_2 | 2,001E+08 | 1,090E+08 |
| **A0A0E1X4Z9** | KH domain protein;Phosphate starvation-inducible protein PhoH, predicted ATPase;Phosphate starvation protein PhoH | HMPREF0769_12108;SAZ172_1584 | 3,592E+09 | 1,923E+09 |
| **A0A0E1X623** | MIP family channel protein;Aquaporin;Glycerol permease | HMPREF0769_12480;glpF | 7,359E+09 | 3,805E+08 |
| **A0A0E1X6Q9** | Phosphoribosylglycinamide formyltransferase | purN | 2,451E+08 | 1,848E+07 |
| **A0A0E1X849** | Uncharacterized protein;Threonine serine exporter;Putative membrane protein | HMPREF0769_11747;ACH32_10230;BN1326_50069 | 1,217E+09 | 5,925E+07 |
| **A0A0E1X887** | DNA repair protein radA | radA;AFO87_13065;ACH32_09930 | 1,587E+09 | 5,382E+08 |
| **A0A0E1XBY7** | HAD hydrolase, family IA, variant 3;HAD family hydrolase;HAD hydrolase, IA, variant 1 family protein | HMPREF0769_10834;ACR58_07965 | 9,031E+08 | 8,713E+08 |
| **A0A0E1XHC1** | Adenylate cyclase | HMPREF0769_12782;HMPREF0776_1992;ACR58 | 1,545E+09 | 5,010E+08 |
| **A0A0E1XM44** | Inositol monophosphatase family protein;Inositol monophosphatase | HMPREF0769_10843;suhB_1 | 3,322E+08 | 1,954E+08 |
| **A0A0E7MS50** | Putative DUF86-containing protein;Uncharacterized protein | yutE;SAOUHSC_00864;QU38_11250 | 1,475E+09 | 1,065E+09 |
| **A0A0E7X8W5** | D-3-phosphoglycerate dehydrogenase | serA | 6,903E+09 | 1,996E+09 |
| **A0A0E7XUD2** | 3-beta hydroxysteroid dehydrogenase;Uncharacterized protein;SA0317 protein | AFO87_05130;SAOUHSC_00309;SA0317 | 3,275E+09 | 9,215E+08 |
| **A0A0E7YL28** | Amidohydrolase;HmrA protein | hmrA;HMPREF0769_11024 | 6,640E+08 | 4,508E+08 |
| **A0A0E8G7K7** | Isochorismate synthase | entC;QU38_05455 | 6,492E+08 | 2,298E+08 |
| **A0A0E8G8K9** | D-alanyl-lipoteichoic acid biosynthesis protein;D-alanyl-lipoteichoic acid biosynthesis protein DltD;Poly D-alanine transfer protein | dltD | 2,089E+10 | 4,142E+09 |
| **A0A0E8G9T5** | Di/tripeptide permease YjdL;Di-/tripeptide transporter;Di-tripeptide transporter | yjdL;ST398NM01_0804;OO23_0206745 | 5,915E+09 | 4,553E+08 |
| **A0A0E8GA15** | Autolysin;Bifunctional autolysin | atl_1;atl | 1,185E+11 | 9,140E+10 |
| **A0A0E8GDF5** | Phosphomevalonate | mvaK2;ACH32_10260 | 3,639E+08 | 1,321E+08 |
| **A0A0E8GDN8** | ABC transporter;ABC transporter, ATP-binding protein;ABC transporter ATP-binding protein uup | yjjK;SAUSA300_0704 | 3,276E+09 | 1,036E+09 |
| **A0A0E8GE10** | PepSY-associated TM helix family protein;Uncharacterized protein | AL498_04090;NWMN_1352;SA1275 | 4,751E+08 | 3,853E+07 |
| **A0A0E8GF34** | Pahtogenicity island protein | AFO87_10795;ERS179246_01627 | 1,568E+09 | 1,310E+09 |
| **A0A0E8HH43** | Small heat shock protein;Crystallin;Small heat shock protein Hsp20;Heat shock protein | ERS092844_01933;CH51_12820;NWMN_2287 | 1,538E+08 | 1,056E+08 |
| **A0A0E8HHC4** | Nitrite reductase | nasD | 6,751E+07 | 2,432E+07 |
| **A0A0E8HIR1** | Drug resistance transporter;Putative transport protein;MW2273 protein | emrB_1;emrB_2 | 9,087E+08 | 1,204E+08 |
| **A0A0E8I3P7** | Fructose phosphotransferase system enzyme fruA homolog;PTS system, fructose-specific IIABC component, putative | manP;SAOUHSC_02975 | 1,324E+10 | 2,239E+09 |

| | | | | |
|---|---|---|---|---|
| **A0A0E8IS76** | Succinyl-diaminopimelate desuccinylase;Uncharacterized protein | dapE;NWMN_1929 | 8,343E+08 | 2,304E+08 |
| **A0A0E8J639** | UDP-N-acetylmuramoyl-tripeptide--D-alanyl-D-alanine ligase;UDP-N-acetylmuramoyl-tripeptide--D-a | murF | 1,107E+09 | 6,111E+08 |
| **A0A0E8J986** | ATPase;Superfamily I DNA/RNA helicase protein;MW0068 protein;SA0089 protein | AFO87_03745;ERS093009_00884;MW0068 | 1,691E+08 | 1,274E+08 |
| **A0A0G2LPA2** | Membrane protein;Lipase family protein;Putative cytosolic protein | CH51_02265;AFO87_01950;ERS093009_02540 | 1,287E+09 | 2,245E+08 |
| **A0A0G2LQC9** | Central glycolytic genes regulator;Central glycolytic gene regulator;Putative sugar-binding domain protein | CH51_04035;ACH32_11220 | 7,121E+08 | 4,522E+08 |
| **A0A0G2LTJ0** | Octanoyltransferase;Octanoyltransferase LipM | CH51_08340;lipM | 4,112E+09 | 1,382E+09 |
| **A0A0H2BKJ8** | Ribosome-binding ATPase YchF | ychF;engD | 1,670E+10 | 6,697E+09 |
| **A0A0H2CCY2** | Glycine betaine transport ATP-binding protein;Glycine betaine/carnitine/choline ABC transporter | proV_2;opuCA | 3,159E+09 | 3,080E+09 |
| **A0A0H2GUK8** | LysR family regulatory protein;Transcriptional regulator, LysR family | AL077_11715;HMPREF0776_1964 | 1,631E+08 | 7,098E+07 |
| **A0A0H2IDV7** | Conserved cytoplasmic protein;Uncharacterized protein | AFO87_10570;SA0738 | 7,958E+07 | 5,300E+07 |
| **A0A0H2JAS5** | ATPase;Phage membrane protein;Phi PVL ORF 30-like protein | AFO87_01640;AL077_01570;BN1326_80029 | 2,266E+10 | 2,976E+08 |
| **A0A0H2WWQ7** | Uncharacterized protein;Uncharacterised protein | SACOL2204;AFO87_12745;SAOUHSC_02474 | 1,191E+09 | 6,193E+08 |
| **A0A0H2WXL1** | Aminotransferase, putative;Aspartate aminotransferase | SACOL2000;CH51_10535;aspC | 2,389E+09 | 1,016E+09 |
| **A0A0H2X055** | Uncharacterized protein;Uncharacterised protein | SACOL0267;AFO87_12600;ST398NM01_0295 | 2,580E+10 | 7,266E+09 |
| **A0A0H2XHM0** | Capsular polysaccharide biosynthesis protein Cap5I;Glycosyltransferase;Uncharacterized protein | cap5I;SAKOR_00137 | 2,969E+08 | 1,722E+08 |
| **A0A0H2XIZ6** | Antibiotic transport-associated protein-like protein;Membrane protein;Uncharacterized protein | SAUSA300_2489;mmpL8;SAO | 1,484E+09 | 7,605E+07 |
| **A0A0H2XKA3** | Accessory gene regulator protein C;Histidine kinase | agrC;SAOUHSC_02264 | 5,702E+09 | 5,225E+08 |
| **A0A0H3JKG5** | Branched-chain amino acid transport system carrier protein | SA0180;SAKOR_00166 | 2,896E+09 | 3,925E+07 |
| **A0A0H3JKT9** | Uncharacterized protein;Orf2 | SA0078;NWMN_0024;AL493_01725 | 1,108E+09 | 4,807E+08 |
| **A0A0H3JLP7** | Uncharacterized protein;Thiamine diphosphokinase | SA1066;HMPREF0776_2222 | 2,070E+08 | 2,053E+08 |
| **A0A0H3JMT9** | Uncharacterized protein;tRNA methylthiotransferase YqeV;30S ribosomal protein S12 methylthiotransferase | SA1405;yqeV | 6,310E+08 | 4,560E+08 |
| **A0A0H3JNF8** | RGD-containing lipoprotein;Putative lipoprotein;Uncharacterized protein | rlp;SAUSA300_0203;HMPR | 3,340E+09 | 6,616E+07 |
| **A0A0H3JP15** | Uncharacterized protein;Efflux transporter | SA0601;yxkD | 1,190E+09 | 1,238E+08 |
| **A0A0H3JSJ5** | Uncharacterized protein;Putative cytosolic protein | SA0170;AFO87_11960;SAZ172_0185 | 4,382E+08 | 4,305E+08 |
| **A0A0H3JV05** | Fructose specific permease;PTS fructose transporter subunit IIC | fruA | 3,442E+10 | 2,279E+09 |
| **A0A0H3JV29** | Transcription activator of glutamate synthase operon | gltC | 5,350E+08 | 1,128E+08 |
| **A0A0H3JVF9** | MW0891 protein;TrkA C-terminal domain protein;Monovalent cation:H+ antiporter-2 | MW0891;HMPREF0769_12776;ACR | 3,424E+09 | 4,821E+07 |
| **A0A0H3JVM5** | Uncharacterized protein;Fe-S cluster assembly protein SufD | MW0796;SA0775;ACH32_11395 | 3,484E+09 | 3,159E+09 |
| **A0A0H3JW61** | Uncharacterized protein;Acetyltransferase%2C GNAT family;GNAT family acetyltransferase | SA2355;AFO87_06865;QU38_16295 | 5,787E+08 | 4,086E+08 |
| **A0A0H3JWI6** | 5-formyltetrahydrofolate cyclo-ligase | MW1502;SAZ172_1563 | 2,245E+08 | 2,022E+08 |
| **A0A0H3JWW2** | MW0990 protein;Inositol monophosphatase;SA0958 protein | MW0990;suhB_1;SA0958 | 1,748E+09 | 4,558E+08 |
| **A0A0H3JX27** | MW1823 protein;Ribonuclease BN;Similar to transporter | MW1823;yihY;SAV1883 | 2,065E+10 | 1,333E+09 |
| **A0A0H3JXA7** | Uncharacterized protein | MW1298;SA1240;HMPREF0776_ | 4,278E+08 | 2,008E+08 |
| **A0A0H3JY62** | MW1802 protein;SA1679 protein;D-isomer specific 2-hydroxyacid dehydrogenase family protein | MW1802;SA1679;SACOL19 | 1,018E+08 | 9,902E+07 |
| **A0A0H3JYG8** | MW2545 protein;Response regulator receiver domain protein;Two-component response regulator YvcP | MW2545;HMPREF0769_10522 | 1,216E+08 | 9,347E+07 |
| **A0A0H3JYP5** | Acetate-CoA ligase;Acyl--CoA ligase;AMP-dependent synthetase and ligase | MW2528;ACR58_09690;acsA2 | 2,981E+09 | 1,908E+09 |
| **A0A0H3JYT9** | Cystathionine gamma-synthase | metB | 2,635E+08 | 5,451E+07 |
| **A0A0H3JYY8** | MW0527 protein;HAD family hydrolase;Uncharacterized protein | MW0527;gph;NWMN_0535 | 2,649E+08 | 1,011E+08 |
| **A0A0H3JZN6** | MW0933 protein;Aminotransferase, class I/II;Aminotransferase, class I | MW0933;HMPREF0769_12733;patA | 1,446E+09 | 5,712E+08 |
| **A0A0H3K0H4** | Chromosome replication initiation/membrane attachment protein | dnaB;HMPREF077 | 1,211E+09 | 7,832E+08 |
| **A0A0H3K3C3** | MW1508 protein;ABC transporter ATP-binding protein;Zinc ABC transporter | MW1508;mreA;znuC | 1,262E+09 | 8,608E+08 |
| **A0A0H3K4A0** | MW2249 protein;NAD(P)-dependent oxidoreductase;Oxidoreductase | MW2249;yghA;RU53_2388 | 1,377E+09 | 7,075E+08 |
| **A0A0H3K687** | Uncharacterized protein;Non-ribosomal peptide synthetase;Aureusimine non-ribosomal peptide synthetase | NWMN_0123;grsB | 1,930E+09 | 3,902E+08 |
| **A0A0H3K8T7** | Propeptide, PepSY amd peptidase M4;Uncharacterized protein;Putative secreted propeptide peptidase | NWMN_1639;MW1689 | 8,647E+09 | 6,855E+08 |
| **A0A0H3K9Q7** | Uncharacterized protein;Membrane protein;Uncharacterized protein OS | NWMN_1977;MW1996;ORF4 | 6,708E+08 | 3,866E+08 |
| **A0A0H3KA27** | Uncharacterized protein;MW0149 protein;SA0169 protein | NWMN_0119;MW0149;SA0169 | 9,511E+09 | 4,724E+09 |
| **A0A0H3KAC4** | Uncharacterized protein;Uncharacterised protein | NWMN_0226;HMPREF0776_1024;AFO87_0534 | 1,343E+08 | 8,384E+07 |
| **A0A0H3KAJ7** | Uncharacterized protein;Integral membrane protein | NWMN_2407;HMPREF0776_0567 | 1,042E+09 | 5,615E+07 |
| **A0A0H3KBU5** | Oligopeptide ABC transporter, ATP-binding protein;Oligopeptide transport ATP-binding protein oppF;ABC transporter, ATP-binding p | oppF;oppF_3 | 5,462E+09 | 4,757E+09 |
| **A0A0H3KDB3** | Aminotransferase, class V;Uncharacterized protein;Aminotransferase | NWMN_1610;SAOUHSC_01825;iscS_1 | 2,265E+08 | 7,630E+07 |
| **A0A0J7T0R5** | Flavin reductase;FAD-dependent oxidoreductase;Putative flavin reductase | AL493_01015;SAKOR_02656;AFO87_04150 | 4,522E+08 | 2,527E+08 |
| **A0A0J7WVX5** | Dynamin family protein;GTPases (Dynamin-related);Dynamin family protein | AL493_08785;ERS411017_01529;ERS093009_01170 | 4,563E+09 | 2,186E+09 |
| **A0A0K6TZ04** | Oligopeptide transport ATP-binding protein oppD;Oligopeptide transport ATP-binding protein OppD | oppD_2;oppD | 4,603E+09 | 4,326E+09 |
| **A0A0K6Z0X1** | LysR family regulatory protein CidR;LysR family regulatory protein;Transcriptional regulator, LysR family, putative | cidR | 2,541E+08 | 9,054E+07 |
| **A0A0K7F311** | Integral membrane protein;Membrane protein;Bacterial membrane protein YfhO OS | ERS365775_00155;CH51_06385;AFO87_09375 | 1,091E+09 | 3,263E+08 |
| **A0A0K7KAG9** | Putative polyribitolphosphotransferase;TagB protein, putative | tagF_1;tagF_3;SAOUHSC_00222 | 2,710E+09 | 1,294E+09 |
| **A0A0K7VUK1** | Alpha-acetolactate decarboxylase | aldC_2;aldB | 2,021E+08 | 8,033E+07 |
| **A0A0K8EWW4** | Alpha/beta hydrolase;Uncharacterized protein | ydeN;ACH32_02060;NWMN_1722 | 5,835E+09 | 1,388E+09 |
| **A0A0M1TWI2** | Uncharacterized protein;Membrane protein | RU53_110;AFO87_03905;SA0121 | 2,699E+09 | 5,641E+08 |
| **A0A0M1TWW5** | Iron (Fe3+) ABC superfamily ATP binding cassette transporter, binding protein;Iron ABC transporter substrate-binding protein | sirA;ACR58_06845 | 9,139E+09 | 1,774E+08 |
| **A0A0M1TXC0** | Lipoprotein | RU53_427;MW0418;metQ_2;met | 6,213E+10 | 2,091E+09 |
| **A0A0M1TXC5** | Putative antitoxin YezG;QueA protein;Uncharacterized protein | yezG_2;AL493_02910;SAOUHSC_00275 | 2,751E+08 | 1,613E+08 |
| **A0A0M1TXR4** | Nucleoside triphosphate pyrophosphohydrolase | mazG_1;mazG | 1,209E+09 | 6,030E+08 |
| **A0A0M1TY42** | Putative permease;ABC transporter permease;Bacitracin ABC transporter permease | RU53_724;vraG | 1,214E+09 | 3,900E+08 |
| **A0A0M1TY86** | Uncharacterized protein;Putative lipoprotein;Un | RU53_873;SACOL0851;AFO87_10560 | 4,836E+10 | 2,303E+09 |
| **A0A0M1TYC5** | Putative Mg2+ and Co2+ transporter, CorB;CBS domain protein;CorC | RU53_915;SACOL0921;ST398NM0 | 5,539E+08 | 2,161E+08 |
| **A0A0M1TYD0** | ABC superfamily ATP binding cassette transporter, ABC protein;Manganese transport system ATP-binding protein | mntA;ACR58_11615 | 2,050E+10 | 1,513E+10 |
| **A0A0M1TZ07** | Teichoic acid biosynthesis protein B;Teichoic acid biosynthesis protein | tagB | 3,051E+08 | 1,882E+08 |
| **A0A0M1TZ20** | Alpha/beta hydrolase;Lipase;Lipase LipA;Similar to lipase LipA | RU53_717;lipA_1;QU38_10295 | 3,699E+09 | 1,100E+08 |
| **A0A0M1U010** | ABC superfamily ATP binding cassette transporter, ABC protein;ABC transporter, ATP-binding protein | ykoD;HMPREF0776_2068 | 4,621E+08 | 1,639E+08 |
| **A0A0M1U044** | Putative competence-damage inducible protein | cinA | 1,253E+09 | 5,411E+08 |
| **A0A0M1U069** | Exonuclease;5-3 exonuclease;DNA polymerase I | RU53_1467;ST398NM01_1439;ypcP | 1,580E+09 | 2,784E+08 |
| **A0A0M1U097** | Putative succinate dehydrogenase flavoprotein subunit;SdhA protein;Succinate dehydrogenase flavoprotein subunit | sdhA | 8,521E+10 | 4,240E+10 |
| **A0A0M1U0K9** | Alpha/beta hydrolase fold-3 domain-containing protein;Probable L-asparaginase;L-asparaginase | ansA | 1,113E+09 | 6,595E+08 |
| **A0A0M1U0L1** | Putative geranyltranstransferase;Polyprenyl synthetase;Geranyl transferase | ispA;HMPREF0776_2557;i | 3,870E+08 | 2,288E+08 |
| **A0A0M1U118** | Uncharacterized protein;Lipoprotein | RU53_1630;ST398NM01_1596;ACH32_ | 8,428E+09 | 4,926E+08 |
| **A0A0M1U143** | Uroporphyrinogen decarboxylase | hemE | 4,329E+08 | 2,292E+08 |
| **A0A0M1U1F3** | ABC superfamily ATP binding cassette transporter, ABC protein;ABC transporter ATP-binding protein;ABC transporter, ATP-binding p | RU53_2071;drrA | 2,141E+09 | 4,090E+08 |
| **A0A0M1U2H4** | Formate/nitrite transporter family protein;SA2191 protein;Formate/nitrite transporter | RU53_2463;SA2191;nirC | 8,767E+08 | 2,242E+08 |
| **A0A0M1U2N6** | Putative lipoprotein YehR;Uncharacterized protein;Lipoprotein | yehR;HMPREF0776_0518; | 2,683E+10 | 1,653E+09 |
| **A0A0M1U330** | Glycerophosphotransferase, TarF;Uncharacterized protein | tarF_2;SAKOR_02344 | 5,115E+08 | 2,453E+08 |
| **A0A0M1U376** | Aminobenzoyl-glutamate transport protein | RU53_2538;abgT | 2,094E+09 | 5,468E+07 |
| **A0A0M1U3F4** | MerR family transcriptional regulator;Transcriptional regulator, MerR family | RU53_2570;zntR | 8,553E+08 | 1,329E+08 |
| **A0A0M1U3J5** | Molybdenum (Mo2+) ABC superfamily ATP binding cassette transporter, binding protein;Probable molybdate-binding protein | modA | 2,632E+10 | 7,283E+08 |
| **A0A0S2JAW7** | Trehalose operon repressor;GntR family transcriptional regulator;GntR | treR;treR_2 | 4,688E+07 | 4,313E+07 |
| **A0A0T7M338** | D-alanyl transfer protein DltB;Activated D-alanine transport protein;Putative D-alanine esterase for lipoteichoic acid and wall teichoi | dltB | 4,181E+08 | 1,768E+08 |
| **A0A0T7M3C5** | Enoyl-[acyl-carrier-protein] reductase [NADPH];Enoyl-[acyl-carrier-protein] reductase [NADPH] FabI | fabI | 7,148E+09 | 2,178E+09 |
| **A0A0T7M7V7** | Cation efflux family protein;MW0143 protein;SA0163 protein | czcD_1;MW0143;SA0163 | 6,368E+08 | 9,142E+07 |
| **A0A0T7M903** | Putative tRNA (cytidine(34)-2-O)-methyltransferase | trmL | 3,088E+08 | 2,347E+08 |
| **A0A0T7M913** | Trehalose-6-phosphate hydrolase;Glucohydrolase | treA;SAZ172_0476 | 1,430E+08 | 1,359E+08 |
| **A0A0T7M9C7** | Acetyl-coenzyme A synthetase;Acetyl-CoA synthetase;AMP-binding | acsA | 7,710E+09 | 2,357E+09 |
| **A0A0T7M9K4** | Telomeric repeat-binding factor 2 family protein;Uncharacterized protein | ERS093009_02688;HMPREF0769_11889 | 6,569E+09 | 3,650E+08 |
| **A0A0T7MRS9** | Iron-sulfur cluster assembly ATPase SufC;ATP-dependent transporter sufC;FeS assembly ATPase SufC | yurY;ACH32_11390;sufC | 7,714E+09 | 4,224E+09 |
| **A0A0T7MTX1** | Thioredoxin reductase;Putative FAD-dependent disulfide oxidoreductase | trxB_2;ypdA | 1,711E+08 | 1,277E+08 |
| **A0A0T7MUK3** | Na+/H+ antiporter;Na+/H+ antiporter, putative;Putative Na+/H+ antiporter | nhaK_2;SACOL2442;HMPREF0769_10706 | 6,238E+09 | 1,022E+09 |
| **A0A0T7MV30** | LytR family transcriptional regulator | lytR_1;ACH32_04930 | 1,728E+10 | 4,132E+09 |
| **A0A0T7MW09** | Putative polyribitolphosphotransferase;CDP-ribitol ribitolphosphotransferase | tagF_1;ACR58_06110;tag | 1,036E+10 | 6,148E+09 |
| **A0A0U0TSZ8** | Allophanate hydrolase subunit 2;Allophanate hydrolase;Uncharacterized protein | ERS195423_00878;AFO87_10985;SAOUHSC_01711 | 2,816E+08 | 7,492E+07 |
| **A0A0U1MF40** | Putative membrane protein;Membrane protein;Uncharacterized protein | BN1321_150055;CH51_03255;ACR58_11530 | 2,154E+09 | 3,579E+08 |
| **A0A0U1MF84** | Iron dependent repressor, N-terminal DNA binding domain protein;DtxR family transcriptional regulator | BN1321_150074;ACH32_10480 | 2,316E+09 | 2,263E+09 |
| **A0A0U1MFR0** | Putative beta-lactamase;Penicillin binding protein 4 | BN1321_150082;pbp4 | 3,939E+09 | 2,896E+08 |
| **A0A0U1MFY6** | DNA polymerase III (Gamma and tau subunits);DNA polymerase III gamma and tau subunits | dnaX | 2,816E+09 | 1,108E+09 |
| **A0A0U1MH00** | Bacterial regulatory helix-turn-helix , lysR family protein;LysR substrate binding domain protein | BN1321_170002;HMPREF0769_11660 | 7,708E+08 | 2,384E+08 |
| **A0A0U1MHE2** | Glycerate kinase family protein;SA0697 protein;Glycerate kinase | BN1321_170076;SA0697;glxK_1 | 6,231E+08 | 5,139E+08 |
| **A0A0U1MHP2** | 1,4-dihydroxy-2-naphthoate octaprenyltransferase | menA | 2,222E+09 | 1,363E+08 |
| **A0A0U1MIE4** | Putative ribonuclease with PIN and NYN domains;Uncharacterized protein;Putative cytosolic protein | yacP;HMPREF0769_11801; | 5,771E+08 | 3,439E+08 |
| **A0A0U1MJ68** | Putative membrane protein;Uncharacterized protein | BN1321_230002;SAUSA300_0980;HMPREF0769_127 | 2,437E+09 | 7,612E+08 |
| **A0A0U1MKY9** | Putative integral membrane protein;Putative membrane protein;Membrane protein | ypdP;HMPREF0769_12315;ACR58_14545 | 5,465E+08 | 7,011E+07 |
| **A0A0U1ML47** | Malate glycosyltransferase for bacillithiol synthesis;N-acetyl-alpha-D-glucosaminyl L-malate synthase;Glycosyltransferase | bshA;QU38_13715; | 2,315E+08 | 9,391E+07 |
| **A0A0U1MLC2** | tRNA N6-adenosine threonylcarbamoyltransferase | tsaD | 9,217E+08 | 4,485E+08 |
| **A0A0U1MM24** | Succinate dehydrogenase (Iron-sulfur protein);Succinate dehydrogenase iron-sulfur subunit | sdhB | 5,462E+10 | 2,272E+10 |
| **A0A0U1MM50** | Putative 5-bromo-4-chloroindolyl phosphate hydrolysis protein;5-bromo-4-chloroindolyl phosphate hydrolase;Uncharacterized prote | BN1326_60523;ACR58_01750 | 6,244E+08 | 2,583E+08 |
| **A0A0U1MM97** | Anaphase-promoting complex, cyclosome, subunit 3 family protein;Tetratricopeptide repeat protein | BN1321_260257;HMPREF0776_2657 | 7,769E+07 | 7,370E+07 |
| **A0A0U1MMF3** | ATP-dependent RecD-like DNA helicase | recD;recD_2 | 1,051E+09 | 6,279E+08 |
| **A0A0U1MMT8** | ATP synthase epsilon chain | atpC | 6,097E+08 | 2,547E+08 |
| **A0A0U1MMU7** | Acetate kinase | ackA | 4,769E+10 | 4,504E+10 |
| **A0A0U1MP49** | Alpha-acetolactate synthase;Acetolactate synthase, catabolic;Acetolactate synthase | alsS | 1,674E+09 | 6,720E+08 |
| **A0A0U1MP68** | Bacterial ABC transporter protein EcsB;EcsB | BN1321_290006;AFO87_07365;HMPREF0769_11843 | 2,669E+09 | 4,165E+08 |

| | | | | |
|---|---|---|---|---|
| **A0A0U1MQC8** | Autolysin;Mannosyl-glycoprotein endo-beta-N-acetylglucosaminidase | AUC48_09100;ACR58_09010;HMPREF0776 | 1,795E+10 | 1,198E+09 |
| **A0A0U1MR46** | Uncharacterized protein | BN1321_340076;MW2059;SACOL2127 | 2,960E+09 | 9,417E+08 |
| **A0A0U1MR56** | Uncharacterized protein;EVE domain protein | BN1321_350004;ERS093009_02338;AFO87_07675 | 3,702E+09 | 2,132E+09 |
| **A0A0U1MRF8** | Putative exported protein | BN1326_90074 | 5,664E+09 | 8,477E+08 |
| **A0A0U1MRQ5** | CAAX protease self-immunity family protein;CAAX amino terminal protease family protein;Uncharacterized protein | BN1321_380152;HMPREF0769_10841 | 1,557E+09 | 1,018E+08 |
| **A0A0U1MRS4** | Phosphotransferase system (PTS) maltose-specific enzyme IICB component;PTS system%2C arbutin-like IIBC component | malP;glvC | 1,139E+09 | 8,928E+07 |
| **A0A0U1MRW1** | ABC-2 transporter family protein;ABC superfamily ATP binding cassette transporter, membrane protein;Uncharacterized protein | BN1321_390008;RU53_2400 | 7,518E+09 | 1,816E+08 |
| **A0A0U1MS08** | L-lactate permease;MW2287 protein;SA2156 protein | lctP;MW2287;SA2156; | 1,374E+09 | 1,495E+08 |
| **A0A0U1MT23** | Ribosomal RNA methyltransferase FmrO domain protein;Uncharacterized protein | BN1321_410046;SA2257 | 3,041E+08 | 2,085E+08 |
| **A0A0U1MV17** | Homoserine O-acetyltransferase | BN1321_430102;metX | 6,642E+08 | 1,330E+08 |
| **A0A0U1MVJ1** | D-lactate dehydrogenase;MW2480 protein;SA2346 protein;D-specific D-2-hydroxyacid dehydrogenase-l | ldhD;MW2480;SA2346 | 1,505E+08 | 1,270E+08 |
| **A0A0U1MVR0** | ABC transporter, solute-binding protein | BN1321_60021;HMPREF0769_10274 | 1,537E+09 | 1,470E+08 |
| **A0A0U1MWK7** | Uncharacterized protein;Membrane protein;Isoprenylcysteine carboxyl methyltransferase family protein | BN1326_30078;ACH32_08335 | 1,126E+09 | 1,336E+08 |
| **A0A0U1MWT4** | Putative ribitol-5-phosphate dehydrogenase;SA0246 protein | tarJ;SA0246 | 8,116E+09 | 2,884E+09 |
| **A0A0U1MWX3** | Putative hemin import ATP-binding protein HrtA;Peptide ABC transporter ATP-binding protein;ABC transporter ATP-binding protein | hrtA;ACR58_05805 | 4,690E+08 | 3,789E+08 |
| **A0A0U1MXC0** | Putative phosphoglycerate mutase family protein;MW0351 protein;Phosphoglycerate mutase | BN1321_80100;MW0351;gpmA | 1,308E+08 | 4,085E+07 |
| **A0A0U1MXZ3** | Putative membrane protein;Uncharacterized protein;Membrane protein | BN1326_60124;BN1321_210016;ACR58_03900 | 1,007E+10 | 2,100E+08 |
| **A0A0U1MZF1** | Glycine betaine transporter;Glycine betaine transporter 1;Transporter, betaine/carnitine/choline family | opuD;NWMN_1261 | 9,106E+09 | 1,676E+08 |
| **A0A0U1MZP0** | Putative sulfur transferase;Putative rhodanese domain-containing sulfurtransferase | yqhL;ACH32_00510 | 1,297E+10 | 2,260E+09 |
| **A0A0U1N079** | NADPH-dependent aldo-keto reductase | yqkF | 1,127E+09 | 5,198E+08 |
| **A0A0U1N151** | Putative membrane protein;Uncharacterized protein;Membrane protein | BN1326_60502;RU53_1388;ACH32_13890 | 6,499E+09 | 4,861E+08 |
| **A0A0V1SBS2** | NADPH:quinone reductase;Putative quinone oxidoreductase YhfP | ACR61_14545;yhfP | 2,428E+09 | 1,093E+09 |
| **A0A0V1TBG6** | Beta-lactamase | ACR61_14555 | 2,466E+11 | 1,233E+11 |
| **A6QDH3** | PTS system glucose-specific EIICBA component;Phosphotransferase system (PTS) glucose-specific enzyme IICBA component | ptsG | 3,380E+10 | 3,930E+09 |
| **A6QDN3** | Iron-sulfur cluster repair protein ScdA | scdA | 7,922E+07 | 3,856E+07 |
| **A6QEJ1** | 50S ribosomal protein L10 | rplJ | 8,666E+09 | 3,136E+09 |
| **A6QGA1** | Aspartate carbamoyltransferase | pyrB | 1,792E+09 | 9,519E+08 |
| **A6QGC9** | Phosphate acyltransferase | plsX | 2,328E+09 | 1,250E+09 |
| **A6QGJ5** | DNA mismatch repair protein MutL | mutL | 2,036E+09 | 1,177E+09 |
| **A6QGP7** | Nuclease SbcCD subunit D;Exonuclease;DNA repair exonuclease | sbcD;CH51_07065 | 1,116E+08 | 4,257E+07 |
| **A6QH23** | Glycerol-3-phosphate dehydrogenase [NAD(P)+] | gpsA | 1,764E+09 | 7,261E+08 |
| **A6QH29** | Elastin-binding protein EbpS;Cell surface elastin binding protein | ebpS;ebp | 4,032E+10 | 2,620E+10 |
| **A6QHB3** | Endoribonuclease YbeY | ybeY;rnrY | 7,514E+08 | 2,318E+08 |
| **A6QHC2** | Chaperone protein DnaJ | dnaJ | 2,940E+09 | 2,016E+09 |
| **A6QHH3** | Histidine--tRNA ligase | hisS | 1,449E+09 | 1,032E+09 |
| **A6QHH7** | Adenine phosphoribosyltransferase;Adenine phos | apt | 8,093E+07 | 4,908E+07 |
| **A6QI49** | UPF0374 protein NWMN_1759;UPF0374 protein ygaC;UPF0374 protein ST398NM01_1960 | NWMN_1759;ygaC;ST398NM01_1960 | 4,781E+08 | 3,388E+08 |
| **A6QK99** | Oxygen-dependent choline dehydrogenase | betA | 8,742E+08 | 6,011E+08 |
| **A6QKD8** | Protein translocase subunit SecA 2;Protein translocase subunit SecA | secA2 | 5,732E+08 | 3,301E+08 |
| **B1Q017** | Gamma-hemolysin subunit A;Panton-Valentine leukocidin chain S | lukS-PV | 1,117E+09 | 4,419E+08 |
| **B6V384** | Lipoprotein;Uncharacterized protein | AL493_04610;QU38_07495;HMPREF0769_12466 | 1,273E+10 | 2,785E+08 |
| **D2J7P6** | Cadmium resistance protein;Cadmium transporter | cadD;ACR61_14535;SAKOR_02717 | 1,372E+09 | 2,735E+07 |
| **D2J801** | Beta-lactamase;Penicillin-binding protein, transpeptidase domain protein | blaR1;ACR61_14560;HMPR | 1,209E+09 | 4,265E+08 |
| **H6UH58** | Glucokinase;Glucose kinase | glcK | 4,373E+08 | 3,022E+08 |
| **O05154** | Putative glycosyltransferase TagX;Glycosyl transferase;Teichoic acid biosynthesis protein X | tagX;SAKOR_00634 | 1,227E+09 | 9,843E+08 |
| **O86491** | UDP-N-acetylmuramoyl-L-alanyl-D-glutamate--L-lysine ligase | murE | 3,652E+09 | 2,174E+09 |
| **P0A022** | DNA polymerase III subunit beta | dnaN;ST398NM01_0002 | 1,334E+10 | 7,478E+09 |
| **P0A092** | Nitric oxide synthase oxygenase | nos;SA | 1,131E+08 | 2,169E+07 |
| **P0A0H6** | Serine-protein kinase RsbW;Serine/threonine protein kinase | rsbW | 4,528E+09 | 2,156E+09 |
| **P0A0Q1** | Uncharacterized protein SAV1055;LytR family transcriptional regulator;Transcriptional regulator, LytR family protein | SAV1055;AL077_04510 | 2,641E+08 | 1,666E+08 |
| **P31337** | UPF0758 protein SAOUHSC_01763;UPF0758 protein MW1604;Uncharacterized protein | SAOUHSC_01763;MW1604;QU38_086 | 2,658E+08 | 1,856E+08 |
| **P52078** | Uncharacterized protein SAOUHSC_00997;Transcriptional regulator, LytR family protein;Uncharacterized protein | SAOUHSC_00997;SAKOR_00977 | 8,496E+09 | 5,237E+08 |
| **P60087** | Arginase | arg;QU38_06060;argI | 1,904E+09 | 5,679E+08 |
| **P60610** | Sensory transduction protein LytR;Two-component response regulator (LytS) | lytR;lytT | 2,540E+08 | 1,262E+08 |
| **P63757** | CDP-diacylglycerol--glycerol-3-phosphate 3-phosphatidyltransferase;CDP-diacylglycerol-glycerol-3-phosphate 3-phosphatidyltransfer | pgsA | 4,981E+09 | 4,504E+08 |
| **P63790** | ATP-dependent Clp protease ATP-binding subunit ClpX;ATP-dependent protease | clpX | 6,662E+09 | 4,312E+09 |
| **P64124** | Ferrochelatase;Ferrochelatase O | hemH | 3,463E+08 | 2,320E+08 |
| **P64235** | Methylenetetrahydrofolate--tRNA-(uracil-5-)-methyltransferase TrmFO | trmFO | 1,048E+09 | 4,657E+08 |
| **P65181** | 4-diphosphocytidyl-2-C-methyl-D-erythritol kinase | ispE | 6,111E+08 | 2,036E+08 |
| **P65228** | Homoserine kinase;Homoserine | thrB | 4,513E+08 | 9,188E+07 |
| **P65445** | Peptide methionine sulfoxide reductase MsrA 2;Peptide methionine sulfoxide | msrA2 | 2,478E+08 | 1,996E+08 |
| **P65475** | UDP-N-acetylmuramate--L-alanine ligase | murC | 3,846E+09 | 1,855E+09 |
| **P65694** | ATP-dependent 6-phosphofructokinase | pfkA | 4,184E+09 | 2,302E+09 |
| **P65937** | Uridylate kinase;Uridylate kinase OS | pyrH | 5,519E+09 | 2,131E+09 |
| **P65943** | Bifunctional protein PyrR | pyrR | 2,530E+09 | 1,556E+09 |
| **P66695** | Ribose-5-phosphate isomerase A | rpiA | 2,774E+08 | 2,581E+08 |
| **P66758** | Holliday junction ATP-dependent DNA helicase RuvB | ruvB | 3,134E+08 | 9,404E+07 |
| **P67062** | Demethylmenaquinone methyltransferase;Menaquinone biosynthesis methyltransferase, putative | menG;ubiF | 3,843E+09 | 2,035E+09 |
| **P67108** | Nucleotide-binding protein SAV0765;Nucleotide-binding protein HMPREF0769_11525;Nucleotide-binding protein SAZ | SAV0765;HMPREF0769_11525 | 4,033E+09 | 1,119E+09 |
| **P67277** | Ribonuclease Y | rny | 3,323E+10 | 6,827E+09 |
| **P67291** | UPF0154 protein SA1178;UPF0154 protein yneF;UPF0154 protein V070_00396;UPF0154 protein CO98_0281 OS | SA1178;yneF;V070_00396 | 2,446E+10 | 1,097E+09 |
| **P67410** | Uridine kinase | udk | 6,150E+08 | 2,679E+08 |
| **P68811** | Protein NrdI | nrdI | 2,051E+08 | 8,627E+07 |
| **P95695** | Capsular polysaccharide type 5 biosynthesis protein cap5A;Capsular polysaccharide type 8 biosynthesis protein cap8A | cap5A;cap8A | 9,518E+08 | 1,206E+08 |
| **P95696** | Cap5B;Capsular polysaccharide synthesis enzyme;Capsular polysaccharide synthesis enzyme Cap5B | cap5b;capB | 7,962E+07 | 4,169E+07 |
| **P95706** | Cap5M;Cap8M;Galactosyl transferase;Capsular biosynthesis protein | cap5M;cap8M;SAZ172_0171 | 2,305E+08 | 6,148E+07 |
| **P99151** | Alanine dehydrogenase 1;Alanine dehydrogenase | ald1 | 1,390E+07 | 9,611E+06 |
| **Q0H0G8** | Deoxyadenosine kinase;Deoxyguanosine kinase;SA0515 protein | dgk;QU38_09800 | 2,239E+08 | 5,715E+07 |
| **Q0H0H0** | Deoxyadenosine kinase;Deoxynucleoside kinase | dak;RU53_544;dck | 9,492E+08 | 3,860E+08 |
| **Q2FC27** | Beta-lactamase | blaZ;ACR62_14540 | 1,873E+09 | 9,638E+08 |
| **Q2FDV0** | Copper-exporting P-type ATPase A;Copper-translocating P-type ATPase | copA | 8,515E+09 | 2,407E+09 |
| **Q2FEA6** | Oxygen regulatory protein NreC;Two-component response regulator;LuxR family transcriptional regulator | nreC | 2,894E+08 | 1,372E+08 |
| **Q2FEC8** | Uncharacterized lipoprotein SAUSA300_2315;Uncharacterized protein | SAUSA300_2315;HMPREF0776_0422 | 4,226E+10 | 4,447E+09 |
| **Q2FED4** | Heme sensor protein HssS | hssS | 1,097E+09 | 9,959E+08 |
| **Q2FEG6** | Imidazolonepropionase | hutI | 1,128E+09 | 3,506E+08 |
| **Q2FEK0** | Urease accessory protein UreG | ureG;ure | 3,270E+08 | 2,773E+08 |
| **Q2FEQ6** | 30S ribosomal protein S5 | rpsE | 1,172E+10 | 8,082E+09 |
| **Q2FER7** | Energy-coupling factor transporter ATP-binding protein EcfA1 | ecfA1 | 1,367E+09 | 9,009E+08 |
| **Q2FER8** | Energy-coupling factor transporter ATP-binding protein EcfA2;Energy-coupling factor transporter ATP-binding protein EcfA | ecfA2;ecfA1_1 | 6,771E+08 | 1,890E+08 |
| **Q2FES9** | Uncharacterized hydrolase SAUSA300_2163;Uncharacterized hydrolase SA2005 | SAUSA300_2163;SA2005;ERS179246_00655 | 4,421E+08 | 3,455E+08 |
| **Q2FF06** | Putative aldehyde dehydrogenase SAUSA300_2076;Aldehyde dehydrogenase | SAUSA300_2076;ERS179246_00402 | 5,451E+09 | 2,532E+09 |
| **Q2FF13** | Low molecular weight protein-tyrosine-phosphatase PtpB;Low molecular weight protein tyrosine phosphatase | ptpB | 3,955E+08 | 1,770E+08 |
| **Q2FF20** | ATP synthase subunit b | atpF | 3,823E+10 | 3,129E+09 |
| **Q2FFI4** | UPF0316 protein SAUSA300_1892;UPF0316 protein SA1727;UPF0316 protein ST398NM01_2006 | SAUSA300_1892;SA1727 | 1,015E+10 | 2,658E+09 |
| **Q2FFQ5** | Foldase protein PrsA | prsA | 3,504E+10 | 3,511E+09 |
| **Q2FFU9** | 2-succinylbenzoate--CoA ligase | menE | 2,096E+08 | 1,116E+08 |
| **Q2FFZ9** | UPF0478 protein SAUSA300_1685 | SAUSA300_1685 | 2,830E+11 | 2,214E+10 |
| **Q2FG51** | Transcriptional repressor NrdR | nrdR | 5,018E+08 | 2,518E+08 |
| **Q2FG69** | Glutamate-1-semialdehyde 2,1-aminomutase 1 | hemL1 | 6,533E+08 | 5,538E+08 |
| **Q2FG97** | Aspartate--tRNA ligase | aspS | 2,733E+09 | 1,092E+09 |
| **Q2FGE1** | Heat-inducible transcription repressor HrcA;Heat-inducible transcription repressor | hrcA | 5,394E+08 | 9,991E+07 |
| **Q2FGG0** | Putative pyruvate, phosphate dikinase regulatory protein | SAUSA300_1523;SAV1563 | 3,269E+09 | 1,741E+09 |
| **Q2FGW7** | GTPase Der | der | 6,192E+09 | 2,538E+09 |
| **Q2FGX6** | 3-phosphoshikimate 1-carboxyvinyltransferase | aroA | 5,121E+08 | 2,194E+08 |
| **Q2FGY6** | Asparagine--tRNA ligase;Asparagine--tRNA ligase O | asnS | 2,953E+10 | 9,092E+09 |
| **Q2FH20** | UDP-N-acetylglucosamine--N-acetylmuramyl-(pentapeptide) pyrophosphoryl-undecaprenol N-acetylglucosamine transferase | murG | 6,677E+09 | 2,479E+09 |
| **Q2FH58** | Putative oligopeptide transport ATP-binding protein oppF2 | oppF2;opp-2F | 8,266E+07 | 4,265E+07 |
| **Q2FH78** | Protein GlcT;Transcriptional regulator | glcT;CH51_07120;QU38_13160 | 3,462E+08 | 2,711E+08 |
| **Q2FH88** | Nuclease SbcCD subunit C | sbcC | 1,300E+09 | 5,051E+08 |
| **Q2FHM9** | Guanylate kinase | gmk | 9,609E+08 | 8,026E+08 |
| **Q2FHQ4** | UDP-N-acetylmuramoylalanine--D-glutamate ligase | murD | 1,359E+09 | 7,313E+08 |
| **Q2FHT1** | Glutamate racemase | murI | 6,084E+09 | 2,284E+09 |
| **Q2FHU8** | High-affinity heme uptake system protein IsdE;Heme transporter IsdDEF, lipoprotein IsdE | isdE | 2,031E+09 | 4,048E+07 |
| **Q2FI05** | Bifunctional purine biosynthesis protein PurH | purH | 1,555E+09 | 6,268E+08 |
| **Q2FI17** | Probable quinol oxidase subunit 2;Quinol oxidase subunit 2;Cytochrome aa3-600 quinol oxidase (Subunit II) | qoxA;QU38_05550; | 9,226E+10 | 1,350E+10 |
| **Q2FI55** | Serine protease HtrA-like;Serine protease, DegP/HtrA, do-like protein | SAUSA300_0923;SACOL1028 | 7,147E+09 | 3,027E+09 |
| **Q2FIM4** | Epimerase family protein SAUSA300_0753;Cell division inhibitor | SAUSA300_0753;SAKOR_00773;CH51_ | 9,333E+09 | 2,906E+09 |
| **Q2FJ31** | Alcohol dehydrogenase;Zinc-binding alcohol dehydrogenase | adh;adh1;RU53_668 | 5,110E+08 | 5,006E+08 |

| | | | | |
|---|---|---|---|---|
| **Q2FJ92** | Elongation factor Tu | tuf;tufA | 4,920E+11 | 3,642E+11 |
| **Q2FJ93** | Elongation factor G | fusA;fus | 6,358E+10 | 5,740E+10 |
| **Q2FJC0** | Pyridoxal 5-phosphate synthase subunit PdxT | pdxT | 4,814E+08 | 3,956E+08 |
| **Q2FJH7** | N-acetylmuramoyl-L-alanine amidase sle1;Autolysin;N-acetylmuramoyl-L-alanine amidase | sle1;SAZ172_0466;amiD | 8,721E+08 | 7,037E+08 |
| **Q2FJI9** | UPF0753 protein SAUSA300_0426;UPF0753 protein SAOUHSC_00413;UPF0753 protein QU38_03380 | SAUSA300_0426;SAOUHSC_00413 | 2,766E+09 | 2,081E+09 |
| **Q2FK59** | Uncharacterized protein SAUSA300_0205;Uncharacterized protein SA0203;Uncharacterized protein SAV0210 | SAUSA300_0205;SA0203 | 1,022E+09 | 8,767E+07 |
| **Q2FKI2** | Probable copper-transporting P-type ATPase B;ATPase;Copper-exporting ATPase | copB;CO98_2152 | 8,813E+10 | 2,854E+09 |
| **Q2FKJ8** | Carbamate kinase 3 | arcC3 | 2,487E+08 | 6,588E+07 |
| **Q2FKQ5** | Chromosomal replication initiator protein DnaA | dnaA | 6,981E+09 | 5,136E+09 |
| **Q2FUQ4** | Ribosomal RNA small subunit methyltransferase G | rsmG | 5,940E+08 | 5,871E+08 |
| **Q2FUW3** | Accessory Sec system protein Asp1;Uncharacterized protein | asp1;SAV2652 | 1,729E+08 | 8,079E+07 |
| **Q2FUW4** | Accessory Sec system protein Asp2;Accessory secretory protein Asp2;Uncharacterized protein | asp2;NWMN_2550 | 1,487E+08 | 1,107E+08 |
| **Q2FVA2** | Uncharacterized protein;HAD family hydrolase;Cof-like hydrolase | SAOUHSC_02831;supH;HMPREF0776_0583 | 6,813E+07 | 4,622E+07 |
| **Q2FVE6** | Uncharacterized protein | SAOUHSC_02768;SAUSA300_2412;N | 1,287E+09 | 3,825E+08 |
| **Q2FVE7** | Peptide ABC transporter, peptide-binding protein, putative;Peptide ABC transporter, peptide-binding proteiin | SAOUHSC_02767;SACOL2476 | 5,812E+09 | 8,699E+07 |
| **Q2FVI3** | Uncharacterized protein;Putative lipoprotein | SAOUHSC_02727;AFO87_00200;ST398NM01_2488 | 8,229E+08 | 2,424E+08 |
| **Q2FVR9** | Isopentenyl-diphosphate delta-isomerase | fni | 1,306E+09 | 4,204E+08 |
| **Q2FVT7** | Uncharacterized protein;N-acetyl-L%2CL-diaminopimelate deacetylase;Amino acid amidohydrolase | SAOUHSC_02605;yxeP_3 | 1,258E+08 | 9,938E+07 |
| **Q2FW81** | Probable uridylyltransferase SAOUHSC_02423;Probable uridylyltransferase SA1974;Uridylyltransferase | SAOUHSC_02423;SA1974 | 1,125E+09 | 9,567E+08 |
| **Q2FWH3** | D-alanine--D-alanine ligase | ddl | 7,767E+09 | 6,630E+09 |
| **Q2FWK9** | Uncharacterized protein | SAOUHSC_02280;MW1976;SA1857 | 4,673E+08 | 2,447E+08 |
| **Q2FWL5** | ABC transporter, ATP-binding protein, putative;ABC transporter ATP-binding protein;Putative ABC transporter ATP-binding protein | SAOUHSC_02274;yheS_1 | 1,947E+09 | 5,187E+08 |
| **Q2FWL7** | Uncharacterized protein;Putative transport system permease protein;Putative membrane spanning protein | SAOUHSC_02272;yeeE | 3,837E+08 | 1,045E+08 |
| **Q2FWY9** | Glutamyl-tRNA(Gln) amidotransferase subunit A;Glutamyl-tRNA(Gln) amidotransferase subunit A | gatA | 6,003E+09 | 3,032E+09 |
| **Q2FWZ0** | Aspartyl/glutamyl-tRNA(Asn/Gln) amidotransferase subunit B | gatB | 6,714E+09 | 3,819E+09 |
| **Q2FX99** | UPF0754 membrane protein SAOUHSC_01978;Uncharacterized protein;UPF0754 membrane protein MW1787 | SAOUHSC_01978;RU53_1946 | 4,408E+09 | 6,024E+08 |
| **Q2FXJ8** | Uncharacterized protein;PTS glucose transporter subunit IIBC | SAOUHSC_01836;ptaA | 3,649E+09 | 6,536E+07 |
| **Q2FXP7** | Threonine--tRNA ligase | thrS | 1,013E+10 | 4,864E+09 |
| **Q2FXT8** | Protein-export membrane protein SecDF;Bifunctional preprotein translocase subunit SecD/SecF | SAOUHSC_01746;secD | 3,416E+10 | 9,749E+09 |
| **Q2FXU8** | Uncharacterized protein;Putative ATPase (AAA family);DNA-dependent ATPase;Uncharacterized protein O | SAOUHSC_01734;rarA | 2,515E+09 | 6,845E+08 |
| **Q2FXZ8** | Uncharacterized protein;Serine protease;Membrane-bound serine protease | SAOUHSC_01677;QU38_09060;BN1321_260212 | 6,306E+09 | 1,278E+09 |
| **Q2FY32** | Shikimate kinase | aroK | 2,090E+08 | 7,950E+07 |
| **Q2FY82** | Conserved hypothetical phage protein;Lipoprotein;Uncharacterized protein | SAOUHSC_01583;AFO87_01650;SAZ172_1502 | 7,650E+08 | 1,309E+08 |
| **Q2FYG6** | Heptaprenyl diphosphate syntase component II, putative;Farnesyl pyrophosphate synthetase | SAOUHSC_01486;hepT | 7,554E+08 | 1,526E+08 |
| **Q2FYQ7** | Putative oligopeptide transport ATP-binding protein oppD2;Putative oligopeptid | oppD2 | 1,611E+08 | 2,958E+07 |
| **Q2FYZ5** | Glycerol kinase | glpK | 2,616E+09 | 9,596E+08 |
| **Q2FYZ9** | DNA mismatch repair protein MutS | mutS | 2,701E+09 | 1,362E+09 |
| **Q2FZ09** | Protein RecA | recA | 1,223E+10 | 6,276E+09 |
| **Q2FZ18** | Uncharacterized protein;DNA translocase FtsK | SAOUHSC_01253;ftsK | 2,761E+09 | 2,390E+09 |
| **Q2FZ28** | ATP-dependent protease ATPase subunit HslU;ATP-dependent protease ATPase subunit | hslU | 2,206E+09 | 1,307E+09 |
| **Q2FZ63** | Uncharacterized protein;Ribosome small subunit-dependent GTPase A | SAOUHSC_01188;rsgA | 3,650E+08 | 1,360E+08 |
| **Q2FZ95** | Cell division protein FtsL | ftsL | 3,907E+08 | 1,058E+08 |
| **Q2FZJ0** | Phosphoribosylformylglycinamidine synthase subunit PurL | purL | 2,140E+09 | 1,169E+09 |
| **Q2FZT4** | Uncharacterized protein SAOUHSC_00906;Fumarylacetoacetate hydrolase family protein | SAOUHSC_00906;AL077_06395 | 1,406E+10 | 1,097E+10 |
| **Q2FZX4** | Lipoyl synthase | lipA | 8,932E+08 | 4,960E+08 |
| **Q2G042** | Uncharacterized protein;TPR repeat family protein;TPR domain protein | SAOUHSC_00784;BN1321_180006;NWMN_0731 | 5,658E+08 | 1,616E+08 |
| **Q2G044** | Prolipoprotein diacylglyceryl transferase | lgt;CH51_03970 | 4,261E+09 | 3,409E+08 |
| **Q2G093** | Lipoteichoic acid synthase;Glycerol phosphate lipoteichoic acid synthase | ltaS;CH51_03760 | 6,125E+08 | 3,096E+08 |
| **Q2G0A6** | Deoxyribodipyrimidine photolyase, putative;Deoxyribodipyrimidine photolyase;MW0653 protein | SAOUHSC_00699;phrB;MW0653 | 1,064E+08 | 7,613E+07 |
| **Q2G0F6** | Iron compound ABC transporter, substrate-binding protein, putative;Periplasmic binding protein | SAOUHSC_00613;HMPREF0776_1614 | 1,959E+10 | 6,297E+08 |
| **Q2G0I1** | Uncharacterized protein;Hypothetical fig\|282458.1.peg.573 homolog | SAOUHSC_00584;AFO87_11280;MW05 | 9,353E+08 | 4,522E+08 |
| **Q2G0P6** | Protein-arginine kinase | mcsB | 1,453E+09 | 6,310E+08 |
| **Q2G0P8** | Transcriptional regulator CtsR;CtsR | ctsR | 1,461E+09 | 2,824E+08 |
| **Q2G0V2** | Methionine import ATP-binding protein MetN 1;Methionine import ATP-binding protein MetN | metN1;metN | 6,897E+09 | 4,005E+09 |
| **Q2G179** | Uncharacterized protein;Putative cytosolic protein;ADP-ribosylating toxin | SAOUHSC_00268;ERS365775_01057;CH51_01365 | 5,595E+08 | 4,538E+08 |
| **Q2G1G5** | PTS system EIIBC component SAOUHSC_00158;PTS system%2C IIB component / PTS system%2C IIC component | SAOUHSC_00158;ERS179246_01482 | 9,292E+10 | 8,505E+09 |
| **Q2G1P4** | Uncharacterized protein;Putative Na+/Pi-cotransporter protein;Na/Pi cotransporter | SAOUHSC_00060;BN1321_40030;BN1326_150313 | 3,064E+09 | 3,388E+08 |
| **Q2G1X6** | 7-cyano-7-deazaguanine synthase | queC | 2,067E+08 | 1,478E+08 |
| **Q2G1Z4** | Proline--tRNA ligase | proS | 7,170E+09 | 2,633E+09 |
| **Q2G218** | L-lactate dehydrogenase 1;L-lactate dehydrogenase | ldh1;ldhA;ldh | 4,097E+08 | 4,091E+08 |
| **Q2G252** | Ribosomal RNA large subunit methyltransferase H | rlmH | 9,375E+08 | 4,821E+08 |
| **Q2G275** | DNA replication and repair protein RecF | recF | 3,389E+08 | 1,175E+08 |
| **Q2G2E0** | Uncharacterized protein;Na+/H+ antiporter;Sodium/hydrogen exchanger family protein;Na+/H | SAOUHSC_00633;nhaK_1;BN1321_150070 | 7,604E+09 | 3,167E+08 |
| **Q2G2N1** | Uncharacterized protein;Putative membrane protein;Membrane protein | SAOUHSC_02001;BN1326_100006;ACR58_12900 | 3,526E+09 | 5,626E+08 |
| **Q2G2P5** | Uncharacterized protein;ABC superfamily ATP binding cassette transporter%2C binding protein | SAOUHSC_00201;gsiB | 3,806E+09 | 1,185E+09 |
| **Q2G2S0** | Adenylosuccinate lyase | purB | 1,306E+09 | 1,273E+09 |
| **Q2G2U3** | Uncharacterized protein;Lipoprotein | SAOUHSC_00022;yycH;ACH32_07145 | 6,754E+09 | 6,447E+07 |
| **Q2G2W2** | Uncharacterized protein;Membrane protein | SAOUHSC_02628;AFO87_00680;ERS179246_01335 | 1,184E+10 | 4,604E+08 |
| **Q33C61** | AraC family regulatory protein;MW1460 protein;AraC family transcriptional regulator | graE;MW1460;ACR58_00715 | 2,619E+08 | 1,230E+08 |
| **Q53635** | o-succinylbenzoate synthase | menc;menC | 2,873E+08 | 1,615E+08 |
| **Q53700** | NAD-dependent protein deacetylase | cobB | 2,907E+08 | 1,148E+08 |
| **Q53727** | ATP-dependent DNA helicase PcrA;DNA helicase | pcrA; | 3,496E+09 | 1,016E+09 |
| **Q5FBD2** | LukF-PV | lukF-PV | 2,437E+09 | 5,204E+08 |
| **Q5HCI3** | tRNA modification GTPase MnmE | mnmE | 1,414E+09 | 5,518E+08 |
| **Q5HCU5** | Probable malate:quinone oxidoreductase 2;Probable malate:quinone oxidoreductase | mqo2;mqo | 4,411E+10 | 2,471E+10 |
| **Q5HDB2** | Protein flp;Beta-lactamase family protein;Beta-lactamase | flp;fmtA_2 | 1,637E+09 | 3,959E+08 |
| **Q5HDJ0** | Probable malate:quinone oxidoreductase 1;Probable malate:quinone oxidoreductase | mqo1;mqo | 6,614E+09 | 2,307E+09 |
| **Q5HDJ9** | Membrane-associated protein TcaA;Membrane-associated protein | tcaA | 4,246E+09 | 9,756E+07 |
| **Q5HDT3** | Cyclic pyranopterin monophosphate synthase accessory protein | moaC | 1,148E+08 | 1,032E+08 |
| **Q5HDW4** | 30S ribosomal protein S3 | rpsC | 5,169E+10 | 4,101E+10 |
| **Q5HE76** | UDP-N-acetylglucosamine 1-carboxyvinyltransferase 2;UDP-N-acetylglucosamine 1-carboxyvinyltransferase | murA2;murA | 4,682E+08 | 2,626E+08 |
| **Q5HE81** | Thymidine kinase | tdk | 3,811E+08 | 1,843E+08 |
| **Q5HE91** | ATP synthase subunit a | atpB;atpA_1 | 2,514E+09 | 1,580E+09 |
| **Q5HEA0** | UDP-N-acetylglucosamine 1-carboxyvinyltransferase 1;UDP-N-acetylglucosamine 1-carboxyvinyltransferase | murA1;murA | 4,214E+09 | 1,683E+09 |
| **Q5HEA9** | Membrane protein insertase YidC | yidC | 1,272E+10 | 4,576E+08 |
| **Q5HEB9** | DEAD-box ATP-dependent RNA helicase CshA | cshA | 6,525E+09 | 5,347E+09 |
| **Q5HEE5** | Ketol-acid reductoisomerase | ilvC | 2,573E+09 | 1,005E+09 |
| **Q5HEK1** | Probable manganese-dependent inorganic pyrophosphatase | ppaC | 1,300E+10 | 1,119E+10 |
| **Q5HEL3** | Staphopain A | sspP;s | 9,411E+08 | 4,777E+08 |
| **Q5HEQ0** | Monofunctional glycosyltransferase | mgt;sgtB | 1,156E+09 | 1,044E+09 |
| **Q5HEY9** | S-adenosylmethionine synthase | metK | 1,004E+10 | 6,153E+09 |
| **Q5HF24** | D-alanine aminotransferase;D-alanine | dat | 1,729E+09 | 1,656E+09 |
| **Q5HF39** | Acetoin utilization protein AcuC;Acetoin utilization acuC protein | acuC | 5,528E+08 | 2,847E+08 |
| **Q5HF56** | Septation ring formation regulator EzrA | ezrA;ezrA_1 | 1,535E+11 | 1,749E+10 |
| **Q5HF59** | Probable tRNA sulfurtransferase | thiI | 5,925E+08 | 3,429E+08 |
| **Q5HF76** | Pyruvate kinase | pyk;pykA;QU38_08455 | 6,307E+10 | 4,724E+10 |
| **Q5HF86** | Glyceraldehyde-3-phosphate dehydrogenase 2;Glyceraldehyde-3-phosphate dehydrogenase | gapA2;gap2 | 2,859E+09 | 1,532E+09 |
| **Q5HFB9** | GTPase Obg | obg | 6,435E+09 | 3,790E+09 |
| **Q5HFC3** | S-adenosylmethionine:tRNA ribosyltransferase-isomerase | queA | 7,579E+08 | 4,569E+08 |
| **Q5HFD1** | Probable cell wall amidase LytH;Probable cell wall amidase lytH | lytH | 4,386E+09 | 2,010E+09 |
| **Q5HFH6** | Elongation factor 4 | lepA | 5,485E+09 | 2,817E+09 |
| **Q5HFJ5** | Glycine--tRNA ligase | glyQS | 1,029E+10 | 9,638E+09 |
| **Q5HFJ8** | DNA primase | dnaG | 8,488E+08 | 2,800E+08 |
| **Q5HFP8** | Exodeoxyribonuclease 7 large subunit | xseA | 5,439E+08 | 3,937E+08 |
| **Q5HFR8** | Ribonuclease Z | rnz;rn | 7,842E+07 | 7,330E+07 |
| **Q5HFZ6** | Thymidylate synthase | thyA | 2,330E+08 | 1,951E+08 |
| **Q5HG01** | Probable CtpA-like serine protease;Carboxy-terminal processing proteinase ctpA;Serine protease | SACOL1455;ctpA;CH51_07455 | 1,237E+10 | 9,157E+09 |
| **Q5HG45** | Aminoacyltransferase FemA;Methicillin resistance protein FemA;FemAB family protein | femA;HM | 2,509E+09 | 2,194E+09 |
| **Q5HG59** | Phosphatidylglycerol lysyltransferase;FmtC (MprF) protein involved in methicillin resistance / L-lysine modification of phosphatidylgl | mprF | 4,369E+09 | 1,019E+09 |
| **Q5HG65** | DNA topoisomerase 4 subunit B | parE;gyrB | 2,557E+09 | 2,148E+09 |
| **Q5HGA3** | Cardiolipin synthase 1;Cardiolipin synthase | cls1;NWMN_1230 | 7,529E+09 | 2,689E+09 |
| **Q5HGG7** | DNA polymerase III PolC-type | polC | 3,299E+09 | 1,270E+09 |
| **Q5HGG9** | Putative zinc metalloprotease SACOL1281;Zinc metalloprotease;Putative zinc metalloprotease MW1145 | SACOL1281;ERS179246_01117;MW | 1,305E+10 | 4,427E+08 |
| **Q5HGM7** | Orotate phosphoribosyltransferase | pyrE | 6,174E+08 | 5,284E+08 |
| **Q5HGN0** | Carbamoyl-phosphate synthase small chain | carA | 3,978E+09 | 2,293E+09 |
| **Q5HGV3** | Iron-regulated surface determinant protein C | isdC | 6,772E+08 | 6,322E+07 |
| **Q5HH13** | Phosphoribosylformylglycinamidine cyclo-ligase;Phosphoribosylformylglycinamidine c | purM | 1,675E+09 | 3,543E+08 |
| **Q5HH38** | 1,4-dihydroxy-2-naphthoyl-CoA synthase | menB | 1,456E+10 | 8,007E+09 |

| | | | | |
|---|---|---|---|---|
| **Q5HHB0** | Chaperone protein ClpB | clpB | 8,516E+09 | 7,195E+09 |
| **Q5HHB9** | Signal peptidase IB;Signal peptidase I | spsB;SAOUHSC_00903 | 2,993E+10 | 8,997E+08 |
| **Q5HHG8** | UPF0051 protein SACOL0918;Uncharacterized protein;FeS cluster formation protein | SACOL0918;ST398NM01_0901;sufB | 1,635E+09 | 1,491E+09 |
| **Q5HHH0** | Probable cysteine desulfurase;Cysteine desulfurase;Selenocysteine lyase | csd | 7,456E+08 | 5,256E+08 |
| **Q5HHR7** | Protein translocase subunit SecA 1;Protein translocase subunit SecA | secA1;secA | 2,357E+10 | 2,206E+10 |
| **Q5HHT2** | UDP-N-acetylenolpyruvoylglucosamine reductase | murB | 3,078E+08 | 2,185E+08 |
| **Q5HHY5** | Undecaprenyl-diphosphatase;U | uppP | 5,898E+09 | 1,677E+08 |
| **Q5HI09** | Response regulator protein GraR | graR | 4,293E+08 | 3,216E+08 |
| **Q5HI96** | Putative pyridoxine kinase;Phosphomethylpyrimidine kinase | pdxK;thiD | 4,279E+09 | 2,231E+09 |
| **Q5HIG5** | Hypoxanthine-guanine phosphoribosyltransferase;Hypoxanthine phosphoribosyltransferase | hpt | 9,188E+08 | 2,717E+08 |
| **Q5HIH5** | Ribose-phosphate pyrophosphokinase | prs | 1,763E+10 | 5,733E+09 |
| **Q5HIJ5** | Thymidylate kinase | tmk | 1,558E+09 | 1,044E+09 |
| **Q5HIQ9** | Xanthine phosphoribosyltransferase | xpt;xprT | 9,816E+09 | 6,241E+09 |
| **Q5HJ87** | Protein EssB;Type VII secretion protein EssB;Putative secretion system component EssB/YukC | essB | 6,100E+09 | 9,976E+08 |
| **Q5HJR8** | Uncharacterized HTH-type transcriptional regulator SACOL0084;Uncharacterized protein;Transcriptional regulator, AraC family | SACOL0084;NWMN_0046 | 1,055E+08 | 9,548E+07 |
| **Q5HJX6** | Sensor protein kinase WalK | walK | 7,741E+09 | 3,055E+09 |
| **Q6WS49** | Domain of uncharacterised function (DUF3578);Uncharacterized protein | ORF;SACOL0026;SAKOR_0002 | 3,717E+08 | 1,935E+08 |
| **Q70MI7** | Gamma-aminobutyrate permease;Lysine-specific permease | lysP | 1,359E+09 | 1,308E+08 |
| **Q79C39** | 35-aminoglycoside phosphotransferase | aphA-3 | 2,316E+10 | 1,401E+10 |
| **Q7A0T9** | UPF0403 protein MW1467;Uncharacterized protein | MW1467;HMPREF0776_2549 | 5,136E+08 | 4,267E+08 |
| **Q7A2T0** | Protein FmtA;Methicillin resistance protein FmtA | fmtA;QU38_05530 | 5,736E+09 | 2,321E+09 |
| **Q7A2V2** | Uncharacterized membrane protein SAV0746;Uncharacterized membrane protein in llm 5region;Uncharacterized membrane protein | SAV0746;BN1321_170080 | 2,973E+09 | 1,627E+08 |
| **Q7A3E1** | Dehydrosqualene synthase;Squalene/phytoene synthase | crtM;RU53_2626 | 1,980E+08 | 9,605E+07 |
| **Q7A3H8** | Putative NAD(P)H nitroreductase SA2311;Putative NAD(P)H nitroreductase SACOL2534;Nitroreductase family protein | SA2311;SACOL2534 | 9,287E+08 | 6,537E+08 |
| **Q7A3W1** | Ferredoxin--NADP reductase | SA2162;SAV2372 | 1,180E+09 | 5,492E+08 |
| **Q7A4B3** | PTS system mannitol-specific EIICB component;PTS mannitol transferase subunit IIB | mtlA | 1,983E+09 | 1,381E+08 |
| **Q7A537** | Tyrosine--tRNA ligase | tyrS | 8,276E+08 | 4,406E+08 |
| **Q7A5N7** | Uncharacterized protein SA1241;Uncharacterized protein SAUSA300_1302;Putative nitric-oxide reductase | SA1241;SAUSA300_1302; | 4,981E+08 | 3,667E+08 |
| **Q7A5W3** | tRNA-2-methylthio-N(6)-dimethylallyladenosine synthase | miaB | 7,356E+08 | 2,542E+08 |
| **Q7A5Z4** | Uncharacterized protein SA1069;Putative dihydroxyacetone/glyceraldehyde kinase;Dihydroxyacetone kinase family protein | SA1069;yloV;ERS09300 | 6,635E+09 | 4,478E+09 |
| **Q7A6H8** | NAD-specific glutamate dehydrogenase;Glutamate dehydrogenase | gluD;gudB | 8,891E+09 | 7,366E+09 |
| **Q7A6J4** | NADH dehydrogenase-like protein SA0802;Pyridine nucleotide-disulfide oxidoreductase;NADH dehydrogenase family | SA0802;RU53_936 | 1,564E+10 | 1,517E+10 |
| **Q7A6T6** | Putative lipid kinase SA0681;Putative lipid kinase NWMN_0695;Putative lipid kinase MW0688 | SA0681;NWMN_0695;MW | 1,776E+09 | 1,443E+09 |
| **Q7A782** | FMN-dependent NADPH-azoreductase;FMN-dependent NADH-azoreductase | azo1;azo | 8,382E+09 | 6,362E+09 |
| **Q7A7B2** | Transcription-repair-coupling factor | mfd | 2,879E+09 | 1,406E+09 |
| **Q7A8E1** | Transcriptional regulatory protein WalR;Two-component response regulator WalR;Transcriptional regulatory protein YycF | walR;yycF | 6,766E+09 | 6,656E+09 |
| **Q7BGA5** | Ferric hydroxamate receptor 2;Iron (Fe+3) ABC superfamily ATP binding cassette transporter, binding protein | fhud2;fhuD | 2,797E+10 | 6,657E+08 |
| **Q7BHL7** | Regulatory protein MsrR;LytR family transcriptional regulator;Peptide methionine sulfoxide reductase regulator MsrR | msrR;ACH32_13790 | 6,138E+08 | 6,123E+08 |
| **Q7DHH4** | MecA;Penicillin-binding protein MecA;PBP2a;Beta-lactam-inducible penicillin-binding protein | mecA;SAKOR_00039 | 4,744E+10 | 4,420E+10 |
| **Q83TG5** | Lipoprotein;Electron transfer DM13 family protein;Uncharacterized protein | AFO87_02840;BN1321_170041;MW0670 | 1,866E+11 | 1,364E+10 |
| **Q840P7** | Histidine protein kinase SaeS | saeS | 1,027E+11 | 3,604E+10 |
| **Q8NUN2** | Pantothenate synthetase | panC | 1,178E+09 | 5,970E+08 |
| **Q8NUQ6** | Dehydrosqualene desaturase | crtN | 9,552E+08 | 5,559E+08 |
| **Q8NUS2** | PTS system glucoside-specific EIICBA component;PTS system glucose-specific transporter subunit IICBA | glcB;QU38_16450 | 2,235E+09 | 6,077E+08 |
| **Q8NVE6** | Glutamine--fructose-6-phosphate aminotransferase [isomerizing] | glmS | 9,012E+09 | 3,762E+09 |
| **Q8NVH5** | Thiamine-phosphate synthase | thiE | 4,099E+08 | 1,224E+08 |
| **Q8NVJ5** | tRNA N6-adenosine threonylcarbamoyltransferase;tRNA N6-adenosine threonylcarbamoyltransferase | tsaD | 8,733E+08 | 3,867E+08 |
| **Q8NVK5** | Accessory gene regulator protein B | agrB | 3,893E+09 | 1,099E+09 |
| **Q8NVS5** | Sodium-dependent dicarboxylate transporter SdcS;Sodium-dependent dicarboxylate transporter SdcS | sdcS | 8,185E+08 | 1,286E+08 |
| **Q8NVU6** | Glutamate-1-semialdehyde 2,1-aminomutase 2;Glutamate-1-semialdehyde 2,1-aminomutase | hemL2 | 1,583E+09 | 8,674E+08 |
| **Q8NW59** | Acetyl-coenzyme A carboxylase carboxyl transferase subunit beta | accD | 2,271E+09 | 1,265E+09 |
| **Q8NW61** | Isocitrate dehydrogenase [NADP] | icd | 3,473E+10 | 2,038E+10 |
| **Q8NW74** | Porphobilinogen deaminase | hemC | 9,448E+08 | 8,801E+08 |
| **Q8NW95** | UPF0271 protein MW1555;UPF0271 protein SACOL1660;UPF0271 protein SA1433;UPF027 | MW1555;SACOL1660;SA1433 | 4,020E+08 | 1,636E+08 |
| **Q8NWQ4** | L-threonine dehydratase catabolic TdcB;Threonine dehydratase | tdcB;ilvA;S | 6,027E+08 | 2,830E+08 |
| **Q8NWR0** | DegV domain-containing protein MW1315;Uncharacterized protein;DegV domain-containing protein SA1258 | MW1315;SAZ172_1438 | 5,414E+09 | 3,939E+09 |
| **Q8NWU0** | Aminoacyltransferase FemB;FemB%2C factor involved in methicillin resistance / Glycine interpeptide bridge formation OS | femB | 4,566E+09 | 2,835E+09 |
| **Q8NWZ5** | Isoprenyl transferase | uppS | 4,485E+08 | 1,951E+08 |
| **Q8NX11** | ATP-dependent DNA helicase RecG | recG | 5,912E+08 | 4,655E+08 |
| **Q8NX16** | Probable dual-specificity RNA methyltransferase RlmN | rlmN | 7,850E+08 | 4,626E+08 |
| **Q8NX38** | Putative cysteine ligase BshC | bshC | 7,490E+07 | 4,096E+07 |
| **Q8NX56** | Endonuclease MutS2 | mutS2;mutSB | 1,066E+09 | 3,399E+08 |
| **Q8NXF2** | Argininosuccinate synthase | argG | 2,168E+08 | 4,754E+07 |
| **Q8NXM4** | DegV domain-containing protein MW0711;EDD domain protein, DegV family | MW0711;HMPREF0776_1759 | 1,669E+09 | 1,407E+09 |
| **Q8NXR5** | Sensor histidine kinase GraS;Histidine kinase | graS;QU38_10325 | 1,332E+09 | 2,547E+08 |
| **Q8NXS6** | Teichoic acids export ATP-binding protein TagH | tagH | 3,686E+09 | 2,300E+09 |
| **Q8NXZ0** | Lysine--tRNA ligase | lysS | 1,730E+10 | 9,843E+09 |
| **Q8NXZ2** | Dihydropteroate synthase;Dihydr | folP | 1,256E+09 | 8,211E+08 |
| **Q8NY69** | GMP synthase [glutamine-hydrolyzing] | guaA | 1,789E+10 | 1,236E+10 |
| **Q8NYC2** | Lipase 2;Glycerol ester hydrolase%3B Lipase;Truncated triacylglycerol lipase | lip2;geh | 1,550E+10 | 1,126E+10 |
| **Q8VUV7** | ACP synthase;Uncharacterized protein | AL493_01530;SAUSA300_0041 | 3,154E+08 | 1,104E+08 |
| **Q8VUV8** | AAA family ATPase;Putative transcriptional regulator | AL493_01535;ERS411017_01126 | 2,226E+09 | 8,581E+08 |
| **Q931H9** | Accessory gene regulator A;Accessory protein regulator protein A | agrA | 1,008E+10 | 7,393E+09 |
| **Q99R74** | 4,4-diaponeurosporenoate glycosyltransferase | crtQ | 1,368E+08 | 5,517E+07 |
| **Q99RD4** | UTP--glucose-1-phosphate uridylyltransferase | gtaB | 3,280E+08 | 1,419E+08 |
| **Q99RL2** | Immunoglobulin-binding protein sbi | sbi | 3,445E+09 | 3,140E+08 |
| **Q99S33** | 50S ribosomal protein L5 | rplE | 1,557E+10 | 1,326E+10 |
| **Q99SC3** | Pyrimidine-nucleoside phosphorylase | pdp | 1,152E+10 | 9,994E+09 |
| **Q99SN8** | Uncharacterized leukocidin-like protein 1;Leukocidin F subunit LukF | SAV2004;SACOL2004 | 2,756E+09 | 4,787E+08 |
| **Q99UZ7** | Divalent metal cation transporter MntH | mntH | 4,134E+09 | 1,051E+08 |
| **Q99V32** | N5-carboxyaminoimidazole ribonucleotide synthase | purK | 3,453E+08 | 1,219E+08 |
| **Q99V37** | Probable quinol oxidase subunit 1;Cytochrome aa3-600 quinol oxidase (Subunit I) | qoxB | 2,292E+10 | 3,295E+09 |
| **Q99V72** | Peptide chain release factor 3 | prfC | 3,300E+09 | 1,096E+09 |
| **Q99V86** | UPF0738 protein SAV1005;UPF0738 protein SACOL1009;UPF0738 protein SAKOR_00921 | SAV1005;SACOL1009 | 1,648E+08 | 9,944E+07 |
| **Q99VJ2** | Extracellular matrix protein-binding protein emp | emp | 7,324E+07 | 3,827E+07 |
| **Q99W36** | Putative proline/betaine transporter | proP | 6,167E+08 | 4,849E+07 |
| **Q99W73** | Cysteine--tRNA ligase | cysS | 1,929E+09 | 1,812E+09 |
| **Q99X00** | Uncharacterized response regulatory protein SAV0223;Uncharacterized response regulatory protein SAUSA300_0217 | SAV0223;SAUSA300_0217 | 5,475E+08 | 1,605E+08 |
| **Q9EYL5** | Disulfide bond protein A;Thiol:disulfide interchange protein dsbA;Lipoprotein | dsbA;SAKOR_02387;BN1321_400055 | 7,233E+09 | 1,968E+08 |
| **Q9F1K0** | DNA polymerase III subunit alpha | dnaE | 5,124E+08 | 3,128E+08 |
| **Q9KWK9** | Putative uncharacterized protein yvqF;Transporter;Putative membrane protein | yvqF;QU38_07135;BN1326_100027 | 2,387E+09 | 1,881E+08 |
| **Q9RDT1** | Uncharacterized protein;YycH family protein | yycI | 2,571E+09 | 5,026E+07 |
| **Q9REV4** | UDP-GlcNAc 2-epimerase;UDP-N-acetylglucosamine 2-epimerase | mnaA | 1,160E+09 | 4,319E+08 |
| **Q9RL81** | Phosphorylated protein phosphatase;Protein phosphatase;Protein phosphatase 2C | prpC;QU38_12430;SAKOR_01146 | 5,741E+08 | 2,309E+08 |
| **Q9RQG6** | Multidrug transporter;SA2056 protein;RND transporter, HAE1/HME family, permease protein | swrC;SA2056;HMPREF0776 | 2,804E+10 | 6,872E+09 |
| **Q9RQQ0** | Biofilm operon icaADBC HTH-type negative transcriptional regulator IcaR;Transcriptional regulator | icaR;QU38_15745 | 6,544E+08 | 3,076E+08 |
| **Q9S446** | LPXTG specific sortase A;Sortase | srtA;ST398NM01_2578 | 1,224E+10 | 5,765E+08 |
| **Q9ZAH8** | Membrane protein;Membrane-flanked domain-containing protein;Uncharacterized protein | ORF3;RU53_2135;NWMN_1978 | 3,441E+09 | 5,734E+08 |
| **Q9ZAH9** | Bacterial membrane flanked domain protein;Uncharacterized protein | ORF2;RU53_2136 | 1,229E+09 | 1,853E+08 |
| **T1Y6D9** | Ribosomal-protein-alanine acetyltransferase;Acetyltransferase, GNAT family | SAKOR_00676;BN1321_170010 | 1,025E+08 | 3,989E+07 |
| **T1Y6E4** | Transport ATP-binding protein cydD;ABC superfamily ATP binding cassette transporter, ABC protein | SAKOR_00681;RU53_747 | 2,427E+09 | 3,002E+08 |
| **T1Y6N4** | Two-component sensor kinase yesM;Uncharacterized sensor-like histidine kinase MW0199;Uncharacterized sensor-like histidine kin | SAKOR_00197;MW0199 | 5,926E+08 | 5,581E+08 |
| **T1Y720** | Histidinol-phosphate aminotransferase | hisC | 1,569E+08 | 1,019E+08 |
| **T1Y7D4** | N-acetylglucosaminyldiphosphoundecaprenol N-acetyl-beta-D-mannosaminyltransferase;Putative N-acetylmannosaminyltransferase | SAKOR_00630;tagA | 1,043E+09 | 5,399E+08 |
| **T1Y893** | Serine/threonine protein kinase;Serine/threonine-protein kinase PrkC;Non-specific serine/threonine protein kinase | SAKOR_01147;prkC | 5,615E+09 | 2,064E+09 |
| **T1Y8P5** | Kinase autophosphorylation inhibitor kipI;Allophanate hydrolase | SAKOR_00714;kipI_1;QU38_10625 | 1,181E+09 | 8,785E+08 |
| **T1Y9P2** | Integral membrane protein;UPF0421 protein MW1829 | SAKOR_01846;MW1829;ERS093009_02228 | 1,118E+09 | 4,461E+08 |
| **T1Y9Y4** | Carbamoyl-phosphate synthase (glutamine-hydrolyzing);Carbamoyl-phosphate synthase large chain | SAKOR_01129;carB | 1,277E+10 | 5,230E+09 |
| **T1YAA1** | Diacylglycerol kinase family protein;Diacylglycerol kinase | SAKOR_01857;dagK | 2,195E+09 | 9,158E+08 |
| **T1YAM7** | Leukocidin S subunit LukS;Leukocidin/lemolysin toxin family protein;Uncharacterized leukocidin-like protein 2 | SAKOR_01987;lukS | 8,610E+09 | 1,849E+09 |
| **T1YAQ5** | DNA mismatch repair protein mutS;DNA mismatch repair ATPase;MW1972 protein | SAKOR_02012;RU53_2105 | 1,149E+10 | 1,893E+08 |
| **T1YAY1** | Penicillin binding protein transpeptidase | SAKOR_01499 | 1,946E+10 | 1,355E+09 |
| **T1YAZ7** | GTPase Era | era | 6,324E+09 | 3,326E+09 |
| **T1YBL4** | Putative membrane protein insertion efficiency factor | SAKOR_01739;SACOL1842 | 2,415E+08 | 2,320E+08 |
| **T1YBN3** | Staphyloxanthin biosynthesis protein CrtP;Diapolycopene oxygenase | SAKOR_02556;crtP | 5,165E+08 | 1,054E+08 |
| **T1YBW8** | Uncharacterized protein | SAKOR_02417;ST398NM01_2485;MW | 1,154E+10 | 1,072E+09 |
| **T1YC07** | Uncharacterized protein;Lysostaphin resistance protein A | SAKOR_02305;lyrA | 1,157E+10 | 1,070E+09 |
| **T1YCI5** | Rod shape-determining protein rodA;Cell shape determining protein RodA;Rod shape-determining protein RodA | SAKOR_02049;rodA | 3,063E+09 | 1,923E+08 |

| | | | | |
|---|---|---|---|---|
| **T1YCQ6** | Acyltransferase family protein;O-acetyltransferase OatA;O-acetyltransferase | SAKOR_02560;oatA | 3,351E+09 | 1,481E+09 |
| **T1YCS8** | Putative membrane associated protein;Putative uncharacterized protein orf2;Uncharacterized protein | SAKOR_02129;orf2;QU38 | 2,570E+10 | 9,442E+08 |
| **T1YCU2** | Siderophore synthase;Sialic acid synthase;Uncharacterized protein | SAKOR_02149;iucC_2;SAOUHSC_02436 | 1,615E+09 | 1,030E+09 |
| **T1YCZ2** | ABC transporter permease protein;ABC superfamily ATP binding cassette transporter, membrane protein | SAKOR_02620;RU53_2684 | 1,894E+08 | 9,036E+07 |
| **T1YD14** | N-acetylmuramidase;N-acetylmuramoyl-L-alanine amidase domain-containing protein SAOUHSC_02979 | SAKOR_02645;SAOUHSC_02979 | 1,314E+09 | 3,686E+08 |
| **T1YD26** | Nickel transport ATP-binding protein nikD;Nickel import ATP-binding protein NikD | SAKOR_02448;opp-1D | 4,518E+07 | 4,086E+07 |
| **T1YDP5** | Glycosyltransferase stabilizing protein Gtf2 | gtf2 | 6,172E+07 | 4,007E+07 |
| **U5NVV0** | Diamine N-acetyltransferase | speG | 2,438E+08 | 2,251E+08 |
| **U5NZ28** | Universal stress protein | AL493_01650 | 7,673E+09 | 3,832E+09 |
| **U5NZ39** | Lipoprotein;Uncharacterized protein;PF07563 family protein | ydhK;CO98_2151 | 3,087E+10 | 1,048E+09 |
| **W8TQF1** | Allergen V5/Tpx-1 like protein;SCP-like protein | AFO87_13590;HMPREF0776_2111;ERS179246_00546 | 9,228E+09 | 4,205E+08 |
| **W8TR17** | AraC family regulatory protein;Transcriptional regulator, AraC family | melR_2;HMPREF077 | 1,758E+09 | 1,204E+09 |
| **W8TRE7** | Glycine/betaine ABC transporter periplasmic protein;ABC transporter, quaternary amine uptake transporter family, substrate-bindin | opuCC_1;BN1321_170056 | 1,665E+10 | 2,818E+09 |
| **W8TRJ9** | Iron-sulfur cluster carrier protein | apbC;SAZ172_2267 | 9,300E+09 | 3,812E+09 |
| **W8TRM3** | Putative thiol-disulfide oxidoreductase%2C DCC family;Uncharacterized protein | AFO87_07670;SAKOR_02108 | 1,054E+09 | 3,091E+08 |
| **W8TRQ9** | Amino acid permease;Putative amino acid permease;MW0539 protein | lysP_1;BN1326_50067;MW0539 | 1,754E+09 | 5,256E+07 |
| **W8TSS5** | Cytosolic protein containing multiple CBS domains;CBS domain containing protein;Uncharacterized protein | ytoI;ST398NM01_1758 | 2,298E+08 | 1,650E+08 |
| **W8TSU2** | DnaI;Primosomal protein;Primosomal protein DnaI | dnaI;HMPREF0776_2717 | 2,131E+09 | 2,028E+09 |
| **W8TSW2** | Bifunctional folylpolyglutamate synthase/dihydrofolate synthase;Folylpolyglutamate synthase;Bifunctional protein FolC | folC;QU38_08645 | 6,289E+08 | 2,618E+08 |
| **W8TTH4** | TetR family regulatory protein;Transcriptional regulator, TetR family;Uncharacterized protein | AFO87_06025;BN1321_420056;NWMN_2452 | 2,989E+08 | 2,646E+08 |
| **W8TTZ1** | Glycerol uptake operon antiterminator regulatory protein;Glycerol uptake operon antiterminator | glpP | 2,885E+08 | 8,721E+07 |
| **W8TUK8** | DNA starvation/stationary phase protection protein;General stress protein | dps;ACH32_04035 | 7,223E+09 | 1,689E+09 |
| **W8TV14** | ABC-2 transporter family protein;Uncharacterized protein | AFO87_13195;ST398NM01_2051;ERS179246_01903 | 8,457E+09 | 3,526E+08 |
| **W8TVJ6** | Multimodular transpeptidase-transglycosylase;Membrane carboxypeptidase;Transglycosylase domain protein | sgtA;mrcB | 1,172E+09 | 1,603E+08 |
| **W8TVM3** | Alkaline phosphatase synthesis transcriptional regulatory protein;PhoP;PhoP family transcriptional regulator | phoP;ST398NM01_1747 | 2,726E+09 | 2,720E+09 |
| **W8TW18** | Acetyl-CoA carboxylase biotin carboxylase subunit;Biotin carboxylase of acetyl-CoA carboxylase | cfiB_2;accC | 2,023E+09 | 1,015E+09 |
| **W8TWI0** | Amino acid carrier protein;Sodium:alanine symporter | alsT;QU38_13155 | 1,537E+10 | 3,121E+08 |
| **W8TWY8** | Bifunctional phosphopantothenoylcysteine decarboxylase/phosphopantothenate--cysteine ligase | coaBC;SAKOR_01138 | 1,762E+09 | 9,194E+08 |
| **W8TZX4** | GTP-binding protein TypA;GTP-binding protein TypA/BipA | typA;QU38_05800;ST398NM01_1106 | 7,794E+09 | 6,052E+09 |
| **W8U0W4** | Lipoprotein;Uncharacterized protein | AFO87_04860;HMPREF0776_1750;QU38_10750 | 2,142E+09 | 3,755E+08 |
| **W8U1K0** | Membrane spanning protein;Uncharacterized protein;YibE/F-like protein | AL077_09845;BN1321_100034;AFO87_01830 | 3,446E+09 | 2,203E+08 |
| **W8U224** | Uncharacterised protein;Uncharacterized protein | AL493_02735;BN1326_30130;OO23_0212065 | 3,798E+08 | 2,699E+08 |
| **W8U2B2** | N-acetylmuramic acid 6-phosphate etherase | murQ | 2,947E+08 | 1,607E+08 |
| **W8U2Z7** | NAD dependent epimerase/dehydratase family protein;NmrA family protein | azoB;HMPREF0776_0637 | 6,042E+08 | 3,143E+08 |
| **W8U368** | GTP pyrophosphokinase;RelA/SpoT domain protein | ywaC;HMPREF0776_0565 | 2,602E+09 | 1,162E+09 |
| **W8U3T6** | Inosine-uridine preferring nucleoside hydrolase | rihB;BN1321_380119 | 1,938E+08 | 9,051E+07 |
| **W8U3Y4** | Energy-coupling factor transporter transmembrane protein EcfT | ecfT | 3,093E+08 | 1,017E+08 |
| **W8U414** | Cobalamin Fe3+-siderophores ABC transporter periplasmic protein;Iron(III) dicitrate-binding protein | yhfQ_1;SAKOR_02144 | 4,000E+10 | 3,922E+09 |
| **W8U458** | Transcription termination factor Rho | rho | 3,304E+09 | 1,395E+09 |
| **W8U4T8** | Membrane spanning protein;Uncharacterized protein | AFO87_08830;ERS195423_02627;RU53_2001 | 1,290E+09 | 2,298E+08 |
| **W8U570** | Bifunctional 3-deoxy-7-phosphoheptulonate synthase/chorismate mutase | aroA_2;aroX | 1,181E+10 | 4,825E+09 |
| **W8U5G4** | Acetyltransferase;Lactococcal prophage ps3 protein 05 | AFO87_10655;QU38_11085;ERS179246_00099 | 3,265E+08 | 2,319E+08 |
| **W8U5S1** | Cobalamin Fe3+-siderophores ABC transporter periplasmic protein;Periplasmic binding protein | yclQ;HMPREF0776_1746 | 7,661E+09 | 2,864E+08 |
| **W8U5T5** | ATP-dependent DNA helicase RecQ;Putative ATP-dependent DNA helicase RecQ | recQ_1;QU38_10650;recQ | 5,795E+08 | 1,544E+08 |
| **W8U6M5** | Lipoprotein;Uncharacterized protein;Putative lipoprotein | AL078_05920;HMPREF0776_2212;ERS179246_01166 | 8,713E+09 | 4,197E+08 |
| **W8U6S6** | Succinate dehydrogenase;Succinate dehydrogenase (Cytochrome b558 subunit) | sdhC | 2,545E+09 | 7,615E+08 |
| **W8U7R7** | ABC transporter ATP-binding protein;Bacteriocin ABC transporter ATP-binding protein | lolD_2;lolD_3;QU38 | 2,691E+08 | 1,884E+08 |
| **W8U818** | Gluconate permease;Transporter, gluconate:H+ symporter family | gntP;CH51_13400 | 3,967E+08 | 2,526E+08 |
| **W8U876** | Bicyclomycin resistance protein TcaB;Drug resistance transporter, Bcr/CflA subfamily | tcaB;HMPREF0769_10711 | 1,077E+09 | 2,219E+08 |
| **W8U8D2** | Exported protein;Putative exported protein;Uncharacterized protein | AFO87_00505;BN1326_140180;BN1321_400032 | 3,543E+10 | 3,611E+09 |
| **W8U8F4** | CorA-like Mg2+ transporter protein;Magnesium transporter CorA | corA_2;QU38_07630;BN1326_140175 | 2,207E+08 | 9,396E+07 |
| **W8U8Y3** | Membrane protein;Small integral membrane protein | AFO87_08140;QU38_03710;AL077_04070 | 4,996E+10 | 1,397E+09 |
| **W8U9K9** | CobB/CobQ-like glutamine amidotransferase domain-containing protein;Adenosylcobyric acid synthase;Uncharacterized protein | AFO87_14825;RU53_2023 | 4,982E+08 | 2,455E+08 |
| **W8UA21** | Serine protease;Putative protease | degP;QU38_08290;BN1326_80259 | 1,061E+11 | 2,815E+10 |
| **W8UB09** | Gamma-aminobutyrate permease;Amino acid permease | lysP_3;CH51_07000;HMPREF0776_2348 | 9,035E+09 | 4,049E+08 |
| **W8UB14** | Homoserine dehydrogenase;Homoserine | hom;dhoM;ST398NM01_1330 | 2,158E+09 | 1,324E+09 |
| **W8USJ1** | Gluconeogenesis factor | AFO87_13450;mgfK;QU38_10895 | 1,598E+09 | 5,604E+08 |
| **W8UT87** | Acetyltransferase;Acetyltransferase, GNAT family | AFO87_01785;HMPREF0769_10006;QU38_03505 | 1,064E+08 | 7,570E+07 |
| **W8UVH2** | Amino acid permease;MW2237 protein | yifK;MW2237;HMPREF0776_0367 | 3,629E+09 | 5,536E+07 |
| **W8UVI1** | Autolysin;Peptidoglycan endo-beta-N-acetylglucosaminidase | atl_1;ST398NM01_2357;QU38_05315 | 2,591E+08 | 1,809E+08 |
| **W8UVT5** | Siderophore biosynthesis protein;Uncharacterized protein;Siderophore biosynthesis protein, IucA/IucC family | iucA;MW2105;H | 9,843E+08 | 3,030E+08 |
| **W8UW64** | Abortive infection protein;CAAX amino terminal protease family;CAAX protease | AFO87_12330;ST398NM01_2068;QU38_06765 | 4,683E+09 | 4,441E+08 |
| **W8UXA3** | Acetyl-CoA carboxylase biotin carboxylase subunit;Uncharacterized protein;MW1556 protein | accC;SAKOR_01553;MW1556 | 1,936E+09 | 6,394E+08 |
| **W8UYI1** | 5-nucleotidase;Putative HAD-hydrolase YfnB;Uncharacterized protein | yfnB;QU38_12210 | 2,484E+08 | 1,027E+08 |
| **X5DRT5** | GntR family transcriptional regulator;Transcriptional regulator;UbiC transcription regulator-associated domain protein | phnF;QU38_12745 | 9,469E+08 | 4,880E+08 |
| **X5DU21** | DNA polymerase III delta prime subunit;DNA polymerase III subunit delta;Probable DNA polymerase III, delta prime subunit | holB | 3,999E+08 | 2,073E+08 |
| **X5DUZ9** | Carboxylesterase | est_1;QU38_03400;est_2 | 8,695E+08 | 3,412E+08 |
| **X5DVD1** | Iron ABC transporter substrate-binding protein;ABC transporter, solute-binding protein | AFO87_08705;HMPREF0776_0951 | 1,302E+09 | 2,410E+08 |
| **X5DW62** | Methyltransferase;Caffeoyl-CoA O-methyltransferase | AFO87_11015;QU38_08860;RU53_1711 | 1,828E+08 | 9,088E+07 |
| **X5DXD1** | HAD family hydrolase;HAD hydrolase, family IA, variant 1 | ppaX;HMPREF0769_11778;QU38_09790 | 7,822E+08 | 7,090E+08 |
| **X5DXF1** | ABC transporter ATP-binding protein;ABC transporter, ATP-binding protein | ybhF_1;HMPREF0776_1008 | 9,714E+08 | 8,294E+08 |
| **X5DXF6** | UvrABC system protein B | uvrB_2;uvrB | 5,845E+09 | 2,669E+09 |
| **X5DXL6** | Flavin reductase domain-containing protein FMN-binding;FAD-dependent oxidoreductase;Uncharacterized protein | AFO87_04130;SAKOR_02661 | 3,000E+08 | 8,821E+07 |
| **X5DYK0** | Membrane protein;Rhomboid family protein;Putative membrane peptidase%2C contains TPR repeat domain | gluP;BN1321_260187 | 3,512E+09 | 1,176E+09 |
| **X5DZQ4** | Cysteine ABC transporter ATP-binding protein;Transport ATP-binding protein cydC;SA0640 protein | cydC;SAKOR_00682;SA0640 | 1,234E+09 | 5,751E+08 |
| **X5E0C0** | Lipoprotein | metQ_2;HMPREF0776_1830;QU38_11155 | 4,966E+10 | 1,179E+09 |
| **X5E0Z1** | Lantibiotic ABC transporter ATP-binding protein;MW2433 protein;ABC transporter, ATP-binding protein | ecsA_2;MW2433 | 1,385E+10 | 1,116E+10 |
| **X5E203** | ABC transporter%2C periplasmic spermidine putrescine-binding protein potD;ABC transporter, solute-binding protein;PotD protein | potD;HMPREF0776_2095 | 3,158E+09 | 2,021E+07 |
| **X5E2G5** | Sodium:dicarboxylate symporter;Sodium:dicarboxylate symporter family protein;MW0359 protein | tcyP;MW0359 | 2,956E+10 | 1,411E+09 |
| **X5E353** | Glutaryl-CoA dehydrogenase;Acyl-CoA dehydrogenase, N-terminal domain protein;Acyl-CoA dehydrogenase FadD homolog | AFO87_07470;BN1321_60039 | 1,102E+09 | 3,882E+08 |
| **X5E3A4** | M24B family Xaa-Pro dipeptidase;Met-Xaa and Xaa-Pro aminopeptidase;Xaa-Pro dipeptidase;Xaa-Pro dipeptidase OS | AFO87_09715;yqhT;ERS093009_01324 | 1,555E+09 | 1,502E+09 |
| **X5E3G6** | Alcohol dehydrogenase;Quinone oxidoreductase, YhdH/YhfP family;Putative zinc-binding dehydrogenase | yhfP;HMPREF0776_0424 | 1,646E+09 | 5,291E+08 |
| **X5E405** | Deoxyguanosinetriphosphate triphosphohydrolase;DNTP triphosphohydrolase, putative;Putative metal-dependent phosphohydrolas | AFO87_11365;SAZ172_0617 | 2,268E+09 | 1,255E+09 |
| **X5E4F8** | Cysteine desulfurase;Cysteine desulfurase involved in tRNA thiolation;Cysteine desulfurase, NifS | iscS_1;iscSA;nifS | 2,279E+08 | 1,032E+08 |
| **X5E4M7** | Hydroxymethylpyrimidine/phosphomethylpyrimidine kinase;Hydroxymethylpyrimidine/phosphomethylpyrimidine | thiD | 3,844E+08 | 1,148E+08 |
| **X5E5Y5** | Dihydroorotate dehydrogenase (quinone) | pyrD | 2,851E+09 | 4,910E+08 |
| **X5EEX2** | Conserved protein YbcLA;Uncharacterized protein;Putative cytosolic protein | ybcI;QU38_03385 | 2,700E+08 | 2,521E+08 |
| **X5EEY9** | Integral membrane protein;Uncharacterized protein | AFO87_09795;BN1321_260151;ACH32_00410 | 3,956E+08 | 2,890E+07 |
| **X5EG65** | Protein of uncharacterised function (DUF2529);Uncharacterized protein | AFO87_13840;BN1321_340065;RU53_ | 1,183E+09 | 3,255E+08 |
| **X5EGU1** | FMN reductase;NADPH-dependent FMN reductase family protein;SA0328 protein | ssuE;BN1321_80059;SA0328 | 6,186E+07 | 2,806E+07 |
| **X5EHG2** | Cytokinin riboside 5-monophosphate phosphoribohydrolase | yvdD | 6,821E+08 | 4,815E+08 |
| **X5EHQ5** | Uncharacterised protein;Uncharacterized protein | AFO87_10500;HMPREF0776_2339;QU38_12985 | 3,912E+10 | 7,222E+09 |
| **X5EIN0** | Acetyltransferase;GNAT family acetyltransferase;Acetyltransferase (GNAT) family protein | AFO87_01190;QU38_05050;ERS179246_00699 | 2,805E+08 | 1,470E+08 |
| **X5EIP1** | HAD superfamily hydrolase;Uncharacterized protein | yqeG;SA1426;QU38_08945 | 1,242E+09 | 1,770E+08 |
| **X5EIQ4** | Dihydroxyacetone kinase;Dihydroxyacetone kinase, L subunit | dhaL;QU38_10275 | 1,442E+08 | 1,185E+08 |
| **X5EIX3** | PTS glucose transporter subunit IIB;PTS system maltose/glucose-specific IICB component;SA0233 protein | ptsG_2;RU53_218 | 1,012E+09 | 4,611E+07 |
| **X5EIY4** | Inosine-uridine preferring nucleoside hydrolase;Pyrimidine-specific ribonucleoside hydrolase RihA;Inosine/uridine-preferring nucleos | rihA | 9,746E+07 | 7,198E+07 |
| **X5EJB2** | DNA polymerase III delta subunit;DNA polymerase III, delta subunit;DNA polymerase III subunit delta | holA;BN1321_260225;CH51_08610 | 3,943E+08 | 3,316E+08 |
| **X5EJN4** | Amino acid ABC transporter substrate-binding protein;SA2202 protein | AFO87_00345;SA2202 | 1,545E+11 | 3,595E+09 |
| **X5EKK9** | Proline dehydrogenase;SA1585 protein | fadM;QU38_04695;SA1585 | 1,815E+10 | 8,774E+09 |
| **X5EKR3** | Lipoate--protein ligase;Lipoate--protein ligase 1;Lipoate--protein l | lplJ_2;SAV1028 | 3,433E+09 | 2,597E+09 |
| **X5ELX0** | 2-oxoisovalerate dehydrogenase;2-oxoisovalerate dehydrogenase beta subunit;Branched-chain alpha-keto acid dehydrogenase E1 su | bfmBAB;ST398NM01_1582 | 1,207E+09 | 5,272E+08 |
| **X5EMG1** | Ferredoxin-dependent glutamate synthase;Glutamate synthase-ferredoxin large subunit;MW2383 protein | gltB_2;gltB2; | 3,186E+09 | 1,943E+09 |
| **X5EMJ0** | Molybdenum ABC transporter ATP-binding protein;Molybdenum transport ATP-binding protein ModC | modC;ACH32_04755 | 2,545E+08 | 1,023E+08 |
| **X5EML3** | TetR family regulatory protein of MDR cluster;SA2144 protein;TetR family transcriptional regulator | AFO87_00665;SA2144;ACH32_ | 1,111E+08 | 4,557E+07 |
| **X5EN63** | Peptidase U32;Peptidase U32 family protein | yhbU_2;QU38_08865;RU53_1710 | 1,636E+08 | 8,700E+07 |

## Population IV. DRM only

| Uniprot-ID | Protein Name | Gene Name | LFQ Intensity DRM | LFQ Intensity |
|---|---|---|---|---|
| **A0A033UAK4** | Copper-translocating P-type ATPase;Copper-exporting ATPase | V070_02647;HMPREF0769_11632 | 5,619E+08 | 1,000E+00 |
| **A0A033UHR6** | DNA-directed RNA polymerase subunit beta | rpoC | 5,790E+08 | 1,000E+00 |
| **A0A033UJ34** | Copper-exporting P-type ATPase A | V070_02321 | 3,246E+08 | 1,000E+00 |
| **A0A033UK35** | Pyridoxal 5-phosphate synthase subunit PdxS | pdxS | 3,269E+08 | 1,000E+00 |

| | | | | |
|---|---|---|---|---|
| **A0A033UNE9** | Uncharacterized protein | V070_01857 | 3,654E+07 | 1,000E+00 |
| **A0A033UV54** | Uncharacterized protein | V070_01283 | 4,537E+07 | 1,000E+00 |
| **A0A033UVX6** | Tryptophan--tRNA ligase;Tryptophanyl-tRNA synthetase (Fragment) | trpS;V070_01096 | 5,102E+07 | 1,000E+00 |
| **A0A033V3I1** | GTPase Der | der | 7,054E+08 | 1,000E+00 |
| **A0A069FUU8** | Phosphoribosylformylglycinamidine synthase subunit PurL | purL | 3,318E+07 | 1,000E+00 |
| **A0A069FZR1** | Cobalt transport protein | CO98_1968 | 1,339E+08 | 1,000E+00 |
| **A0A069G4D8** | AntA/AntB antirepressor | CO98_0206 | 1,785E+07 | 1,000E+00 |
| **A0A069G4W6** | Preprotein translocase, YajC subunit | CO98_1375 | 5,367E+08 | 1,000E+00 |
| **A0A069GC35** | Succinate dehydrogenase flavoprotein subunit | sdhA | 4,463E+09 | 1,000E+00 |
| **A0A069GEF2** | PF06103 family protein | CO98_1067 | 2,514E+08 | 1,000E+00 |
| **A0A077UBG8** | General stress protein-like protein | ytxG | 1,615E+08 | 1,000E+00 |
| **A0A077UG21** | Component of the preprotein translocase | yajC | 3,075E+09 | 1,000E+00 |
| **A0A0B6XKR3** | UDP-D-quinovosamine 4-dehydrogenase;Capsular polysaccharide biosynthesis protein Cap5D, putative | capD_1;ERS092844_01182 | 9,129E+07 | 1,000E+00 |
| **A0A0B6XLP0** | Azaleucine resistance protein AzlC;AzlC family protein;Uncharacterized protein | ygaZ;BN1321_430100;NWMN_0009 | 2,319E+08 | 1,000E+00 |
| **A0A0D1FYV9** | Glycerol-3-phosphate cytidylyltransferase | tagD | 1,444E+08 | 1,000E+00 |
| **A0A0D1GCF8** | Beta-lactamase domain-containing protein;Metallo-beta-lactamase;Hydroxyacylglutathione hydrolase | ACR58_00915;BN1321_260183;ERS179246_01030 | 5,690E+07 | 1,000E+00 |
| **A0A0D1H6D2** | Transport permease protein | tagG;AL077_08370;HMPREF0776_1 | 3,114E+09 | 1,000E+00 |
| **A0A0D1HE99** | Putative rRNA methylase;SAM-dependent methyltransferase%2C MraW methylase family;Putative RNA methylase | ACR58_09085;ERS179246_01518;ytqB | 7,843E+07 | 1,000E+00 |
| **A0A0D1I100** | Guanine permease;Uncharacterized protein;Hypoxanthine/guanine permease | pbuG;SAOUHSC_02516 | 1,674E+09 | 1,000E+00 |
| **A0A0D1I4H7** | Putative ribitol-5-phosphate dehydrogenase;SA0242 protein | OO23_0205575;tarJ;SA024 | 1,400E+08 | 1,000E+00 |
| **A0A0D1IFH5** | Peptidase family U32;MW1562 protein;Peptidase, U32 family | ACH32_00905;MW1562;SACOL1667 | 1,679E+08 | 1,000E+00 |
| **A0A0D1J6Y7** | Glucarate transporter;Putative transporter protein;MFS family major facilitator transporter, glucarate:cation symporter | ACH32_05960;BN1326_150015 | 6,968E+07 | 1,000E+00 |
| **A0A0D1JI26** | Uncharacterized protein | ACR58_03075;SAOUHSC_01123;BN1326_60280 | 1,995E+08 | 1,000E+00 |
| **A0A0D1JL38** | Lipoprotein;Putative cytosolic protein;Uncharacterized protein | QU38_09485;ST398NM01_1487;BN1326_70047 | 3,190E+07 | 1,000E+00 |
| **A0A0D1JR08** | Membrane protein;Tellurite resistance protein terC;TerC | ACH32_11950;SAKOR_00939;ST398NM01_1017 | 9,708E+08 | 1,000E+00 |
| **A0A0D1JWB0** | Uncharacterized protein | QU38_16700 | 6,489E+07 | 1,000E+00 |
| **A0A0D1K059** | Phage infection protein;ABC-2 transporter family protein;ABC-2 family transporter protein | QU38_07620;BN1321_400025 | 8,824E+08 | 1,000E+00 |
| **A0A0D1K2Q2** | Cytosolic protein containing cobalamin binding site | ACH32_02625 | 1,029E+08 | 1,000E+00 |
| **A0A0D1K4U9** | Uncharacterized protein | ACH32_10575;BN1326_50155 | 8,002E+07 | 1,000E+00 |
| **A0A0D3Q479** | ABC transporter permease;Uncharacterized protein;Putative membrane protein | AFO87_05020;BN1321_80069;BN1326_30218 | 1,274E+08 | 1,000E+00 |
| **A0A0D3Q4R0** | Deacetylase SIR2;SIR2 family protein | AFO87_05140;ERS179246_01076;ST398NM01_0406 | 5,953E+07 | 1,000E+00 |
| **A0A0D3Q617** | Uncharacterised protein;Uncharacterized protein | AFO87_04680;MW0918;HMPREF0769_12748 | 1,650E+08 | 1,000E+00 |
| **A0A0D3QA44** | Conserved domain protein%2C putative;Uncharacterized protein | AFO87_12755;CH51_11955;NWMN_2113 | 2,791E+08 | 1,000E+00 |
| **A0A0D3QBB6** | Na+/H+ antiporter NhaC;Putative Na+/H+ antiporter;Sodium:proton antiporter OS | nhaC | 1,090E+08 | 1,000E+00 |
| **A0A0D6G261** | Glycosyltransferase;MW0666 protein;SA0659 protein;Glycosyltransferase, group 2 family protein OS | yfdH;MW0666;SA0659 | 3,354E+08 | 1,000E+00 |
| **A0A0D6GGT6** | ATP synthase subunit c | ktrB_1;QU38_11725;ktrD | 3,688E+08 | 1,000E+00 |
| **A0A0D6GHQ4** | Membrane protein;Uncharacterized protein;Membrane protein, putative | AFO87_07485;NWMN_0172;SAOUHSC_00200 | 1,917E+09 | 1,000E+00 |
| **A0A0D6GJI1** | Capsular polysaccharide synthesis enzyme Cap8N;UDP-N-acetyl-D-quinovosamine 4-epimerase;Cap8N | capN;SAKOR_00142;cap8N | 1,350E+08 | 1,000E+00 |
| **A0A0D6H0U4** | 3-hydroxyacyl-CoA dehydrogenase;Uncharacterized protein | AFO87_00695;SA2138;ACH32_05125 | 5,215E+07 | 1,000E+00 |
| **A0A0D6HBS7** | Aliphatic amidase amiE;Hydrolase, carbon-nitrogen family;Beta-ureidopropionase | amiE;HMPREF0769_11128;ST398NM01_207 | 7,023E+07 | 1,000E+00 |
| **A0A0D6HVU1** | Phosphoribosylamine--glycine ligase | purD | 4,156E+07 | 1,000E+00 |
| **A0A0D6HZA6** | Carboxylesterase;MW0741 protein | est_2;SAKOR_00783;est | 1,833E+08 | 1,000E+00 |
| **A0A0E0VL70** | SgaT;PTS system, L-ascorbate specific IIC component ulaA;Putative membrane protein | ST398NM01_0410;SAKOR_00324;BN13 | 1,437E+08 | 1,000E+00 |
| **A0A0E0VN96** | Antibacterial protein 3 | ST398NM01_1170;psm | 1,973E+09 | 1,000E+00 |
| **A0A0E0VQS2** | Multidrug resistance protein;Multidrug MFS transporter;Transporter, major facilitator family protein | ST398NM01_1812;yttB | 9,749E+07 | 1,000E+00 |
| **A0A0E0VTN7** | Anaerobic ribonucleoside-triphosphate reductase;Anaerobic ribonucleosid | ST398NM01_2664;ACH32_06515 | 3,993E+07 | 1,000E+00 |
| **A0A0E1AG73** | Putative hydrolase;Uncharacterized protein;Hydrolase | SAZ172_1387;MW1265;ERS179246_01232 | 1,815E+08 | 1,000E+00 |
| **A0A0E1AGR6** | Uncharacterized protein;ABC transporter permease protein;Binding-protein-dependent transport system inner membrane protein | SAZ172_0183;ssuC | 1,669E+09 | 1,000E+00 |
| **A0A0E1AJI7** | Oligopeptide transporter putative membrane permease domain protein;NikC;Peptide ABC transporter permease | opp-1C;ST398NM01_2515 | 1,528E+08 | 1,000E+00 |
| **A0A0E1AJK2** | Uncharacterized protein;Uncharacterized protein SAOUHSC_02783;Tandem lipoprotein | SAZ172_2583;SAOUHSC_02783 | 1,694E+08 | 1,000E+00 |
| **A0A0E1AKC0** | Uncharacterized protein;YlmH protein;RNA binding protein | SAZ172_1193;ylmH;ERS179246_02049 | 7,011E+07 | 1,000E+00 |
| **A0A0E1AKG7** | Release factor glutamine methyltransferase | prmC | 5,811E+07 | 1,000E+00 |
| **A0A0E1AKM3** | Heme ABC type transporter HtsABC, permease protein HtsC;Cobalamin Fe3+-siderophores ABC transporter permease | SAZ172_2276;fecD | 9,374E+07 | 1,000E+00 |
| **A0A0E1AM31** | Glycosyltransferase Gtf1;Glycosyltrans | gtf1;gftA | 4,669E+07 | 1,000E+00 |
| **A0A0E1APM1** | Fibronectin binding protein FnbA;Fibronectin-binding protein A | fnbA | 9,698E+07 | 1,000E+00 |
| **A0A0E1VHB5** | 3-dehydroquinate synthase | aroB | 3,188E+08 | 1,000E+00 |
| **A0A0E1VJC0** | Oxidoreductase, short chain dehydrogenase/reductase family protein;Short chain dehydrogenase | HMPREF0776_2527;SAZ172_1515 | 1,745E+08 | 1,000E+00 |
| **A0A0E1VMF5** | Uncharacterized protein;ATP-grasp domain protein | HMPREF0776_1412;AFO87_12885;HMPRE | 4,133E+07 | 1,000E+00 |
| **A0A0E1VQD3** | Uncharacterized protein;Integral membrane protein | HMPREF0776_0412;AFO87_00635;BN1321_400004 | 1,558E+09 | 1,000E+00 |
| **A0A0E1XC35** | Molybdenum cofactor biosynthesis protein A;Cyclic pyranopterin monophosphate synthase | moaA | 2,984E+07 | 1,000E+00 |
| **A0A0E7DYF4** | Staphylococcus aureus paralogous family;Uncharacterized protein | AFO87_11285;SAOUHSC_00585;MW0552 | 1,622E+09 | 1,000E+00 |
| **A0A0E7Y8C1** | ACT domain protein;Prephenate dehydratase;Prephenate dehydratase | pheA;BN1321_320008;HMPREF0769_11255 | 8,776E+07 | 1,000E+00 |
| **A0A0E8G8W5** | Enterotoxin I;Staphylococcal enterotoxin Q;Staphylococcal enterotoxin type I | entQ;CH51_04275;seq | 1,114E+08 | 1,000E+00 |
| **A0A0E8GAK5** | Ferrichrome transport system permease protein fhuG | fhuG | 2,137E+08 | 1,000E+00 |
| **A0A0E8GLL3** | Uncharacterised protein | ERS092844_00944 | 6,570E+07 | 1,000E+00 |
| **A0A0E8H1H6** | Lipoate--protein ligase | lplJ_1;HMPREF0776_1072 | 4,112E+07 | 1,000E+00 |
| **A0A0E8HMR4** | Molybdenum cofactor biosynthesis protein B | moaB | 7,437E+07 | 1,000E+00 |
| **A0A0G2LRX1** | Histidine kinase;Two-component sensor histidine kinase | CH51_06940;desK | 3,508E+08 | 1,000E+00 |
| **A0A0H2AM14** | Uncharacterised protein;Uncharacterized protein | AFO87_05335;NWMN_0227;MW0267 | 1,937E+08 | 1,000E+00 |
| **A0A0H2DL58** | Uncharacterised protein | AL493_15205 | 3,009E+08 | 1,000E+00 |
| **A0A0H2F9P7** | Membrane protein;Uncharacterized protein | AFO87_06755;SAZ172_2710;QU38_16180 | 5,030E+08 | 1,000E+00 |
| **A0A0H2WXU5** | Peptide ABC transporter, permease protein;Membrane protein | SACOL0185;gsiC;QU38_14790 | 7,319E+08 | 1,000E+00 |
| **A0A0H2XDV0** | Uncharacterized protein;Putative cytosolic protein | SAUSA300_0294;AFO87_05300;HMPREF0 | 5,745E+07 | 1,000E+00 |
| **A0A0H2XFJ7** | Transporter gate domain protein;Membrane protein;Putative membrane spanning protein | SAUSA300_2133;AFO87_12560;SAKOR_02 | 5,624E+08 | 1,000E+00 |
| **A0A0H2XGP6** | PTS system, mannitol specific IIA component;Mannitol-specific phosphotransferase enzyme IIA component | mtlA;mtlF | 1,379E+08 | 1,000E+00 |
| **A0A0H2XH23** | Putative lipoprotein;Lipoprotein;Uncharacterized protein | SAUSA300_2355;AFO87_00365;SAOUHSC_02695 | 3,898E+08 | 1,000E+00 |
| **A0A0H2XI08** | Alcohol dehydrogenase, zinc-containing;Alcohol dehydrogenase | SAUSA300_0055;flhA | 2,612E+08 | 1,000E+00 |
| **A0A0H3JL25** | Uncharacterized protein;Branched-chain amino acid transport protein | SA0609;HMPREF0776_1657 | 2,997E+09 | 1,000E+00 |
| **A0A0H3JL37** | SA0628 protein;Sugar efflux transpoter;Sugar efflux transporter | SA0628;SAZ172_0685;ST398NM01_0751 | 2,402E+08 | 1,000E+00 |
| **A0A0H3JLG2** | Uncharacterized protein;Uncharacterised protein | SA0377;ST398NM01_0484;AFO87_08885 | 1,915E+08 | 1,000E+00 |
| **A0A0H3JPF1** | Uncharacterized protein;Membrane protein;Putative membrane protein | SA2483;AFO87_13965;BN1321_430068 | 1,357E+09 | 1,000E+00 |
| **A0A0H3JPI5** | SA0871 protein;Uncharacterized protein;Sodium/proton-dependent alanine transporter | SA0871;SAOUHSC_00949;yrbD | 1,038E+08 | 1,000E+00 |
| **A0A0H3JPP1** | NPQTN specific sortase B;Sortase, SrtB family | srtB | 1,060E+08 | 1,000E+00 |
| **A0A0H3JQ67** | Glycerol-3-phosphate transporter;Glycerol-3-phosphate ABC transporter | glpT | 1,760E+09 | 1,000E+00 |
| **A0A0H3JTB1** | Uncharacterized protein;Transporter, DASS family;DASS family divalent anion:sodium (Na+) symporter | SA0645;HMPREF0776_1694 | 2,705E+08 | 1,000E+00 |
| **A0A0H3JUT0** | Uncharacterized protein;Uncharacterized conserved protein | MW0277;ERS195423_01553;AFO87_05280 | 4,744E+07 | 1,000E+00 |
| **A0A0H3JV25** | Uncharacterized protein;Membrane protein | MW0420;ACH32_09500;SA0424 | 1,544E+08 | 1,000E+00 |
| **A0A0H3JV73** | Octanoyl-[GcvH]:protein N-octanoyltransferase;Octanoyl-[GcvH]:protein N-octanoyltransferase | lipL | 1,008E+08 | 1,000E+00 |
| **A0A0H3JVB3** | MW0257 protein;SA0270 protein;Staphyloxanthin biosynthesis protein | MW0257;SA0270;QU38_14380 | 6,810E+07 | 1,000E+00 |
| **A0A0H3JYD3** | Uncharacterized protein;Fructosamine kinase;Ribulosamine/erythrulosamine 3-kinase potentially involved in protein deglycation | MW2508;AFO87_06765 | 1,329E+08 | 1,000E+00 |
| **A0A0H3JYJ9** | MW2093 protein;Multidrug MFS transporter;Multidrug resistance protein B | MW2093;emrB_1;emrB_3 | 5,309E+07 | 1,000E+00 |
| **A0A0H3JYW6** | Uncharacterized protein;Acetyltransferase;GNAT family acetyltransferase | MW2324;ytmI | 4,319E+07 | 1,000E+00 |
| **A0A0H3JZ35** | MW2432 protein;SA2301 protein;Alkaline phosphatase | MW2432;SA2301;ACH32_05965 | 5,307E+08 | 1,000E+00 |
| **A0A0H3JZ54** | Uncharacterized protein | MW2455;SAZ172_2631;SAOUHSC_02 | 4,461E+08 | 1,000E+00 |
| **A0A0H3JZ55** | Uncharacterized protein;3-beta hydroxysteroid dehydrogenase | MW0620;AFO87_03105;RU53_720 | 5,592E+08 | 1,000E+00 |
| **A0A0H3K0K9** | Riboflavin biosynthesis protein RibD | ribD;CH51_09495 | 1,325E+08 | 1,000E+00 |
| **A0A0H3K193** | Uncharacterized protein;Transporter, major facilitator family protein;Multidrug MFS transporter | MW2184;HMPREF0776_0313 | 1,932E+08 | 1,000E+00 |
| **A0A0H3K1Q0** | MW0188 protein;M23/M37 peptidase domain protein;Peptidase M23;Peptidase family M23 O | MW0188;SAZ172_0212;lytM_2 | 2,222E+08 | 1,000E+00 |
| **A0A0H3K4A6** | MW2266 protein;Sodium/glutamate symporter;Sodium:glutamate symporter | MW2266;gltS;ACH32_05110 | 1,032E+09 | 1,000E+00 |
| **A0A0H3K7E4** | Ferrichrome transport permease fhuB;Ferrichrome ABC transporter (Permease);Iron (Fe3+) ABC superfamily ATP binding cassette tra | fhuB;feuB | 9,241E+07 | 1,000E+00 |
| **A0A0H3K8M8** | Uncharacterized protein | NWMN_1353;MW1332;ACR58_00050 | 2,953E+08 | 1,000E+00 |
| **A0A0H3K9T0** | ABC transporter, ATP-binding protein;ABC transporter ATP-binding protein;ABC superfamily ATP binding cassette transporter, ABC p | NWMN_2055;metN_2 | 4,953E+07 | 1,000E+00 |
| **A0A0H3KDG3** | Capsular polysaccharide biosynthesis protein CapP;UDP-N-acetylglucosamine 2-epimerase | capP;HMPREF0776_0900 | 1,025E+08 | 1,000E+00 |
| **A0A0K6ZPG0** | Zn-dependent hydrolase (Beta-lactamase superfamily);Metallohydrolase;Uncharacterized protein | ERS365775_01675;AFO87_14245;ST398NM01_0 | 6,945E+07 | 1,000E+00 |
| **A0A0M1TWJ4** | Uncharacterized protein | RU53_15;MW0013;SAZ172 | 1,352E+08 | 1,000E+00 |
| **A0A0M1TWM0** | ABC-2 family transporter protein;Uncharacterized protein | RU53_256;MW0255;S | 7,640E+08 | 1,000E+00 |
| **A0A0M1TXD5** | Putative sodium/glucose cotransporter;MW0291 protein;SA0303 protein | RU53_289;MW0291;SA0303 | 1,378E+08 | 1,000E+00 |
| **A0A0M1TXN4** | Sa tandem lipoprotein;Lipoprotein;Uncharacterized lipoprotein SAUSA300_0410 | lpl1_1;lpl9_4;SAUSA300_0410 | 1,043E+08 | 1,000E+00 |
| **A0A0M1TY95** | Uncharacterized protein | RU53_654;BN1326_50091;SAOUHSC_0059 | 7,827E+07 | 1,000E+00 |
| **A0A0M1TYL8** | Uncharacterized protein;Membrane protein | RU53_738;AFO87_03010;ACH32_10700 | 4,178E+08 | 1,000E+00 |
| **A0A0M1TZ82** | Anthranilate synthase;Aminobenzoate synthetase;Para-aminobenzoate synthetase component I | pabB | 7,213E+07 | 1,000E+00 |
| **A0A0M1U1R9** | Toxin regulatory protein;SA1676 protein;PTS system, IIC component | RU53_1960;SA1676;AL077_07310 | 1,782E+09 | 1,000E+00 |
| **A0A0M1U1V5** | Uncharacterized protein;Uncharacterised protein | RU53_2022;CH51_10300;ERS179246_02390 | 4,670E+07 | 1,000E+00 |
| **A0A0M1U279** | Iron compound ABC transporter permease;Cobalamin Fe3+-siderophores ABC transporter permease;Uncharacterized protein | RU53_2244;htsB | 6,103E+08 | 1,000E+00 |
| **A0A0M1U338** | ABC superfamily ATP binding cassette transporter, ABC/membrane protein;SA2216 protein | RU53_2490;SA2216 | 2,485E+08 | 1,000E+00 |
| **A0A0M1U353** | MFS family major facilitator transporter;Integral membrane efflux protein;MW2368 protein | RU53_2508;NWMN_2343 | 1,846E+09 | 1,000E+00 |
| **A0A0S3DYH0** | Uncharacterized protein | BN1321_170073;MW0701;HMPREF0769_11554 | 3,906E+07 | 1,000E+00 |
| **A0A0T7M2E8** | Acetyltransferase;Putative acetyltransferase | ERS093009_00097;ACR58_11465;BN1326_50102 | 1,239E+08 | 1,000E+00 |

| | | | | |
|---|---|---|---|---|
| **A0A0T7M7L5** | Membrane protein;Uncharacterized protein;Putative membrane protein | ERS093009_01925;BN1321_80002;BN1326_30139 | 9,933E+07 | 1,000E+00 |
| **A0A0T7M7R2** | Oligopeptide transport system permease protein;MW0183 protein;SA0200 protein | gsiD_2;MW0183;SA0200 | 2,824E+08 | 1,000E+00 |
| **A0A0T7M7U6** | 2-isopropylmalate synthase | leuA_2;leuA | 6,163E+06 | 1,000E+00 |
| **A0A0T7M9H7** | Putative deoxyribonuclease YcfH;Hydrolase, TatD family;DNase, TatD family | ycfH;HMPREF0776_1340;ACH32_09660 | 1,668E+08 | 1,000E+00 |
| **A0A0T7MUK5** | ABC transporter permease;Uncharacterized protein;ABC-2 transporter family protein | ERS179246_01102;SAOUHSC_00334;ASU36_13395 | 1,333E+09 | 1,000E+00 |
| **A0A0T7MW40** | Uncharacterised protein;Uncharacterized protein;Un | ERS179246_01728;SA0090;SAZ172_0111 | 9,070E+08 | 1,000E+00 |
| **A0A0T7MXQ6** | Phage membrane protein;Uncharacterized protein | ERS179246_02309;CH51_10715;ERS365775_02601 | 2,114E+08 | 1,000E+00 |
| **A0A0T7MY12** | Citrate transporter;CitMHS family citrate-magnesium (Mg2+):proton (H+) citrate-calcium (Ca2+):proton (H+) symporter;MW2538 pro | citN;citM | 1,572E+09 | 1,000E+00 |
| **A0A0U0TXD8** | Lipoprotein;Uncharacterized protein | ERS195423_01708;AFO87_04020;SAKOR_02684 | 1,655E+08 | 1,000E+00 |
| **A0A0U0U0N7** | Membrane lipoprotein;Uncharacterized lipoprotein SAUSA300_0417;Lipoprotein | lpl9_4;SAUSA300_0417;lpl9_1 | 6,795E+08 | 1,000E+00 |
| **A0A0U0U2M9** | Staphylococcal tandem lipoprotein;Uncharacterized lipoprotein SACOL0481;Uncharacterized lipoprotein SAUSA300_0414 | lpl9_2;SACOL0481 | 1,001E+08 | 1,000E+00 |
| **A0A0U0USQ1** | UDP-sugar hydrolase%3B 5-nucleotidase;Bifunctional 5-nucleotidase/2,3-cyclic phosphodiesterase;5-nucleotidase | yhcR;RU53_919; | 8,268E+07 | 1,000E+00 |
| **A0A0U0V0S8** | Acetyltransferase;N-acetyltransferase GCN5;Acetyltransferase, GNAT family | bltD;RU53_431;HMPREF0769_10016 | 6,265E+07 | 1,000E+00 |
| **A0A0U1MD67** | Putative Oligopeptide transporter membrane permease;Oligopeptide transporter putative membrane permease domain protein | BN1326_140266;opp-1B | 3,045E+07 | 1,000E+00 |
| **A0A0U1MDU5** | N-acetylmuramoyl-L-alanine amidase domain-containing protein SAOUHSC_02979 | BN1326_150163 | 7,616E+07 | 1,000E+00 |
| **A0A0U1MEU0** | Transcriptional regulator (GntR family);GntR family transcriptional regulator;Gluconate operon transcriptional repressor | gntR;AUC48_12780;ydfH | 1,603E+08 | 1,000E+00 |
| **A0A0U1MF79** | Uncharacterized protein;Membrane protein | BN1321_150075;MW0597;ACR58_11625 | 2,853E+08 | 1,000E+00 |
| **A0A0U1MFQ7** | ABC transporter permease protein;Uncharacterized protein | BN1326_150208;MW2602;SA2475 | 5,516E+07 | 1,000E+00 |
| **A0A0U1MG12** | Putative PTS multidomain regulator;Transcription antiterminator, BglG family;Bacterial regulatory s, luxR family protein | BN1326_30200;SAKOR_00327 | 7,132E+06 | 1,000E+00 |
| **A0A0U1MGK2** | PTS-dependent dihydroxyacetone kinase, phosphotransferase subunit dhaM;PTS mannnose transporter subunit IIA | dhaM;ACR58_11710 | 2,486E+08 | 1,000E+00 |
| **A0A0U1MHE1** | ABC transporter, substrate-binding protein, family 5;Oligopeptide ABC transporter substrate-binding protein+ | BN1321_200025;RU53_986 | 2,260E+08 | 1,000E+00 |
| **A0A0U1MHH0** | Uncharacterized protein;Membrane protein | BN1321_200019;ERS092844_00420;ST398NM01_0981 | 5,047E+08 | 1,000E+00 |
| **A0A0U1MHR5** | Acyltransferase family protein;Putative acetyltransferase;Uncharacterized protein | BN1321_220013;RU53_1047 | 2,471E+08 | 1,000E+00 |
| **A0A0U1MI94** | Uncharacterized protein;Protein of hypothetical function DUF72 | yunF;yecE;HMPREF0776_1910 | 1,229E+08 | 1,000E+00 |
| **A0A0U1MIL4** | Putative membrane protein;Transporter, drug/metabolite exporter family protein;Triose-phosphate Transporter family protein | BN1326_50233;SAKOR_00729 | 3,676E+08 | 1,000E+00 |
| **A0A0U1MJW8** | Putative cytochrome bd menaquinol oxidase subunit I;Cytochrome d ubiquinol oxidase subunit I;Cytochrome ubiquinol oxidase | ythA;cydA | 4,929E+08 | 1,000E+00 |
| **A0A0U1MKY3** | KR domain protein;Putative 3-oxoacyl-acyl carrier protein reductase;Acetoacetyl-CoA reductase | BN1321_240128;RU53_1279 | 7,824E+07 | 1,000E+00 |
| **A0A0U1ML00** | Serine/threonine exchanger SteT;Amino acid permease;Serine/threonine | steT;SACOL1476;ACR58_00015 | 1,341E+08 | 1,000E+00 |
| **A0A0U1ML69** | DNA polymerase III PolC-type | polC | 1,048E+08 | 1,000E+00 |
| **A0A0U1MLF1** | Segregation and condensation protein A | scpA | 7,561E+07 | 1,000E+00 |
| **A0A0U1MLK1** | Thermonuclease;Micrococcal nuclease;Nuclease-like protein | nucI;nuc;HMPREF0769_12423 | 1,605E+09 | 1,000E+00 |
| **A0A0U1MLS4** | Putative ABC transport system permease protein;Spermidine/putrescine ABC transporter, permease | BN1326_60216;BN1321_230022 | 1,027E+09 | 1,000E+00 |
| **A0A0U1MM79** | Putative membrane protein | BN1326_110014 | 9,082E+07 | 1,000E+00 |
| **A0A0U1MQ49** | Glycine betaine transporter;Choline transport protein BetT;MW2111 protein | opuD;betT;MW2111 | 1,936E+09 | 1,000E+00 |
| **A0A0U1MRA3** | Putative permease of an ABC transporter;ABC superfamily ATP binding cassette transporter, membrane protein;Conserved hypotet | ybbM;RU53_2517 | 2,097E+08 | 1,000E+00 |
| **A0A0U1MRL7** | Molybdopterin biosynthesis adenylyltransferase;Molybdopterin biosynthesis MoeB protein;Thiamine/molybdopterin biosynthesis Th | moeB;moeB_2 | 1,185E+08 | 1,000E+00 |
| **A0A0U1MRN8** | Potassium/proton-divalent cation antiporter;Cation diffusion facilitator family transporter;Cation transporter | czcD;czrB | 1,114E+08 | 1,000E+00 |
| **A0A0U1MSJ3** | Aminoacyltransferase FemA;FemAB family peptidoglycan biosynthesis protein;FmhA protein | femA;fmhA | 2,500E+07 | 1,000E+00 |
| **A0A0U1MSP2** | Glycine betaine/carnitine/choline/choline sulfate ABC transporter (Permease);ABC transporter, permease protein;Glycine/betaine A | opuCB;HMPREF0769_10698 | 6,860E+08 | 1,000E+00 |
| **A0A0U1MSP9** | Staphylococcal secretory antigen ssaA2 | ssaA;ssaA2 | 1,849E+09 | 1,000E+00 |
| **A0A0U1MTV5** | 2-dehydropantoate 2-reductase | BN1321_420101;MW2519;SA2393 | 1,840E+08 | 1,000E+00 |
| **A0A0U1MU98** | HTH-type transcriptional regulator NorG;Bacterial regulatory s%2C gntR family protein;Uncharacterized protein | norG;SAKOR_00082 | 3,403E+07 | 1,000E+00 |
| **A0A0U1MUD9** | Uncharacterized protein;Adenine nucleotide alpha hydrolases superfamily protein | BN1321_420094;AFO87_06750;BN1326_150097 | 8,880E+07 | 1,000E+00 |
| **A0A0U1MUE8** | Proton/sodium-glutamate symport protein;Proton/sodium-glutamate symporter;Glutamate-aspartate carrier protein | gltT;NWMN_2284 | 4,445E+09 | 1,000E+00 |
| **A0A0U1MUG2** | Amino acid permease family protein;Amino acid permease | BN1321_420104;BN1326_150108;HMPREF0769_10549 | 1,553E+08 | 1,000E+00 |
| **A0A0U1MUH8** | Arabinogalactan oligomer permease;Maltose/maltodextrin ABC transporter%2C permease protein MalF | ganP;malF | 1,012E+08 | 1,000E+00 |
| **A0A0U1MVW6** | Putative membrane protein;Low temperature requirement protein A | BN1326_30221;CH51_01695;AFO87_05005 | 1,014E+08 | 1,000E+00 |
| **A0A0U1MXG7** | Methionine ABC transporter, permease component;ABC transporter permease protein;Uncharacterized protein | metP;metI;SA0770 | 1,075E+09 | 1,000E+00 |
| **A0A0U1MXU3** | UDP-N-acetylglucosamine:undecaprenyl-P N-acetylglucosaminyl-1-P transferase;Undecaprenyl-phosphate N-acetylglucosaminyl 1-pl | tagO;llm | 5,256E+08 | 1,000E+00 |
| **A0A0U1MY64** | ABC-2 transporter family protein;Uncharacterized protein | BN1321_70020;MW0254;SA0267 | 5,954E+08 | 1,000E+00 |
| **A0A0U1N0H6** | Putative lantibiotic ABC transporter protein;Lantibiotic ABC transporter permease | BN1326_90051;AFO87_00060 | 1,676E+09 | 1,000E+00 |
| **A6QFG0** | Na(+)/H(+) antiporter subunit C1;ShaC;Na(+) H(+) antiporter subunit C | mnhC1;ST398NM01_0933;mnhC | 1,494E+09 | 1,000E+00 |
| **B6V385** | Putative exported protein;Uncharacterized protein;Transposon-related protein | AFO87_14660;CH51_06835;AN169_05145 | 1,080E+09 | 1,000E+00 |
| **C4B834** | Coagulase;Staphylocoagulase repeat protein | coa;HMPREF0776_ | 3,453E+07 | 1,000E+00 |
| **D2J6A6** | Bacitracin ABC ATP binding cassette transporter%2C membrane protein | AL078_10275 | 1,788E+08 | 1,000E+00 |
| **D7URR6** | Membrane protein;Uncharacterized protein | AFO87_12985;AL078_01705;QU38_08675 | 7,968E+08 | 1,000E+00 |
| **O34091** | Uroporphyrinogen III synthase;Uroporphyrinogen III synthase O | hemD;CH51_08970 | 7,990E+07 | 1,000E+00 |
| **P0A071** | Gamma-hemolysin component A;Gamma-hemolysin subunit A | hlgA;QU38_07850;hlgA_1 | 3,636E+07 | 1,000E+00 |
| **P0A0M2** | Delta-hemolysin;Delta hemolysin | hld | 5,156E+10 | 1,000E+00 |
| **P0A0N0** | DegV domain-containing protein SAV0749;DegV family protein | SAV0749;SAKOR_00751 | 4,150E+07 | 1,000E+00 |
| **P0C7Y5** | Phenol-soluble modulin alpha 1 peptide;Phenol-soluble modulin alpha 2 peptide | psmA1;psmA2 | 5,499E+09 | 1,000E+00 |
| **P63820** | Phosphopantetheine adenylyltransferase | coaD | 7,264E+07 | 1,000E+00 |
| **P65267** | Lipoprotein signal peptidase;Signal peptidase II | lspA | 8,431E+08 | 1,000E+00 |
| **Q2FH64** | Tryptophan synthase beta chain | trpB | 1,546E+07 | 1,000E+00 |
| **Q2FHI6** | Tyrosine recombinase XerC | xerC | 5,356E+07 | 1,000E+00 |
| **Q2FI19** | Probable quinol oxidase subunit 3;Cytochrome aa3-600 quinol oxidase (Subunit III) | qoxC | 2,068E+07 | 1,000E+00 |
| **Q2FIC9** | Na(+)/H(+) antiporter subunit G1;ShaG | mnhG1;mnhG;ST398NM01_0929 | 2,107E+09 | 1,000E+00 |
| **Q2FK78** | N-acetyl-gamma-glutamyl-phosphate reductase | argC | 3,762E+07 | 1,000E+00 |
| **Q2FKF9** | Uncharacterized lipoprotein SAUSA300_0102;Staphylococcus tandem lipoproteins;Uncharacterized lipoprotein SACOL0081/SACOL0( | SAUSA300_0102;AFO87_03770 | 8,675E+08 | 1,000E+00 |
| **Q2FUR5** | Permease, putative;VraE protein;ABC transporter permease | SAOUHSC_03037;vraE;AL493_01245 | 1,152E+08 | 1,000E+00 |
| **Q2FUW5** | Accessory Sec system protein Asp3;Accessory secretory protein Asp3 | asp3;NWMN_2549;as | 6,632E+07 | 1,000E+00 |
| **Q2FVB0** | Uncharacterized protein;Putative membrane protein | SAOUHSC_02823;AUC48_12850;BN1326_150022 | 3,407E+08 | 1,000E+00 |
| **Q2FVL1** | Uncharacterized protein;Drug resistance transporter EmrB/QacA subfamily protein | SAOUHSC_02700;NWMN_2314 | 5,359E+08 | 1,000E+00 |
| **Q2FVU1** | Na+/H+ antiporter, putative;Na+/H+ antiporter family protein;NhaC family sodium:proton (Na+:H) antiporter | SAOUHSC_02601;BN1321_380165 | 1,259E+09 | 1,000E+00 |
| **Q2FVX3** | Sulfurtransferase FdhD | fdhD | 1,160E+08 | 1,000E+00 |
| **Q2FVY4** | Probable molybdenum cofactor guanylyltransferase;Molybdopterin-guanine dinucleotide biosynthesis protein A | mobA;CH51_12245 | 7,284E+07 | 1,000E+00 |
| **Q2FXD3** | Uncharacterized protein;Exported protein | SAOUHSC_01930;AFO87_09085;SAKOR_01753 | 1,193E+08 | 1,000E+00 |
| **Q2FXI1** | Uncharacterized protein;Choline kinase;Phosphotransferase enzyme family | SAOUHSC_01866;AFO87_06950;HMPREF0 | 5,180E+07 | 1,000E+00 |
| **Q2FZA8** | Uncharacterized protein;Arginine/ornithine antiporter;ArcD-like protein | SAOUHSC_01130;SAKOR_01092;arcD-like | 3,003E+08 | 1,000E+00 |
| **Q2FZX9** | Probable nitronate monooxygenase;Enoyl-(Acyl-carrier-protein) reductase (FMN);2-nitropropane dioxygenase | SAOUHSC_00855;SAZ172_0862 | 9,813E+07 | 1,000E+00 |
| **Q2G129** | Uncharacterized protein;Putative high-affinity Fe2+/Pb2+ permease-like protein;High-affinity iron permease | SAOUHSC_00327;RU53_320 | 4,450E+07 | 1,000E+00 |
| **Q2G165** | Uncharacterized protein;Regulatory protein;Perfringolysin O regulator protein | SAOUHSC_00290;pfoR_1;pfoR | 2,641E+08 | 1,000E+00 |
| **Q2G1P5** | Uncharacterized protein;SA0099 protein;Antibiotic resistance-related transmembrane efflux protein | SAOUHSC_00058;SA0099; | 4,442E+08 | 1,000E+00 |
| **Q2G1T1** | Uncharacterized protein;Phosphoglycerate mutase family protein | SAOUHSC_00827;gpmA1 | 5,659E+07 | 1,000E+00 |
| **Q2G256** | CCA-adding enzyme | SAOUHSC_01474;cca;papS | 5,913E+07 | 1,000E+00 |
| **Q2G2I3** | Uncharacterized protein;Drug resistance transporter, Bcr/CflA family protein;Bicyclomycin transporter TcaB | SAOUHSC_02633;SAKOR_02330 | 2,550E+08 | 1,000E+00 |
| **Q53645** | Fructokinase;Kinase, PfkB family | iolC;HMPREF0776_0189;SAZ172_2040 | 1,780E+07 | 1,000E+00 |
| **Q5HDD3** | Gamma-hemolysin component B | hlgB | 2,686E+08 | 1,000E+00 |
| **Q5HDT6** | Molybdopterin synthase catalytic subunit;Molybdopterin synthase (Large subunit);Molybdenum cofactor biosynthesis protein E | moaE | 6,088E+07 | 1,000E+00 |
| **Q5HHC0** | Inactive signal peptidase IA;Signal peptidase I | spsA;lepB | 3,051E+08 | 1,000E+00 |
| **Q5HHD6** | Na(+)/H(+) antiporter subunit D1 | mnhD1 | 1,106E+09 | 1,000E+00 |
| **Q5HHD7** | Na(+)/H(+) antiporter subunit E1;Non essential component of Na+/H+ antiporter;Na(+) H(+) antiporter subunit E | mnhE1;mrpE;mnhE | 1,384E+09 | 1,000E+00 |
| **Q5HHN9** | Probable protein-export membrane protein SecG;Protein translocase subunit SecG | secG;SAOUHSC_00801 | 2,161E+09 | 1,000E+00 |
| **Q5HHU8** | Putative 5(3)-deoxyribonucleotidase;5(3)-deoxyribonucleotidase | SACOL0785;AL493_05230 | 2,448E+08 | 1,000E+00 |
| **Q5HHX4** | Quinolone resistance protein NorA;Multidrug MFS transporter | norA;QU38_105 | 4,858E+08 | 1,000E+00 |
| **Q5HIC3** | Ribulokinase | araB | 4,324E+07 | 1,000E+00 |
| **Q5HIN4** | Uncharacterized lipoprotein SACOL0483;Tandem lipoprotein | SACOL0483;lpl8 | 1,650E+08 | 1,000E+00 |
| **Q5HIN5** | Uncharacterized lipoprotein SACOL0482 | SACOL0482 | 5,471E+07 | 1,000E+00 |
| **Q5HJX8** | Adenylosuccinate synthetase | purA | 5,881E+07 | 1,000E+00 |
| **Q6X7U0** | Diaminopimelate decarboxylase | sbnH;QU38_15175;ST398NM01_01 | 1,731E+07 | 1,000E+00 |
| **Q6X7U6** | 2,3-diaminopropionate biosynthesis protein SbnB;Ornithine cyclodeaminase, putative;Ornithine cyclodeaminase family protein | sbnB;SACOL0101 | 6,829E+07 | 1,000E+00 |
| **Q7A039** | Heme response regulator HssR;Heme transporter CcmC;Two-component response regulator | hssR;QU38_07450;SAKOR_0 | 1,296E+08 | 1,000E+00 |
| **Q7A2Q0** | Sensor protein VraS | vraS;liaS | 2,913E+09 | 1,000E+00 |
| **Q7A3U8** | Probable nitrate transporter NarT;Nitrate transporter NarT | narT;QU38_ | 3,538E+08 | 1,000E+00 |
| **Q7A4E6** | ATP synthase subunit c | atpE;V070_02071 | 7,430E+06 | 1,000E+00 |
| **Q7A5C5** | UPF0365 protein SA1402;UPF0365 protein SAOUHSC_01676 | SA1402;SAOUHSC_01676 | 6,309E+10 | 1,000E+00 |
| **Q7A5Q7** | Putative oligopeptide transport system permease protein oppC2 | oppC2 | 9,770E+07 | 1,000E+00 |
| **Q7A6D2** | Processive diacylglycerol beta-glucosyltransferase | ugtP | 4,139E+07 | 1,000E+00 |
| **Q7A7M5** | UPF0324 membrane protein SA0329;UPF0324 membrane protein SACOL0411;Membrane protein | SA0329;SACOL0411;CH51_01630 | 4,175E+08 | 1,000E+00 |
| **Q8NUH8** | Putative ABC transporter ATP-binding protein MW2603;Putative ABC transporter ATP-binding protein SACOL2708 | MW2603;SACOL2708 | 2,210E+08 | 1,000E+00 |
| **Q8NX68** | Protoheme IX farnesyltransferase | ctaB | 5,746E+06 | 1,000E+00 |
| **Q8NYB7** | Protein-ADP-ribose hydrolase;Macro domain protein | MW0302;SACOL0396;RU5 | 1,904E+08 | 1,000E+00 |
| **Q8NYU1** | Uncharacterized lipoprotein MW0072;Uncharacterized lipoprotein SAOUHSC_00053;Tandem lipoprotein within Pathogenicity island | MW0072;SAOUHSC_00053 | 1,530E+08 | 1,000E+00 |
| **Q931V3** | UPF0413 protein SAV1001;UPF0413 protein SACOL1006;Uncharacterized protein | SAV1001;SACOL1006;H | 6,657E+07 | 1,000E+00 |
| **Q93PN3** | Ferric hydroxamate receptor 1 | fhuD1;BN1321_330035;fhuD | 2,429E+09 | 1,000E+00 |
| **Q99R72** | Glycosyl-4,4-diaponeurosporenoate acyltransferase | crtO | 6,132E+07 | 1,000E+00 |
| **Q99RN7** | Oxygen sensor histidine kinase NreB;Sensor histidine kinase | nreB | 2,584E+08 | 1,000E+00 |
| **Q99S77** | PTS system lactose-specific EIICB component;PTS system, lactose-specific IIB component / PTS system | lacE | 1,690E+08 | 1,000E+00 |

| | | | | |
|---|---|---|---|---|
| **Q99S97** | Multidrug efflux pump SdrM;Multidrug resistance protein B;MW2095 protein | sdrM;bmr3;MW2095 | 1,200E+09 | 1,000E+00 |
| **Q99SC8** | Type II pantothenate kinase | coaW | 1,724E+08 | 1,000E+00 |
| **Q99U80** | Putative branched-chain amino acid carrier protein SAV1407;Branched-chain amino acid transport system carrier protein | SAV1407;BN1321_260047 | 2,288E+08 | 1,000E+00 |
| **Q99W51** | tRNA-specific adenosine deaminase | tadA | 9,715E+07 | 1,000E+00 |
| **Q99WT7** | Virulence factor EsxB | esxB | 1,461E+08 | 1,000E+00 |
| **Q99WT8** | Protein EsaC;Uncharacterized protein | esaC | 2,483E+08 | 1,000E+00 |
| **Q9FDP4** | Membrane protein | ERS092844_02688 | 1,308E+09 | 1,000E+00 |
| **Q9X668** | Na+ dependent nucleoside transporter domain-containing protein;Purine nucleoside transport protein NupG;MW0607 protein | nupG;MW0607 | 4,047E+09 | 1,000E+00 |
| **Q9ZGN7** | Antibiotic resistance protein;MW2386 protein;Transporter, putative | AFO87_06460;MW2386;SACOL2471 | 3,074E+07 | 1,000E+00 |
| **Q9ZHA1** | Cell division protein;Factor involved in shape determination and osmotic tolerance;YGGT family protein | ylmG;HMPREF0776_ | 7,646E+08 | 1,000E+00 |
| **T1Y4U6** | Capsular polysaccharide synthesis glycosyltransferase CapL;Glycosyltransferase, group 1 family protein | SAKOR_00140;BN1321_50024 | 3,058E+07 | 1,000E+00 |
| **T1Y5C0** | Na+ driven multidrug efflux pump;Multidrug export protein MepA | SAKOR_00330;mepA | 1,487E+08 | 1,000E+00 |
| **T1Y5P6** | ABC transporter permease protein;Methionine ABC transporter, permease component;Binding-protein-dependent transport system | SAKOR_00446;metP | 3,809E+09 | 1,000E+00 |
| **T1Y6U1** | Type VII secretion protein EssA;Protein EssA | SAKOR_00267;essA | 9,639E+07 | 1,000E+00 |
| **T1Y878** | Phosphate-binding protein;Phosphate-binding protein PstS;Phosphate ABC transporter periplasmic protein | SAKOR_01326;pstS | 1,298E+08 | 1,000E+00 |
| **T1Y890** | Peptide deformylase;Peptide deformylase-like | def;MW1098;SAV1215 | 4,605E+07 | 1,000E+00 |
| **T1Y931** | Sodium/proton antiporter protein shaA;Na(+)/H(+) antiporter subunit A1 | SAKOR_00864;mnhA1 | 2,391E+09 | 1,000E+00 |
| **T1Y9Y1** | Multidrug resistance protein B;Quinolone resistance protein NorB | SAKOR_01378;norB | 1,764E+08 | 1,000E+00 |
| **T1YA34** | Phosphatidate cytidylyltransferase | SAKOR_01189;cdsA | 7,362E+08 | 1,000E+00 |
| **T1YC30** | 2-oxoglutarate/malate translocator;2-oxoglutarate translocator;Sodium:sulfate symporter family protein | SAKOR_02696;2486 | 3,905E+09 | 1,000E+00 |
| **T1YDK8** | Integral membrane protein;Putative membrane protein;Membrane protein | SAKOR_02444;BN1321_410038;ERS195423_01356 | 8,225E+07 | 1,000E+00 |
| **U5NW20** | Nickel (Ni2+)/peptide ABC superfamily ATP binding cassette transporter, binding protein | nikA;CO98_1215 | 9,056E+08 | 1,000E+00 |
| **W8TPT7** | Antiporter;Hexose phosphate transporter;Hexose phosphate transport protein | uhpT;QU38_14720 | 1,002E+09 | 1,000E+00 |
| **W8TQV7** | Glycerate kinase;Uncharacterized protein | glxK_2;glxK;MW2356 | 7,704E+07 | 1,000E+00 |
| **W8TR97** | Membrane protein;Uncharacterized protein | AFO87_10600;QU38_10965;HMPREF0769_11511 | 3,444E+09 | 1,000E+00 |
| **W8TSC7** | Lipoprotein;Uncharacterized protein | AFO87_05265;QU38_14280;ERS179246_01053 | 9,520E+08 | 1,000E+00 |
| **W8TTE9** | Short chain dehydrogenase/reductase oxidoreductase;Short-chain dehydrogenase/reductase family oxidoreductase;Short chain deh | cpnA;BN1321_420082 | 2,417E+08 | 1,000E+00 |
| **W8TTV5** | MerR family transcriptional regulator;MW2312 protein | mta;MW2312 | 4,434E+09 | 1,000E+00 |
| **W8TWM6** | Uncharacterised protein;Uncharacterized protein | AFO87_10370;HMPREF0769_12452;ST398NM01_131 | 5,943E+07 | 1,000E+00 |
| **W8U174** | NAD(P)H-dependent oxidoreductase;Putative NAD(P)H-dependent FMN-containing oxidoreductase YwqN | ywqN_2;QU38_10030 | 1,006E+08 | 1,000E+00 |
| **W8U1Z5** | Formate nitrite transporter;Formate/nitrite transporter;Formate/nitrite transporter family protein | focA;QU38_14270;NWMN_02 | 4,353E+08 | 1,000E+00 |
| **W8U3F9** | Cation diffusion facilitator family transporter;Uncharacterized transporter YdbO;Cobalt-zinc-cadmium resistance protein | fieF;ydbO | 3,624E+08 | 1,000E+00 |
| **W8U4M8** | Membrane protein;Uncharacterized protein | AFO87_13210;SAKOR_01901;SAOUHSC_02148 | 1,047E+09 | 1,000E+00 |
| **W8U4P5** | Putative cysteine protease inhibitor (Staphostatin A);Staphostatin A;Uncharacterized protein | sspA_2;HMPREF0769_11261 | 9,862E+07 | 1,000E+00 |
| **W8U5G5** | Iron transporter;Membrane spanning protein;Mn2 and Fe2 transporter | AFO87_10965;ERS092844_01089;ERS365775_01407 | 3,300E+08 | 1,000E+00 |
| **W8U5U5** | 7-carboxy-7-deazaguanine synthase | queE | 1,173E+08 | 1,000E+00 |
| **W8U5Y5** | Inorganic phosphate transporter;Low-affinity inorganic phosphate transporter | pitA;SAV0664 | 3,401E+08 | 1,000E+00 |
| **W8U6S2** | Phosphoesterase | AFO87_08370;BN1321_230075;NWMN_1065 | 1,912E+08 | 1,000E+00 |
| **W8UA48** | D-serine/D-alanine/glycine transporter;Gamma-aminobutyrate permease | aapA;QU38_08460;ydgF | 2,293E+09 | 1,000E+00 |
| **W8UBG3** | Uracil permease;Putative permease | pyrP;ACH32_12935;HMPREF0769_12580 | 1,492E+09 | 1,000E+00 |
| **W8UT95** | DNA mismatch repair protein MutT;MutT domain containing protein | AL078_09265;AFO87_01845;NWMN_0431 | 7,887E+07 | 1,000E+00 |
| **W8UTD9** | Xanthine permease | pbuX;SAKOR_00387 | 1,013E+07 | 1,000E+00 |
| **W8UWY3** | Polysaccharide biosynthesis protein;Putative polysaccharide biosynthesis protein | ytgP_2;BN1326_80287;BN1321_260388 | 6,941E+08 | 1,000E+00 |
| **X5DTA3** | Damage-inducible protein DinB;Uncharacterized protein | AFO87_13955;SA2485;SAKOR_02695 | 1,180E+08 | 1,000E+00 |
| **X5DU72** | Phosphate/phosphite/phosphonate ABC transporter, periplasmic binding family protein;MW0117 protein;SA0138 protein | phnD;MW0117 | 6,504E+08 | 1,000E+00 |
| **X5DYC0** | Glucokinase;SA0305 protein | bglK;SA0305;SAKOR_00310 | 1,012E+08 | 1,000E+00 |
| **X5DZB1** | Urea transporter;MW2205 protein;SA2081 protein | AFO87_01020;MW2205;SA2081 | 1,169E+08 | 1,000E+00 |
| **X5DZU0** | 3-beta hydroxysteroid dehydrogenase;Putative nucleoside-diphosphate-sugar epimerase;NmrA family protein | qorB;BN1321_90012;HM | 1,670E+08 | 1,000E+00 |
| **X5E034** | Acetyltransferase;Acetyltransferase, GNAT family protein | AFO87_01045;ERS093009_01698;NWMN_2181 | 5,114E+07 | 1,000E+00 |
| **X5E195** | Membrane protein;Uncharacterized protein;Uncharacterized protein | AFO87_08905;MW0380;SA0380 | 8,660E+08 | 1,000E+00 |
| **X5E273** | GCN5-related N-acetyltransferase (GNAT);Acetyltransferase, GNAT family;Acetyltransferase, GNAT family protein | AFO87_03445;BN1326_100148; | 2,787E+07 | 1,000E+00 |
| **X5EH84** | 2-amino-4-hydroxy-6-hydroxymethyldihydropteridin e pyrophosphokinase;2-amino-4-hydroxy-6-hydroxymethyldihydropteridine pyr | folK | 8,183E+07 | 1,000E+00 |
| **X5EIW2** | Membrane protein;Putative membrane spanning protein;Putative membrane protein | AFO87_03740;ST398NM01_0108;BN1326_150294 | 3,792E+08 | 1,000E+00 |
| **X5EK76** | Orf1;Uncharacterized protein | AFO87_04135;ASU36_11630;MW2578 | 7,334E+08 | 1,000E+00 |
| **X5ELZ2** | Histidine kinase;MW1790 protein;Two-component sensor protein yhcY;YhcY | vraS_1;MW1790;SAKOR_01805 | 5,600E+07 | 1,000E+00 |
