# Supplementary material for: Flotillin scaffold activity contributes to type VII secretion system assembly in Staphylococcus aureus
Source: PLoS Pathog. 2017 Nov 22;13(11):e1006728. doi: 10.1371/journal.ppat.1006728 (PMC5718613; doi:10.1371/journal.ppat.1006728)
Supplement: S2 Table — (PDF) [file ppat.1006728.s011.pdf]

S2_Table

Strain	Organism	Genotype / plasmid	Reference
		**Wild types**	
BM-176	*S. aureus* RN4220	wild type	Kreiswirth et al. 1983 1
BM-178	*S. aureus* USA300_TCH1516	wild type	Gonzalez et al. 2005
DL-95	*E. coli* DH5α	wild type	Reusch et al. 1986
DL-1124	*E. coli* BL21 DE3 Gold	wild type	Stratagene
BM-263	*E. coli* BTH101	wild type	Euromedex
		**Plasmids**	
BM-257	*E. coli* DH5α	pKT25	Euromedex
BM-258	*E. coli* DH5α	pKNT25	Euromedex
BM-259	*E. coli* DH5α	pUT18	Euromedex
BM-260	*E. coli* DH5α	pUT18C	Euromedex
BM-308	*E. coli* DH5α	pMAD	Arnaud et al. 2004
BM-292	*E. coli* DH5α	pLac	Yepes et al. 2014
BM-485	*E. coli* DH5α	$pAmy_{xyl}$	Yepes et al. 2014
BM-372	*E. coli* DH5α	$pLac_{xyl}$	Yepes et al. 2014
BM-511	*E. coli* DH5α	pSEVA641	Silva-Rocha et al. 2012
BM-261	*E. coli* DH5α	pKT25-*zip*	Euromedex
BM-262	*E. coli* DH5α	pUT18C-*zip*	Euromedex
BM-579	*E. coli* DH5α	pKT25-*floA*	This study
BM-271	*E. coli* DH5α	pKNT25-*floA*	This study
BM-310	*E. coli* DH5α	pUT18-*floA*	This study
BM-577	*E. coli* DH5α	pUT18C-*floA*	This study
BM-543	*E. coli* DH5α	pKT25-*esaA*	This study
BM-503	*E. coli* DH5α	pKNT25-*esaA*	This study
BM-281	*E. coli* DH5α	pUT18-*esaA*	This study
BM-546	*E. coli* DH5α	pUT18C-*esaA*	This study
BM-458	*E. coli* DH5α	pKT25-*essA*	This study
BM-311	*E. coli* DH5α	pKNT25-*essA*	This study
BM-268	*E. coli* DH5α	pUT18-*essA*	This study
BM-299	*E. coli* DH5α	pUT18C-*essA*	This study
BM-327	*E. coli* DH5α	pKT25-*essB*	This study
BM-312	*E. coli* DH5α	pKNT25-*essB*	This study
BM-269	*E. coli* DH5α	pUT18-*essB*	This study
BM-280	*E. coli* DH5α	pUT18C-*essB*	This study
BM-499	*E. coli* DH5α	pKT25-*essC*	This study
BM-500	*E. coli* DH5α	pKNT25-*essC*	This study
BM-270	*E. coli* DH5α	pUT18-*essC*	This study
BM-305	*E. coli* DH5α	pUT18C-*essC*	This study
GK-418	*E. coli* DH5α	pUT18-*ftsA*	Yepes et al. 2014
GK-417	*E. coli* DH5α	pKNT25-*ftsA*	Yepes et al. 2014
BM-514	*E. coli* DH5α	pSEVA641-P_{lac}-*floA*	Koch et al. 2017
BM-424	*E. coli* DH5α	pET20b-*esxA*	This study
BM-425	*E. coli* DH5α	pET20b-*esxB*	This study
BM-456	*E. coli* DH5α	pET20b-*esxC*	This study
BM-461	*E. coli* DH5α	pET20b-*esxD*	This study
DL-1321	*E. coli* DH5α	pET15-*ytnP*	Schneider et al.2012
NM-40	*E. coli* DH5α	pASK-IBA3C-*essB*	This study

BM-614	*E. coli* DH5α	pASK-IBA3C-*floA*	This study
BM-615	*E. coli* DH5α	pBAD-*esaA*essABC	This study
Staphylococcus aureus strains			
BM-199	*S. aureus* USA300_TCH1516	Δ*floA::spc*	This study
BM-493	*S. aureus* USA300_TCH1516	Δ*T7SS* (markerless)	This study
BM-554	*S. aureus* USA300_TCH1516	Δ*essA* (markerless)	This study
BM-516	*S. aureus* USA300_TCH1516	Δ*essB* (markerless)	This study
BM-585	*S. aureus* USA300_TCH1516	Δ*essA* (markerless)<br>*amy::P*$_{xyl}$*-essA-mars*	This study
BM-590	*S. aureus* USA300_TCH1516	Δ*essA* (markerless)<br>*amy::P*$_{xyl}$*-essA-mars*<br>Δ*floA::spc*	This study
BM-426	*S. aureus* USA300_TCH1516	pLac$_{xyl}$-*flag-essB*	This study
BM-460	*S. aureus* USA300_TCH1516	*amy::P*$_{xyl}$*-floA-his*	This study
BM-465	*S. aureus* USA300_TCH1516	*amy::P*$_{xyl}$*-floA-his*<br>pLac$_{xyl}$-*flag-essB*	This study
RW-227	*S. aureus* USA300_TCH1516	Δ*spA::spc*	This study
BM-605	*S. aureus* USA300_TCH1516	pLac$_{xyl}$-*esxA-flag*	This study
BM-606	*S. aureus* USA300_TCH1516	Δ*floA::spc*<br>pLac$_{xyl}$-*esxA-flag*	This study
BM-607	*S. aureus* USA300_TCH1516	Δ*T7SS* (markerless)<br>pLac$_{xyl}$-*esxA-flag*	This study
BM-481	*S. aureus* USA300_TCH1516	pLac$_{xyl}$-*esxB-flag*	This study
BM-482	*S. aureus* USA300_TCH1516	Δ*floA::spc*<br>pLac$_{xyl}$-*esxB-flag*	This study
BM-604	*S. aureus* USA300_TCH1516	Δ*T7SS* (markerless)<br>pLac$_{xyl}$-*esxB-flag*	This study
BM-589	*S. aureus* USA300_TCH1516	Δ*essB* (markerless)<br>*lac::P*$_{esxA}$*-gfp-essB*	This study
BM-593	*S. aureus* USA300_TCH1516	Δ*essB* (markerless)<br>*lac::P*$_{esxA}$*-gfp-essB*<br>Δ*floA::spc*	This study
BM-566	*S. aureus* USA300_TCH1516	Δ*essB* (markerless)<br>*lac::P*$_{xyl}$*-flag-essB*	This study
BM-571	*S. aureus* USA300_TCH1516	Δ*essB* (markerless)<br>*lac::P*$_{xyl}$*-flag-essB*<br>Δ*floA::spc*	This study
BM-586	*S. aureus* USA300_TCH1516	Δ*essB* (markerless)<br>*amy::P*$_{xyl}$*-essA-mars*<br>*lac::P*$_{xyl}$*-flag-essB*	This study
BM-591	*S. aureus* USA300_TCH1516	Δ*essB* (markerless)<br>*amy::P*$_{xyl}$*-essA-mars*<br>*lac::P*$_{xyl}$*-flag-essB*<br>Δ*floA::spc*	This study
BM-400	*S. aureus* USA300_TCH1516	*amy::P*$_{SA1403}$*-floA-mars*	This study

References

Kreiswirth BN, Kravitz GR, Schlievert PM, Novick RP. Nosocomial Transmission of a Strain of *Staphylococcus aureus* Causing Toxic Shock Syndrome. Ann Intern Med. 1986;105: 704–707.

Gonzalez BE, Martinez-Aguilar G, Hultén KG, Hammerman WA, Coss-Bu J, Avalos-Mishaan A, et al. Severe Staphylococcal Sepsis in Adolescents in the Era of Community-Acquired Methicillin-Resistant *Staphylococcus aureus*. Pediatrics. American Academy of Pediatrics; 2005;115: 642–648. doi:10.1542/peds.2004-2300

Reusch RN, Hiske TW, Sadoff HL. Poly-beta-hydroxybutyrate membrane structure and its relationship to genetic transformability in *Escherichia coli*. Journal of Bacteriology. American Society for Microbiology; 1986;168: 553–562. doi:10.1128/jb.168.2.553-562.1986

Arnaud M, Chastanet A, Débarbouillé M. New vector for efficient allelic replacement in naturally nontransformable, low-GC-content, gram-positive bacteria. Appl Environ Microbiol. 2004;70: 6887–6891. doi:10.1128/AEM.70.11.6887-6891.2004

Yepes A, Koch G, Waldvogel A, García-Betancur J-C, López D. Reconstruction of mreB expression in *Staphylococcus aureus* via a collection of new integrative plasmids. Appl Environ Microbiol. 2014;80: 3868–3878. doi:10.1128/AEM.00759-14

Silva-Rocha R, Martínez-García E, Calles B, Chavarría M, Arce-Rodríguez A, las Heras de A, et al. The Standard European Vector Architecture (SEVA): a coherent platform for the analysis and deployment of complex prokaryotic phenotypes. Nucleic Acids Res. Oxford University Press; 2013;41: D666–D675. doi:10.1093/nar/gks1119

Koch G, Wermser C, Acosta IC, Kricks L, Stengel ST, Yepes A, et al. Attenuating *Staphylococcus aureus* virulence by targeting flotillin protein scaffold activity. Cell Chemical Biology. 2017 (in press).

Schneider J, Yepes A, Garcia-Betancur JC, Westedt I, Mielich B, López D. Streptomycin-induced expression in *Bacillus subtilis* of YtnP, a lactonase-homologous protein that inhibits development and streptomycin production in *Streptomyces griseus*. Appl Environ Microbiol. 2012;78: 599–603. doi:10.1128/AEM.06992-11
