# Supplementary material for: Flotillin scaffold activity contributes to type VII secretion system assembly in Staphylococcus aureus
Source: PLoS Pathog. 2017 Nov 22;13(11):e1006728. doi: 10.1371/journal.ppat.1006728 (PMC5718613; doi:10.1371/journal.ppat.1006728)
Supplement: S3 Table — (PDF) [file ppat.1006728.s012.pdf]

Table S3

Purpose	Name	Sequence (5'-3')
	**Markerless deletions**	
For cloning of *essA*-flankings into pMAD	ΔessA_LFH1_SalI	AAAAGTCGACTATAGTTATGAACGTGCCAA
	ΔessA_LFH2	ACTTTTACGTGCTGATTCATTTAGATTAATCTCTCTTTCTTA
	ΔessA_LFH3	TAAGAAAGAGAGATTAATCTAAATGAATCAGCACGTAAAAGT
	ΔessA_LFH4_BamHI	AAAAGGATCCGTATGATTGTCATTAATGTCA
For cloning of *essB*-flankings into pMAD	ΔessB_LFH1_SalI	AAAAGTCGACAAGACAGCAAAGCGGTTAA
	ΔessB_LFH2	TCTTTGCCTCAGTCCTATACTATTTTTCCTCCTATAGTAA
	ΔessB_LFH3	TTACTATAGGAGGAAAAATAGTATAGGACTGAGGCAAAGA
	ΔessB_LFH4_BamHI	AAAAGGATCCTATGATCACCAATGTAAGCT
For cloning of T7SS-flankings into pMAD	ΔT7SS_LFH1_SalI	AAAAGTCGACTACTGATTGTTGTTAAGATCA
	ΔT7SS_LFH2	TTTAGTCTTACATTAAGATAGTAACTAGAAACCTCCTGAATA
	ΔT7SS_LFH3	TATTCAGGAGGTTTCTAGTTACTATCTTAATGTAAGACTAAA
	ΔT7SS_LFH4_BamHI	AAAAGGATCCAAAGATTACACAGTGCAAATA
	**Translational fusions**	
For cloning of *essA-mars* into pAmy$_{xyl}$	EssANheIfw	TTTTGCTAGCGGAAGGAGTTTTTGCTTATGTTGATGAATAGCGTGAT
	EssArv + MARS tail	CATCTTCTGATGATGCCATAATGTTACTTTTACGTGCTG
	MARSfw + EssA tail	CAGCACGTAAAAGTAACATTATGGCATCATCAGAAGATG
	MARSEcoRIrv	AAAAGAATTCTTATCCTGCACCTGTTGAA
For cloning of *flag-essB* into pLac$_{xyl}$	FLAGNheIfw	TTTTGCTAGCGGAAGGAGTTTTTGCTTATGGACTACAAAGACCATGAC
	FLAGrv + EssB tail	AGGGTTATGATTTTTAACCATTTTATCGTCGTCATCTTTGTAG
	EssBfw + FLAG tail	CTACAAAGATGACGACGATAAAATGGTTAAAAATCATAACCCT
	EssBXhoIrv	AAAACTCGAGGCCTCAGTCCTATACTATT
For cloning of *PesxA-gfp-essB* into pLac	PesxABamHIfw	AAAAGGATCCTACTGATTGTTGTTAAGATCA
	PesxArv + GFP tail	AGTTCTTCTCCTTTACTCATAACTAGAAACCTCCTGAATA
	GFPfw + PesxA tail	TATTCAGGAGGTTTCTAGTTATGAGTAAAGGAGAAGAACT
	GFPrv + EssB tail	AGGGTTATGATTTTTAACCATTTTGTATAGTTCATCCATGC
	EssBfw + GFP tail	GCATGGATGAACTATACAAAATGGTTAAAAATCATAACCCT
	EssBSpeIrv	AAAAACTAGTTTGCCTCAGTCCTATACTA
For cloning of *esxA-flag* into pLac$_{xyl}$	EsxANheIfw	TTTTGCTAGCGGAAGGAGTTTTTGCTTATGGCAATGATTAAGATGAG
	EsxArv + FLAG tail	GTCATGGTCTTTGTAGTCTTGCAAACCGAAATTATTAGA
	FLAGfw + EsxA tail	TCTAATAATTTCGGTTTGCAAGACTACAAAGACCATGAC
	FLAGXhoIrv	AAAACTCGAGTTACTATTTATCGTCGTCATC
For cloning of *esxB-flag* into pLac$_{xyl}$	EsxBNheIfw	TTTTGCTAGCGGAAGGAGTTTTTGCTTATGGGTGGATATAAAGGTAT
	EsxBrv + FLAG tail	GTCATGGTCTTTGTAGTCTGGGTTCACCCTATCAAG
	FLAGfw + EsxB tail	CTTGATAGGGTGAACCCAGACTACAAAGACCATGAC
For cloning of *floA-mars* into pAmy-P_{floA}-*floA-yfp*	FloAHindIIIfw	AAAAAAAAGCTTATGTTTAGTTTAAG
	FloArv + MARS tail	CATCTTCTGATGATGCCATATGTTCAGGTGACTCATCATCA
	MARSfw + FloA tail	TGATGATGAGTCACCTGAACATATGGCATCATCAGAAGATG
	MARSBamHIrv	AAAAGGATCCTTATCCTGCACCTGTTGAA
	**Bacterial two hybrid plasmids**	
For cloning of *esaA* into pUT18 and pKNT25	EsaAHindIIIfw	AAAAAAGCTTATGAAAAAGAAAAATTGGATTTA
	EsaAKpnIrv	AAAAGGTACCCGGATTAATCTCTCTTTCTTAAA
For cloning of *esaA* into pUT18C	EsaAKpnIfw	AAAAGGTACCGATGAAAAAGAAAAATTGGATTTA
	EsaASacIrv	AAAAGAGCTCTTAGATTAATCTCTCTTTCTTA
For cloning of *esaA* into pKT25	EsaAXbaIfw	AAAATCTAGACATGAAAAAGAAAAATTGGATTTA
	EsaAKpnIrv	AAAAGGTACCTTAGATTAATCTCTCTTTCTTA
For cloning of *essA* into (all) B2H vectors	EssABamHIfw	AAAAGGATCCCATGTTGATGAATAGCGTGAT
	EssAKpnIrv	AAAAGGTACCCGTCAATGTTACTTTTACGTGCTG
For cloning of *essB* into pUT18 and pKNT25	EssBHindIIIfw	AAAAAAGCTTATGGTTAAAAATCATAACCCT
	EssBBamHIrv	AAAAGGATCCTCTTTTTTTCTTTCAGCTTCTTG

For cloning of *essB* into pUT18C and pKT25	EssB(pUT18)SalIfw	AAAAGTCGACTATGGTTAAAAATCATAACCCT
	EssB(pKT25)PstIfw	AAAACTGCAGGGATGGTTAAAAATCATAACCCT
	EssBBamHIrv	AAAAGGATCCTCTTTTTTTCTTTCAGCTTCTTG
For cloning of *essC* into pUT18	EssCSalfw	AAAAGTCGACATGCATAAATTGATTATAAAATAT
	EssCBamHIrv	AAAAGGATCCTCTTTAAACCATCTAATCTTTTGA
For cloning of *essC* into pUT18C	EssCSalIfw	AAAAGTCGACTATGCATAAATTGATTATAAAATAT
	EssCBamHIrv	AAAAGGATCCCTATTTAAACCATCTAATCTTT
For cloning of *essC* into pKT25	EssCPstIfw	AAAACTGCAGGGATGCATAAATTGATTATAAAATAT
	EssCBamHIrv	AAAAGGATCCTCTTTAAACCATCTAATCTTTTGA
For cloning of *floA* into (all) BATCH vectors	FloABamHIfw	AAAAGGATCCCATGTTTAGTTTAAGTTTTATCG
	FloAKpnIrv	AAAAGGTACCCGATGTTCAGGTGACTCATCA
**Overexpression vectors**		
For cloning of *esxA* into pET20b(+)	EsxANdeIfw	AAAACATATGGCAATGATTAAGATGAG
	EsxAXhoIrv	AAAACTCGAGTTGCAAACCGAAATTATTAGA
For cloning of *esxB* into pET20b(+)	EsxBNdeIfw	AAAACATATGGGTGGATATAAAGGTAT
	EsxBXhoIrv	AAAACTCGAGTGGGTTCACCCTATCAAG
For cloning of *esxC* into pET20b(+)	EsxCNdeIfw	AAAACATATGATGAATTTTAATGATATTGAAAC
	EsxCXhoIrv	AAAACTCGAGATTCATTGCTTTATTAAAATATTC
For cloning of *esxD* into pET20b(+)	EsxDNdeIfw	AAAACATATGATGACGTTGAGTGGAAAAAT
	EsxDXhoIrv	AAAACTCGAGTCCCTCAATATTATAGTAAAG
For cloning of *floA* into pASK-IBA3C	FW_FloA_IBA3C	TAACGAGGGCAAAAAATGTTTAGTTTAAGTTTTATCGT
	RV_FloA_IBA3C	GTGGCTCCAAGCGCTATGTTCAGGTGACTCATCAT
	FW_IBA3C_revPCR	AGCGCTTGGAGCCACCCG
	RV_IBA3C_revPCR	TTTTTGCCCTCGTTATCTAGATTTTTGTCGA
For cloning of pBAD-*esaAessABC*	FW_pBadHisB	TGAAAGCTTGGCTGTTTTGG
	RV_pBAdHisB	GGTTAATTCCTCCTGTTAGCCCA
	FW_operon_pBAD	CAGGAGGAATTAACCATGAAAAAGAAAAATTGGATTTACG
	RV_operon_pBAD	CAAAACAGCCAAGCTTTCATTTGAACCAACGGATTTTC
For cloning of *essB* into pASK-IBA3C	FW_essB_IBA3C	TAACGAGGGCAAAAAATGGTGAAAAACCACAACCC
	RV_essB_IBA3C	GTGGCTCCAAGCGCTTTTTTTGCGTTCGGCTTCC
	FW_IBA3C_revPCR	AGCGCTTGGAGCCACCCG
	RV_IBA3C_revPCR	TTTTTGCCCTCGTTATCTAGATTTTTGTCGA
